# Enantioselective, convergent synthesis of the ineleganolide core by a tandem annulation cascade†
†Electronic supplementary information (ESI) available: Experimental procedures, ^1^H NMR, ^13^C NMR, and IR spectra, X-ray crystallographic data. CCDC 853379, 1061009–1061014 and 1061016. For ESI and crystallographic data in CIF or other electronic format see DOI: 10.1039/c6sc03347d



**DOI:** 10.1039/c6sc03347d

**Published:** 2016-08-17

**Authors:** Robert A. Craig, II, Jennifer L. Roizen, Russell C. Smith, Amanda C. Jones, Scott C. Virgil, Brian M. Stoltz

**Affiliations:** a Warren and Katherine Schlinger Laboratory for Chemistry and Chemical Engineering , Division of Chemistry and Chemical Engineering , California Institute of Technology , Pasadena , California 91125 , USA . Email: stolz@caltech.edu

## Abstract

An enantioselective and diastereoselective approach toward the synthesis of the polycyclic norditerpenoid ineleganolide is disclosed. A palladium-catalyzed enantioselective allylic alkylation is employed to stereoselectively construct the requisite chiral tertiary ether and facilitate the synthesis of a 1,3-*cis*-cyclopentenediol building block. Careful substrate design enabled the convergent assembly of the ineleganolide [6,7,5,5]-tetracyclic scaffold by a diastereoselective cyclopropanation–Cope rearrangement cascade under unusually mild conditions. Computational evaluation of ground state energies of late-stage synthetic intermediates was used to guide synthetic development and aid in the investigation of the conformational rigidity of these highly constrained and compact polycyclic structures. This work represents the first successful synthesis of the core structure of any member of the furanobutenolide-derived polycyclic norcembranoid diterpene family of natural products. Advanced synthetic manipulations generated a series of natural product-like compounds that were shown to possess selective secretory antagonism of either interleukin-5 or interleukin-17. This bioactivity stands in contrast to the known antileukemic activity of ineleganolide and suggests the norcembranoid natural product core may serve as a useful scaffold for the development of diverse therapeutics.

## Abstract 2

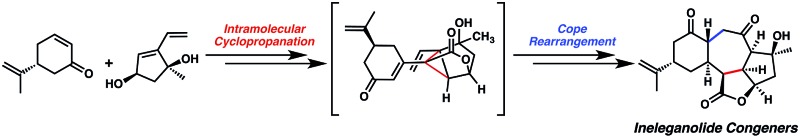
An enantioselective and diastereoselective approach toward the synthesis of the polycyclic norditerpenoid ineleganolide is disclosed.

## Introduction

**Fig. 1:**
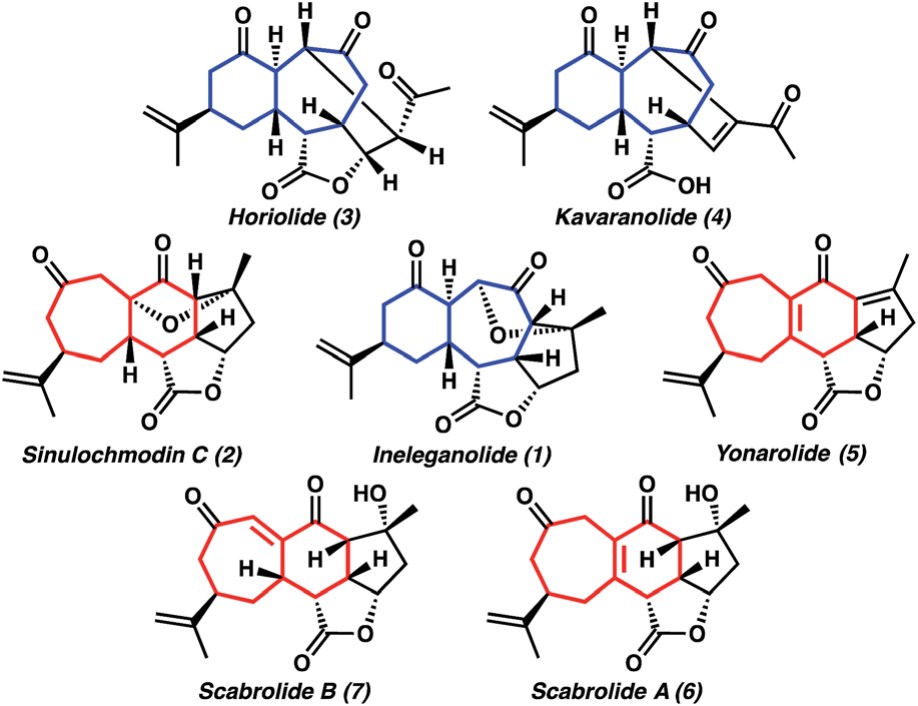
Isomeric [6,7]- and [7,6]-norcembranoid diterpene natural products (blue and red, respectively).

Target-directed synthesis provides an irreplaceable platform for the invention of approaches to not only the structures of interest, but also to previously unknown complex molecules of potential biological importance. Our group has been fascinated with the beautifully complex, highly oxygenated, and compact polycyclic norcembranoid ineleganolide (**1**) since its initial isolation in 1999 (Fig. 1).^1^ Over the following decades, the subsequent isolation of sinulochmodin C (**2**)^2^ and a series of closely related constitutional isomers (**3–7**)^3^ as well as the disclosure of the biological activity of this family of norditerpenoids (*e.g.* antileukemic activity of ineleganolide)^1,3a,d,4^ has fortified our interest in the synthesis of these molecules. Ineleganolide (**1**) poses a particularly formidable synthetic challenge. Characterized by a fused [6,7,5]-carbocyclic core, the natural product is constrained within a highly cupped configuration by a bridging dihydrofuranone ring. The periphery of this rigid polycyclic scaffold is decorated with a network of nine stereogenic centers, eight of which are contiguous.

**Scheme 1:**
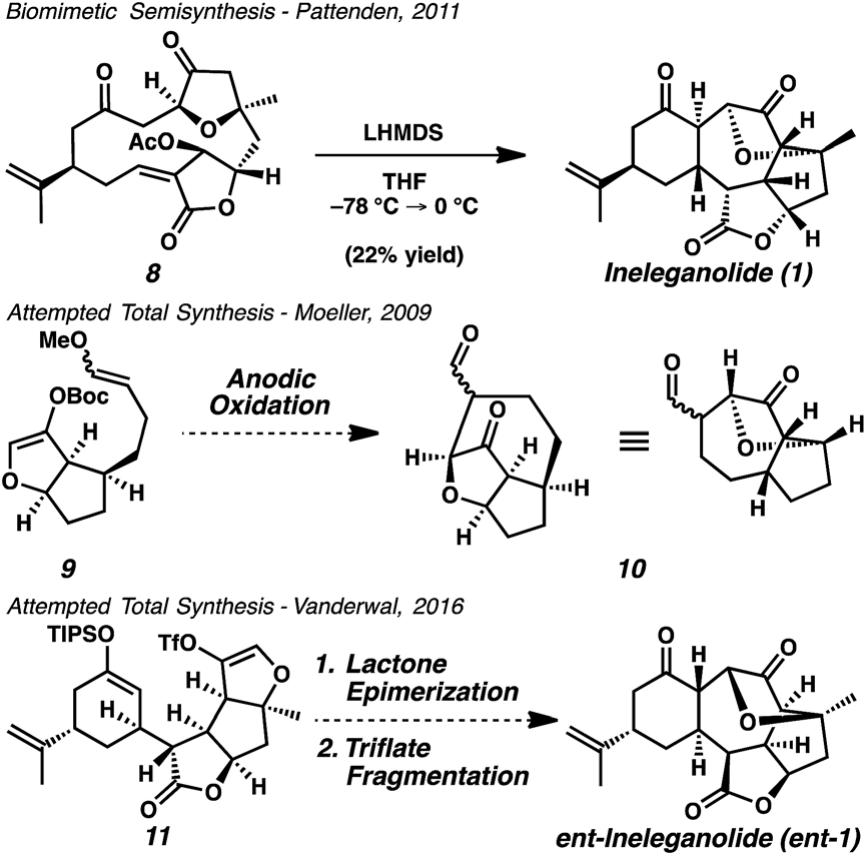
Biomimetic semisynthesis and previous synthetic attempts toward ineleganolide (**1**).

Owing to intricate structural complexity found in these isomeric norditerpenoids, no *de novo* synthetic method exists that enables even the construction of the core scaffolding of any member of the family.^5^ The only laboratory-furnished sample of ineleganolide (**1**) was produced by Pattenden in 2011 through biomimetic semisynthesis *via* transannular anionic cyclization cascade (Scheme 1).^6^ Nicolaou,^7^ Frontier,^8^ and Romo^9^ were unable to construct the carbocyclic core of ineleganolide through the synthetic application of similar cyclization cascades. Moeller sought to forge the ineleganolide skeleton (**10**) through electrochemical macrocyclization, but could not successfully construct the required cycloheptanone.^10^ The Vanderwal group planned to construct the same C–C bond in the final stages of the synthetic route by intramolecular cyclization through a latent oxocarbenium (*i.e.* triflate **11**).^11^ Representing the most advanced synthetic effort toward ineleganolide (**1**) to date, the prerequisite lactone epimerization could not be accomplished despite exhaustive efforts, thwarting the synthetic strategy.

## Results and discussion

**Scheme 2:**
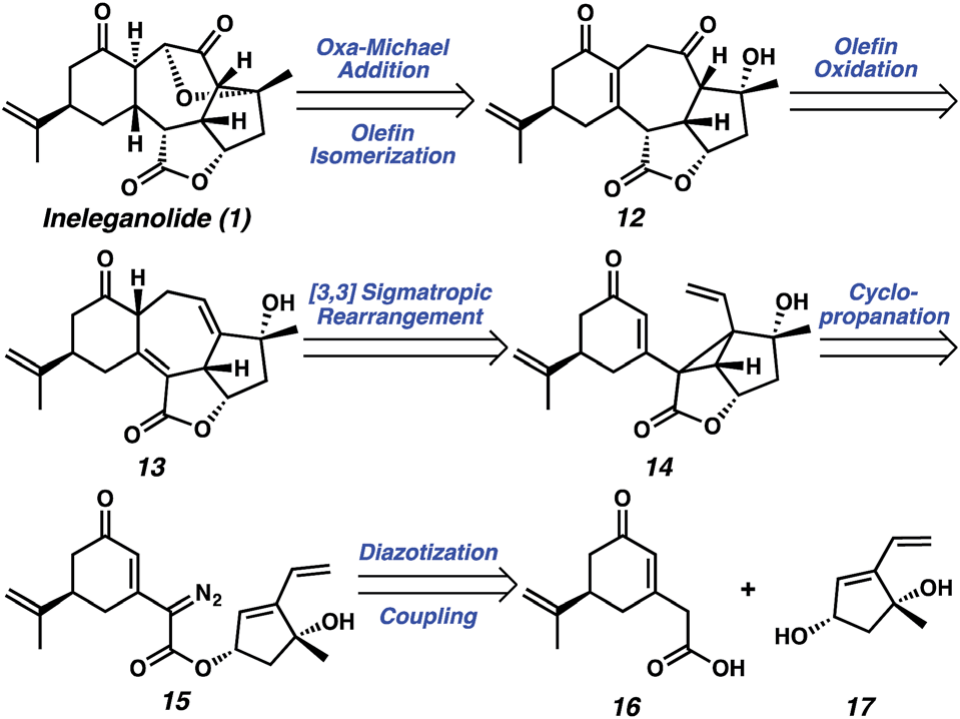
Retrosynthetic analysis of ineleganolide (**1**).

Exploring the retrosynthetic dissection of ineleganolide (**1**) with an eye toward the isomeric norcembranoids, the common 1,3-*cis*-cyclopentanediol moiety became an attractive synthon for the development of a convergent and modifiable synthetic strategy. In contrast to the unsuccessful previous synthetic strategies (*cf.* Scheme 1), which incorporate the dihydrofuranone ring at an early stage, we believed the late-stage introduction of this heterocycle would facilitate construction of the carbocyclic core. Thus, ineleganolide (**1**) was envisioned to arise from enone **12** after formation of the dihydrofuranone ring by oxa-Michael addition (Scheme 2). Enone **12** would be synthesized through selective olefin oxidation of cycloheptadiene **13**. Cope rearrangement of divinylcyclopropane **14** would forge tetracycle **13**.^12^ Intramolecular cyclopropanation of α-diazoester **15** would construct cyclopropane **14**. Cyclization precursor **15** would be assembled by the coupling of carboxylic acid **16** and 1,3-*cis*-cyclopentenediol **17**.

**Scheme 3:**
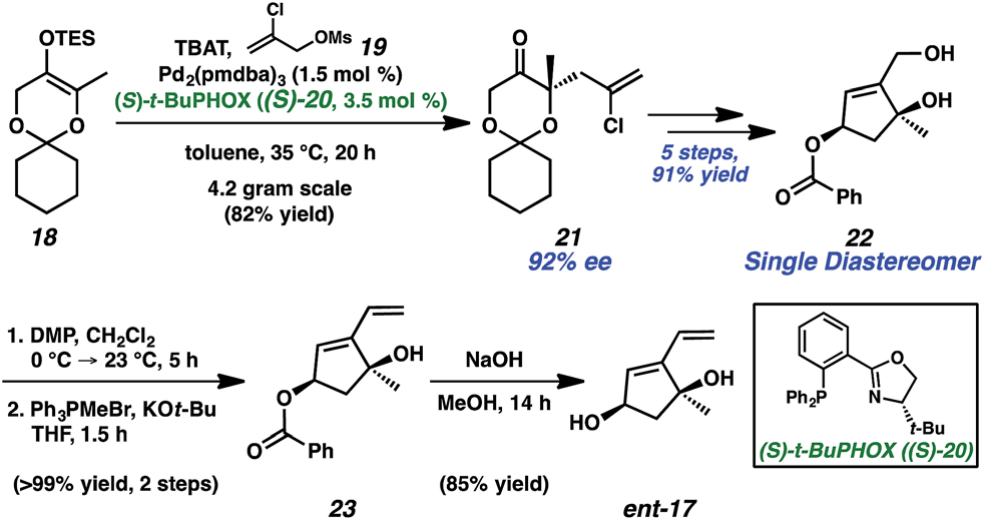
Enantioselective synthesis of 1,3-*cis*-cyclopentenediol coupling partner ***ent*-17**.

Starting with the preparation of diol **17**, we adapted a strategy based on our previously reported efficient and highly enantioselective synthesis of 1,3-*cis*-cyclopentenediol **22** (Scheme 3).^13^ Using a palladium-catalyzed enantioselective allylic alkylation to form the chiral tertiary ether, silyl enol ether **18** was transformed into ketone **21** in 82% yield with 92% enantiomeric excess (ee). Judicious choice of the chiral ligand, the more readily available and cost-effective (*S*)-*t*-BuPHOX (**(*S*)-20**) provided chiral tertiary ether **21** in the (*S*)-configuration. A series of substrate-controlled diastereoselective transformations from ketone **21** then provided diol **22** in five steps, which serves as a building block for norcembranoids in the non-natural enantiomeric series (*cf.* Fig. 1).^14^ Methylenation of diol **22** afforded diene **23** in near-quantitative yield. Ultimately, saponification of ester **23** furnished diol coupling partner ***ent*-17** in a combined 63% yield over 9 steps from enol ether **18**.

**Scheme 4:**
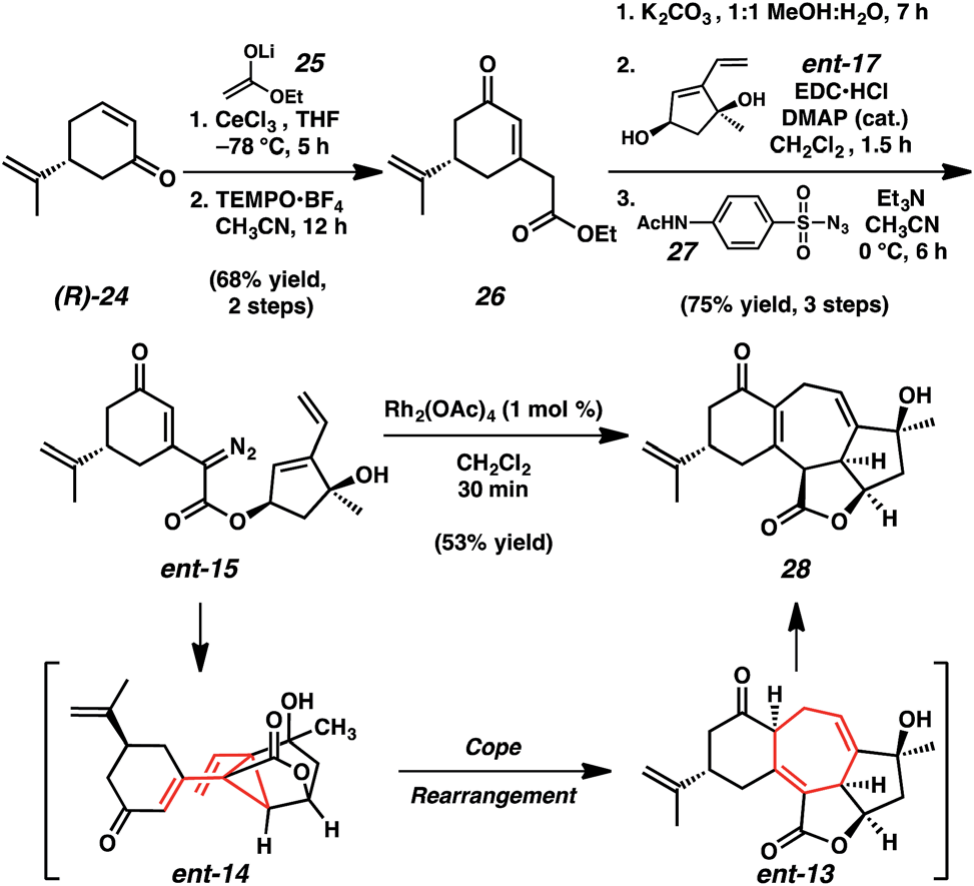
Convergent assembly of the ineleganolide [6,7,5,5]-tetracyclic core.

Construction of the complementary carboxylic acid fragment (***ent*-16**) began with (*R*)-desmethylcarvone (**(*R*)-24**, Scheme 4).^15^ The 1,2-addition of lithium enolate **25** under cerium-mediated reaction conditions followed by oxidative 1,3-allylic transposition of the intermediate allylic alcohol with oxoammonium salt TEMPO·BF_4_ (ref. 16) provided cyclohexenone **26**. Saponification of ethyl ester **26** followed by coupling with diol fragment ***ent*-17** and diazotransfer using *p*-ABSA (**27**) provided the pivotal tandem cyclization cascade precursor, α-diazoester ***ent*-15** in 75% yield over three steps.

Pleasingly, initial attempts to accomplish the planned sequential intramolecular cyclopropanation–Cope rearrangement demonstrated that the transformation proceeded efficiently under mild conditions. Exposure of α-diazoester ***ent*-15** to catalytic dirhodium tetraacetate (1 mol%) in dichloromethane at ambient temperature enabled the construction of cycloheptadiene **28**, containing the complete carbocyclic core of *ent*-ineleganolide (***ent*-1**).^17^ This cyclization cascade is notable given that it is completed under high dilution in less than 20 minutes at ambient temperature and employs an electronically deactivated olefin in the [3,3]-sigmatropic rearrangement. While examples of divinylcyclopropane rearrangements with electron-neutral and electron-rich olefins are abundant,^12,18^ those employing conjugated olefins are limited and typically require forcing conditions.^12c,19^

We speculate that the deleterious effect of an electronically deactivated π-bond in the sequential Cope rearrangement is mitigated by substrate design. Cleavage of highly strained bridging cyclopropane ***ent*-14** upon isomerization to the cycloheptadiene product ***ent*-13** is associated with an increased enthalpic gain compared to a prototypical cyclopropane-fused Cope rearrangement that employs an isolated carbocycle.^20^ Additionally, the unsubstituted vinyl group contributes minimal steric hindrance within the bis-*endo* configuration (*cf.* ***ent*-14**, Scheme 4) and highly organized boat-like transition state required for the *cis*-divinylcyclopropane rearrangement to proceed.^12a,21^

Although the construction of the carbocyclic core of *ent*-ineleganolide (***ent*-1**) proceeds smoothly from α-diazoester ***ent*-15**, no trace of the anticipated diene product (***ent*-13**) was ever detected. Rather, α,β-unsaturated enone **28** was isolated from the reaction mixture as the exclusive product. The 1,3-allylic isomerization is proposed to occur through a base-mediated olefin migration. γ-Deprotonation of α,β-unsaturated ester ***ent*-13** by adventitious acetate and formation of an intermediate conjugated enolate facilitates this process. We hypothesize this isomerization is further aided by a relief of ring strain. These assertions are supported by the observation of an analogous olefin migration under similarly basic reaction conditions.^22^

**Scheme 5:**
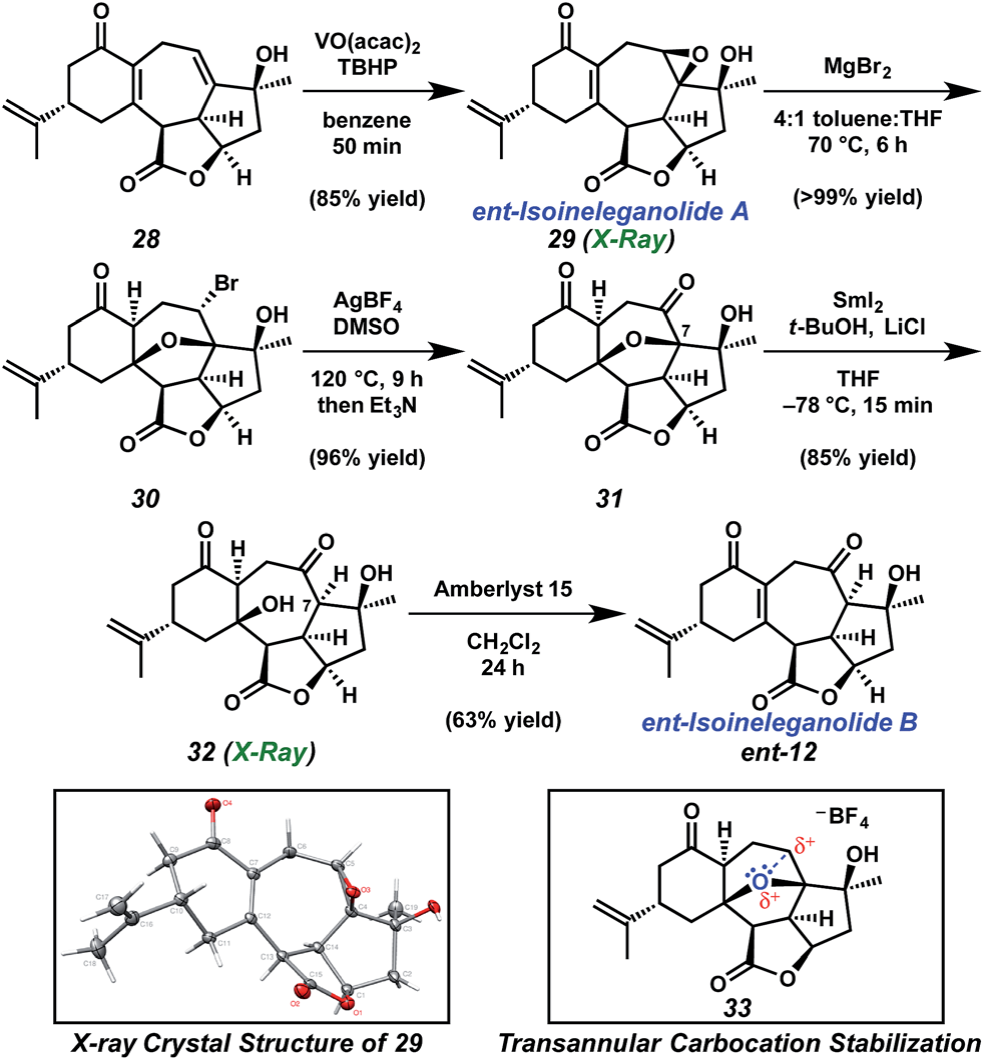
Synthesis of *ent*-isoineleganolides A and B.

Installation of the final requisite atom of *ent*-ineleganolide was accomplished by a hydroxyl-directed epoxidation of tetracycle **28** to provide epoxide **29** in 85% yield as a crystalline white solid, enabling the confirmation of the relative configuration by single crystal X-ray diffraction (Scheme 5). Henceforth known as *ent*-isoineleganolide A, epoxide **29** is the first known synthetic isomer of *ent*-ineleganolide (***ent*-1**) that: (1) contains the full carbocyclic skeleton, (2) possesses all of the required atoms, and (3) has an identical overall oxidation state.

The direct transformation of *ent*-isoineleganolide A (**29**) into enone ***ent*-12** was planned *via syn*-facial 1,2-hydride migration, but this approach proved unsuccessful despite exhaustive investigation.^23^ Instead, alternative access to tetracycle ***ent*-12** was developed through the nucleophilic opening of the epoxide moiety within *ent*-isoineleganolide A (**29**). This ring opening proceeded with concomitant transannular oxa-Michael addition to furnish bromide **30** in near-quantitative yield. Gratifyingly, direct oxidation of the secondary alkyl bromide could be affected through a Kornblum oxidation manifold,^24^ affording diketone **31** in 96% yield.

This efficient transformation stands in stark contrast against canonical examples of oxidation under Kornblum conditions. Substrates are usually limited to primary or benzylic halides; only rare and uniformly low yielding examples of the successful Kornblum oxidation of an unactivated secondary halide are found.^24b,25^ We hypothesize that the bridging furyl oxygen is critical for this transformation, aiding in the abstraction of the secondary bromide and stabilization of the intermediate carbocation (*cf.* **33**, Scheme 5). Indeed, the analog lacking the furyl tether fails to produce any trace of oxidation product under similar conditions.^26^

Pleased to have achieved access to ketopyran **31**, we refocused our attention on advancement toward *ent*-ineleganolide (***ent*-1**). Chemoselective reduction of ketopyran **31** was observed by tuning the reduction potential of samarium(ii) iodide using lithium chloride as an additive.^27^ Under these conditions, *in situ* generated SmCl_2_ cleaves the α-alkoxyketone bond to provide tetracyclic diol **32**. Installation of the hydrogen from the α-face upon protonation of the intermediate Sm-enolate was confirmed by single crystal X-ray diffraction. This stereochemical outcome provides the C(7) configuration found within *ent*-ineleganolide. Selective dehydration of diol **32** under acidic conditions provided the desired enone (***ent*-12**), another non-natural isomer of *ent*-ineleganolide (*ent*-isoineleganolide B).

**Scheme 6:**
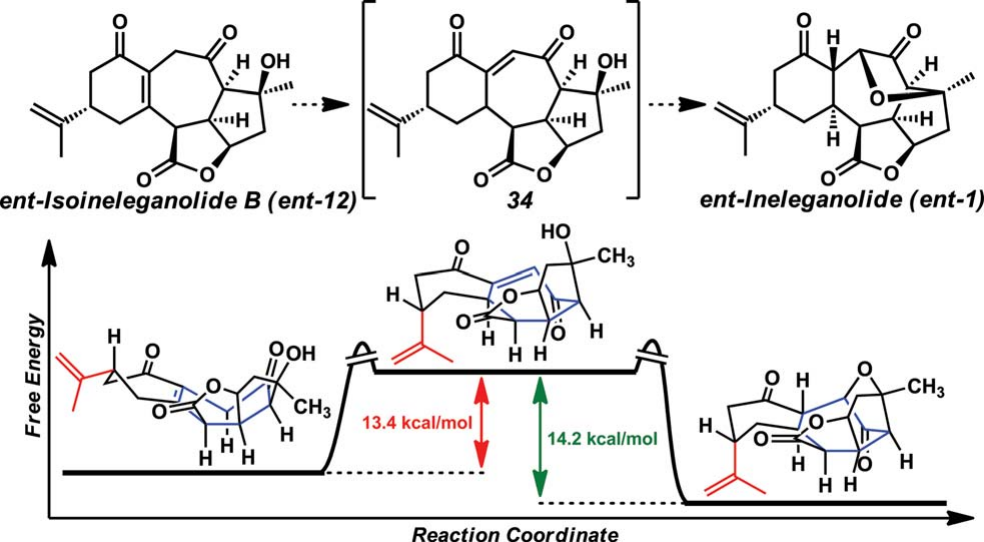
Conformational assessment and relative ground state energies.

Although we were optimistic this isomer would proceed to *ent*-ineleganolide (***ent*-1**) spontaneously by tandem olefin isomerization-oxa-Michael addition, this outcome was not observed. Therefore, with enone ***ent*-12** in hand, this transformation was investigated in a stepwise manner. Despite intensive efforts to accomplish the isomerization of tetrasubstituted enone ***ent*-12** to vinylogous diketone **34** (Scheme 6), no trace of either intermediate **34** or *ent*-ineleganolide (***ent*-1**) was ever detected.^28^ Surprised by the lack of productive reactivity, density functional theory (DFT) was used to explore the thermodynamics of the desired transformation.^29^ *ent*-Isoineleganolide B (***ent*-12**) likely exists in the conformation as shown in Scheme 6 (left).^30^ In this configuration, the isopropenyl group prefers the pseudoequatorial position and the central cycloheptenone is creased, bisecting the molecule. Relative to the ground state energy of this intermediate, the ground state energy of vinylogous diketone **34** is 13.4 kcal mol^–1^ higher in its lowest energy product-like conformation, which posits a pseudoaxial isopropenyl moiety and isomerized cycloheptenone. However, the ground state energy of the natural product (***ent*-1**) in its known configuration^1^ is 14.2 kcal mol^–1^ lower than vinylogous diketone **34** and overall 0.8 kcal mol^–1^ lower in energy than *ent*-isoineleganolide B (***ent*-12**).

The large ground state energy difference between tetra-substituted enone ***ent*-12** and vinylogous diketone **34** in addition to the empirical evidence for the inability to accomplish the desired olefin isomerization in the laboratory suggest the energy barrier for the conversion from enone ***ent*-12** to diketone **34** is experimentally insurmountable. This is likely due to the large conformational shift required for the isomerization event and the reduced enthalpic stability of the conjugated system. The necessary contortion of the central cycloheptenone renders production of the fully conjugated vinylogous diketone moiety unfeasible. The comparative minimum ground state energy of the natural product (***ent*-1**) and the large exergonic difference compared to vinylogous diketone **34** reinforced the use of this intermediate in continued synthetic efforts.

**Scheme 7:**
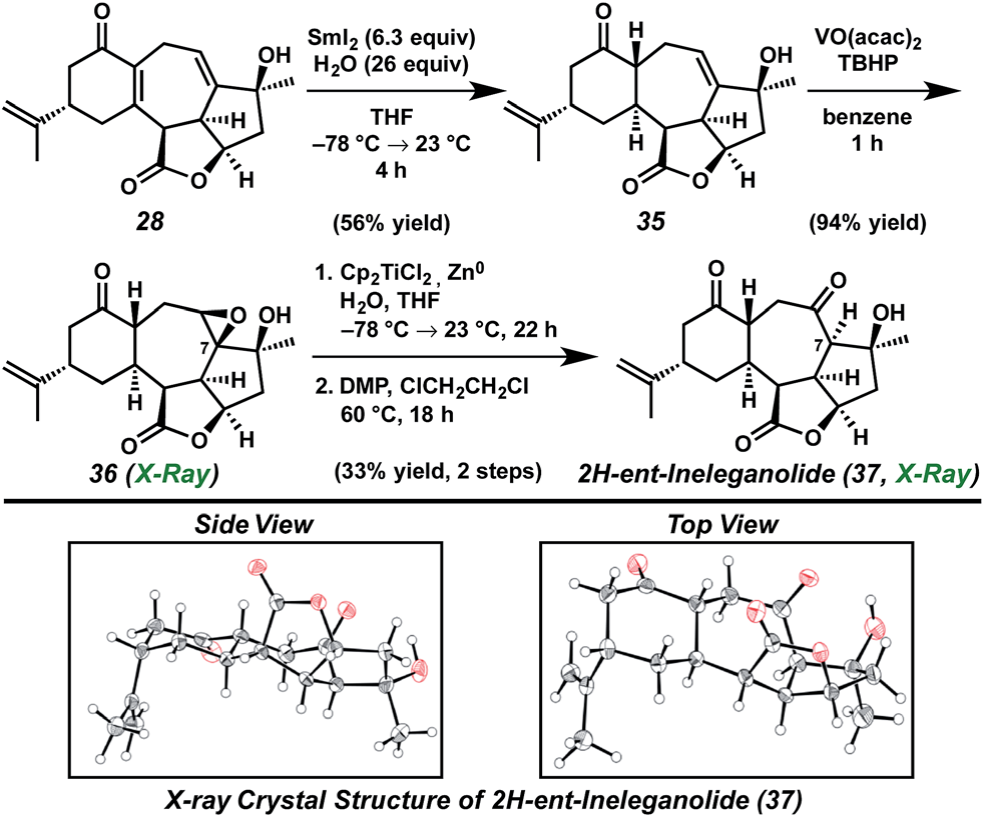
Redox manipulation of diene **28**.

Toward this end, the oxidation state manipulation of carbocyclic diene core **28** was explored (Scheme 7). Although conjugate reduction of enone **28** could not be accomplished using nucleophilic hydride sources, development of samarium(ii) iodide-mediated reaction conditions, including careful control of reaction temperature and sensible selection of additive, enabled diastereoselective conjugate reduction to provide allylic alcohol **35**.^27^ Subsequent hydroxyl-directed epoxidation afforded saturated ketone **36** as a crystalline white solid. The relative configuration of epoxide **36** was established by single crystal X-ray diffraction analysis, confirming the installation of the [6,7]-ring junction in the thermodynamically preferred *trans* configuration identical to that found within *ent*-ineleganolide (***ent*-1**). As anticipated, application of the previously developed three step Kornblum oxidation procedure for the conversion of epoxide **36** to ketone **37** failed in the absence of the transannular furyl bridge (*vide supra*), forcing a strategic revaluation.^26^

In place of a nucleophilic epoxide opening, methods for a radical-mediated reductive epoxide cleavage were investigated. Employing *in situ* generated titanocene(iii) chloride using zinc metal as the optimal reductant, epoxide **36** was opened in regioselective fashion to give the 1,3-diol product.^31^ Elaboration to the cycloheptanone was accomplished by oxidation of the newly revealed secondary alcohol to provide 2*H-ent*-ineleganolide (**37**) in 33% yield in only two steps from epoxide **36**.

The intermediacy of a tertiary radical at C(7) after the C–O bond scission, in analogous fashion to the reductive opening of ketopyran **31** (see Scheme 5), enabled the installation of the hydrogen on the α-face as desired. Single crystal X-ray diffraction served not only to confirm the assignment of this relative configuration, but also revealed the conformational similarities between 2*H-ent*-ineleganolide (**37**) and *ent*-ineleganolide (*cf.* ***ent*-1**, Scheme 6). 2*H-ent*-Ineleganolide (**37**) contains the *trans*-[6,7]-ring junction, with the isopropenyl substituent in the axial position, and all required stereocenters and functional moieties except the dihydrofuranone bridge. Unfortunately, the conversion of 2*H-ent*-ineleganolide (**37**) to *ent*-ineleganolide (***ent*-1**) proved nontrivial. Formation of the final requisite bond through Suárez reaction,^32^ selenoxide elimination, palladium-mediated oxidative desaturation^33^ or by employing either lead(iv) acetate or hypervalent iodine reagents^34^ failed to produce any traces of oxidation at the apical cycloheptanone methylene.

**Fig. 2:**
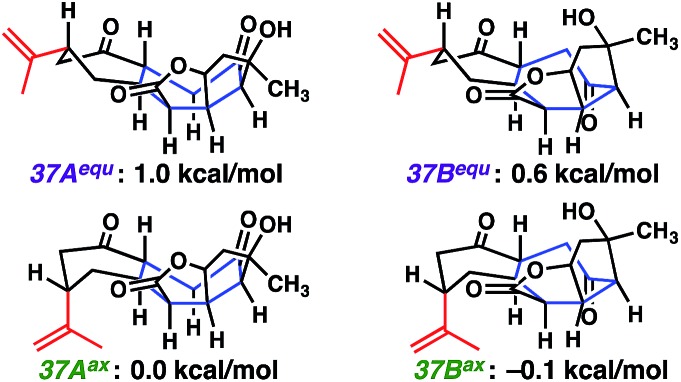
Conformational isomers of 2*H-ent*-ineleganolide.

Surprised by the difficulty of functionalizing the central cycloheptanone within 2*H-ent*-ineleganolide (**37**), we again turned to computational chemistry. The solid-state conformation of 2*H-ent*-ineleganolide (**37A^Ax^**, Fig. 2), determined by single crystal X-ray diffraction, revealed the prototypical conformation encountered throughout our synthetic endeavors (*e.g.* **29**, Scheme 5 and ***ent*-12**, Scheme 6). Conformational isomerization of the isopropenyl moiety into the equatorial position (**37A^Ax^** → **37A^equ^**) increases the ground state energy by 1.0 kcal mol^–1^. Comparatively, the energy minimized conformations **37B^ax^** and **37B^equ^**, in which 2*H-ent*-ineleganolide has adopted the natural product-like configuration within the central cycloheptanone (*cf.* ***ent*-1**, Scheme 6),^1^ are slightly lower. Although the lowest energy conformations of each state (**37A^ax^** *vs.* **37B^ax^**) are equivalent within the error of the computational method, empirical evidence for the difficulty of functionalizing the apical cycloheptanone methylene suggests that there is a large energy barrier that hinders the interconversion between these conformational isomers. Further computational studies are being pursued in order to more completely understand the energetics of the desired transformation.

**Fig. 3:**
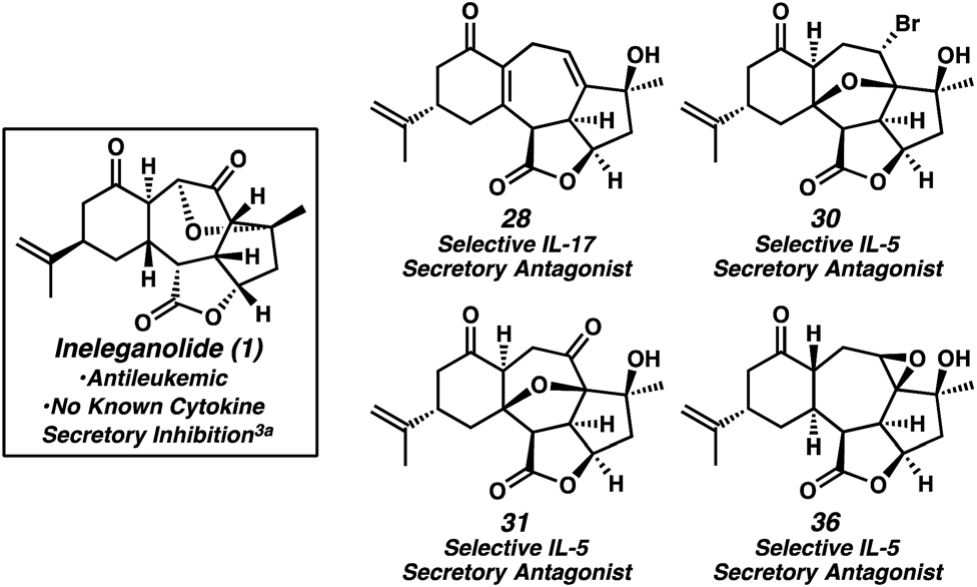
Biological activity of select ineleganoloids.

Throughout the course of these studies, a reasonably large collection of natural-product like compounds was generated. Owing to the known antileukemic properties of ineleganolide, these “ineleganoloids,” in the non-natural enantiomeric series, were evaluated for their activity in DU145 (human prostate cancer) and A2038 (human melanoma) cell viability assays^35^ as well as against other oncological targets (EZH2 (ref. 36) and CD73 (ref. 37)). Although no notable activity toward any oncological target was discovered, the concurrent screening of this library for activity against targets in other therapeutic areas (*i.e.*, neurological, cardiological, autoimmune, and endocrine function) revealed activity of various ineleganoloids.^38^ Diene **28**, bromide **30**, ketopyran **31**, and epoxide **36** were identified as selective interleukin-5 or -17 (IL-5 and IL-17, respectively) secretory inhibitors without significant cytotoxicity (Fig. 3).^39^ Both IL-5 and IL-17 represent attractive pharmaceutical targets considering their pivotal role in autoimmune response and autoimmune disease (*e.g.*, rheumatoid arthritis).^40^ The activity of the ineleganoloids stands in stark contrast with the inability of ineleganolide (**1**) to inhibit cytokine release.^3*a*^ Further investigation of these preliminary results is ongoing and assessment of the biological activity of the ineleganoloids in the natural enantiomeric series will follow in due course.

## Conclusions

In conclusion, an efficient enantioselective and diastereoselective synthetic route to the tetracyclic core of ineleganolide has been disclosed. Convergent assembly of the core scaffold was accomplished by the coupling of two enantioenriched fragments, including a 1,3-*cis*-cyclopentenediol building block common to the polycyclic norcembranoid diterpenes. Tandem intramolecular cyclopropanation–Cope cyclization cascade enabled the diastereoselective construction of the tetracyclic [6,7,5,5]-scaffold of ineleganolide in a single step, providing synthetic access to the core of the polycyclic norcembranoid diterpenes for the first time. Guided by computational data, synthetic advancement facilitated the construction of the first synthetic isomers and analogs of ineleganolide. These natural product-like ineleganoloids advanced the understanding of the conformational restraints influencing chemistry of the highly compact norcembranoid diterpene scaffold and have led to the identification of biologically active ineleganolide analogs.
