# Correction: Enantioselective, convergent synthesis of the ineleganolide core by a tandem annulation cascade†
†Electronic supplementary information (ESI) available: X-ray crystal structure analysis of enone ***ent*-12A**. CCDC 1885720. For ESI and crystallographic data in CIF or other electronic format see DOI: 10.1039/c8sc90236d


**DOI:** 10.1039/c8sc90236d

**Published:** 2019-01-14

**Authors:** Robert A. Craig, Jennifer L. Roizen, Russell C. Smith, Amanda C. Jones, Scott C. Virgil, Brian M. Stoltz

**Affiliations:** a Warren and Katharine Schlinger Laboratory for Chemistry and Chemical Engineering , Division of Chemistry and Chemical Engineering , California Institute of Technology , Pasadena , California 91125 , USA . Email: stolz@caltech.edu

## Abstract

Correction for ‘Enantioselective, convergent synthesis of the ineleganolide core by a tandem annulation cascade’ by Robert A. Craig II *et al.*, *Chem. Sci.*, 2017, **8**, 507–514.



## 

Since publication of the original manuscript, the authors have carried out some additional research and can now unambiguously confirm the reassignment of a few late-stage, intermediate compounds that were incorrectly assigned in the original manuscript. Specifically, they have obtained an X-ray structure of the product of Amberlyst^®^ treatment of the mixture of compounds **32** and **S2** and the product obtained is epimeric at C7. To avoid confusion, this new product is called product ***ent*-12A**. An updated and corrected Scheme 5 is provided below.

**Scheme 1:**
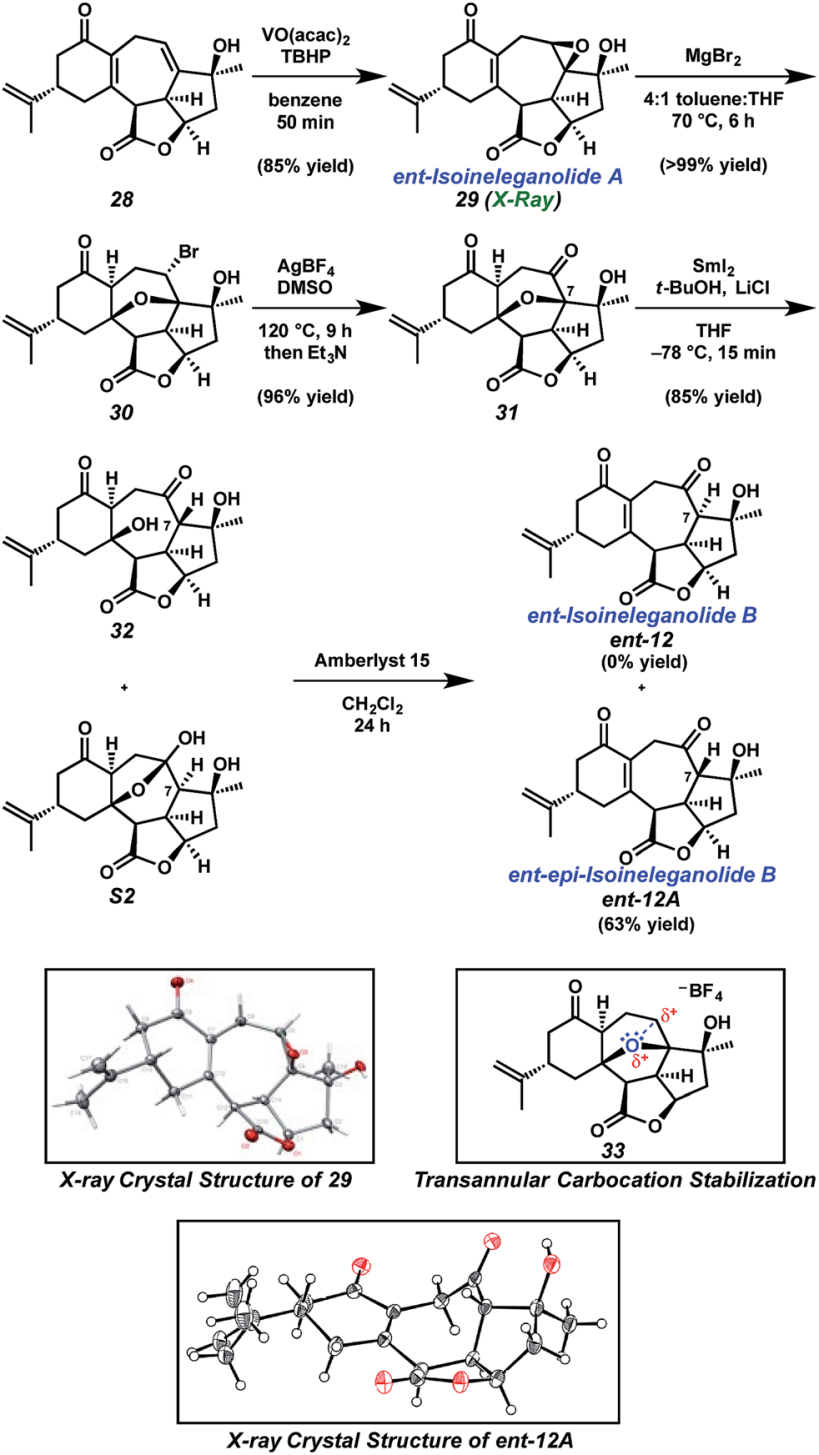
Synthesis of *ent*-isoineleganolide A (**29**) and X-ray crystal structure of *ent-epi*-isoineleganolide B (***ent*-12A**).

Additional Supplementary Information is provided, containing details of the newly solved X-ray crystal structure.

For a complete discussion, please see the authors’ recently published account of their research program toward the enantioselective synthesis of ineleganolide.^1^

The Royal Society of Chemistry apologises for these errors and any consequent inconvenience to authors and readers.

## Supplementary Material

Supplementary information

Crystal structure data
