# Supplementary material for: Enantioselective, convergent synthesis of the ineleganolide core by a tandem annulation cascade
Source: Chem Sci. 2016 Aug 17;8(1):507–14. doi: 10.1039/c6sc03347d (PMC5321630; doi:10.1039/c6sc03347d)
Supplement: Supplementary file 1 [file SC-008-C6SC03347D-s001.pdf]

Electronic Supplementary Material (ESI) for Chemical Science.
This journal is © The Royal Society of Chemistry 2016



**Enantioselective, Convergent Synthesis of the Ineleganolide Core by a Tandem Annulation Cascade**

Robert A. Craig, II, Jennifer L. Roizen, Russell C. Smith,
Amanda C. Jones, Scott C. Virgil, and Brian M. Stoltz*

*Warren and Katharine Schlinger Laboratory for Chemistry and Chemical Engineering, Division of Chemistry and Chemical Engineering, California Institute of Technology, MC 101-20, Pasadena, CA 91125, U.S.A.*

**Table of Contents**

Materials and Methods S2

Experimental Procedures S3

Biological Assay S24
*Table S1. IL-5 and IL-17 Secretory Inhibition of Ineleganoloids* S26

Computational Assessment of Isomerization of Enone ***ent*-12** S27
*Table S2. Calculated Ground State Energies for Enone **ent-12** and Isomers* S28

Computational Assessment of Conformations of 2*H*-*ent*-Ineleganolide (**37**) S29
*Table S3. Calculated Ground State Energies for 2H-ent-Ineleganolide (37)* S29

Notes & References S30

NMR & IR Spectra S32

X-Ray Crystal Structure Analysis of Diene **28** S58

X-Ray Crystal Structure Analysis of *ent*-Isoineleganolide A (**29**) S72

X-Ray Crystal Structure Analysis of Bromide **30** S87

X-Ray Crystal Structure Analysis of Ketopyran **31** S102

X-Ray Crystal Structure Analysis of Hemiketal **S2** S117

X-Ray Crystal Structure Analysis of Epoxide **36** S132

X-Ray Crystal Structure Analysis of 2*H*-*ent*-Ineleganolide **37** S147

**Materials and Methods**

Unless stated otherwise, reactions were performed at ambient temperature (23 °C) in flame-dried or oven-dried glassware under an argon or nitrogen atmosphere using dry, deoxygenated solvents (distilled or passed over a column of activated alumina).[1] Commercially available reagents were used as received. $Et_3N$ was distilled from calcium hydride immediately prior to use. MeOH was distilled from magnesium methoxide immediately prior to use. Hydroxymethyl-*cis*-1,3-cyclopentenediol **22**,[2] TEMPO•$BF_4$,[3] (*R*)-desmethylcarvone (**(*R*)-24**),[4] and *p*-acetamidobenzenesulfonyl azide (*p*-ABSA, **27**)[5] were prepared by known methods. Reactions requiring external heat were modulated to the specified temperatures using an IKAmag temperature controller. Thin-layer chromatography (TLC) was performed using E. Merck silica gel 60 F254 precoated plates (250 nm) and visualized by UV fluorescence quenching, potassium permanganate, or *p*-anisaldehyde staining. Silicycle SiliaFlash P60 Academic Silica gel (particle size 40-63 nm) was used for flash chromatography. $^1$H and $^{13}$C NMR spectra were recorded on a Varian Inova 600 (600 MHz and 151 MHz, respectively), Varian Inova 500 (500 MHz and 126 MHz, respectively), or a Varian Mercury 300 spectrometer (300 MHz and 76 MHz, respectively) and are reported in terms of chemical shift relative to residual $CHCl_3$ (in $CDCl_3$, δ 7.26 and δ 77.16, respectively). Data for $^1$H NMR spectra are reported as follows: chemical shift (δ ppm) (multiplicity, coupling constant (Hz), integration). Infrared (IR) spectra were recorded on a Perkin Elmer Paragon 1000 Spectrometer and are reported in frequency of absorption (cm$^{-1}$). High resolution mass spectra (HRMS) were acquired using an Agilent 6200 Series TOF mass spectrometer with an Agilent G1978A Multimode source in atmospheric pressure chemical ionization

(APCI) or mixed (MultiMode: ESI-APCI) ionization mode or were obtained from the Caltech Mass Spectral Facility using either a JEOL JMS-600H High Resolution Mass Spectrometer in fast atom bombardment (FAB+) or electron ionization (EI+) mode or an LCT Premier XE TOF mass spectrometer equipped with an electrospray ionization source (ES+). Optical rotations were measured on a Jasco P-2000 polarimeter using a 100 mm path length cell at 589 nm.

**Experimental Procedures**

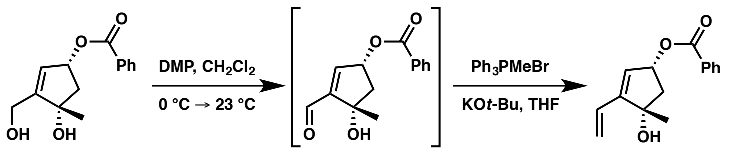


*22* *S1* *23*

**Diene 23**: To a pale yellow solution of diol **22** (241 mg, 0.97 mmol, 1.00 equiv) in $CH_2Cl_2$ (49 mL) at 0 °C (ice/$H_2O$ bath) was added Dess–Martin periodinane (DMP, 823 mg, 1.94 mmol, 2.00 equiv) as a solid in one portion. After 3 h, the off-white heterogeneous reaction mixture was removed from the bath and allowed to warm to ambient temperature (ca. 23 °C). After an additional 2 h, the consumption of starting material was complete as determined by TLC (1:1 EtOAc:Hexanes eluent). The reaction was quenched by the addition of saturated aqueous $Na_2S_2O_3$ (100 mL) in one portion. The biphasic mixture was allowed to stir for 10 minutes and subsequently poured into saturated $NaHCO_3$ (70 mL). The organics were separated and the aqueous layer was extracted with $Et_2O$ (3 x 70 mL). The combined organic layers were washed with brine

(50 mL), dried quickly over $MgSO_4$, filtered, and concentrated in vacuo to provide crude aldehyde **S1**, which was immediately used without further purification.

To a round-bottom flask in an $N_2$-filled glovebox were charged $Ph_3PMeBr$ (1.040 g, 2.91 mmol, 3.00 equiv) and KO*t*-Bu (294 mg, 2.62 mmol, 2.70 equiv) as solids followed by THF (97 mL). The bright yellow reaction mixture was then sealed with a rubber septum, removed from the glovebox, and placed under an argon atmosphere with stirring. After 2 h, a solution of crude aldehyde **S1** in THF (3.00 mL) was added dropwise, causing the reaction mixture to become dark orange-brown. After 1.5 h, the consumption of starting material was complete as determined by TLC (3:7 EtOAc:Hexanes eluent). The reaction was poured onto a mixture of $H_2O$ (90 mL) and $Et_2O$ (30 mL). The organics were separated and the aqueous layer was extracted with $Et_2O$ (3 x 30 mL). The combined organic layers were washed with brine (30 mL), dried over $MgSO_4$, filtered, and concentrated in vacuo. The crude dark brown residue was purified by silica gel column chromatography (40% $Et_2O$ in hexanes eluent) to afford diene **23** (236 mg, >99% yield) as a pale yellow oil: $R_f$ = 0.30 (2:3 $Et_2O$:Hexanes eluent); $^1H$ NMR ($CDCl_3$, 500 MHz) δ 8.04–7.99 (m, 2H), 7.56–7.51 (m, 1H), 7.41 (dddd, *J* = 7.6, 6.8, 1.5, 0.9 Hz, 2H), 6.35 (ddt, *J* = 17.8, 11.3, 0.7 Hz, 1H), 5.92 (d, *J* = 2.3 Hz, 1H), 5.80 (ddt, *J* = 17.8, 1.5, 0.6 Hz, 1H), 5.75–5.70 (m, 1H), 5.31 (ddd, *J* = 11.4, 1.6, 0.7 Hz, 1H), 2.73 (dd, *J* = 14.0, 7.3 Hz, 1H), 2.26 (dq, *J* = 6.6, 3.9, 3.0 Hz, 1H), 2.16 (ddd, *J* = 14.0, 4.7, 0.7 Hz, 1H), 1.48 (s, 3H); $^{13}C$ NMR ($CDCl_3$, 126 MHz) δ 166.5, 151.2, 133.1, 130.3, 129.7, 129.1, 128.4, 127.0, 119.2, 81.1, 76.0, 49.3, 26.9; IR (Neat Film, NaCl) 3447, 2973, 1714, 1451, 1355, 1315, 1271, 1177, 1111, 1070, 1026, 954, 858, 712 $cm^{-1}$;

HRMS (APCI) *m/z* calc'd for $C_{15}H_{15}O_2$ $[M–OH]^+$: 227.1067, found 227.1064; $[\alpha]_D^{25.0}$ +126.9° (*c* 3.850, $CHCl_3$).

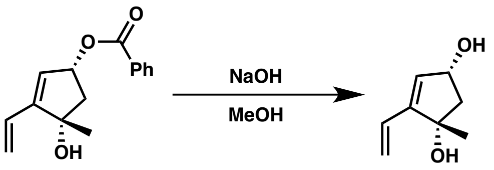


***23*** ***ent-17***

**Diol *ent*-17**: To a pale yellow solution of diene **23** (2.04 g, 8.33 mmol, 1.00 equiv) in distilled MeOH (167 mL) was added NaOH (16.7 mmol, 2.00 equiv) as a 0.50 M solution in distilled MeOH quickly dropwise over 5 mintues. After 14 h, the consumption of starting material was complete as determined by TLC (2:3 $Et_2O$:Hexanes eluent). The reaction was then concentrated to less than one-half of the original volume (ca. 70 mL) and then poured onto $H_2O$ (150 mL). This homogeneous aqueous mixture was then extracted with 1:1 $CHCl_3$:*i*-PrOH (5 x 200 mL). The combined organic layers were dried over $Na_2SO_4$ for 1 h, filtered, and concentrated in vacuo. The crude off-white solid was then adsorbed onto Celite (6.0 g) and purified by silica gel column chromatography (75% EtOAc in hexanes eluent) to afford diol ***ent*-17** (1.11 g, 85% yield) as an amorphous white solid: $R_f$ = 0.13 (1:5 EtOAc:$CH_2Cl_2$); $^1$H NMR (300 MHz, $CDCl_3$) δ 6.30 (dd, *J* = 17.8, 11.3, 1H), 5.82 (d, *J* = 2.1, 1H), 5.73 (dd, *J* = 17.9, 1.7, 1H), 5.26 (dd, *J* = 11.2, 1.7, 1H), 4.65 (dd, *J* = 11.5, 5.1, 1H), 2.54 (dd, *J* = 13.6, 6.8, 1H), 1.85 (app dd, *J* = 13.6, 4.8, 2H), 1.68 (d, *J* = 6.6, 1H), 1.40 (s, 3H); $^{13}$C NMR (76 MHz, $CDCl_3$) δ 149.5, 131.3, 129.5, 118.6, 81.3, 73.0, 53.1, 26.8; IR (Neat film, NaCl) 3287, 3252, 2968, 2930, 2873, 1587, 1481, 1445, 1370, 1341, 1316, 1124, 1088, 1056, 1032, 987, 945, 926 cm$^{-1}$; HRMS

(EI+) *m/z* calc'd for $C_8H_{12}O_2$ $[M•]^+$: 140.0837, found 140.0859; $[\alpha]_D^{25.0}$ +73.4° (*c* 0.600, MeOH).

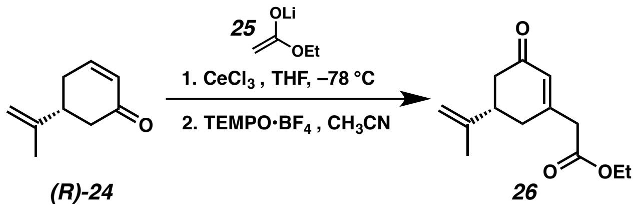


**Ethyl Ester 26**: To a flame-dried 250 mL round-bottom flask in an nitrogen-filled glovebox was charged anhydrous $CeCl_3$ (3.60 g, 14.6 mmol, 2.00 equiv). The flask was seal with a rubber septum, removed from the glovebox, placed under vacuum, and heated in an oil bath to 140 °C with vigorous stirring.[6] After 12 h the flask was removed from the oil bath, allowed to cool to ambient temperature (ca. 23 °C), placed under an atmosphere of argon, and charged with THF (49 mL). After 3.5 h, the reaction was cooled to –78 °C (*i*-PrOH/dry ice bath). (*R*)-Desmethylcarvone ((***R*)-24**, 994 mg, 7.30 mmol) was then added as a solution in THF (7.3 mL) and stirred for 1 h. Simultaneously, in a separate flask, to a solution of LDA (0.80 M in THF, 2.22 equiv) at –78 °C was added anhydrous EtOAc (1.47 mL, 15.0 mmol, 2.06 equiv) as a solution in THF (10.0 mL) dropwise, forming a solution of **25**. After 40 m, to the solution of enone **(*R*)-24** was added the solution of metal enolate **25** dropwise via cannula transfer with an overpressure of argon over 1 h. Exactly 3 h after the completion of addition, the reaction was quenched at temperature with saturated $NH_4Cl$ (24 mL) and warmed slowly to ambient temperature (ca. 23 °C) overnight. The reaction mixture was then filtered through a Celite plug, washing with 100% $Et_2O$. To the resulting solvent mixture was added $H_2O$ (80 mL) and the aqueous layer was then extracted with $Et_2O$ (2 x 120 mL). The combined organic

layers were washed with brine (60 mL), dried over $MgSO_4$, filtered, and concentrated in vacuo. The resultant crude orange-brown oil (1.64 g, >99% yield) was carried on without further purification.

To a solution of intermediate allylic alcohol (408 mg, 1.82 mmol) in $CH_3CN$ (18 mL) was added TEMPO•$BF_4$ (664 mg, 2.73 mmol, 1.5 equiv) as a solid in one portion with stirring. Consumption of starting material was complete after 12 h, as determined by TLC (3:2 $Et_2O$:Hexanes eluent), and the reaction was diluted with $Et_2O$ (125 mL), washed with $H_2O$ (20 mL), brine (20 mL), dried over $MgSO_4$, filtered, and concentrated in vacuo. The crude orange-red oil was purified by silica gel column chromatography (40% $Et_2O$ in hexanes eluent) to afford cyclohexenone ester **26** (275 mg, 68% yield) as an orange-tan oil: $R_f$ = 0.26 (3:2 $Et_2O$:Hexanes eluent); $^1$H NMR (500 MHz, $CDCl_3$) δ 5.93 (s, 1H), 4.79 (s, 1H), 4.75 (s, 1H), 4.14 (q, *J* = 7.1 Hz, 2H), 3.22 (s, 2H), 2.68 (ddd, *J* = 18.2, 9.5, 4.5 Hz, 1H), 2.49 (ddd, *J* = 16.3, 3.7, 1.1 Hz, 1H), 2.46–2.33 (m, 2H), 2.33–2.24 (m, 1H), 1.73 (s, 3H), 1.24 (t, *J* = 7.1 Hz, 3H); $^{13}$C NMR (126 MHz, $CDCl_3$) δ 199.1, 169.2, 156.3, 146.0, 128.3, 110.8, 61.2, 43.2, 42.0, 41.7, 34.7, 20.4, 14.0; IR (Neat Film, NaCl) 2979, 1735, 1672, 1415, 1369, 1329, 1294, 1248, 1176, 1029, 891 cm$^{-1}$; HRMS (MM: ESI-APCI) *m/z* calc'd for $C_{13}H_{19}O_3$ $[M+H]^+$: 223.1329, found 223.1326; $[\alpha]_D^{25.0}$ +40.3° (*c* 3.400, $CHCl_3$).

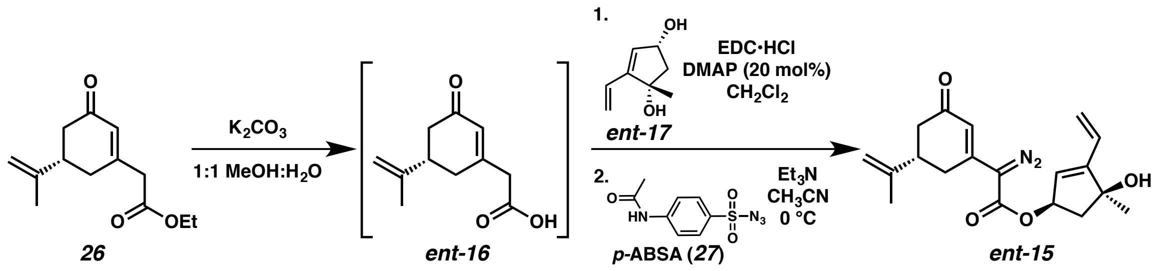


**Diazoester *ent*-15**: To a stirred solution of ethyl ester **26** (2.07 g, 9.32 mmol, 1.00 equiv) in MeOH (31 mL) and $H_2O$ (31 mL) was added $K_2CO_3$ (5.16 g, 37.3 mmol, 4.00 equiv). After 7 h, the consumption of starting material was complete as determined by TLC (2:3 $Et_2O$:Hexanes eluent). The reaction mixture was cooled to 0 °C (ice/$H_2O$ bath) and the pH was adjusted to between 1 and 2 by the careful addition of aqueous 1 N HCl (CAUTION: Vigorous gas evolution!). The reaction mixture was then poured onto a mixture of EtOAc (200 mL) and $H_2O$ (100 mL). The organics were separated and the aqueous layer was extracted with EtOAc (3 x 200 mL). The combined organics were dried over $Na_2SO_4$, filtered and concentrated in vacuo. The crude dark orange oil of carboxylic acid ***ent*-16** (1.81 g, >99% yield) was carried on without further purification.

To a stirred solution of diol ***ent*-17** (119 mg, 0.85 mmol, 1.00 equiv) in $CH_2Cl_2$ (28 mL) were added a portion of crude carboxylic acid ***ent*-16** (330 mg, 1.70 mmol, 2.00 equiv) and EDC•HCl (326 mg, 1.70 mmol, 2.00 equiv). The orange reaction mixture was cooled to 0 °C (ice/$H_2O$ bath) at which time DMAP (21 mg, 0.17 mmol, 0.20 equiv) was added in a single portion. After 30 minutes, the dark red-orange reaction mixture was removed from the cooling bath and allowed to warm to ambient temperature (ca. 23 °C). After 1 h, the consumption of starting material was complete as determined by TLC (3:1 EtOAc:Hexanes eluent). The reaction was quenched by the addition of 0.50 N HCl (8.0 mL) quickly dropwise with vigorous stirring. After 10 minutes, the heterogeneous

solution was poured onto a mixture of EtOAc (100 mL) and $H_2O$ (40 mL). The organics were separated and washed with 0.50 N HCl (20 mL) followed by 5 wt % $K_2CO_3$ (3 x 30 mL), brine (30 mL), and saturated $NH_4Cl$ (30 mL). The organics were then dried over $MgSO_4$, filtered, and concentrated in vacuo. The crude dark brown-orange oil of intermediate ester (269 mg, 0.85 mmol, >99% yield) was carried on without further purification.

Additionally, the combined $K_2CO_3$ washes were cooled to 0 °C (ice/$H_2O$ bath) and the pH was adjusted to between 1 and 2 by the careful addition of aqueous 1 N HCl (CAUTION: Vigorous gas evolution!). The aqueous mixture was extracted with EtOAc (4 x 50 mL). The combined organics were dried over $Na_2SO_4$, filtered and concentrated in vacuo providing a recovered portion (60 mg) of excess carboxylic acid ***ent*-16**.

To a solution of crude ester (269 mg, 0.85 mmol, 1.00 equiv) in $CH_3CN$ (8.5 mL) in the dark was added *p*-acetamidobenzenesulfonyl azide (*p*-ABSA, **27**, 226 mg, 0.94 mmol, 1.10 equiv) as a solid in one portion. The dark orange homogeneous reaction mixture was cooled to 0 °C (ice/$H_2O$ bath). $Et_3N$ (0.36 mL, 2.55 mmol, 3.00 equiv) was then added slowly dropwise. After 6 h, the consumption of starting material was complete as determined by TLC (1:4 EtOAc:$CH_2Cl_2$ eluent). The reaction was quenched by the addition of EtOAc (20 mL), removed from the cooling bath, and allowed warm to ambient temperature (ca. 23 °C). The reaction mixture was then concentrated in vacuo. The crude tan solid was the adsorbed onto Celite (2.0 g) and purified by silica gel column chromatography (20% EtOAc in $CH_2Cl_2$ eluent) to afford diazoester ***ent*-15** (218 mg, 75% yield from diol ***ent*-17**) as a dark yellow oil: $R_f$ = 0.26 (1:4 EtOAc:$CH_2Cl_2$ eluent); $^1$H NMR ($CDCl_3$, 600 MHz) δ 6.39 (d, *J* = 2.0 Hz, 1H), 6.32 (dd, *J* = 17.8, 11.4 Hz, 1H),

5.82 (s, 1H), 5.78 (s, 1H), 5.62 (ddd, *J* = 7.2, 4.8, 2.2 Hz, 1H), 5.34 (dd, *J* = 11.4, 1.6 Hz, 1H), 4.86 (t, *J* = 1.4 Hz, 1H), 4.80 (s, 1H), 2.78–2.71 (m, 1H), 2.70–2.64 (m, 2H), 2.53 (ddd, *J* = 16.1, 3.7, 1.4 Hz, 1H), 2.43 (ddd, *J* = 17.4, 10.9, 2.2 Hz, 1H), 2.34 (dd, *J* = 16.3, 13.0 Hz, 1H), 2.05 (dd, *J* = 14.0, 4.8 Hz, 1H), 1.78 (s, 3H), 1.45 (s, 3H); $^{13}$C NMR ($CDCl_3$, 126 MHz) δ 197.2, 162.5, 151.7, 146.6, 145.9, 128.9, 126.0, 120.2, 119.6, 111.5, 80.8, 76.9, 67.3, 49.2, 41.8, 41.6, 31.7, 27.1, 20.5; IR (Neat Film, NaCl) 3406, 2971, 2102, 1708, 1645, 1579, 1377, 1328, 1250, 1222, 1140, 1061, 992, 952, 893, 744 cm$^{-1}$; HRMS (FAB+) *m/z* calc'd for $C_{19}H_{23}O_4N_2$ [M+H]$^+$: 343.1659, found 343.1634; $[\alpha]_D^{25.0}$ +233.5° (*c* 5.470, $CHCl_3$).

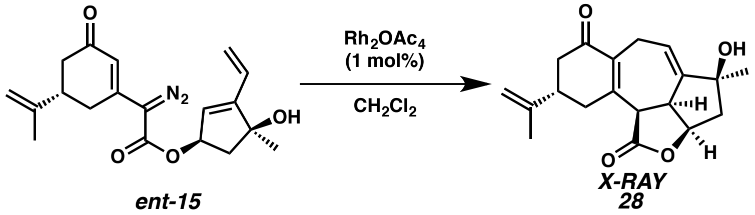


**Diene 28**: To a stirred solution of diazoester ***ent*-15** (630 mg, 1.84 mmol, 1.00 equiv) in $CH_2Cl_2$ (184 mL) in an nitrogen-filled glovebox was added $Rh_2OAc_4$ (8 mg, 0.018 mmol, 0.01 equiv) at ambient temperature (ca. 30 °C). After 30 minutes, the consumption of starting material was complete as determined by TLC (1:4 EtOAc:$CH_2Cl_2$ eluent). The reaction mixture was then concentrated in vacuo and the yellow solid was purified by silica gel column chromatography (20% EtOAc in $CH_2Cl_2$ eluent) to afford diene **28** (306 mg, 53% yield) as a crystalline pale yellow solid. Colorless, translucent X-ray quality crystals were obtained by slow diffusion of pentane into a solution of diene **28** in $Et_2O$, mp: 150–153 °C: $R_f$ = 0.38 (1:4 EtOAc:$CH_2Cl_2$ eluent); $^1$H NMR ($CDCl_3$, 500 MHz) δ 6.19 (dt, *J* = 8.6, 3.5 Hz, 1H), 4.81 (dd, *J* = 4.5, 3.9 Hz, 1H), 4.76 (td, *J* = 1.4, 0.7 Hz,

1H), 4.67 (td, *J* = 1.3, 0.7 Hz, 1H), 3.83–3.77 (m, 1H), 3.63–3.52 (m, 2H), 3.32–3.21 (m, 1H), 2.74–2.64 (m, 2H), 2.61 (ddd, *J* = 16.5, 4.0, 1.7 Hz, 1H), 2.44 (d, *J* = 15.5 Hz, 1H), 2.29 (dd, *J* = 16.5, 12.6 Hz, 1H), 2.15–2.06 (m, 1H), 1.95 (dd, *J* = 15.4, 4.0 Hz, 1H), 1.71 (dt, *J* = 1.3, 0.6 Hz, 3H), 1.38 (s, 3H); $^{13}$C NMR ($CDCl_3$, 126 MHz) δ 198.2, 172.9, 149.2, 148.8, 146.3, 133.5, 128.1, 110.5, 82.2, 78.1, 49.6, 47.0, 45.9, 42.7, 40.0, 36.4, 27.8, 22.6, 20.8; IR (Neat Film, NaCl) 3435, 2923, 2853, 1761, 1661, 1443, 1377, 1263, 1148, 1106 cm$^{-1}$; HRMS (FAB+) *m/z* calc'd for $C_{19}H_{23}O_4$ $[M+H]^+$: 315.1596, found 315.1608; $[\alpha]_D^{25.0}$ +39.6° (*c* 0.680, $CHCl_3$).

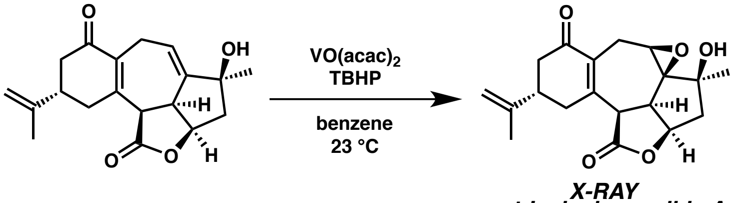


28

ent-Isoineleganolide A
29

***ent*-Isoineleganolide A (29)**: To a pale yellow stirred solution of diene **28** (100 mg, 0.32 mmol, 1.00 equiv) in a vial open to air in benzene (10.7 mL) was added $VO(acac)_2$ (0.9 mg, 0.0032 mmol, 0.01 equiv). After 5 minutes, to this dark green solution was added *t*-butyl hydroperoxide (TBHP, 72 μL, 0.036 mmol, 1.10 equiv) as a 5 M solution in decane dropwise causing the reaction to immediately become deep ruby red. After 45 minutes, the reaction had lost all red color and become pale yellow. The consumption of starting material was complete as determined by TLC (1:4 EtOAc:$CH_2Cl_2$ eluent). The reaction was concentrated in vacuo and the crude tan solid was purified by silica gel column chromatography (25% EtOAc in $CH_2Cl_2$ eluent) to afford epoxide **29** (89 mg, 89% yield) as a white crystalline solid. Colorless, translucent X-ray quality crystals were obtained by

slow diffusion of 1% benzene in heptane into a solution of epoxide **29** in EtOAc, mp: 272–275 °C: $R_f$ = 0.22 (1:4 EtOAc:$CH_2Cl_2$ eluent); $^1$H NMR ($CDCl_3$, 600 MHz) δ 4.84–4.80 (m, 2H), 4.74 (s, 1H), 3.75 (dd, *J* = 19.1, 6.1 Hz, 1H), 3.50–3.46 (m, 1H), 3.42–3.35 (m, 2H), 3.26 (ddd, *J* = 17.3, 3.9, 2.0 Hz, 1H), 2.78 (ddt, *J* = 14.3, 10.7, 3.9 Hz, 1H), 2.66 (ddd, *J* = 16.6, 3.9, 1.9 Hz, 1H), 2.48 (dt, *J* = 19.1, 2.0 Hz, 1H), 2.41 (m, 1H), 2.36 (m, 1H), 2.27 (dd, *J* = 16.5, 13.4 Hz, 1H), 2.10 (ddd, *J* = 17.3, 11.0, 3.8 Hz, 1H), 1.76 (d, *J* = 1.3 Hz, 3H), 1.35 (s, 3H); $^{13}$C NMR ($CDCl_3$, 126 MHz) δ 198.8, 172.1, 148.7, 146.4, 129.9, 110.6, 79.9, 75.0, 70.2, 54.4, 50.2, 45.8, 43.6, 42.6, 39.8, 37.3, 26.7, 22.4, 20.9; IR (Neat Film, NaCl) 3479, 2965, 1767, 1647, 1625, 1369, 1233, 1154, 1102, 992, 975, 907 cm$^{-1}$; HRMS (FAB+) *m/z* calc'd for $C_{19}H_{23}O_5$ [M+H]$^+$: 331.1545, found 331.1540; $[\alpha]_D^{25.0}$ +161.3° (*c* 0.900, $CHCl_3$).

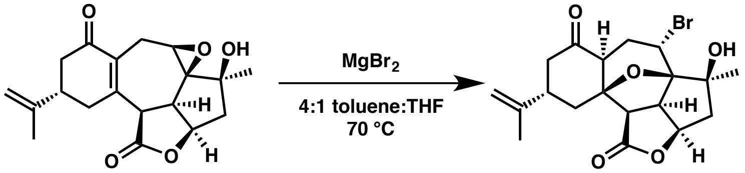


*ent-Isoineleganolide A*
*29*

*X-Ray*
*30*

**Bromide 30**: To a stirred colorless solution of *ent*-isoineleganolide A (**29**, 133 mg, 0.40 mmol, 1.00 equiv) in a mixture of toluene (27 mL) and THF (7 mL) in a nitrogen-filled glovebox was added $MgBr_2$ (370 mg, 2.01 mmol, 5.00 equiv) in a single portion. The reaction mixture was then sealed and heated to 70 °C. After 6 h, the consumption of starting material was complete as determined by TLC (1:4 EtOAc:$CH_2Cl_2$ eluent). The golden yellow solution was removed from the glovebox and concentrated in vacuo to approximately 25% of the original reaction volume. The reaction was then filtered through a silica gel plug, eluting the product with 20% EtOAc in $CH_2Cl_2$ to afford

spectroscopically pure bromide **30** (166 mg, >99% yield) as a white crystalline solid. Colorless, translucent X-ray quality crystals were obtained by slow diffusion of 1% benzene in heptane into a solution of bromide **30** in EtOAc, mp: 150–153 °C: $R_f$ = 0.26 (1:19 EtOAc:$CH_2Cl_2$ eluent); $^1$H NMR ($CDCl_3$, 600 MHz) δ 4.89 (dt, *J* = 9.1, 7.6 Hz, 1H), 4.84 (t, *J* = 1.5 Hz, 1H), 4.81 (s, 1H), 4.39 (dd, *J* = 11.3, 5.5 Hz, 1H), 3.74 (t, *J* = 9.2 Hz, 1H), 3.25 (d, *J* = 9.0 Hz, 1H), 3.08 (ddd, *J* = 14.1, 5.6, 1.4 Hz, 1H), 2.66 (tt, *J* = 13.2, 3.9 Hz, 1H), 2.56 (ddd, *J* = 13.2, 3.7, 2.0 Hz, 1H), 2.52 (d, *J* = 5.8 Hz, 1H), 2.36–2.27 (m, 2H), 2.22 (dd, *J* = 14.6, 12.9 Hz, 1H), 2.15 (m, 1H), 1.94 (m, 2H), 1.76 (d, *J* = 1.0 Hz, 3H), 1.49 (d, *J* = 1.2 Hz, 3H); $^{13}$C NMR ($CDCl_3$, 126 MHz) δ 205.7, 175.2, 145.7, 111.2, 96.3, 88.9, 80.8, 77.5, 55.5, 55.4, 48.4, 46.2, 45.4, 41.5, 41.1, 36.7, 32.3, 26.6, 20.2; IR (Neat Film, NaCl) 3508, 2970, 1767, 1716, 1443, 1354, 1271, 1203, 1173, 1073, 1016, 755 cm$^{-1}$; HRMS (FAB+) *m/z* calc'd for $C_{19}H_{24}O_5{}^{79}Br$ [M+H]$^+$: 411.0807, found 411.0800; $[\alpha]_D^{25.0}$ +26.7° (*c* 1.150, $CHCl_3$).

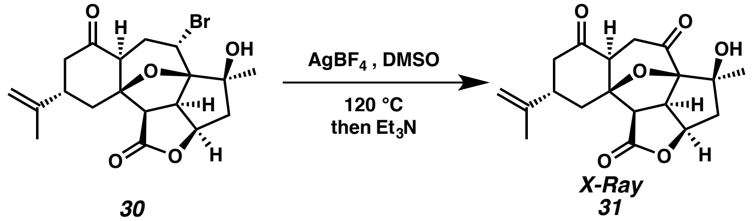


30

31

**Ketopyran 31**: A reaction vessel in a nitrogen-filled glove box was charged with $AgBF_4$ (43 mg, 0.22 mmol, 3.00 equiv) followed by bromide **30** (30 mg, 0.073 mmol, 1.00 equiv) as a solution in DMSO (1.5 mL) with stirring. The reaction vessel was sealed and after 5 minutes the white suspension had become a completely homogenous, pale yellow solution. The reaction vessel was removed from the glovebox, and introduced to an argon atmosphere and a preheated 120 °C bath. After 9 h, the consumption of starting material

was complete as determined by TLC (1:19 EtOAc:$CH_2Cl_2$ eluent). The dark brown, heterogeneous solution was removed from the heating bath and allowed to cool to ambient temperature (ca. 23 °C). Once the temperature had equilibrated, $Et_3N$ (0.30 mL, 2.15 mmol, 29.5 equiv) was added quickly dropwise with vigorous stirring. After 2 h, the reaction was filtered through a Celite plug, washing with EtOAc. The combined organics were diluted with EtOAc (30 mL) and washed with $H_2O$ (4 x 20 mL). The combined aqueous layers were then extracted with EtOAc (3 x 20 mL). The combined organics were dried over $MgSO_4$, filtered, and concentrated in vacuo. The crude brown solid was purified by silica gel column chromatography (40% EtOAc in $CH_2Cl_2$ eluent) to furnish ketopyran **31** (24 mg, 96% yield) as a crystalline white solid. Colorless, translucent X-ray quality crystals were obtained by slow diffusion of 1% benzene in heptane into a solution of ketopyran **31** in EtOAc, mp: 270–273 °C: $R_f$ = 0.40 (1:3 EtOAc:$CH_2Cl_2$ eluent); $^1$H NMR ($CDCl_3$, 500 MHz) δ 4.89–4.84 (m, 2H), 4.83 (dd, *J* = 2.3, 1.2 Hz, 1H), 3.56 (t, *J* = 9.0 Hz, 1H), 3.44 (d, *J* = 9.1 Hz, 1H), 3.20 (d, *J* = 15.6 Hz, 1H), 3.07 (dt, *J* = 7.4, 1.0 Hz, 1H), 2.76 (tt, *J* = 13.2, 3.6 Hz, 1H), 2.61 (ddd, *J* = 13.0, 3.6, 2.1 Hz, 1H), 2.43–2.28 (m, 5H), 2.13 (ddd, *J* = 14.7, 3.6, 2.1 Hz, 1H), 1.77 (t, *J* = 1.0 Hz, 3H), 1.50 (d, *J* = 1.1 Hz, 3H); $^{13}$C NMR ($CDCl_3$, 126 MHz) δ 204.9, 199.1, 174.6, 145.8, 111.4, 95.2, 90.8, 78.1, 77.5, 57.4, 54.4, 51.2, 46.1, 45.5, 41.0, 36.6, 34.9, 24.6, 20.4; IR (Neat Film, NaCl) 3484, 2965, 2923, 1766, 1732, 1204, 1172, 1071, 1032, 947, 754 cm$^{-1}$; HRMS (EI+) *m/z* calc'd for $C_{19}H_{22}O_6$ [M•]$^+$: 346.1416, found 346.1403; $[\alpha]_D^{25.0}$ –30.8° (*c* 0.800, $CHCl_3$).

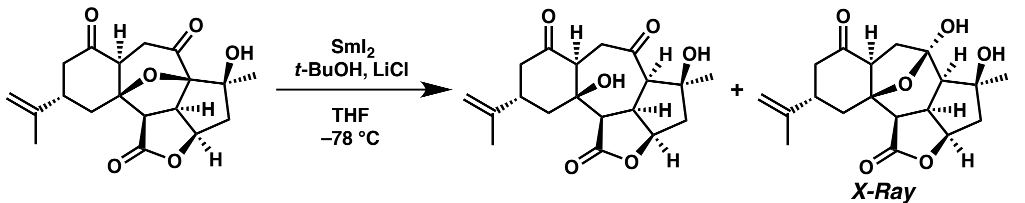


**Diol tetracycle 32**:

*Preparation of a 0.07 M Stock Solution $SmI_2$*

Into a Schlenk tube was added freshly filed samarium metal (150 mg, 1.00 mmol, 1.41 equiv). The reaction vessel was then thoroughly flame-dried, backfilled with argon, and allowed to cool to ambient temperature (ca. 23 °C). To the reaction vessel was then added THF (10.0 mL) that had previously been sparged with argon for 60 minutes and cooled to 0 °C (ice/$H_2O$ bath) with stirring. 1,2-Diiodoethane (200 mg, 0.71 mmol, 1.00 equiv) was then added in separate 100 mg portions 30 minutes apart. After the addition of the second portion, the Schlenk tube was removed from the cooling bath, allowed to warm to ambient temperature (ca. 23 °C), and the pale yellow solution was stirred overnight (ca. 14 h) causing the reaction to become deep blue, indicating the formation of $SmI_2$.

*Reduction of Ketopyran **31***

A reaction vessel in a nitrogen-filled glovebox was charged with LiCl (49 mg, 1.15 mmol, 19.8 equiv), sealed, removed from the glovebox, and introduced to an argon atmosphere. To the reaction vessel was added a solution of ketopyran **31** (20 mg, 0.058 mmol, 1.00 equiv) in THF (26 mL) followed by *t*-BuOH (15 μL, 0.16 mmol, 1.23 equiv). The white suspension was then sparged with argon for 1 h, reducing the reaction volume to 20 mL. The reaction solution was then cooled to –78 °C (*i*-PrOH/dry ice bath) at which time $SmI_2$ (2.00 mL, 0.07 M in THF, 1.08 equiv) was added slowly dropwise over 5

minutes, dropping the $SmI_2$ solution down the sides of the reaction flask. After 15 minutes, the consumption of starting material was complete as determined by TLC (1:3 EtOAc:$CH_2Cl_2$ eluent). The reaction was quenched by the addition of saturated aqueous $NH_4Cl$ (100 μL), immediately removed from the cooling bath, and allowed to warm to ambient temperature (23 °C). The yellow reaction mixture was filtered through a silica gel plug, eluting the product with 100% EtOAc. The organics were concentrated in vacuo and the crude pale yellow solid was purified by silica gel column chromatography (75%→85% EtOAc in $CH_2Cl_2$ eluent) to provide diol **32** (17 mg, 85% yield) as a crystalline white solid. Diol **32** was characterized as an inseparable, equilibrium mixture of free diol **32** and hemiketal **S2**. Colorless, translucent X-ray quality crystals of hemiketal **S2** were obtained by slow diffusion of 1% benzene in heptane into a solution of diol **32** and **S2** in EtOAc, mp: 225–228 °C: $R_f$ = 0.32 (3:1 EtOAc:$CH_2Cl_2$ eluent); $^1$H NMR ($CDCl_3$, 500 MHz) δ 4.92 (dd, *J* = 7.0, 5.1 Hz, 1.00 H), 4.84 (dt, *J* = 2.8, 1.4 Hz, 2.28 H), 4.82–4.76 (m, 3.44 H), 3.63 (s, 0.95 H), 3.38 (tdd, *J* = 10.4, 7.0, 0.7 Hz, 0.99 H), 3.30 (d, J = 13.6 Hz, 1.23 H), 3.24 (d, *J* = 8.2 Hz, 1.23 H), 3.22–3.09 (m, 3.39 H), 3.02–2.95 (m, 2.16 H), 2.86–2.80 (m, 2.43 H), 2.76–2.55 (m, 8.89 H), 2.46 (dd, *J* = 14.0, 10.1 Hz, 1.00 H), 2.39–2.22 (m, 6.90 H), 2.19 (d, *J* = 10.3 Hz, 0.98 H), 2.15–2.09 (m, 1.16 H), 1.90 (dd, *J* = 15.3, 12.4 Hz, 1.03 H), 1.82–1.75 (m, 6.90 H), 1.72 (dd, *J* = 14.3, 12.1 Hz, 1.13 H), 1.53 (s, 2.90 H), 1.35 (d, *J* = 1.0 Hz, 3.61 H); $^{13}$C NMR ($CDCl_3$, 126 MHz) δ 209.9, 209.1, 206.1, 174.8, 173.2, 146.5, 146.2, 111.0, 110.9, 105.6, 83.9, 81.2, 80.5, 79.3, 78.9, 75.0, 58.0, 54.4, 54.3, 52.2, 51.9, 47.6, 47.4, 46.4, 46.0, 45.6, 44.5, 42.7, 41.7, 41.1, 40.3, 38.2, 37.5, 36.1, 28.8, 24.8, 20.8, 20.7; IR (Neat Film, NaCl) 3358, 2921, 1752, 1711, 1689, 1358, 1261, 1182, 1098, 1026, 936, 896, 799, 756 cm$^{-1}$; HRMS (MM:

ESI-APCI) *m/z* calc'd for $C_{19}H_{23}O_6$ [M–H]$^-$: 347.1500, found 347.1509; $[\alpha]_D^{25.0}$ +3.1° (*c* 0.250, $CHCl_3$).

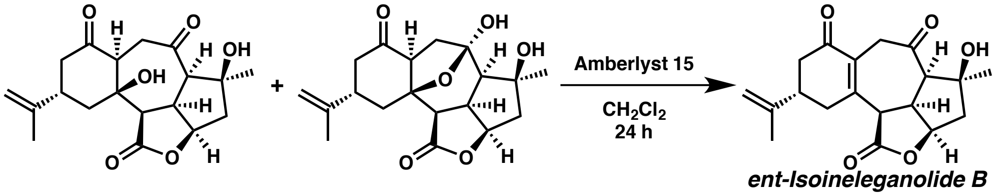


***ent*-Isoineleganolide B (*ent*-12)**: To a heterogeneous reaction mixture of diol **32** and hemiketal **S2** (30 mg, 0.086 mmol, 1.00 equiv) in $CH_2Cl_2$ (6.0 mL) was added Amberlyst® 15 (75 mg, 2.5 equiv by wt. to diol mixture **32** and **S2**) as a solid in one portion. After 24 h, the consumption of starting material was complete as determined by TLC (3:1 EtOAc:$CH_2Cl_2$ eluent). The heterogeneous, light yellow reaction mixture was filtered and the organics were concentrated in vacuo. The crude yellow solid was purified by silica gel column chromatography (30% EtOAc in $CH_2Cl_2$ eluent) to provide *ent*-isoineleganolide B (***ent*-12**, 19 mg, 63% yield) as an amorphous yellow solid: $R_f$ = 0.27 (2:3 EtOAc:$CH_2Cl_2$ eluent); $^1$H NMR ($CDCl_3$, 600 MHz) δ 4.86 (dd, *J* = 2.3, 1.2 Hz, 1H), 4.80 (ddd, *J* = 7.0, 5.7, 2.6 Hz, 1H), 4.71–4.68 (m, 1H), 4.21 (d, *J* = 15.3 Hz, 1H), 3.91 (ddt, *J* = 7.4, 2.6, 1.3 Hz, 1H), 3.71 (dtd, *J* = 17.9, 2.8, 1.3 Hz, 1H), 3.31 (ddd, *J* = 13.2, 7.4, 5.8 Hz, 1H), 3.08 (dq, *J* = 15.3, 2.8 Hz, 1H), 2.91–2.83 (m, 1H), 2.78 (dp, *J* = 9.1, 4.5 Hz, 1H), 2.71 (ddd, *J* = 16.5, 4.5, 1.2 Hz, 1H), 2.53 (m, 1H), 2.48–2.41 (m, 1H), 2.41–2.35 (m, 1H), 2.26–2.22 (m, 1H), 1.79–1.78 (m, 3H), 1.30 (s, 3H); $^{13}$C NMR ($CDCl_3$, 126 MHz) δ 207.9, 196.5, 172.1, 149.7, 145.6, 128.2, 111.3, 79.5, 78.2, 59.8, 51.0, 46.2, 43.0, 42.4, 39.7, 39.5, 33.8, 25.2, 21.3; IR (Neat Film, NaCl) 3458, 2960,

2923, 2854, 1767, 1709, 1662, 1438, 1377, 1262, 1139, 1038 $cm^{-1}$; HRMS (FAB+) *m/z* calc'd for $C_{19}H_{23}O_5$ $[M+H]^+$: 331.1545, found 331.1548; $[\alpha]_D^{25.0}$ –66.3° (*c* 0.275, $CHCl_3$).

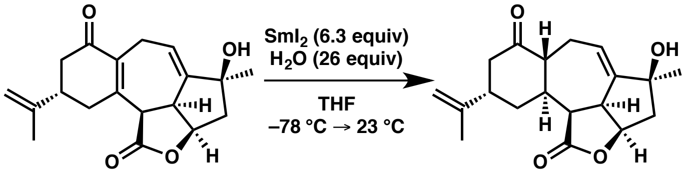


**28** **35**

**Allylic alcohol 35**:

*Preparation of* $SmI_2$

Into each of two Schlenk tubes was added freshly filed samarium metal (150 mg, 1.00 mmol, 6.25 equiv). The reaction vessel was then thoroughly flame-dried, backfilled with argon, and allowed to cool to ambient temperature (ca. 23 °C). To each reaction vessel was added THF (10.0 mL) that had previously been sparged with argon for 60 minutes and cooled to 0 °C (ice/$H_2O$ bath) with stirring. Diiodoethane (200 mg, 0.71 mmol, 4.44 equiv) was then added to each Schlenk tube in separate 100 mg portions 30 minutes apart. After the addition of the second portion, the Schlenk tubes were removed from the cooling bath, allowed to warm to ambient temperature (ca. 23 °C), and the pale yellow solution was stirred overnight (ca. 14 h) causing the reaction to become deep blue, indicating formation of $SmI_2$.

*Reduction of Diene* ***28***

Each Schlenk tube was cooled to –78 °C (*i*-PrOH/dry ice bath) followed by the addition of $H_2O$ (75 $\mu$L, 4.16 mmol, 26.0 equiv). After stirring for 5 minutes, the addition of diene **28** (50 mg, 0.16 mmol, 1.00 equiv) as solution in thoroughly sparged THF (1.60 mL) was accomplished quickly dropwise over 4 minutes. After 2 h, the reaction vessel

was warmed to 0 °C (ice/$H_2O$ bath). After an additional 2 h, the Schlenk tube was removed from the cooling bath and allowed to warm. After 20 minutes, before warming all the way to ambient temperature (ca. 23 °C), the consumption of starting material was complete as determined by TLC (1:4 EtOAc:$CH_2Cl_2$ eluent). The dark blue reaction mixture was quenched by the addition of hexanes (10.0 mL) and $H_2O$ (0.10 mL). After stirring for 5 minutes, both reaction mixtures were combined, filtered through a pad of silica gel (50% acetone in hexanes eluent), and concentrated in vacuo. The crude tan solid was purified by silica gel column chromatography (20% acetone in hexanes eluent) to afford allylic alcohol **35** (56 mg, 56% yield) as an amorphous white solid: $R_f$ = 0.21 (1:4 Acetone:Hexanes eluent); $^1$H NMR ($CDCl_3$, 500 MHz) δ 6.32–6.24 (m, 1H), 4.93–4.86 (m, 1H), 4.74–4.70 (m, 1H), 4.63 (dt, *J* = 1.8, 0.9 Hz, 1H), 3.37 (dq, *J* = 6.0, 2.9 Hz, 1H), 3.12 (ddd, *J* = 15.9, 9.7, 1.7 Hz, 1H), 2.99–2.89 (m, 2H), 2.84 (dq, *J* = 7.8, 4.4 Hz, 1H), 2.68 (ddd, *J* = 15.1, 3.2, 2.3 Hz, 1H), 2.62 (ddd, *J* = 15.2, 6.0, 0.9 Hz, 1H), 2.40–2.29 (m, 2H), 2.19 (tt, *J* = 11.4, 3.8 Hz, 1H), 2.08 (dd, *J* = 15.7, 4.3 Hz, 1H), 2.02 (s, 1H), 1.83 (dtd, *J* = 14.1, 3.8, 2.0 Hz, 1H), 1.74 (dt, *J* = 1.5, 0.7 Hz, 3H), 1.73–1.65 (m, 1H), 1.35 (s, 3H); $^{13}$C NMR ($CDCl_3$, 126 MHz) δ 210.3, 174.8, 149.9, 146.2, 128.2, 113.0, 83.1, 79.4, 49.7, 49.4, 48.4, 45.8, 44.5, 40.0, 39.8, 32.4, 30.0, 26.9, 22.5; IR (Neat Film, NaCl) 3479, 2965, 1760, 1699, 1444, 1372, 1224, 1138, 992, 900, 754 cm$^{-1}$; HRMS (FAB+) *m/z* calc'd for $C_{19}H_{25}O_4$ [M+H]$^+$: 315.1596, found 315.1600; $[\alpha]_D^{25.0}$ –17.7° (*c* 0.400, $CHCl_3$).

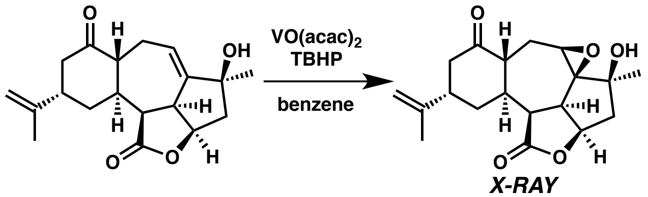


35 36

**Epoxytetracycle 36**: To a pale yellow stirred solution of allylic alcohol **35** (50 mg, 0.16 mmol, 1.00 equiv) in a vial open to air in benzene (5.3 mL) was added $VO(acac)_2$ (0.5 mg, 0.0016 mmol, 0.01 equiv). After 5 minutes, to this dark green solution was added *t*-butyl hydroperoxide (TBHP, 36 μL, 0.018 mmol, 1.10 equiv) as a 5 M solution in decane dropwise causing the reaction to immediately became deep ruby red. After 1 h, the reaction had lost all red color and become pale yellow and the consumption of starting material was complete as determined by TLC (1:4 Acetone:Hexanes eluent). The reaction was concentrated in vacuo and the crude tan solid was purified by silica gel column chromatography (25% acetone in hexanes eluent) to afford epoxide **36** (47 mg, 94% yield) as a white crystalline solid. Colorless, translucent X-ray quality crystals were obtained by slow diffusion of 1% benzene in heptane into a solution of epoxide **36** in EtOAc, mp: 183–185 °C: $R_f$ = 0.15 (1:4 Acetone:Hexanes eluent); $^1$H NMR ($CDCl_3$, 600 MHz) δ 4.87 (s, 1H), 4.79–4.73 (m, 1H), 4.63–4.57 (m, 1H), 3.34 (d, *J* = 7.1 Hz, 1H), 3.24 (ddd, *J* = 15.9, 7.2, 1.8 Hz, 1H), 3.05 (dd, *J* = 6.0, 4.4 Hz, 1H), 2.97 (ddd, *J* = 13.5, 11.8, 4.5 Hz, 1H), 2.84 (dd, *J* = 6.5, 3.4 Hz, 1H), 2.76 (dd, *J* = 6.0, 3.5 Hz, 1H), 2.67 (m, 2H), 2.63 (dd, *J* = 15.3, 6.0 Hz, 1H), 2.48 (bs, 1H), 2.38 (dd, *J* = 15.8, 6.7 Hz, 1H), 2.18 (d, *J* = 15.8 Hz, 1H), 1.89–1.74 (m, 2H), 1.74 (s, 3H), 1.53 (dd, *J* = 15.8, 10.8 Hz, 1H), 1.31 (s, 3H); $^{13}$C NMR ($CDCl_3$, 126 MHz) δ 210.3, 173.8, 146.2, 113.0, 79.2, 75.3, 71.4, 54.4, 46.8, 46.2, 45.3, 44.3, 44.2, 39.7, 38.0, 31.8, 26.3, 25.6, 22.4; IR (Neat Film, NaCl) 3518, 2963, 2931, 1766, 1703, 1442, 1370, 1259, 1131, 985, 893, 758 cm$^{-1}$; HRMS

(FAB+) *m/z* calc'd for $C_{19}H_{25}O_5$ $[M+H]^+$: 333.1702, found 333.1716; $[\alpha]_D^{25.0}$ +13.2° (*c* 0.200, $CHCl_3$).

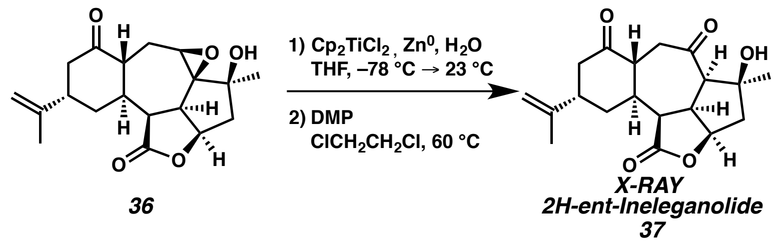


**2*H*-*ent*-Ineleganolide (37)**:

*Preparation of a 0.50 M Stock Solution of Titanocene Monchloride ($Cp_2TiCl$)*

Into a thoroughly flame-dried Schlenk tube under an overpressure of argon was charged with zinc(0) dust (647 mg, 9.90 mmol, 3.00 equiv) and titanocene dichloride ($Cp_2TiCl_2$, 822 mg, 3.30 mmol, 1.00 equiv). The flask was then evacuated and back filled with argon (3 x 5 minute cycles). To the reaction vessel was then added THF (6.6 mL) that had previously been sparged with argon for 60 minutes and stirring commenced. After 1.5 h, the bright red reaction mixture had become dark green and stirring was halted. After 30 minutes, the supernatant was used as a 0.50 M stock solution of $Cp_2TiCl$.

*Reductive Opening of Epoxide **36***

A stirred solution of epoxide **36** (26 mg, 0.078 mmol, 1.00 equiv) in THF (2.5 mL) was sparged with argon for 1 h, resulting in a reaction volume of 1.5 mL. The homogeneous, off-white reaction mixture was then cooled to –78 °C (*i*-PrOH/dry ice bath) followed by the addition of $H_2O$ (108 $\mu$L, 6.00 mmol, 76.9 equiv). After stirring for 5 minutes, $Cp_2TiCl$ (1.50 mmol, 0.50 M in THF, 19.2 equiv) was added dropwise over 8 minutes. After 2 h, the reaction vessel was warmed to 0 °C (ice/$H_2O$ bath). After an additional 1.5 h, the Schlenk tube was removed from the cooling bath and allowed to

warm to ambient temperature (ca. 23 °C). After an additional 18.5 h, the consumption of starting material was complete as determined by TLC (3:7 Acetone:Hexanes eluent). The reaction was quenched by the addition of saturated $NaH_2PO_4$ (1.0 mL) and brine (1.0 mL), sparged with compressed air for 5 minutes, and allowed to stir for an additional 15 minutes. The reaction mixture was then filtered through a Celite® plug, washing with 50% acetone in hexanes eluent. The combined organics were concentrated in vacuo and immediately purified by silica gel column chromatography (50% EtOAc in $CH_2Cl_2$ eluent), furnishing a mixture of diol products (21 mg, 81% yield) that was directly carried on without further purification.

*Oxidation of Intermediate Diol Products*

To a portion of the diol products (8 mg, 0.024 mmol, 1.00 equiv) in wet DCE[7] (3.0 mL) was added DMP (60 mg, 0.14 mmol, 5.82 equiv) at ambient temperature (ca. 23 °C) with stirring. The reaction vessel was then sealed and heated to 65 °C. After 18 h, the consumption of starting material was complete as determined by TLC (1:1 EtOAc:$CH_2Cl_2$ eluent). The reaction vessel was removed from the heating bath and allowed to cool to ambient temperature (ca. 23 °C). The reaction mixture was then quenched by the addition of saturated $NaS_2O_3$ (3.0 mL) and saturated $NaHCO_3$ (3.0 mL). After stirring to 10 minutes, the reaction mixture was diluted with $H_2O$ (10 mL) and extracted with $CH_2Cl_2$ (3 x 10 mL). The combined organics were then dried over $MgSO_4$, filtered, and concentrated in vacuo. The crude brown solid was purified by silica gel column chromatography (25% acetone in hexanes eluent) to furnish 2*H*-*ent*-ineleganolide (**37**, 4 mg, 50% yield) as a crystalline white solid. Colorless, translucent X-ray quality crystals were obtained by slow diffusion of 1% benzene in heptane into a solution of 2*H*-

*ent*-ineleganolide (**37**) in EtOAc, mp: 218–220 °C: $R_f$ = 0.23 (3:7 Acetone:Hexanes eluent); $^1$H NMR ($CDCl_3$, 500 MHz) δ 4.96–4.92 (m, 1H), 4.80 (ddd, *J* = 7.2, 6.2, 3.8 Hz, 1H), 4.68 (dt, *J* = 1.7, 0.8 Hz, 1H), 3.62 (q, *J* = 1.3 Hz, 1H), 3.21 (td, *J* = 9.3, 6.2 Hz, 1H), 3.08 (d, *J* = 9.6 Hz, 1H), 3.03 (dd, *J* = 9.0, 1.7 Hz, 1H), 2.96 (dd, *J* = 11.6, 3.8 Hz, 1H), 2.89–2.78 (m, 3H), 2.73 (dt, *J* = 14.7, 2.3 Hz, 1H), 2.67 (ddd, *J* = 14.7, 6.1, 1.0 Hz, 1H), 2.51–2.37 (m, 3H), 2.24–2.15 (m, 1H), 1.91 (ddd, *J* = 10.9, 6.0, 2.6 Hz, 1H), 1.78–1.74 (m, 3H), 1.28 (t, *J* = 1.0 Hz, 3H).; $^{13}$C NMR ($CDCl_3$, 126 MHz) δ 213.8, 208.2, 174.1, 145.7, 113.7, 80.9, 80.4, 61.8, 48.0, 46.4, 46.3, 46.1, 44.0, 43.2, 40.3, 37.0, 34.0, 28.7, 22.6; IR (Neat Film, NaCl) 3501, 2965, 2925, 1761, 1698, 1440, 1368, 1318, 1262, 1160, 1081, 1030, 1003, 800, 758 $cm^{-1}$; HRMS (FAB+) *m/z* calc'd for $C_{19}H_{25}O_5$ $[M+H]^+$: 333.1702, found 333.1714; $[\alpha]_D^{25.0}$ –32.6° (*c* 0.150, $CHCl_3$).

**Biological Assay[8]**

***PBMC IL-17 Secretion ELISA and Cell TiterGlo Viabiltiy Assay***

*PBMC Isolation*

PBMC's (peripheral blood mononuclear cells) are isolated from whole blood buffy coats by first combining fresh buffy coats (obtained from the Indiana blood center) with equal volumes of phosphate buffered saline (PBS). Thirty five mL of PBS/buffy coat solution are then gently overlaid onto 15 mL of ficol in 50 mL conical tubes. Following centrifugation for 30 minutes at 500xG (with slow acceleration and deceleration) the top layer of plasma is discarded and the layer of cells along the ficol interface is collected and pooled (five donors per 250 mL conical tube). Each 250 mL tube is filled to the top with room temperature RPMI-1640 media. Tubes are spun for 10 minutes at 500xG (with slow acceleration and deceleration), media is removed by aspiration and the wash step is repeated. Cells are resuspended in ice cold Recovery Cell Culture Freezing Medium from Life Technologies (Catalog number 12648-010) on ice. The cell concentration is adjusted to 66.7 million cells/mL. Cells are slow frozen at –1° C/minute in vials with 100 million cells and stored in liquid nitrogen.

*Stimulation of IL-17 Secretion and Compound Addition*

PBMC's from 20–30 combined donors are brought out of thaw by re-suspending with 1mL of complete media (RPMI-1640 containing 30 mM HEPES, 100 units/mL penicillin, 100 μg/mL streptomycin, 3.25 mM L-Glutamine, 0.2 μM beta-mercaptoethanol, and 10% fetal bovine serum) followed by the dropwise addition of 2mL, 4mL, 8mL, and finally 16mL of complete media with gentle swirling. Cells are spun down for 5 minutes and the cell pellet is resuspended in complete media. Clumps of

cells are broken up by running the cell solution through a 23 gauge syringe needle and a 40 μM cell strainer. One hundred thousand cells per well are added to 384 well polystyrene tissue culture treated flat-bottomed plates in a total of 30uL. Stimulation cocktail containing anti-human CD3 antibody, anti-human CD28 antibody, and IL23 (all produced in-house) and compounds prepared in complete media are added to the cells simultaneously in a total volume of 30 uL. The final concentration of added stimulants is 160ng/mL, 500ng/mL, and 5ng/mL for anti-CD3 antibody, anti-CD28 antibody , and IL-23 respectively and 0.3% for DMSO. The final concentration of the compound of interest was 10 μM. Plates are sealed with AeraSeal® sealing film and incubated for 48 hours at 37° C, 95% humidity, and 5% $CO_2$.

*IL-17 ELISA and Analysis*

Following the incubation period the plates are spun at 200xG for five minutes. Supernatants are diluted one to two in 1%BSA/PBS and tested for IL-17 with a human IL-17 ELISA kit from R&D system (catalog #D317E) according to the protocol provided with the kit. Absorbance at 492 nm is measured with the Envision multi-label plate reader. A492 values are converted to concentration of IL-17 based on the IL-17 standard curve as shown:

$$\text{pg/mL IL-17} = \text{EC50}*[[(\text{Top-Bottom})/(\text{A492-Bottom})]-1]^{(1/-\text{Hill})}$$

*Cell Viability Testing and Analysis*

Equal volumes of Cell TiterGlo® cell viability testing reagent (Promega Cat# G7573) are added to the cells remaining in the plates, and following a fifteen minute incubation with gentle shaking at room temperature luminescence is measures with the Envision multi-label plate reader. Percent cell death is calculated by setting 100% activity

(cell death) to zero luminescence units and minimum activity (max number of viable cells) as the average luminescence units of wells containing stimulants alone and no added compound.

*IL-5 ELISA and Analysis*

Following the incubation period the plates are spun at 200xG for five minutes. Undiluted supernatants are tested for IL-5 with a human IL-5 ELISA kit from R&D system (catalog #DY205) according to the protocol provided with the kit. Absorbance is measured with the Envision multi-label plate reader. Absorbance values are converted to concentration of IL-5 based on the IL-5 standard curve as shown:

pg/mL IL-5 = EC50*[[(Top-Bottom)/(Abs-Bottom)]-1](1/-Hill)

*Table S1. IL-5 and IL-17 Secretory Inhibition of Ineleganoloids*

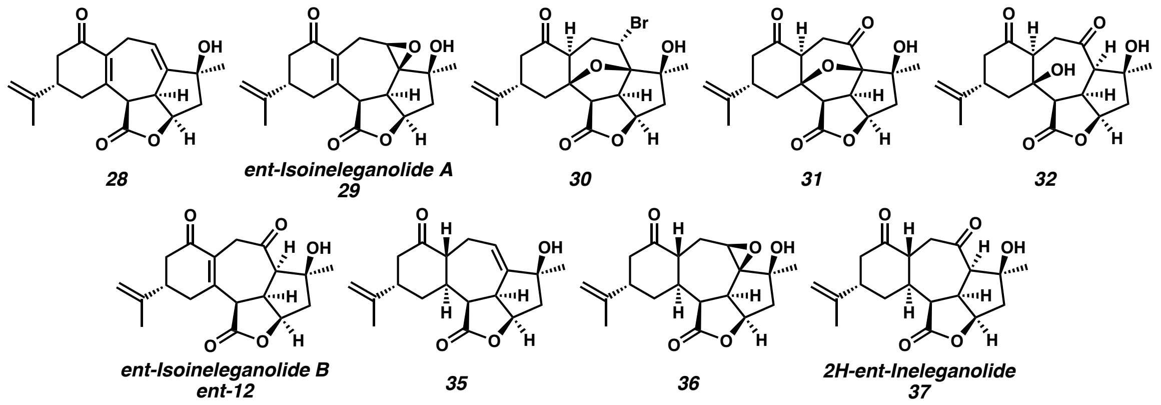


| Entry | Compound | IL-5 Secretion Inhibition (%) | IL-17 Secretion Inhibition (%) | Cytotoxicity (%) |
|---|---|---|---|---|
| 1 | ***28*** | 7.8 | 38.1 | 3.9 |
| 2 | ***29*** | 23.3 | 24.5 | 4.9 |
| 3 | ***30*** | 47.2 | 3.8 | 4.9 |
| 4 | ***31*** | 40.2 | < 2 | 6.7 |
| 5 | ***32*** | < 2 | 23.9 | < 2 |
| 6 | ***33*** | 26.8 | 15.3 | 8.8 |
| 7 | ***35*** | 15.3 | 7.0 | 3.4 |
| 8 | ***36*** | 40.2 | < 2 | 5.9 |
| 9 | ***37*** | 6.1 | < 2 | < 2 |

**Computational Assessment of Isomerization of Enone *ent*-12**

As noted in footnote 23 of the manuscript, all calculations were performed with Spartan '10 (Wavefunction, Inc., Irvine, CA). The in vacuo equilibrium geometry for each structure was calculated by a series of sequential calculations as follows: Hartree–Fock computation (equilibrium geometry, 3-21G basis set), DFT (equilibrium geometry, B3LYP/6-31G basis set), DFT (energy, B3LYP/6-311+G** basis set), DFT (equilibrium geometry, B3LYP/6-311+G** basis set). The error from these calculations is ±0.23 kcal/mol, thus all energy differences larger than 0.46 kcal/mol were considered significant. Except for molecular mechanics and semi-empirical models, the calculation methods used in Spartan have been documented in the literature.[9] All ground state energies have been expressed relative to the energy of *ent*-ineleganolide (***ent*-1**) in the naturally occurring conformation (cf. ***ent*-1**$^{ax}$, Scheme 4, Table S2) as determined on the initial isolation by single crystal X-ray diffraction.[10]

*Table S2. Calculated Ground State Energies for Enone **ent-12** and Isomers*

| *entry* | *compound* | | *relative ground state free energy (kcal/mol)** |
|---|---|---|---|
| *1* | 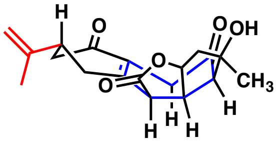 | *ent-12*$^{\mathrm{equ}}$ | *0.8* |
| *2* | 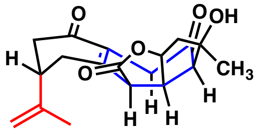 | *ent-12*$^{\mathrm{ax}}$ | *2.1* |
| *3* | 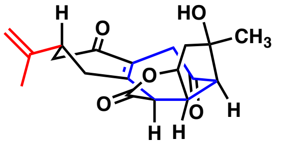 | *ent-12A*$^{\mathrm{equ}}$ | *–1.4* |
| *4* | 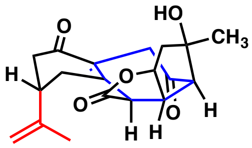 | *ent-12A*$^{\mathrm{ax}}$ | *0.7* |
| *5* | 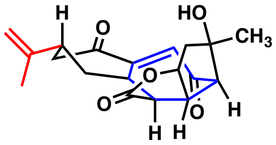 | *34*$^{\mathrm{equ}}$ | *14.1* |
| *6* | 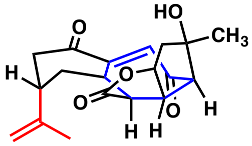 | *34*$^{\mathrm{ax}}$ | *14.2* |
| *7* | 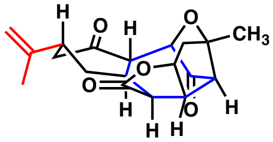 | *ent-1*$^{\mathrm{equ}}$ | *1.9* |
| *8* | 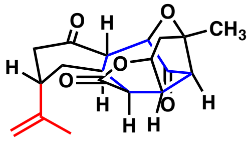 | *ent-1*$^{\mathrm{ax}}$ | *0.0* |

*: Relative ground state free energies are calculated relative to the energy of ***ent-1***$^{\mathrm{ax}}$ (entry 8) where posititve and negative values denote higher and lower relative ground state energies, respectively.

**Computational Assessment of Conformations of 2*H*-*ent*-Ineleganolide (37)**

As above, all energy minimizations were performed with Spartan '10 (Wavefunction, Inc., Irvine, CA)[9] by the following series: Hartree–Fock computation (equilibrium geometry, 3-21G basis set), DFT (equilibrium geometry, B3LYP/6-31G basis set), DFT (energy, B3LYP/6-311+G** basis set), DFT (equilibrium geometry, B3LYP/6-311+G** basis set). The error from these calculations is ±0.23 kcal/mol, thus all energy differences larger than 0.46 kcal/mol were considered significant. All ground state energies have been expressed relative to the energy of 2*H*-*ent*-ineleganolide (**37**) in the observed conformation (cf. **37A$^{ax}$**, Scheme 7, Table S3, pp. S147–170, vide infra) as determined by single crystal X-ray diffraction.

*Table S3. Calculated Ground State Energies for 2H-ent-Ineleganolide (**37**)*

| *entry* | *compound* | | *relative ground state free energy (kcal/mol)** |
|---|---|---|---|
| ***1*** | 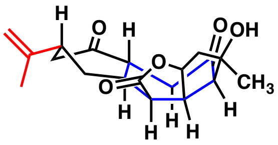 | ***37A$^{equ}$*** | ***1.0*** |
| ***2*** | 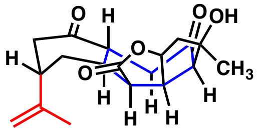 | ***37A$^{ax}$*** | ***0.0*** |
| ***3*** | 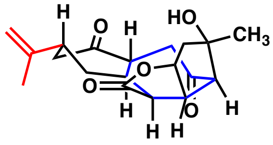 | ***37B$^{equ}$*** | ***0.6*** |
| ***4*** | 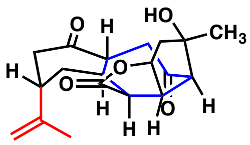 | ***37B$^{ax}$*** | ***–0.1*** |

*: Relative ground state free energies are calculated relative to the energy of **37A$^{ax}$** (entry 2) where posititve and negative values denote higher and lower relative ground state energies, respectively.

**Notes & References**

1. A. M. Pangborn, M. A. Giardello, R. H. Grubbs, R. K. Rosen and F. J Timmers, *Organometallics* 1996, **15**, 1518.

2. R. A. Craig, II, J. L. Roizen, R. C. Smith, A. C. Jones and B. M. Stoltz, *Org. Lett.* 2012, **14**, 5716.

3. M. Shibuya, M. Tomizawa and Y. Iwabuchi, *J. Org. Chem.* 2008, **73**, 4750.

4. (a) M. A. González, S. Ghosh, F. Rivas, D. Fischer and E. A. Theodorakis, *Tetrahedron Lett.* 2004, **45**, 5039; (b) J. Chen and J. N. Marx, *Tetrahedron Lett.* 1997, **38**, 1889; (c) J.-F. Lavallée, C. Spino, R. Ruel, K. T. Hogan and P. Deslongchamps, *Can. J. Chem.* 1991, **70**, 1406.

5. H. M. L. Davies, W. R. Cantrell, Jr., K. R. Romines and J. S. Baum, *Org. Synth.* 1992, **70**, 93.

6. Although we began with anhydrous $CeCl_3$, the drying procedure greatly increased the yield. It is likely increased surface area of the $CeCl_3$ after grinding due to stirring during the drying procedure that facilitated the observed increase in yield.

7. Solvent was not distilled or dried prior to use.

8. Biological assays were developed by and implemented in collaboration with the Open Innovation Drug Discovery program at Eli Lilly & Co. For full details on the program, please visit the program website at https://openinnovation.lilly.com (last accessed on 12-09-2015).

9. Y. Shao, L. F. Molnar, Y. Jung, J. Kussmann, C. Ochsenfeld, S. T. Brown, A. T. B. Gilbert, L. V. Slipchenko, S. V. Levchenko, D. P. O'Neill, R. A. DiStasio, Jr., R. C. Lochan, T. Wang, G. J. O. Beran, N. A. Besley, J. M. Herbert, C. Y. Lin, T.

Van Voorhis, S. H. Chien, A. Sodt, R. P. Steele, V. A. Rassolov, P. E. Maslen, P. P. Korambath, R. D. Adamson, B. Austin, J. Baker, E. F. C. Byrd, H. Dachsel, R. J. Doerksen, A. Dreuw, B. D. Dunietz, A. D. Dutoi, T. R. Furlani, S. R. Gwaltney, A. Heyden, S. Hirata, C.-P. Hsu, G. Kedziora, R. Z. Khalliulin, P. Klunzinger, A. M. Lee, M. S. Lee, W. Z. Liang, I. Lotan, N. Nair, B. Peters, E. I. Proynov, P. A. Pieniazek, Y. M. Rhee, J. Ritchie, E. Rosta, C. D. Sherrill, A. C. Simmonett, J. E. Subotnik, H. L. Woodcock, III, W. Zhang, A. T. Bell, A. K. Chakraborty, D. M. Chipman, F. J. Keil, A. Warshel, W. J. Hehre, H. F. Schaefer, III, J. Kong, A. I. Krylov, P. M. W. Gill and M. Head-Gordon *Phys. Chem. Chem. Phys.* 2006, **8**, 3172.

10. C.-Y. Duh, S.-K. Wang, M.-C. Chia and M. Y. Chiang, *Tetrahedron Lett.* 1999, **40**, 6033.

**NMR & IR Spectra**

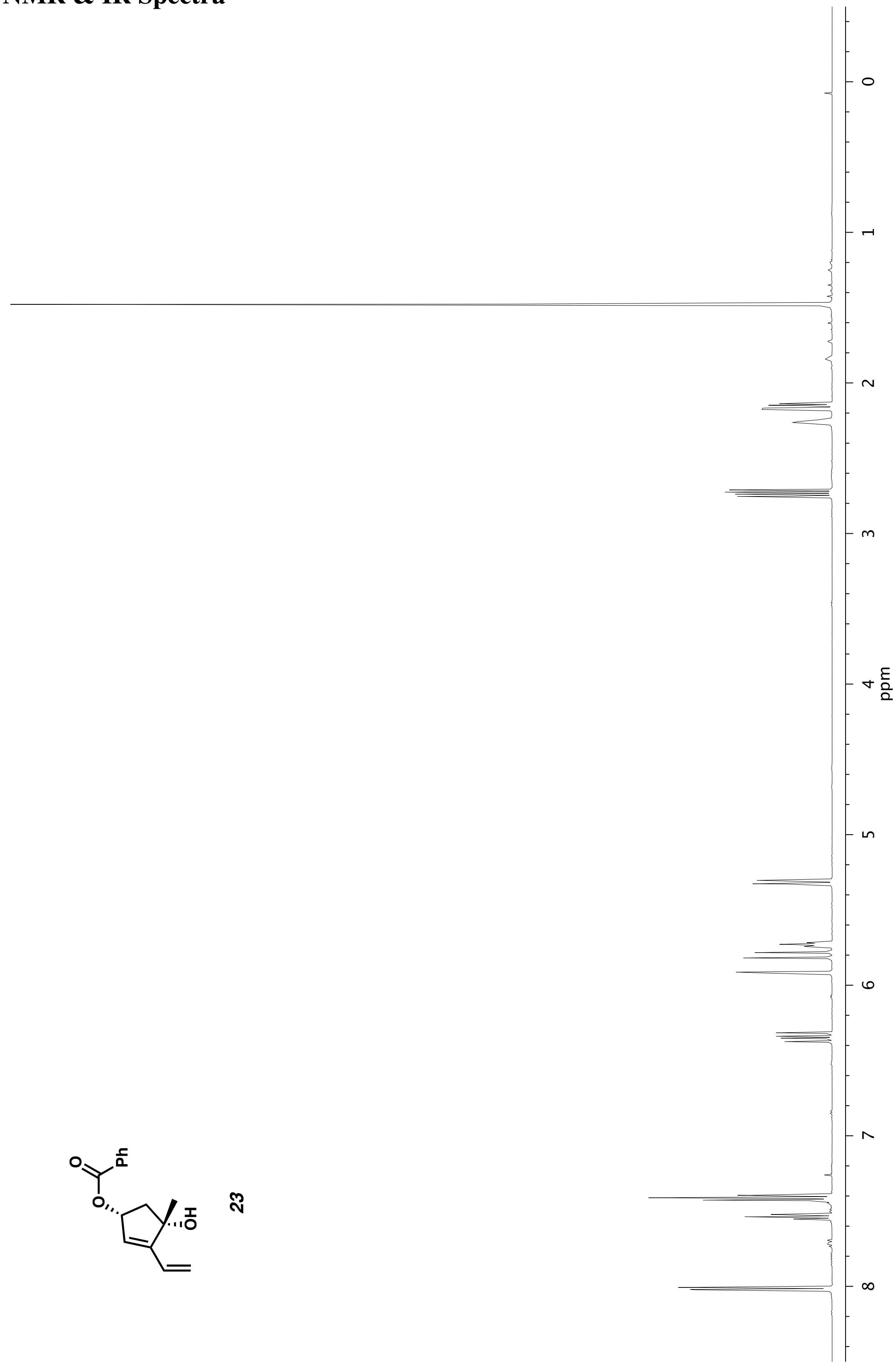


$^{1}$H NMR (500 MHz, $CDCl_3$) of compound **23**.

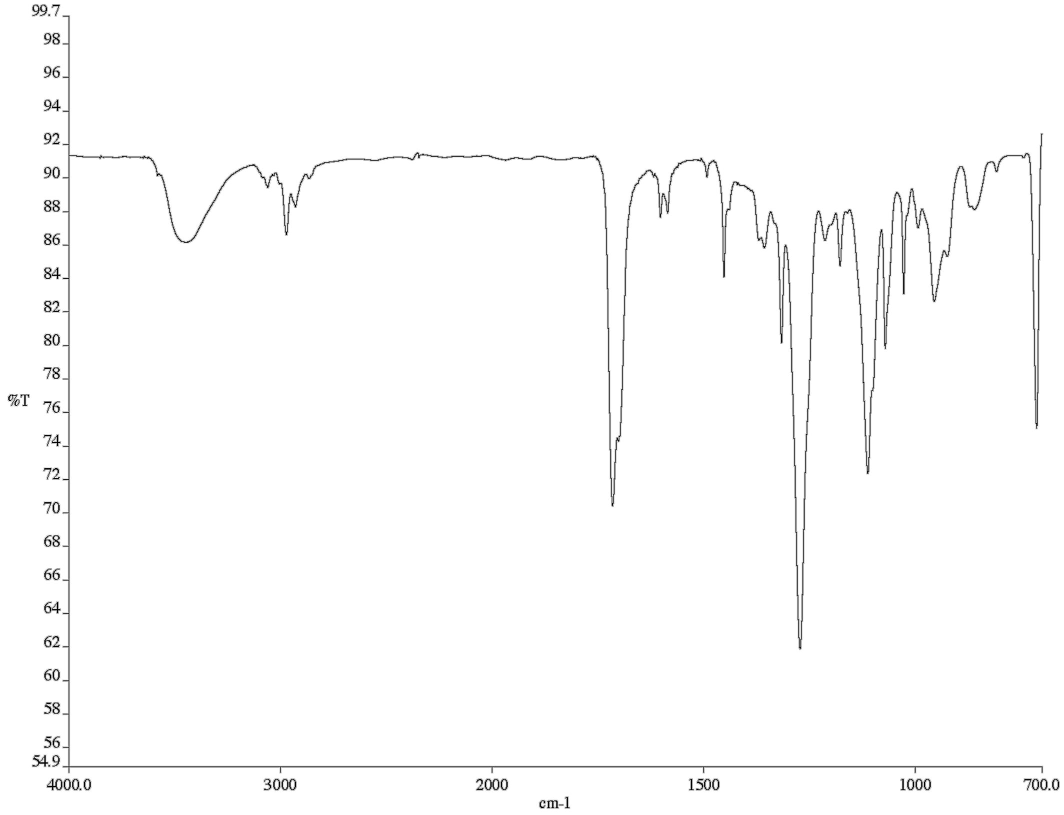


Infrared spectrum (Thin Film, NaCl) of compound **23**.

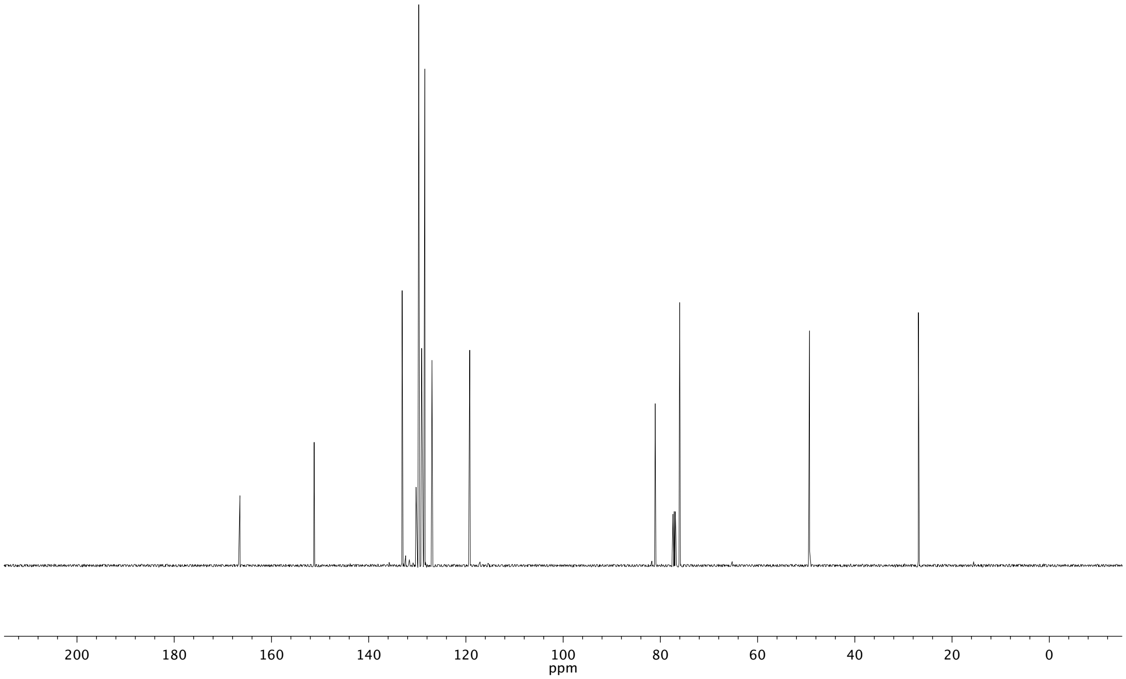


$^{13}$C NMR (126 MHz, $CDCl_3$) of compound **23**.

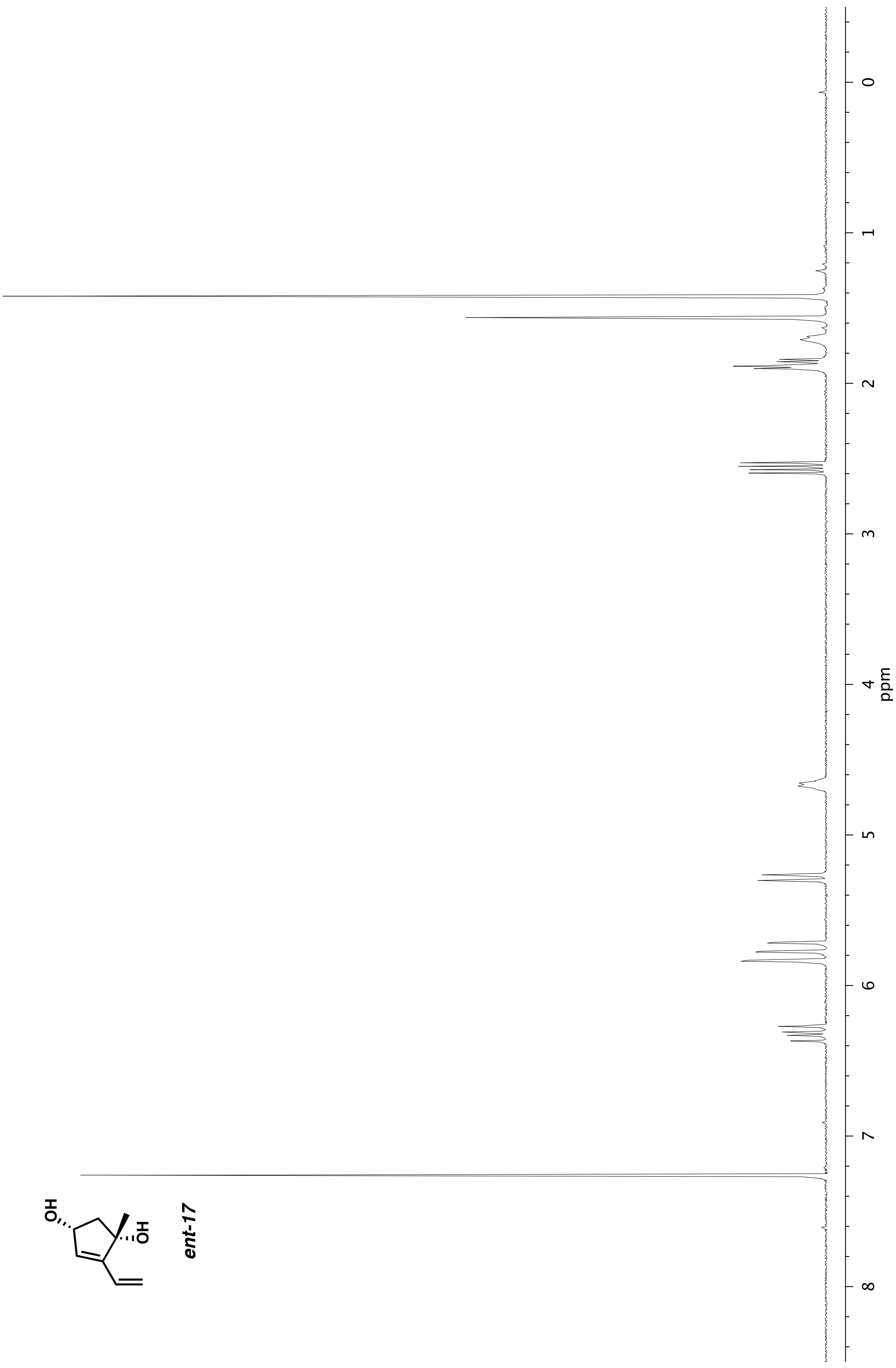


$^{1}$H NMR (300 MHz, $CDCl_3$) of compound ***ent*-17**.

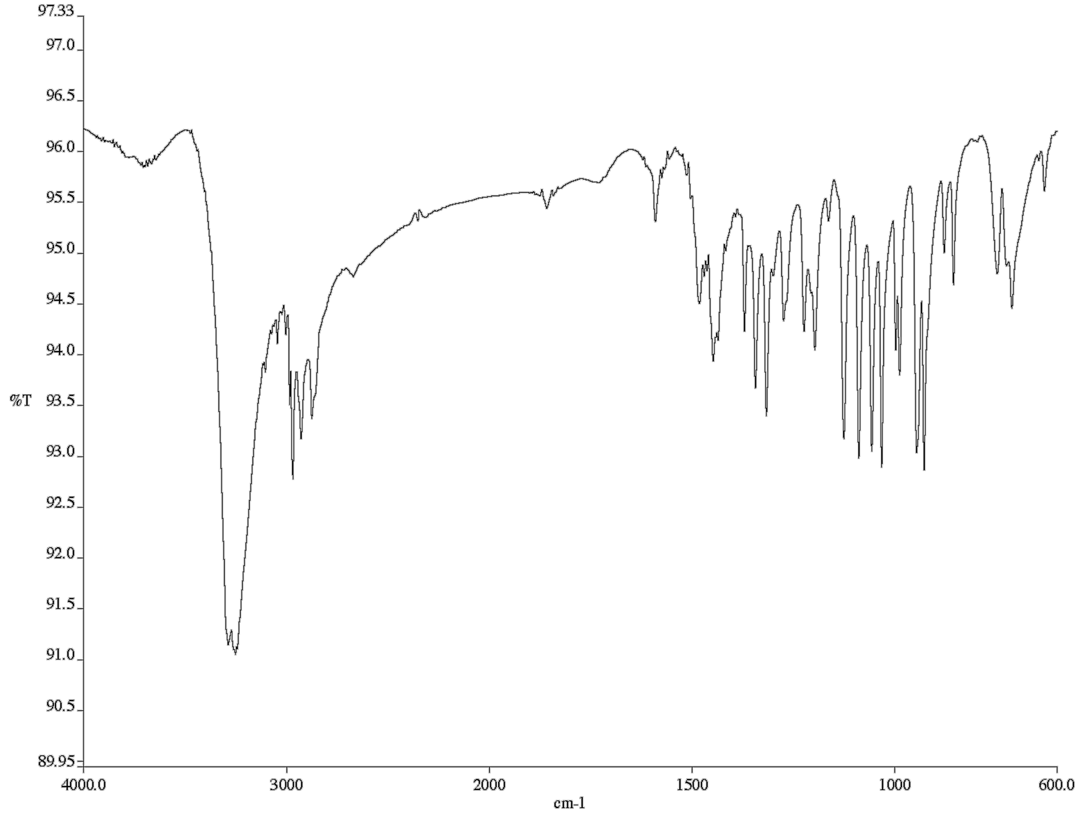


Infrared spectrum (Thin Film, NaCl) of compound ***ent*-17**.

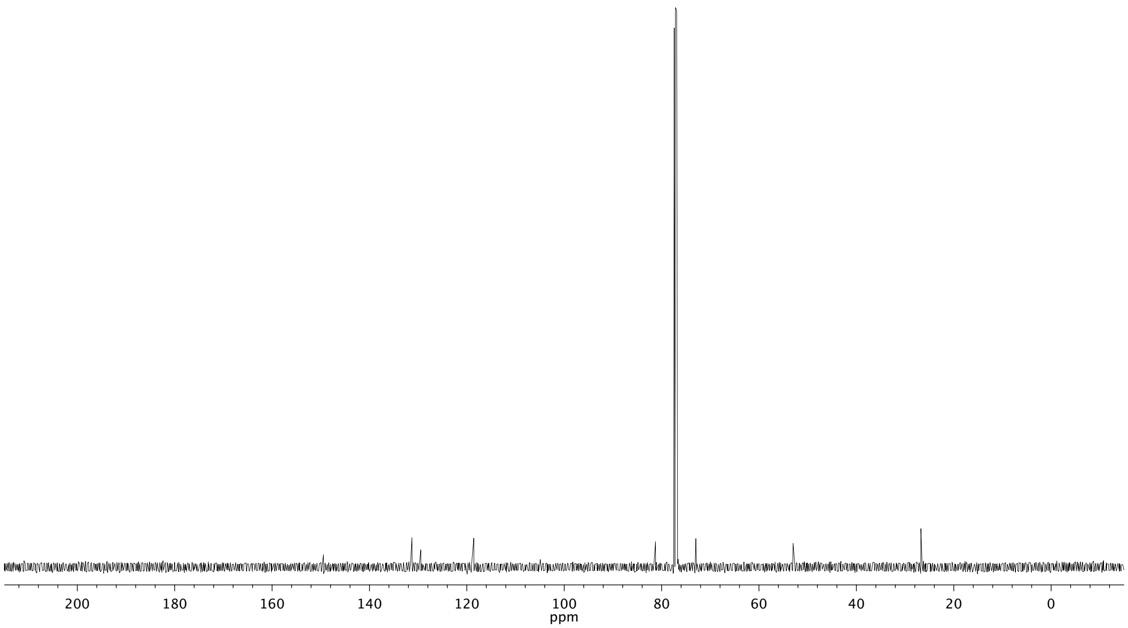


$^{13}$C NMR (76 MHz, $CDCl_3$) of compound ***ent*-17**.

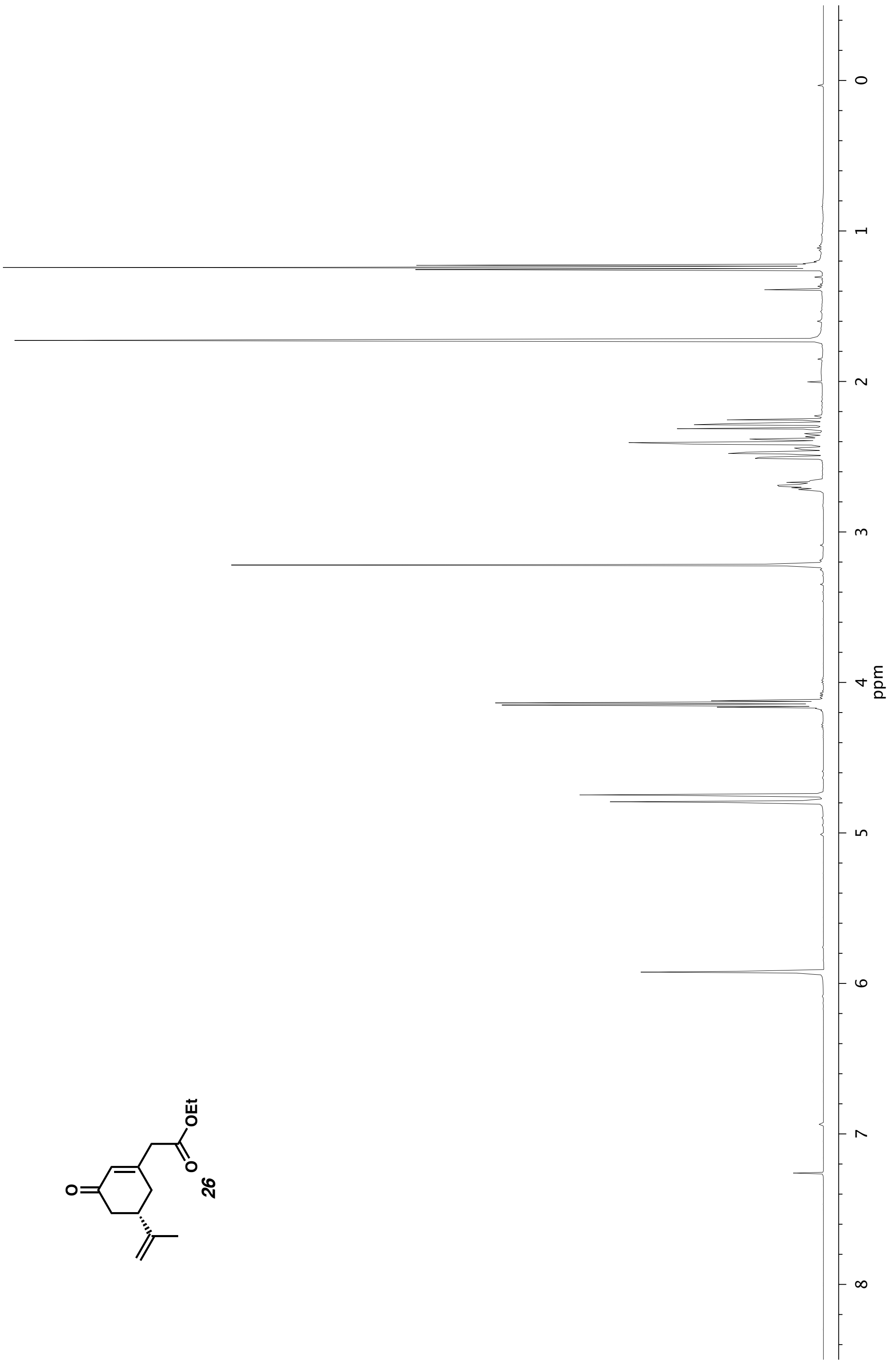


$^1H$ NMR (500 MHz, $CDCl_3$) of compound **26**.

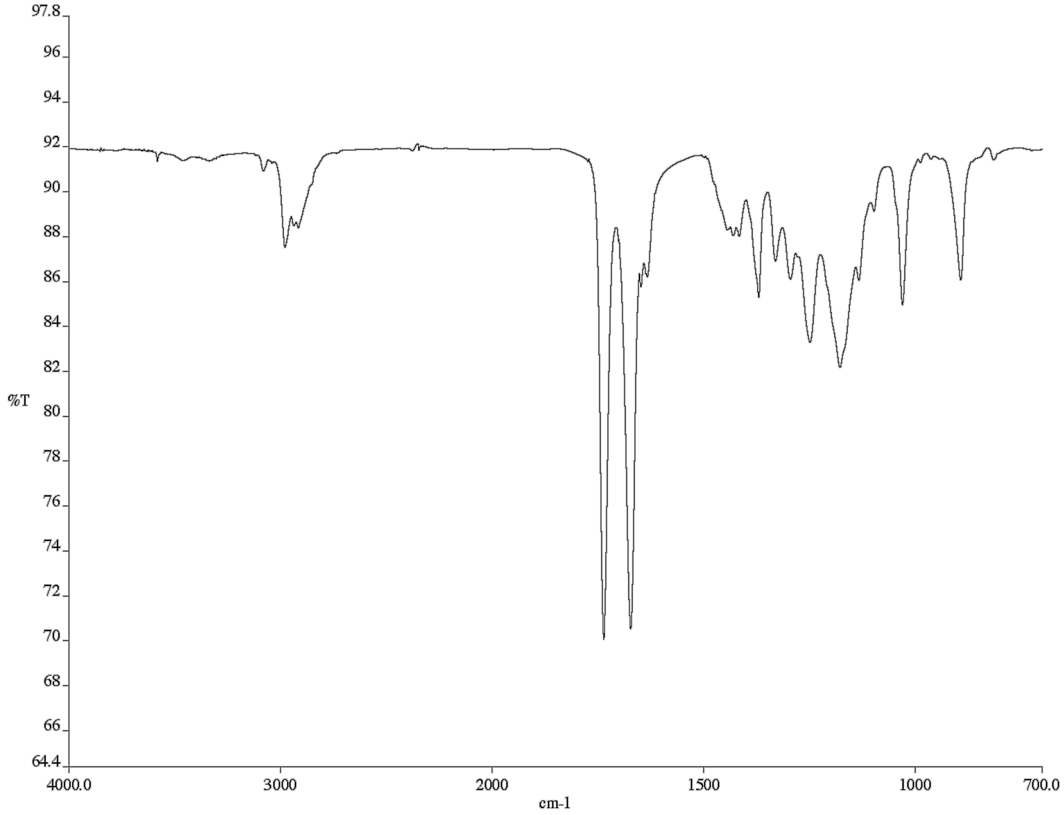


Infrared spectrum (Thin Film, NaCl) of compound **26**.

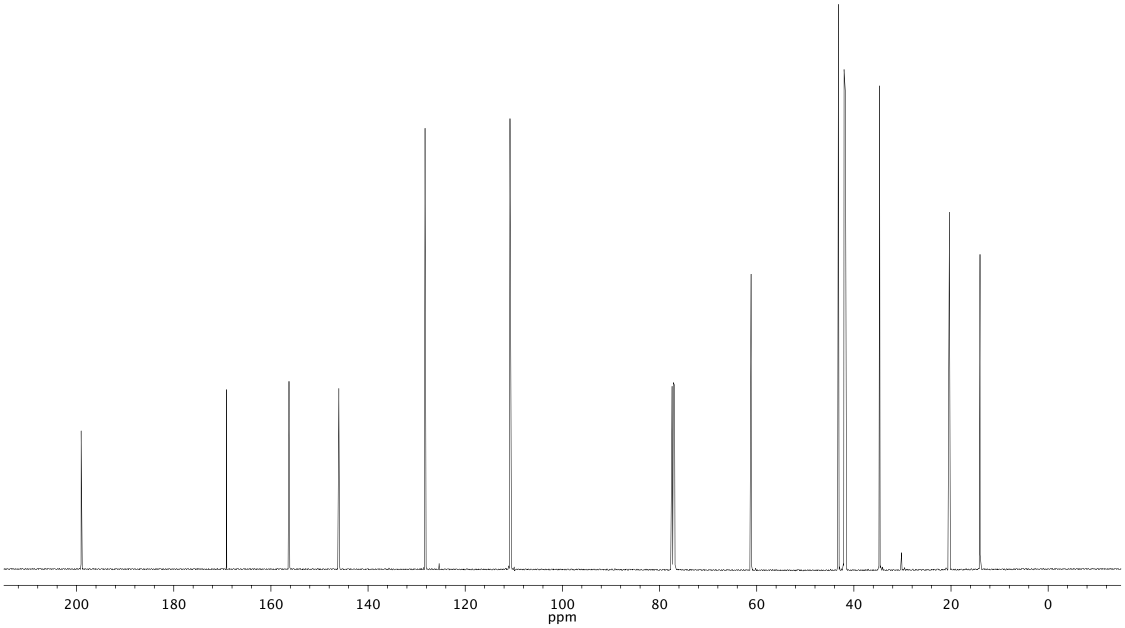


$^{13}C$ NMR (126 MHz, $CDCl_3$) of compound **26**.

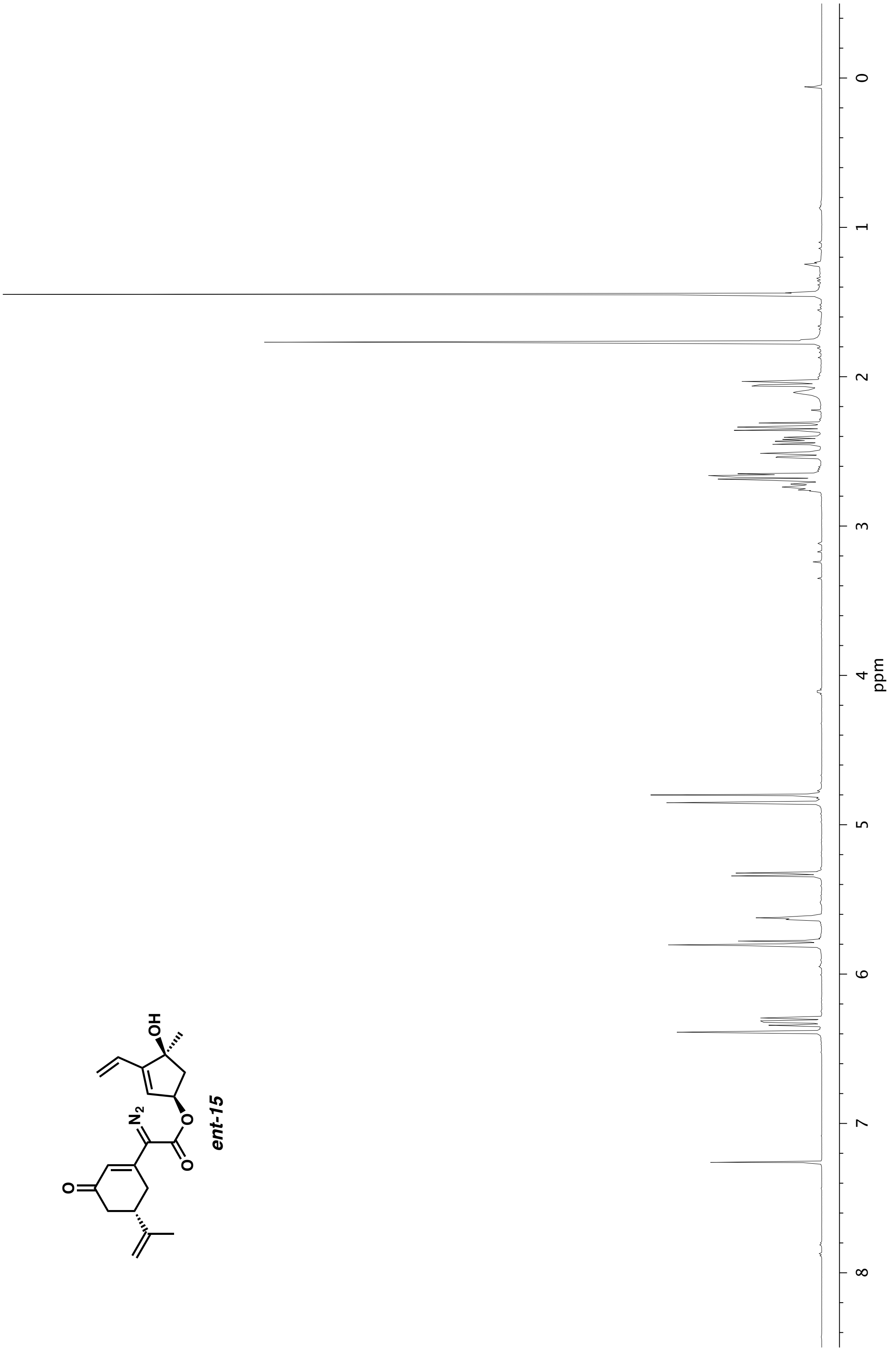


$^{1}H$ NMR (600 MHz, $CDCl_3$) of compound ***ent*-15**.

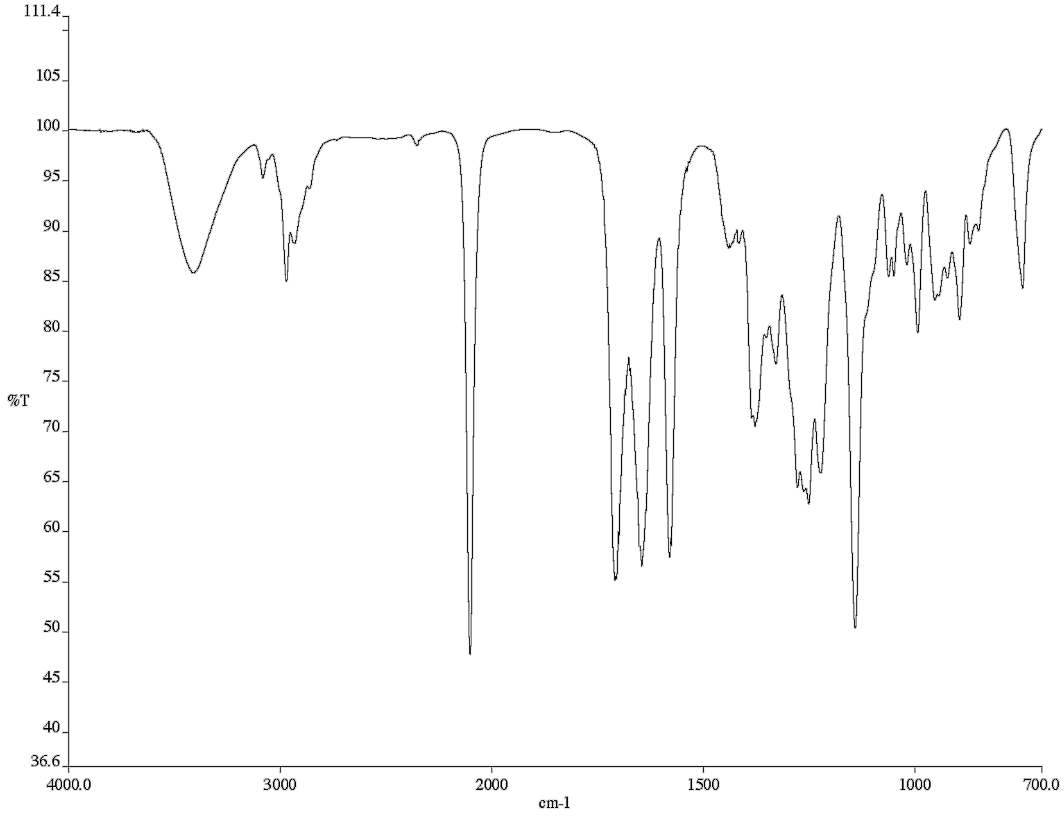


Infrared spectrum (Thin Film, NaCl) of compound ***ent*-15**.

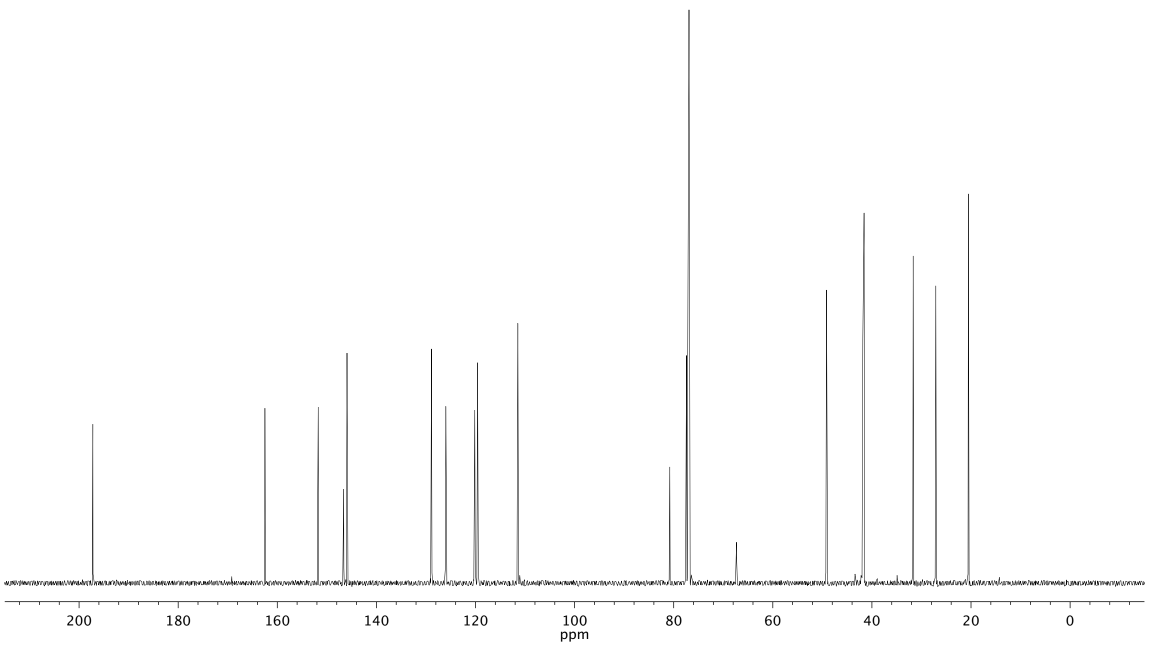


$^{13}$C NMR (126 MHz, $CDCl_3$) of compound ***ent*-15**.

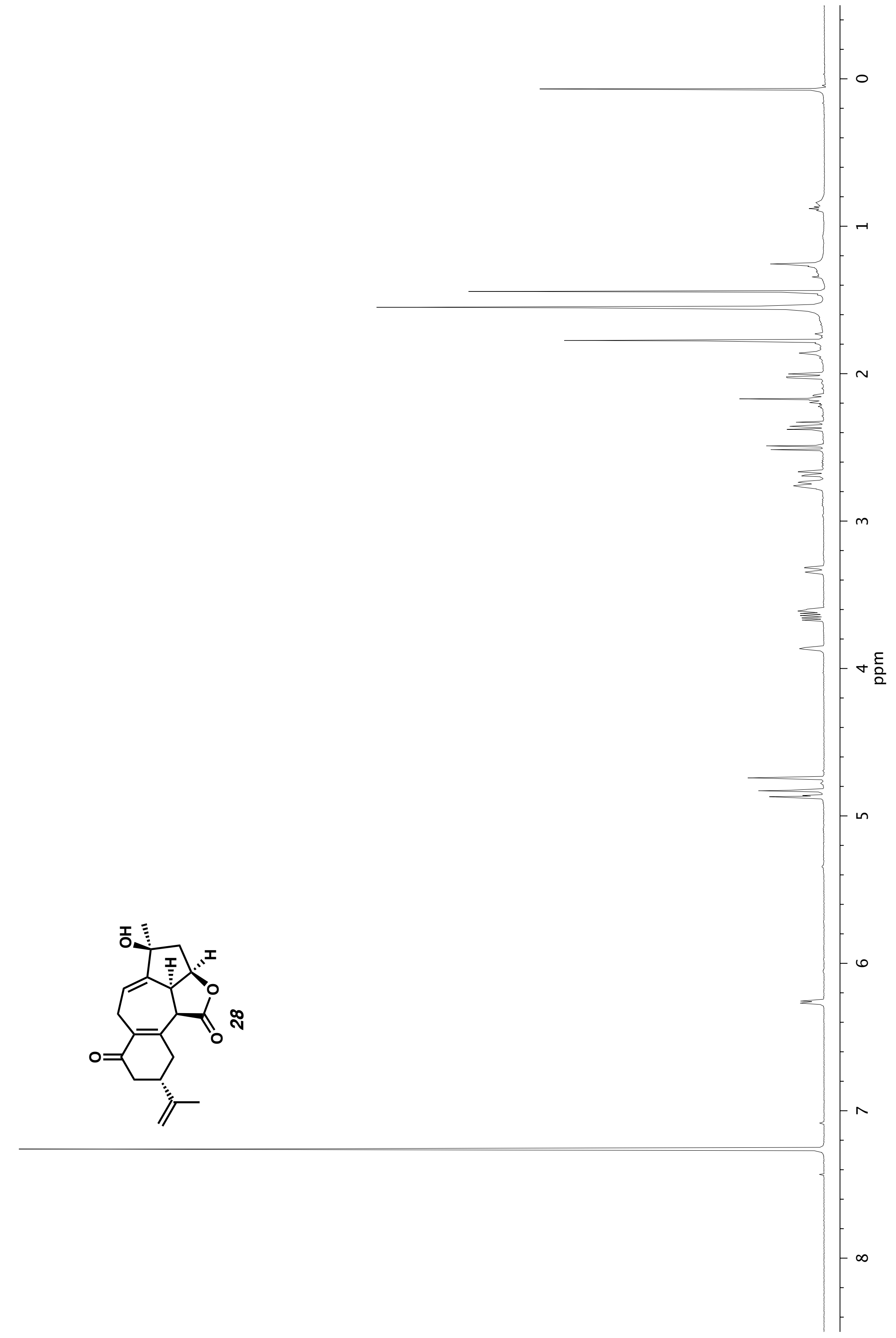


$^1$H NMR (500 MHz, $CDCl_3$) of compound **28**.

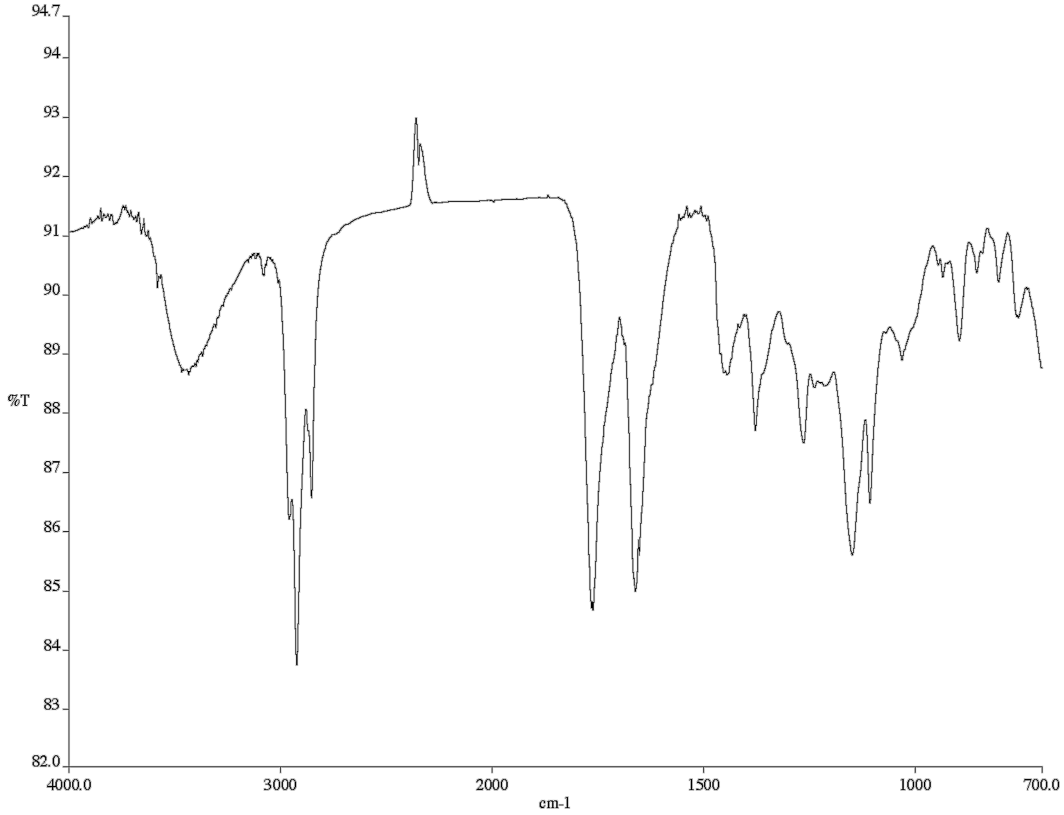


Infrared spectrum (Thin Film, NaCl) of compound **28**.

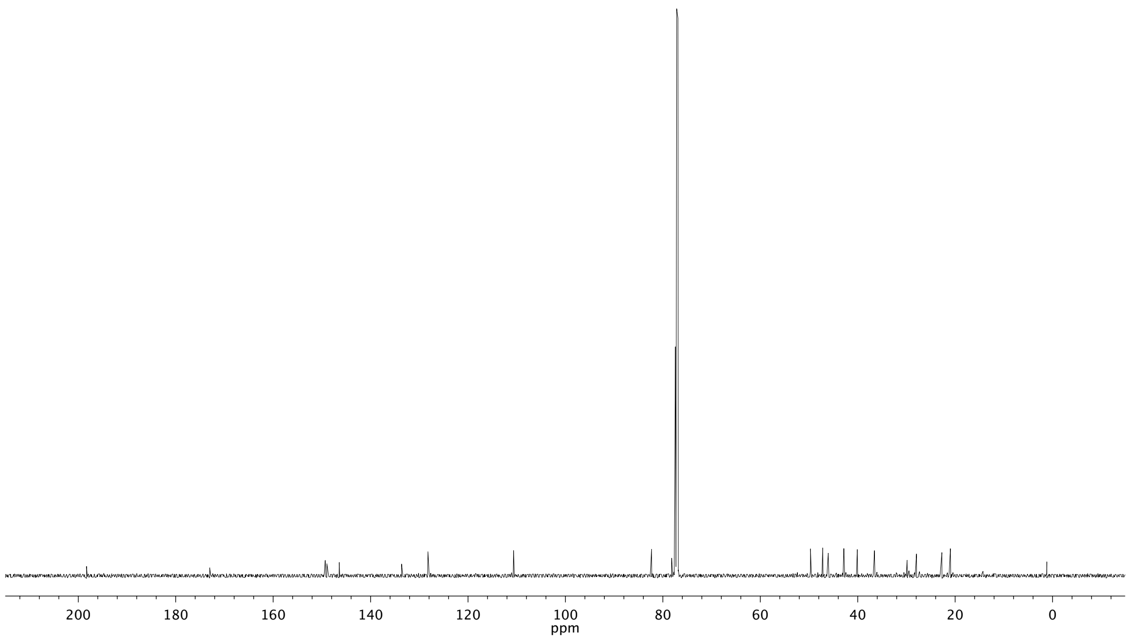


$^{13}C$ NMR (126 MHz, $CDCl_3$) of compound **28**.

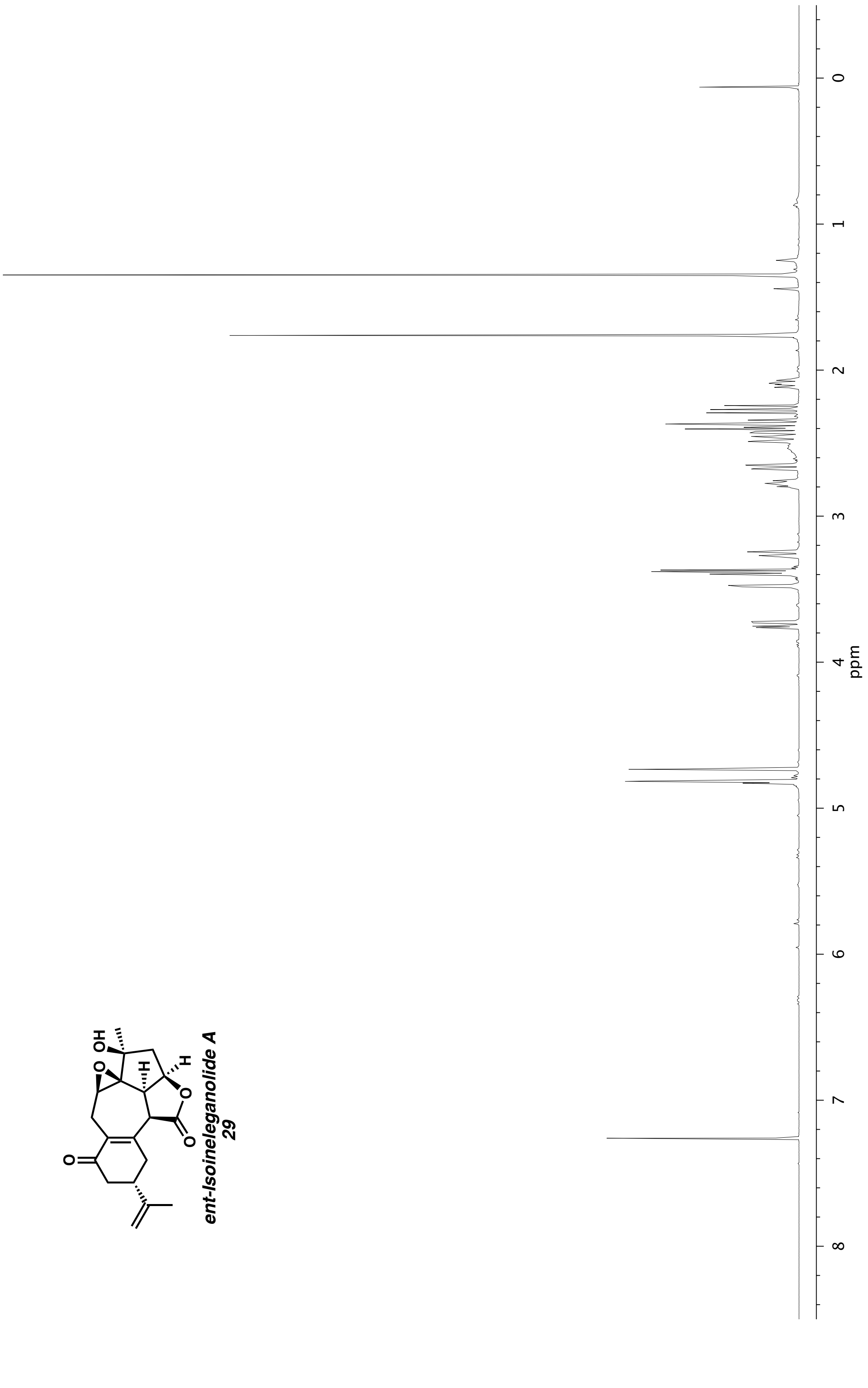


$^1$H NMR (600 MHz, $CDCl_3$) of compound **29**.

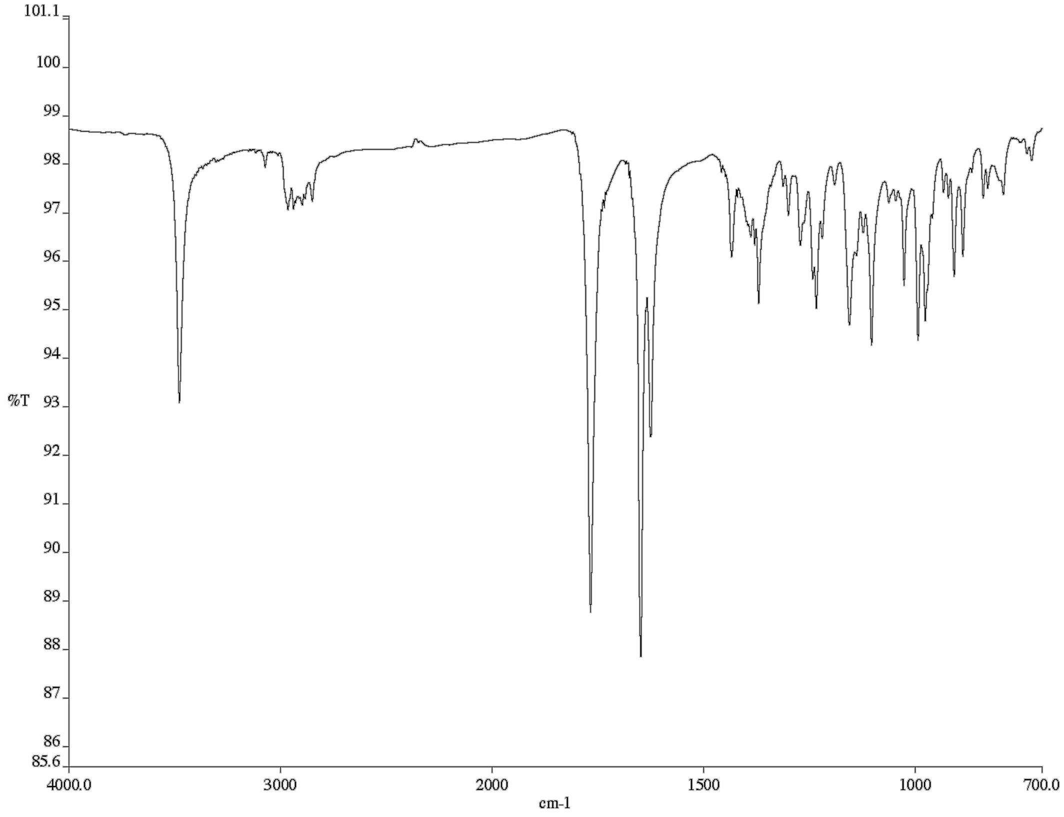


Infrared spectrum (Thin Film, NaCl) of compound **29**.

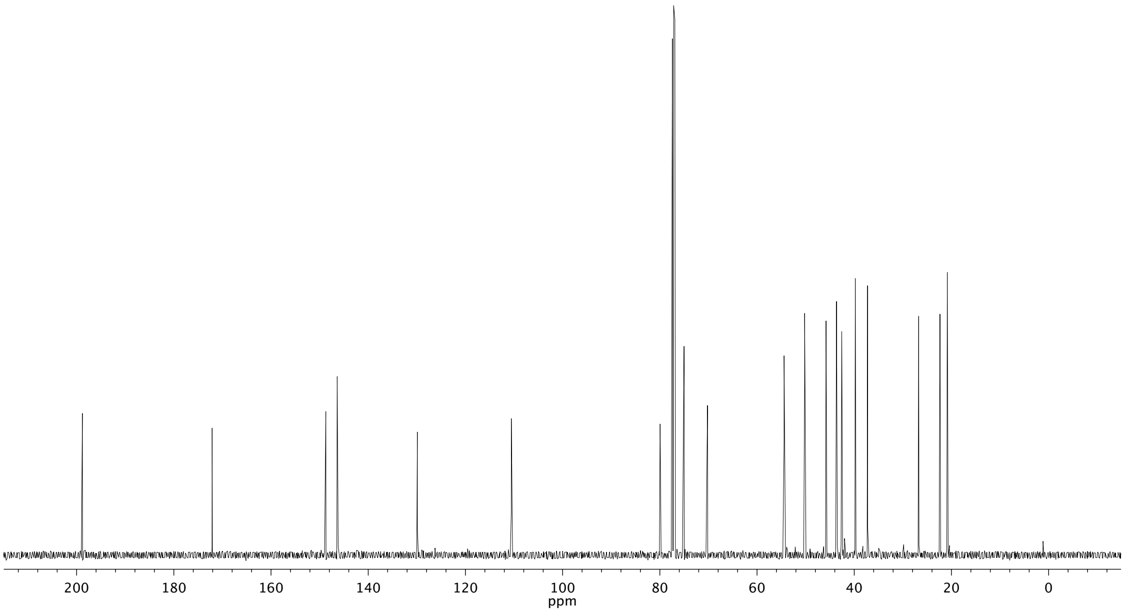


$^{13}$C NMR (126 MHz, $CDCl_3$) of compound **29**.

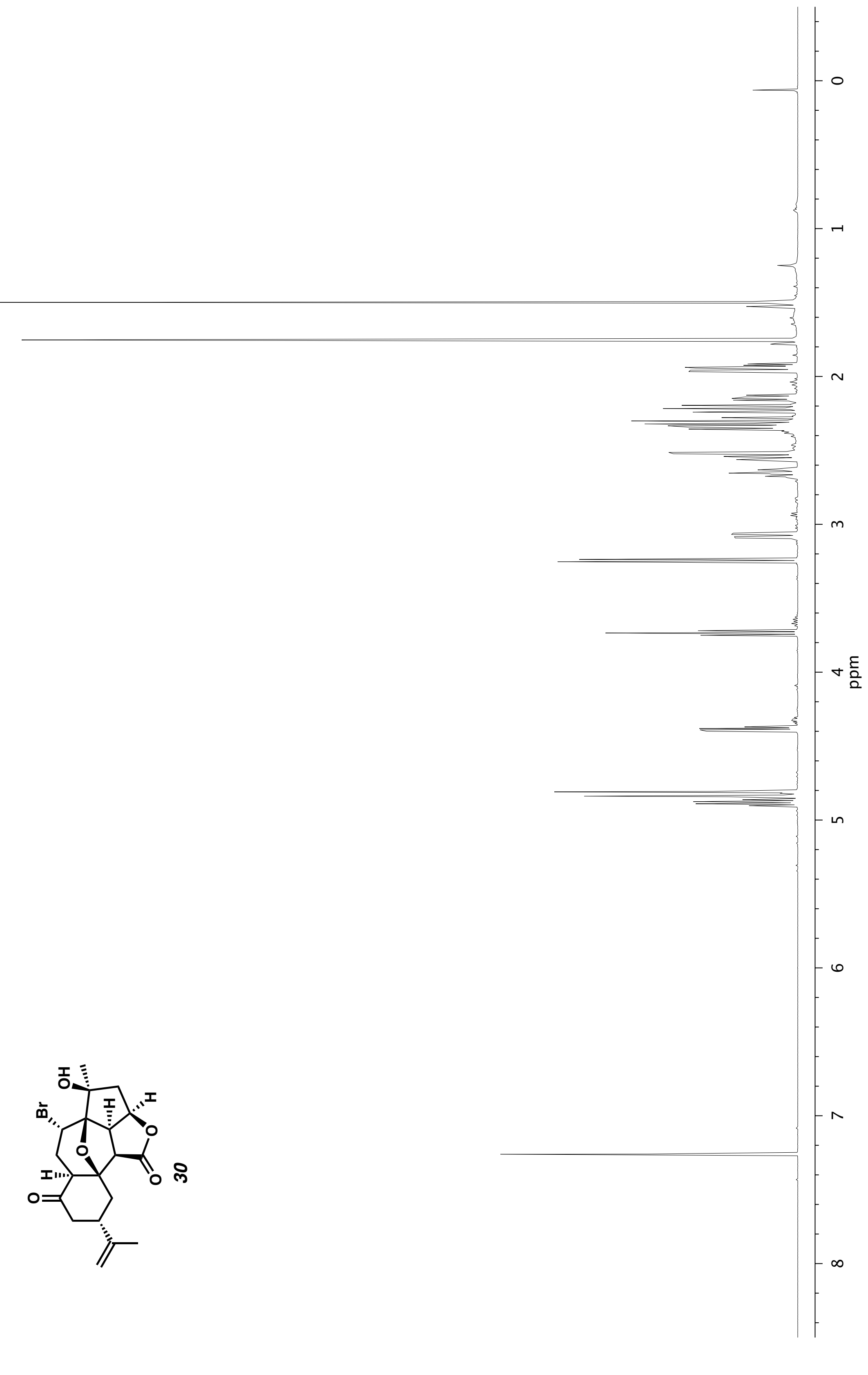


$^1H$ NMR (600 MHz, $CDCl_3$) of compound **30**.

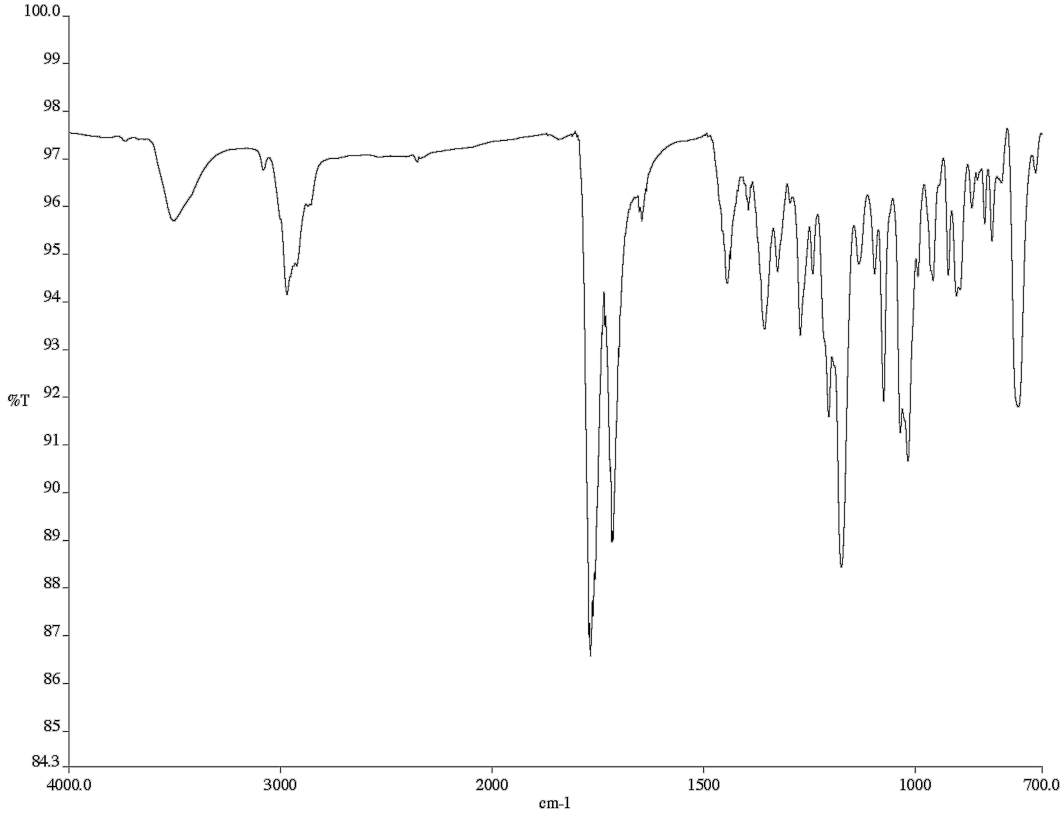


Infrared spectrum (Thin Film, NaCl) of compound **30**.

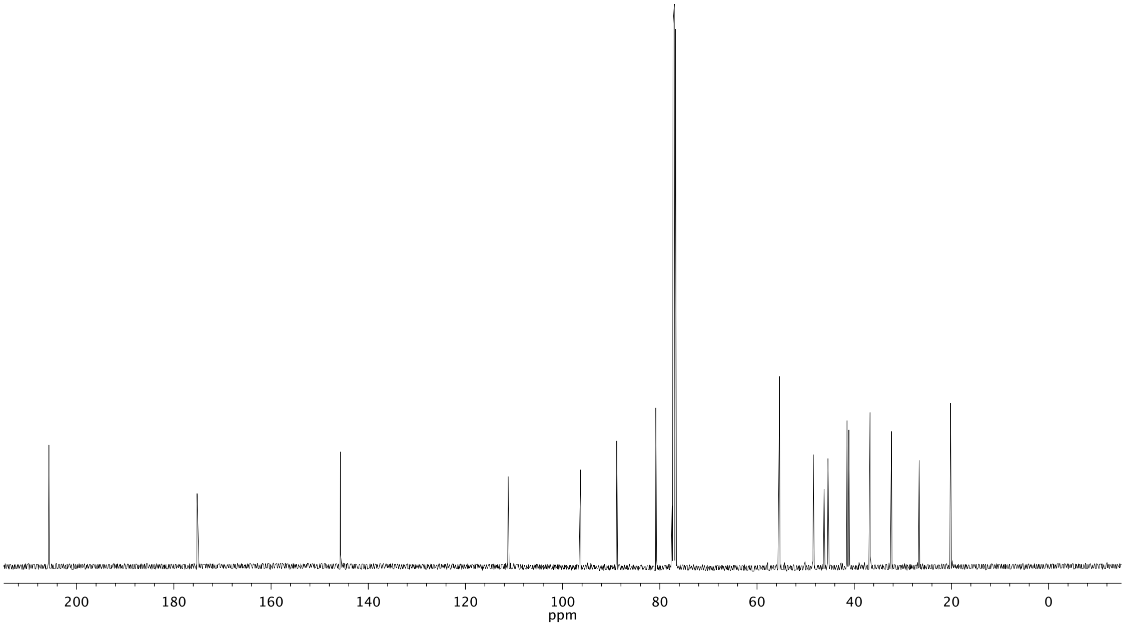


$^{13}$C NMR (126 MHz, $CDCl_3$) of compound **30**.

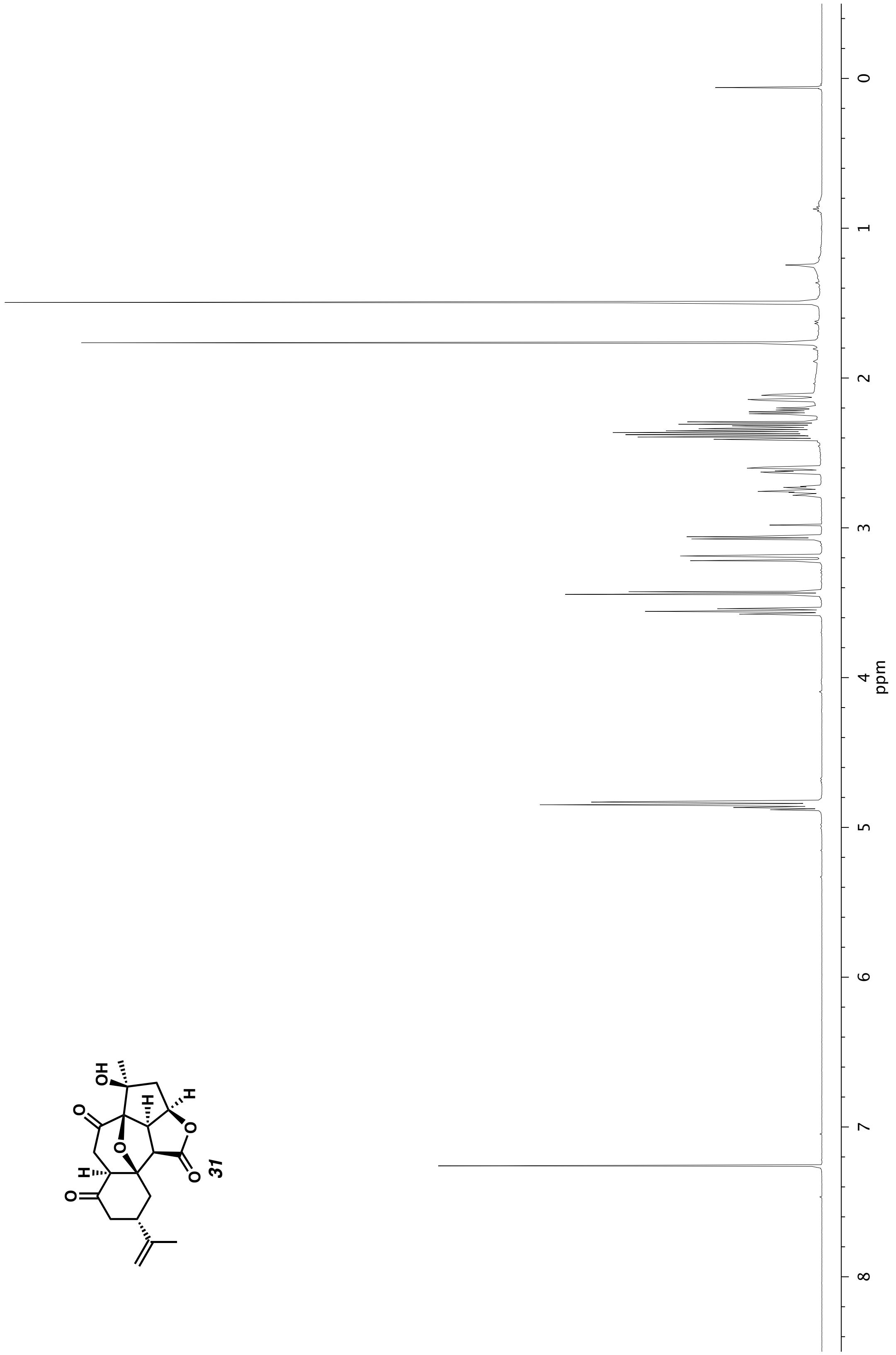


$^1$H NMR (500 MHz, $CDCl_3$) of compound **31**.

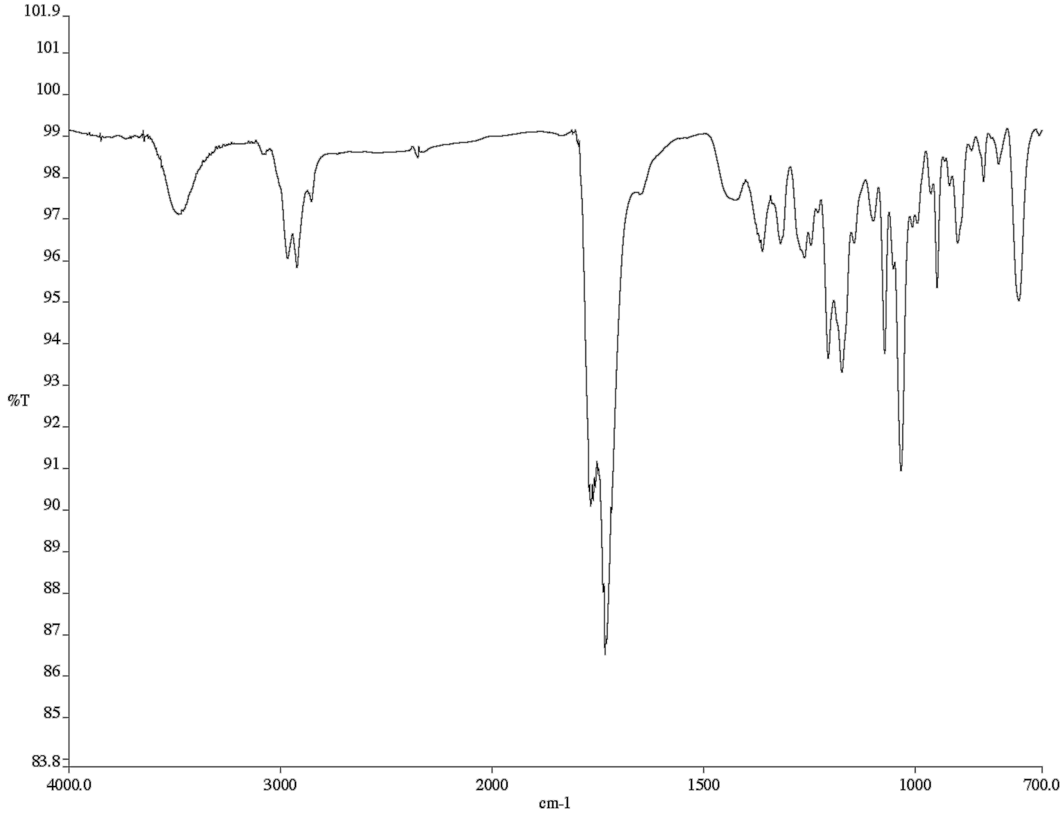


Infrared spectrum (Thin Film, NaCl) of compound **31**.

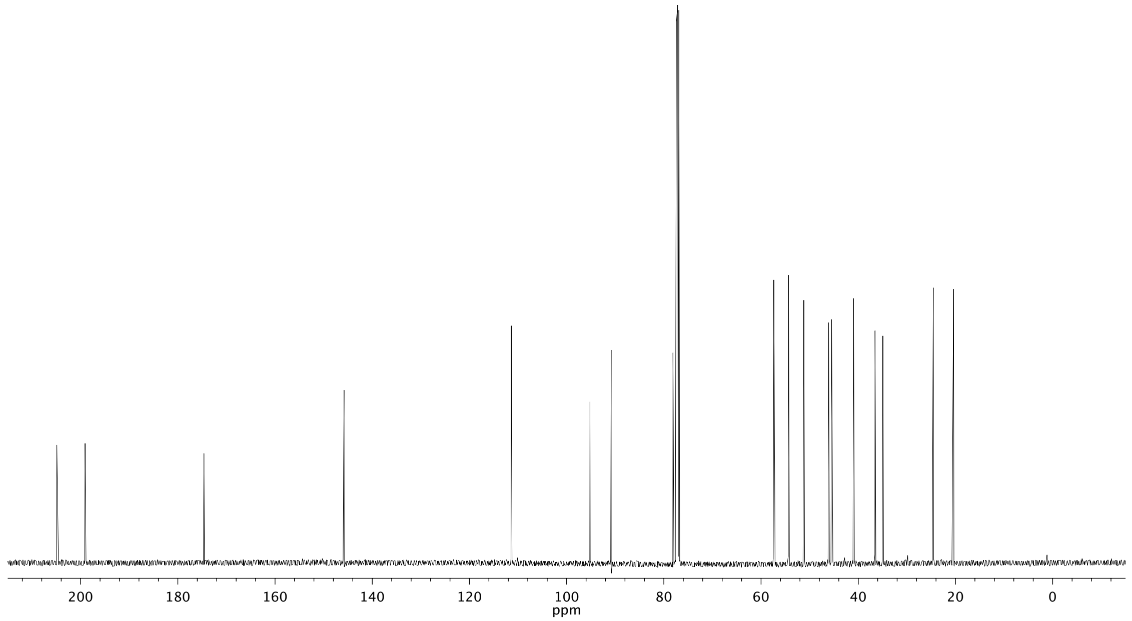


$^{13}$C NMR (126 MHz, $CDCl_3$) of compound **31**.

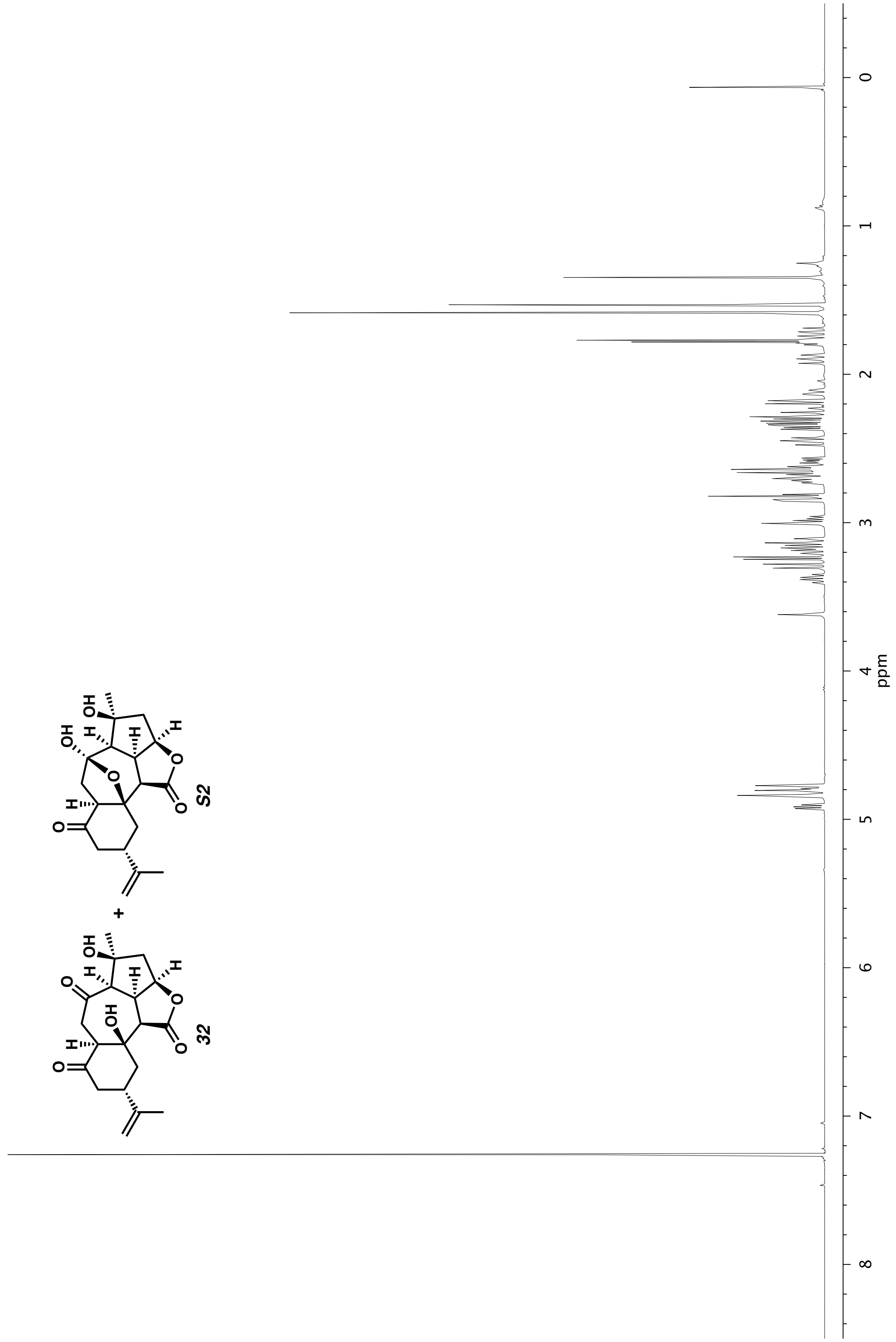


$^1$H NMR (500 MHz, $CDCl_3$) of compound **32/S2**.

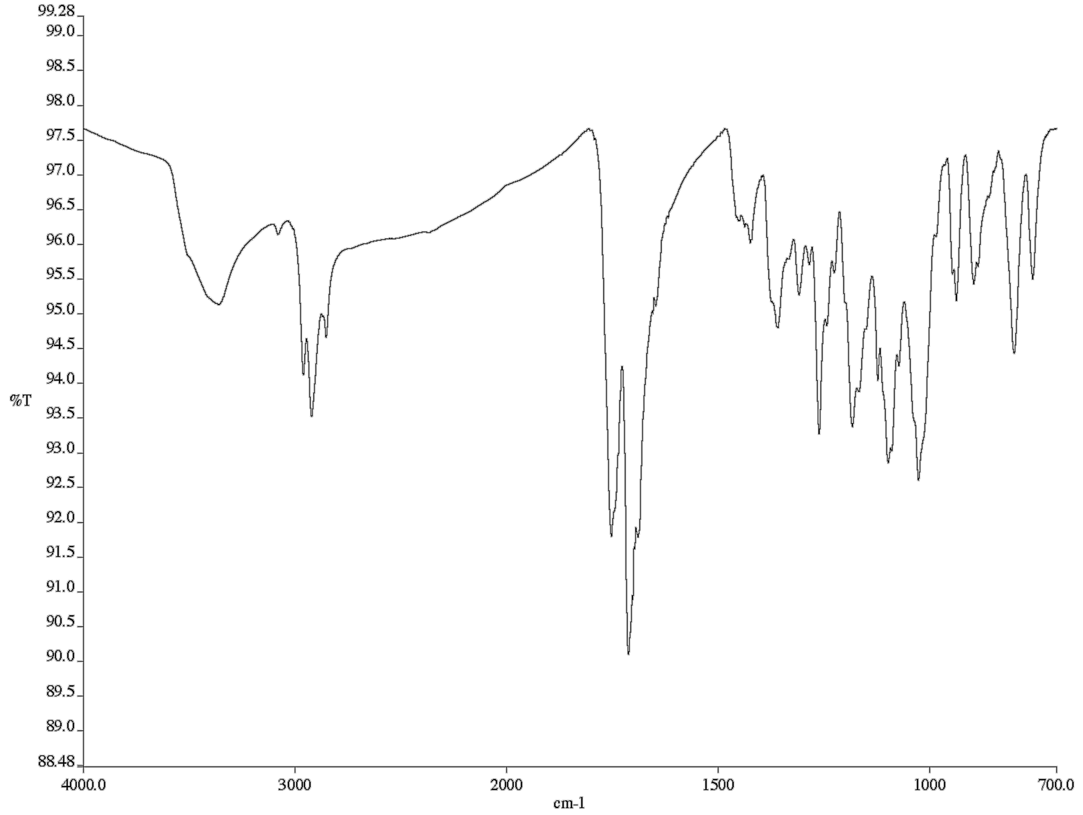


Infrared spectrum (Thin Film, NaCl) of compound **32**/**S2**.

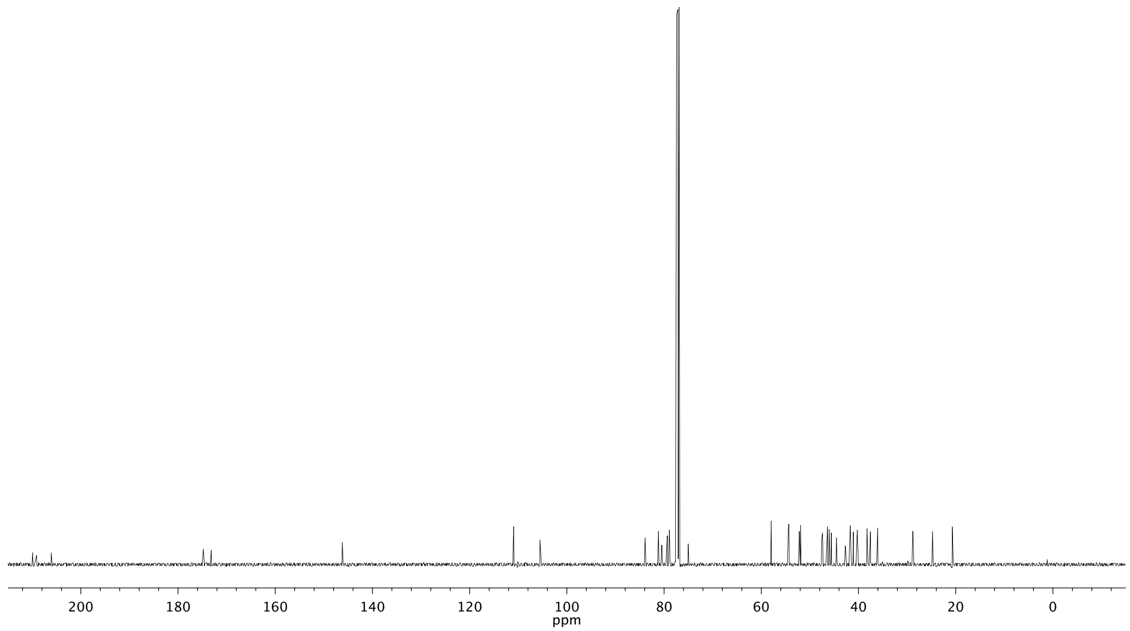


$^{13}C$ NMR (126 MHz, $CDCl_3$) of compound **32**/**S2**.

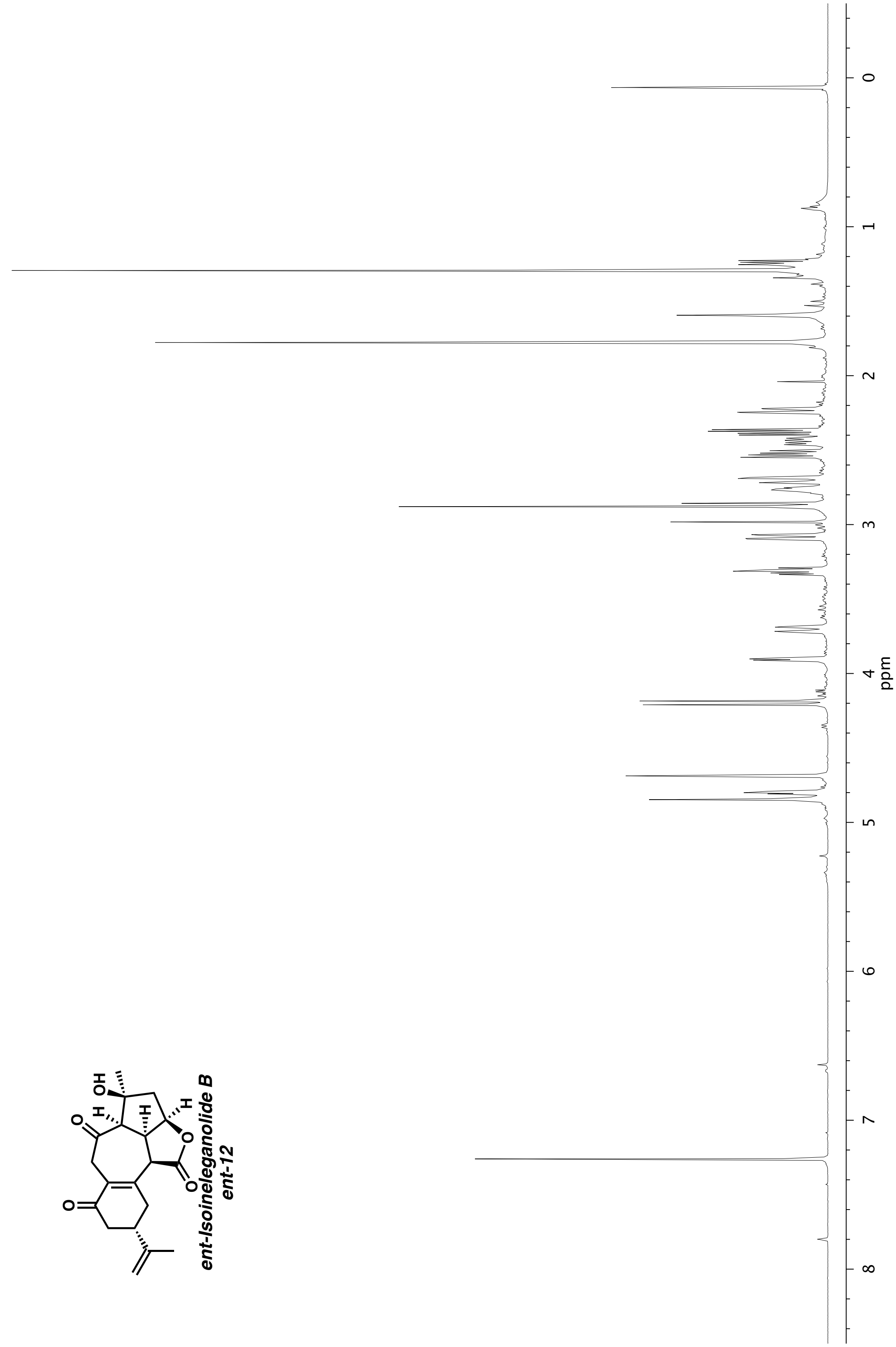


$^{1}$H NMR (600 MHz, $CDCl_3$) of compound ***ent*-12**.

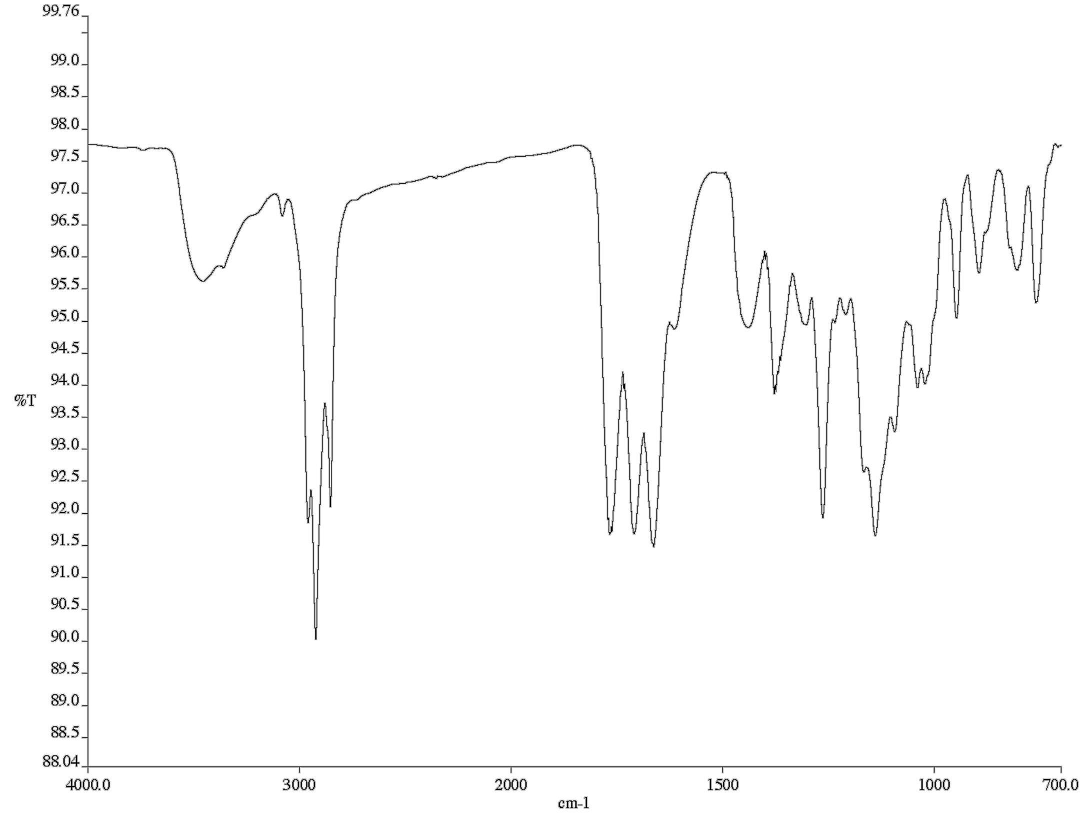


Infrared spectrum (Thin Film, NaCl) of compound ***ent*-12**.

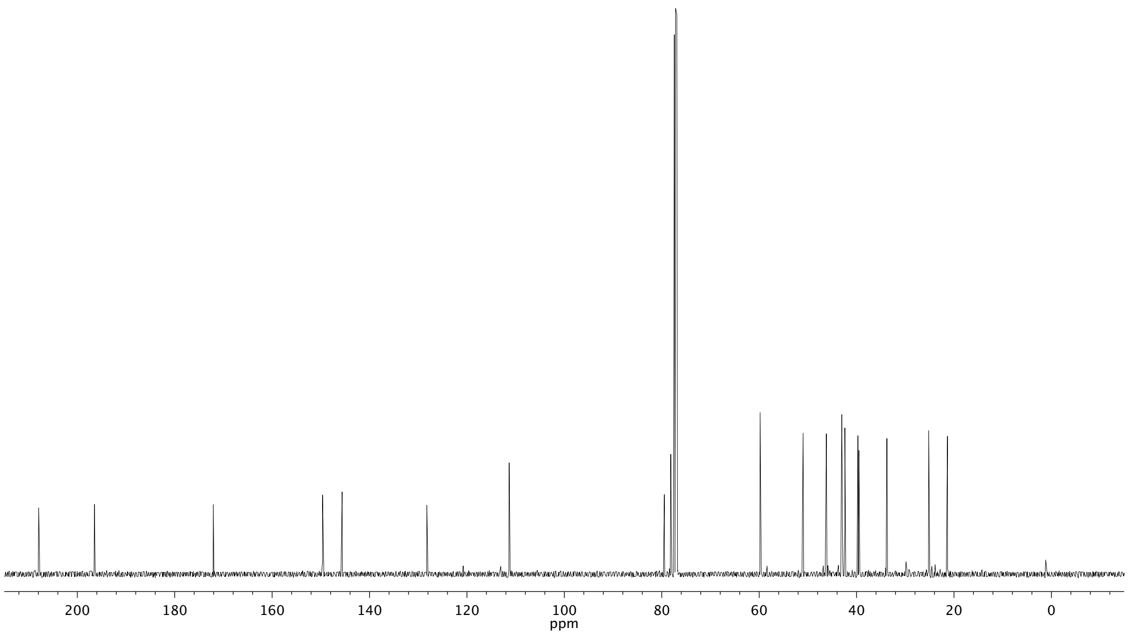


$^{13}$C NMR (126 MHz, $CDCl_3$) of compound ***ent*-12**.

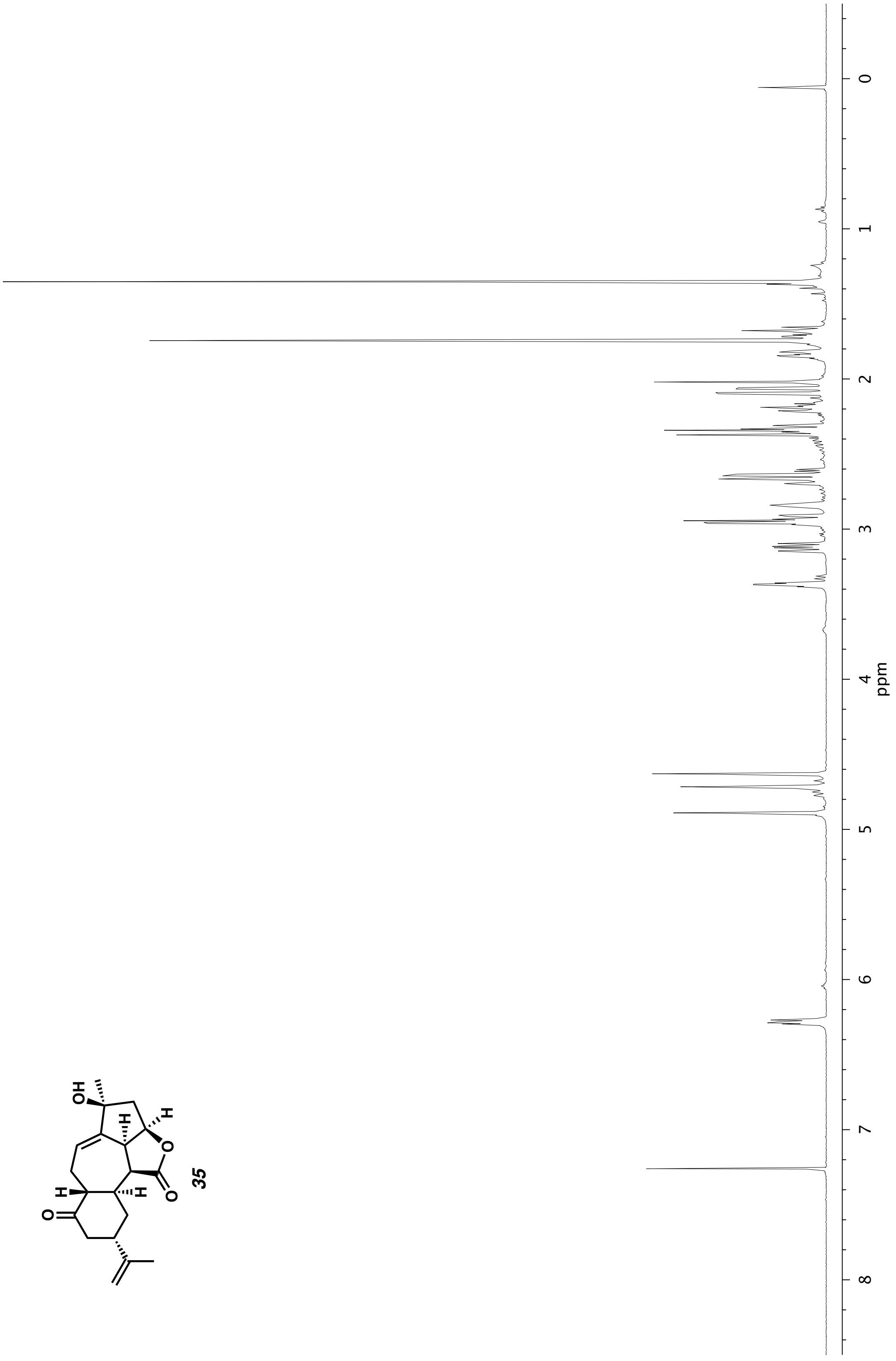


$^1H$ NMR (500 MHz, $CDCl_3$) of compound **35**.

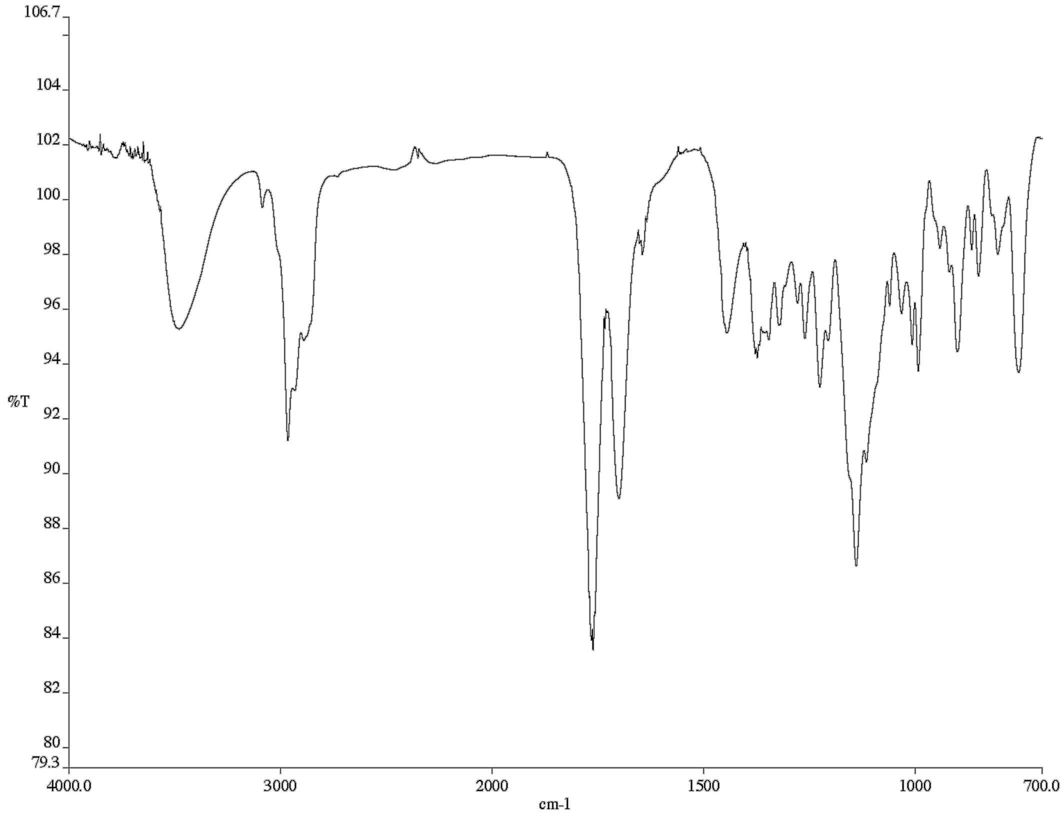


Infrared spectrum (Thin Film, NaCl) of compound **35**.

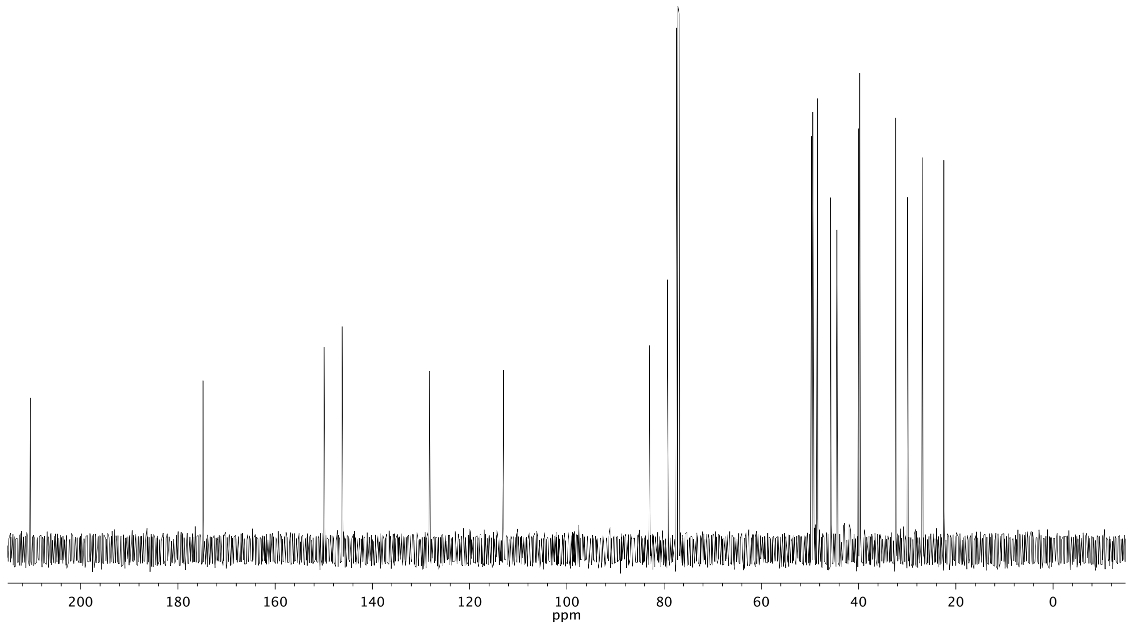


$^{13}C$ NMR (126 MHz, $CDCl_3$) of compound **35**.

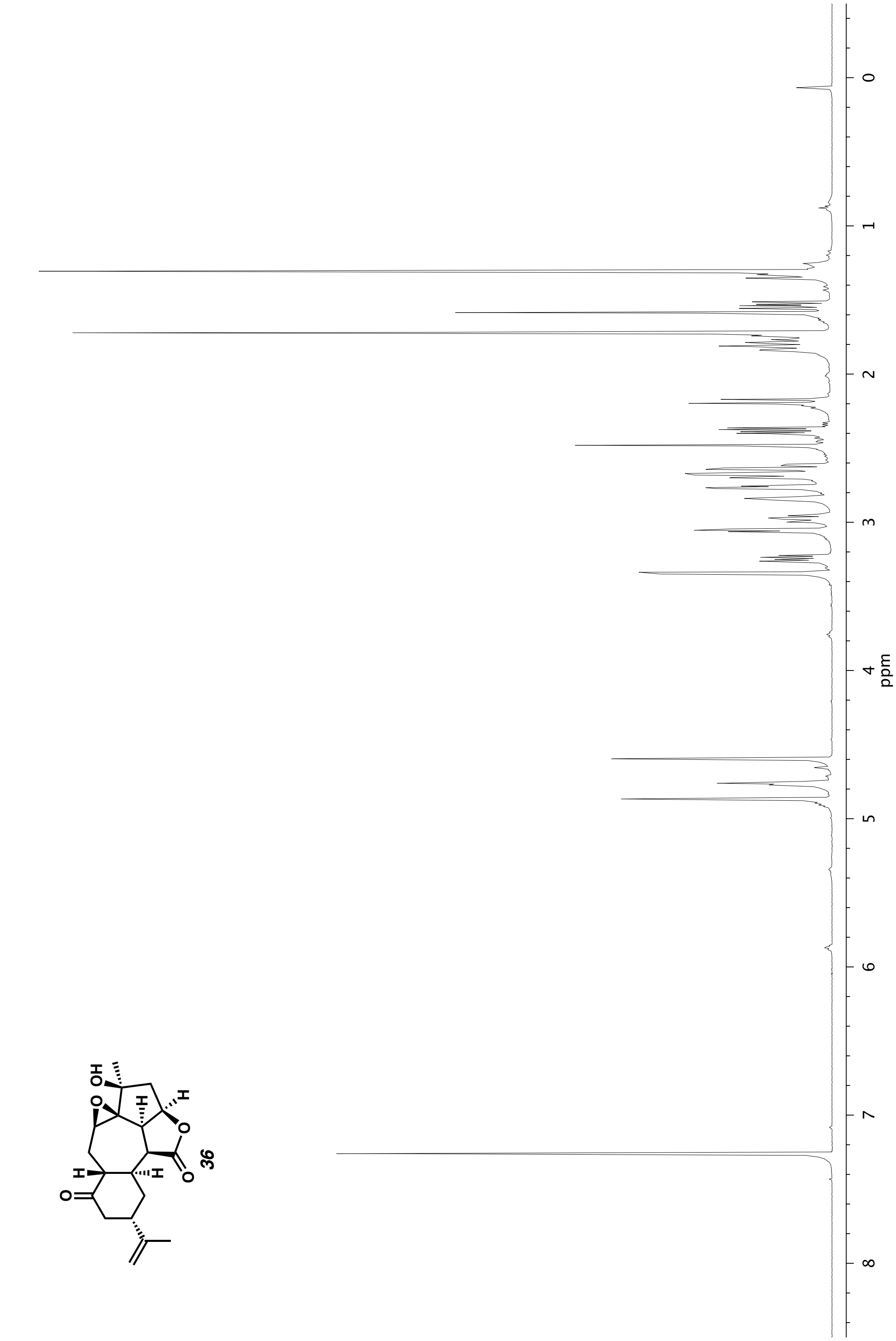


$^1H$ NMR (600 MHz, $CDCl_3$) of compound **36**.

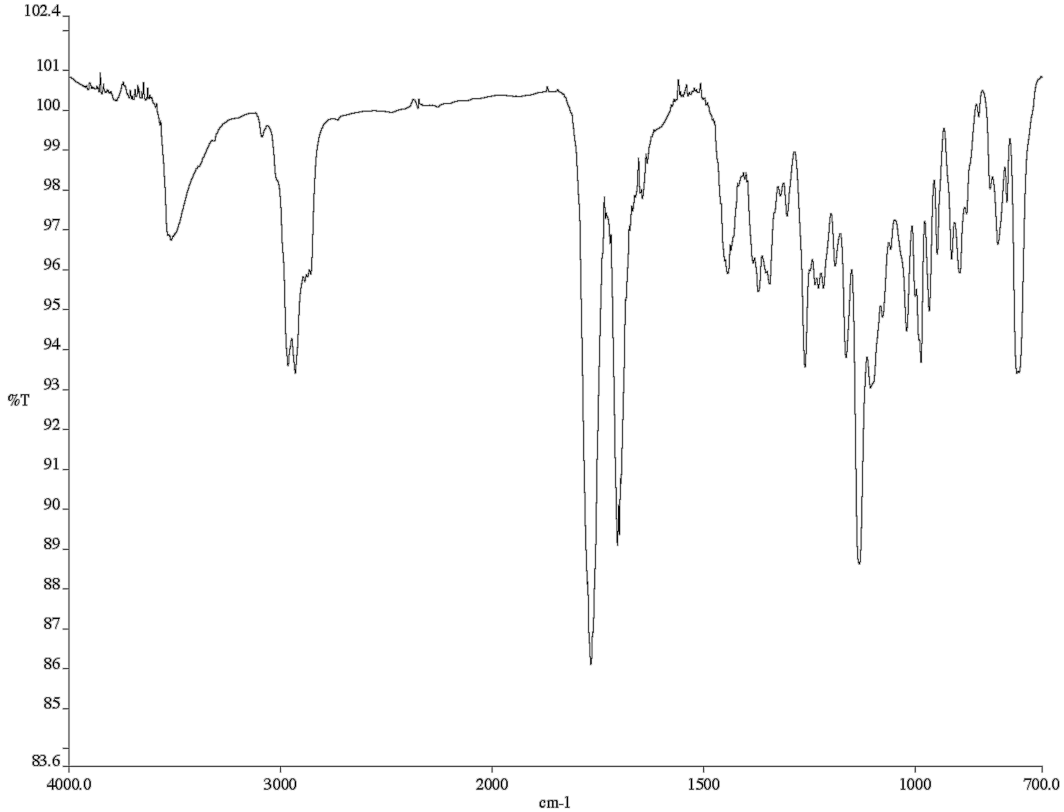


Infrared spectrum (Thin Film, NaCl) of compound **36**.

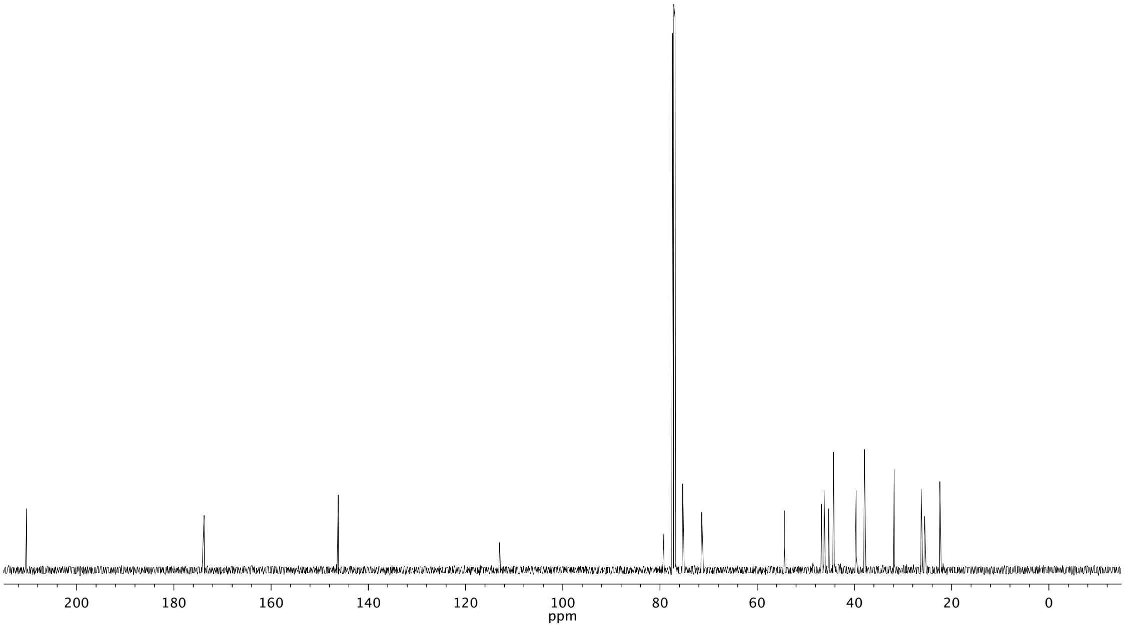


$^{13}C$ NMR (126 MHz, $CDCl_3$) of compound **36**.

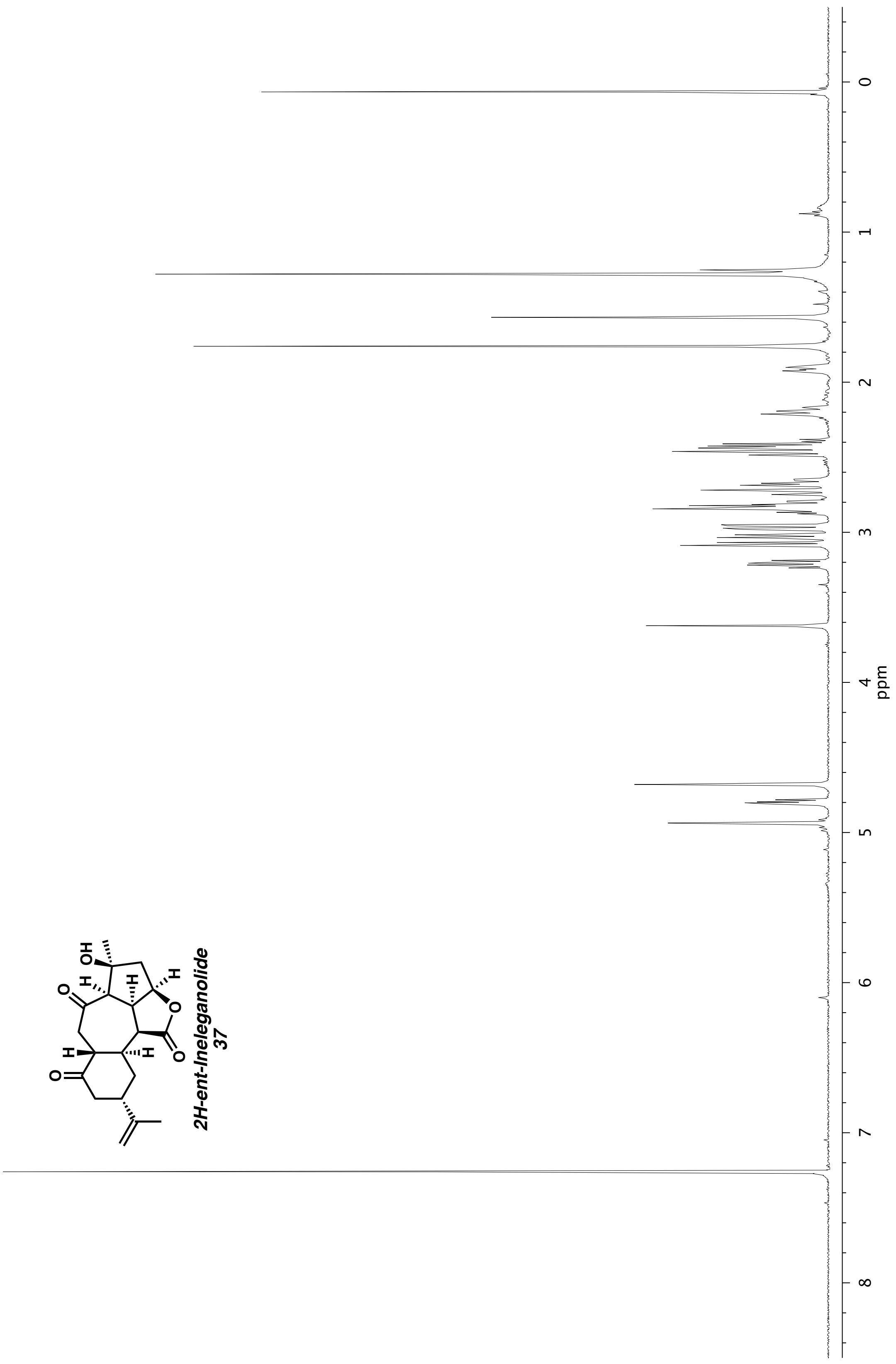


$^{1}H$ NMR (500 MHz, $CDCl_3$) of compound **37**.

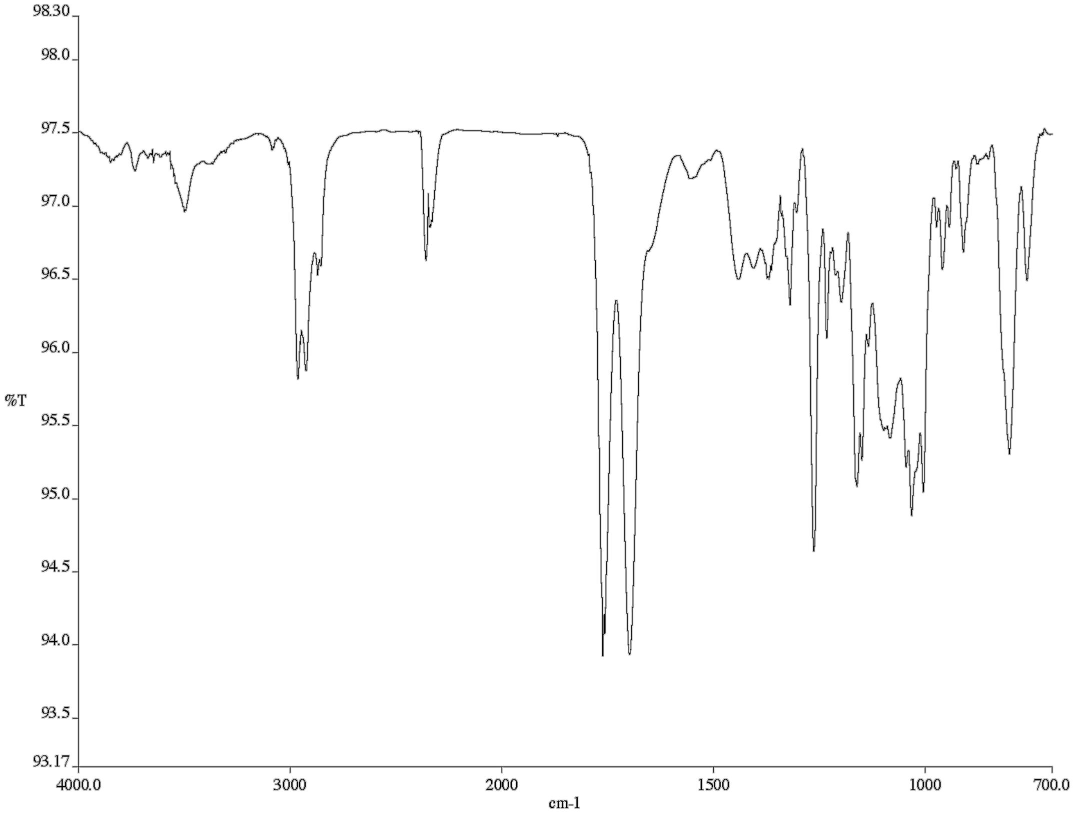


Infrared spectrum (Thin Film, NaCl) of compound **37**.

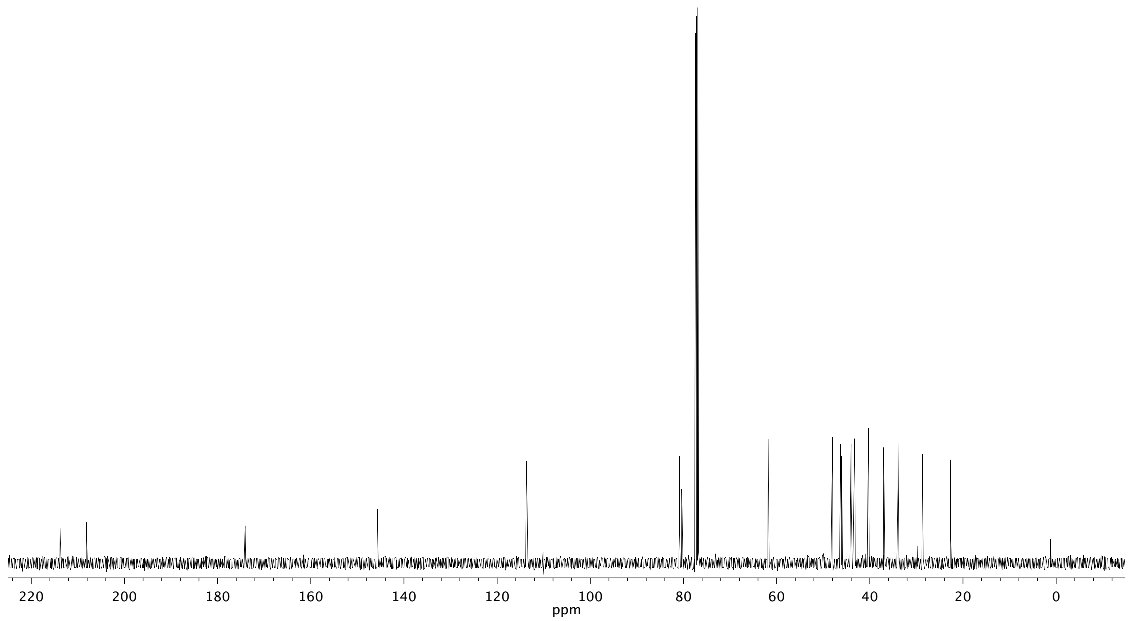


$^{13}$C NMR (126 MHz, $CDCl_3$) of compound **37**.

## X-Ray Crystal Structure Analysis of Diene 28

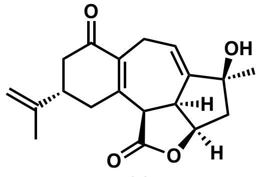


***28***

Contents

Table 1. Experimental Details

Table 2. Crystal Data

Table 3. Atomic Coordinates

Table 4. Full Bond Distances and Angles

Table 5. Anisotropic Displacement Parameters

Table 6. Hydrogen Atomic Coordinates

Table 7. Torsion Angles

Table 8. Hydrogen Bond Distances and Angle

*X-Ray Crystal Structure of Diene* ***28***

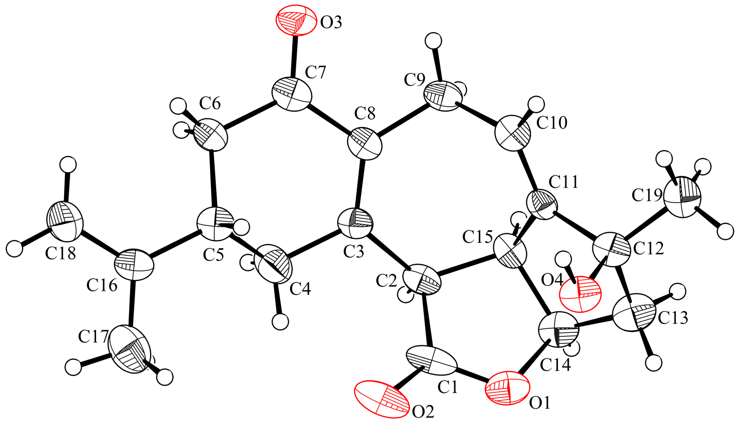


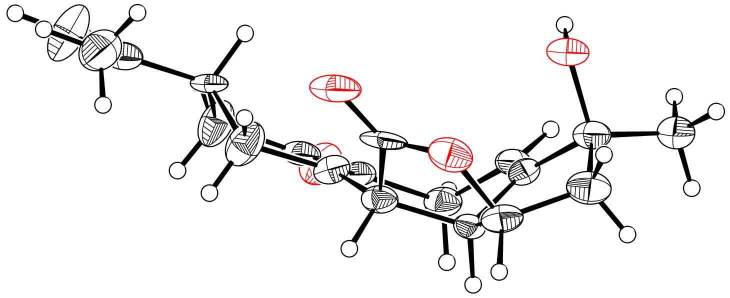

*Table 1. Experimental Details for X-Ray Structure Determination of Diene **28**.*

Low-temperature diffraction data ($\phi$-and $\omega$-scans) were collected on a Bruker AXS KAPPA APEX II diffractometer coupled to a APEX II CCD detector with graphite monochromated Mo $K_\alpha$ radiation ($\lambda$ = 0.71073 Å) for the structure of diene **28**. The structure was solved by direct methods using SHELXS[i] and refined against $F^2$ on all data by full-matrix least squares with SHELXL-2014[ii] using established refinement techniques.[iii] All non-hydrogen atoms were refined anisotropically. All hydrogen atoms were included into the model at geometrically calculated positions and refined using a riding model. The isotropic displacement parameters of all hydrogen atoms were fixed to 1.2 times the *U* value of the atoms they are linked to (1.5 times for methyl groups).

Diene **28** crystallizes in the monoclinic space group $P2_1$ with one molecule in the asymmetric unit. The coordinates for the hydrogen atom bound to O4 was located in the difference Fourier synthesis and refined semi-freely with the help of a restraint on the O-H distance (0.84(4) Å). The crystal diffracted to 0.93 Å leading to low pond precision. The structure is sufficient to determine the relative stereochemistry of the molecule.

[i] G. M. Sheldrick, *Acta Cryst*. **1990**, A46, 467–473.
[ii] G. M. Sheldrick, *Acta Cryst*. **2008**, A64, 112–122.
[iii] P. Müller, *Crystallography Reviews* **2009**, *15*, 57–83.

*Table 2. Crystal Data and Structure Refinement for Diene **28**.*

| | | |
|---|---|---|
| Caltech Identification code | rac01 | |
| CCDC Deposition Number | 1061010 | |
| Empirical formula | C19 H22 O4 | |
| Formula weight | 314.36 | |
| Temperature | 100(2) K | |
| Wavelength | 0.71073 Å | |
| Crystal system | Monoclinic | |
| Space group | $P2_1$ | |
| Unit cell dimensions | a = 11.7563(19) Å | $\alpha$= 90°. |
| | b = 5.3917(8) Å | $\beta$= 104.529(8)°. |
| | c = 12.861(2) Å | $\gamma$ = 90°. |
| Volume | 789.2(2) $Å^3$ | |
| Z | 2 | |
| Density (calculated) | 1.323 $Mg/m^3$ | |
| Absorption coefficient | 0.092 $mm^{-1}$ | |
| F(000) | 336 | |
| Crystal size | 0.500 x 0.100 x 0.050 $mm^3$ | |
| Theta range for data collection | 1.636 to 22.464°. | |
| Index ranges | $-12 \leq h \leq 11, -5 \leq k \leq 5, -13 \leq l \leq 13$ | |
| Reflections collected | 8970 | |
| Independent reflections | 2037 [R(int) = 0.0848] | |
| Completeness to theta = 22.464° | 100.0 % | |
| Absorption correction | Semi-empirical from equivalents | |
| Refinement method | Full-matrix least-squares on $F^2$ | |
| Data / restraints / parameters | 2037 / 2 / 213 | |
| Goodness-of-fit on $F^2$ | 1.016 | |
| Final R indices [I>2sigma(I)] | R1 = 0.0505, wR2 = 0.1037 | |
| R indices (all data) | R1 = 0.0898, wR2 = 0.1180 | |
| Absolute structure parameter | 0.4(10) | |
| Extinction coefficient | n/a | |
| Largest diff. peak and hole | 0.181 and -0.210 e.$Å^{-3}$ | |

*Table 3. Atomic coordinates (x $10^4$) and equivalent isotropic displacement parameters ($Å^2$x $10^3$) for Diene **28**. U(eq) is defined as one third of the trace of the orthogonalized $U^{ij}$ tensor.*

| | x | y | z | U(eq) |
|---|---|---|---|---|
| O(1) | 3461(4) | 5365(8) | 3878(3) | 40(1) |
| C(1) | 4556(7) | 4881(12) | 3816(5) | 36(2) |
| O(2) | 5025(4) | 2972(8) | 4167(3) | 49(1) |
| C(2) | 5051(5) | 7063(10) | 3307(5) | 29(2) |
| C(3) | 6016(6) | 6448(10) | 2746(5) | 29(2) |
| C(4) | 7038(6) | 5073(14) | 3464(5) | 46(2) |
| C(5) | 7733(5) | 3556(10) | 2834(5) | 28(2) |
| C(16) | 8739(6) | 2141(10) | 3541(5) | 32(2) |
| C(17) | 8632(7) | 1281(13) | 4544(6) | 56(2) |
| C(18) | 9737(6) | 1587(14) | 3197(6) | 62(2) |
| C(6) | 8111(6) | 5325(13) | 2068(6) | 44(2) |
| C(7) | 7114(6) | 6803(11) | 1381(5) | 32(2) |
| O(3) | 7222(4) | 7704(8) | 541(4) | 44(1) |
| C(8) | 6042(5) | 7252(10) | 1764(5) | 27(2) |
| C(9) | 5096(5) | 8726(11) | 995(5) | 32(2) |
| C(10) | 3886(5) | 7674(10) | 819(5) | 31(2) |
| C(11) | 3363(5) | 7471(10) | 1604(5) | 26(2) |
| C(12) | 2148(6) | 6428(10) | 1542(5) | 32(2) |
| O(4) | 2230(4) | 3726(8) | 1651(3) | 36(1) |
| C(19) | 1219(5) | 7101(11) | 538(5) | 40(2) |
| C(13) | 1888(6) | 7406(13) | 2568(5) | 41(2) |
| C(14) | 3051(6) | 7757(11) | 3367(5) | 35(2) |
| C(15) | 3927(5) | 8399(10) | 2713(5) | 27(2) |

Table 4. Bond lengths [Å] and angles [°] for Diene **28**.

| | |
|---|---|
| O(1)-C(1) | 1.336(8) |
| O(1)-C(14) | 1.473(7) |
| C(1)-O(2) | 1.201(8) |
| C(1)-C(2) | 1.530(8) |
| C(2)-C(3) | 1.526(8) |
| C(2)-C(15) | 1.531(8) |
| C(2)-H(2) | 1.0000 |
| C(3)-C(8) | 1.342(8) |
| C(3)-C(4) | 1.513(9) |
| C(4)-C(5) | 1.525(8) |
| C(4)-H(4A) | 0.9900 |
| C(4)-H(4B) | 0.9900 |
| C(5)-C(16) | 1.506(8) |
| C(5)-C(6) | 1.516(8) |
| C(5)-H(5) | 1.0000 |
| C(16)-C(18) | 1.387(8) |
| C(16)-C(17) | 1.406(8) |
| C(17)-H(17A) | 0.9800 |
| C(17)-H(17B) | 0.9800 |
| C(17)-H(17C) | 0.9800 |
| C(18)-H(18A) | 0.9500 |
| C(18)-H(18B) | 0.9500 |
| C(6)-C(7) | 1.506(9) |
| C(6)-H(6A) | 0.9900 |
| C(6)-H(6B) | 0.9900 |
| C(7)-O(3) | 1.221(7) |
| C(7)-C(8) | 1.484(8) |
| C(8)-C(9) | 1.515(8) |
| C(9)-C(10) | 1.495(8) |
| C(9)-H(9A) | 0.9900 |
| C(9)-H(9B) | 0.9900 |
| C(10)-C(11) | 1.311(7) |
| C(10)-H(10) | 0.9500 |

*Table 4. (cont'd)*

| | |
|---|---|
| C(11)-C(15) | 1.499(8) |
| C(11)-C(12) | 1.519(8) |
| C(12)-O(4) | 1.465(7) |
| C(12)-C(19) | 1.512(8) |
| C(12)-C(13) | 1.522(8) |
| O(4)-H(4O) | 0.83(3) |
| C(19)-H(19A) | 0.9800 |
| C(19)-H(19B) | 0.9800 |
| C(19)-H(19C) | 0.9800 |
| C(13)-C(14) | 1.502(8) |
| C(13)-H(13A) | 0.9900 |
| C(13)-H(13B) | 0.9900 |
| C(14)-C(15) | 1.524(8) |
| C(14)-H(14) | 1.0000 |
| C(15)-H(15) | 1.0000 |

| | |
|---|---|
| C(1)-O(1)-C(14) | 110.9(5) |
| O(2)-C(1)-O(1) | 120.2(6) |
| O(2)-C(1)-C(2) | 129.2(7) |
| O(1)-C(1)-C(2) | 110.5(6) |
| C(3)-C(2)-C(1) | 116.3(5) |
| C(3)-C(2)-C(15) | 121.1(5) |
| C(1)-C(2)-C(15) | 101.7(5) |
| C(3)-C(2)-H(2) | 105.5 |
| C(1)-C(2)-H(2) | 105.5 |
| C(15)-C(2)-H(2) | 105.5 |
| C(8)-C(3)-C(4) | 122.1(6) |
| C(8)-C(3)-C(2) | 124.7(5) |
| C(4)-C(3)-C(2) | 112.8(5) |
| C(3)-C(4)-C(5) | 112.8(5) |
| C(3)-C(4)-H(4A) | 109.0 |
| C(5)-C(4)-H(4A) | 109.0 |
| C(3)-C(4)-H(4B) | 109.0 |
| C(5)-C(4)-H(4B) | 109.0 |

*Table 4. (cont'd)*

| | |
|---|---|
| H(4A)-C(4)-H(4B) | 107.8 |
| C(16)-C(5)-C(6) | 113.4(5) |
| C(16)-C(5)-C(4) | 113.3(5) |
| C(6)-C(5)-C(4) | 107.0(5) |
| C(16)-C(5)-H(5) | 107.6 |
| C(6)-C(5)-H(5) | 107.6 |
| C(4)-C(5)-H(5) | 107.6 |
| C(18)-C(16)-C(17) | 120.0(6) |
| C(18)-C(16)-C(5) | 121.1(6) |
| C(17)-C(16)-C(5) | 118.9(6) |
| C(16)-C(17)-H(17A) | 109.5 |
| C(16)-C(17)-H(17B) | 109.5 |
| H(17A)-C(17)-H(17B) | 109.5 |
| C(16)-C(17)-H(17C) | 109.5 |
| H(17A)-C(17)-H(17C) | 109.5 |
| H(17B)-C(17)-H(17C) | 109.5 |
| C(16)-C(18)-H(18A) | 120.0 |
| C(16)-C(18)-H(18B) | 120.0 |
| H(18A)-C(18)-H(18B) | 120.0 |
| C(7)-C(6)-C(5) | 113.7(5) |
| C(7)-C(6)-H(6A) | 108.8 |
| C(5)-C(6)-H(6A) | 108.8 |
| C(7)-C(6)-H(6B) | 108.8 |
| C(5)-C(6)-H(6B) | 108.8 |
| H(6A)-C(6)-H(6B) | 107.7 |
| O(3)-C(7)-C(8) | 120.9(6) |
| O(3)-C(7)-C(6) | 119.5(6) |
| C(8)-C(7)-C(6) | 119.5(6) |
| C(3)-C(8)-C(7) | 118.7(5) |
| C(3)-C(8)-C(9) | 126.7(5) |
| C(7)-C(8)-C(9) | 114.5(5) |
| C(10)-C(9)-C(8) | 114.5(5) |
| C(10)-C(9)-H(9A) | 108.6 |
| C(8)-C(9)-H(9A) | 108.6 |

*Table 4. (cont'd)*

| | |
|---|---|
| C(10)-C(9)-H(9B) | 108.6 |
| C(8)-C(9)-H(9B) | 108.6 |
| H(9A)-C(9)-H(9B) | 107.6 |
| C(11)-C(10)-C(9) | 121.8(6) |
| C(11)-C(10)-H(10) | 119.1 |
| C(9)-C(10)-H(10) | 119.1 |
| C(10)-C(11)-C(15) | 121.6(5) |
| C(10)-C(11)-C(12) | 127.4(5) |
| C(15)-C(11)-C(12) | 111.0(5) |
| O(4)-C(12)-C(19) | 109.7(5) |
| O(4)-C(12)-C(11) | 109.0(5) |
| C(19)-C(12)-C(11) | 115.1(5) |
| O(4)-C(12)-C(13) | 106.5(5) |
| C(19)-C(12)-C(13) | 113.5(5) |
| C(11)-C(12)-C(13) | 102.6(5) |
| C(12)-O(4)-H(4O) | 106(5) |
| C(12)-C(19)-H(19A) | 109.5 |
| C(12)-C(19)-H(19B) | 109.5 |
| H(19A)-C(19)-H(19B) | 109.5 |
| C(12)-C(19)-H(19C) | 109.5 |
| H(19A)-C(19)-H(19C) | 109.5 |
| H(19B)-C(19)-H(19C) | 109.5 |
| C(14)-C(13)-C(12) | 106.9(5) |
| C(14)-C(13)-H(13A) | 110.4 |
| C(12)-C(13)-H(13A) | 110.4 |
| C(14)-C(13)-H(13B) | 110.4 |
| C(12)-C(13)-H(13B) | 110.4 |
| H(13A)-C(13)-H(13B) | 108.6 |
| O(1)-C(14)-C(13) | 109.5(5) |
| O(1)-C(14)-C(15) | 104.8(5) |
| C(13)-C(14)-C(15) | 106.0(5) |
| O(1)-C(14)-H(14) | 112.0 |
| C(13)-C(14)-H(14) | 112.0 |
| C(15)-C(14)-H(14) | 112.0 |

*Table 4. (cont'd)*

| | |
|---|---|
| C(11)-C(15)-C(14) | 104.5(5) |
| C(11)-C(15)-C(2) | 116.0(5) |
| C(14)-C(15)-C(2) | 103.6(5) |
| C(11)-C(15)-H(15) | 110.8 |
| C(14)-C(15)-H(15) | 110.8 |
| C(2)-C(15)-H(15) | 110.8 |

Symmetry transformations used to generate equivalent atoms:

*Table 5. Anisotropic displacement parameters ($Å^2x10^3$) for Diene **28**. The anisotropic displacement factor exponent takes the form: $-2\pi^2[ h^2a^{*2}U^{11} + ... + 2hka^*b^*U^{12}]$.*

| | $U^{11}$ | $U^{22}$ | $U^{33}$ | $U^{23}$ | $U^{13}$ | $U^{12}$ |
|---|---|---|---|---|---|---|
| O(1) | 53(4) | 37(3) | 32(3) | -5(2) | 14(3) | -10(3) |
| C(1) | 74(6) | 13(4) | 16(4) | -6(3) | 5(4) | -11(4) |
| O(2) | 91(4) | 20(2) | 29(3) | -2(2) | 3(3) | -6(3) |
| C(2) | 43(4) | 23(3) | 20(3) | -3(3) | 5(3) | -1(3) |
| C(3) | 38(5) | 22(3) | 29(4) | 0(3) | 12(4) | 3(3) |
| C(4) | 54(5) | 54(4) | 32(4) | 3(4) | 10(4) | 22(4) |
| C(5) | 40(4) | 11(3) | 34(4) | -6(3) | 9(4) | -4(3) |
| C(16) | 43(5) | 22(3) | 30(4) | -2(3) | 10(4) | -6(3) |
| C(17) | 61(6) | 46(4) | 55(5) | -1(4) | 5(4) | 12(4) |
| C(18) | 53(5) | 70(5) | 69(6) | 30(5) | 26(5) | 32(5) |
| C(6) | 40(5) | 47(4) | 48(4) | 23(4) | 14(4) | 12(4) |
| C(7) | 44(5) | 17(3) | 32(4) | -4(3) | 7(4) | -6(3) |
| O(3) | 50(3) | 37(2) | 48(3) | 18(2) | 20(3) | 4(2) |
| C(8) | 35(4) | 16(3) | 28(4) | -3(3) | 5(3) | -1(3) |
| C(9) | 42(5) | 22(3) | 30(4) | 3(3) | 9(4) | -3(3) |
| C(10) | 37(4) | 21(3) | 33(4) | -6(3) | 7(3) | -2(3) |
| C(11) | 30(4) | 17(3) | 30(4) | 3(3) | 6(3) | 2(3) |
| C(12) | 42(5) | 23(3) | 32(4) | -4(3) | 9(4) | -1(3) |
| O(4) | 48(3) | 23(2) | 39(3) | -6(2) | 14(3) | -6(2) |
| C(19) | 40(4) | 29(4) | 48(5) | -3(3) | 8(4) | -2(3) |
| C(13) | 53(5) | 35(4) | 39(4) | -10(3) | 18(4) | -4(4) |
| C(14) | 49(5) | 24(3) | 35(4) | -7(3) | 16(4) | 2(3) |
| C(15) | 33(4) | 20(3) | 25(4) | -6(3) | 0(3) | -1(3) |

*Table 6. Hydrogen coordinates (x10[4]) and isotropic displacement parameters (Å[2]x10[3]) for Diene **28**.*

| | x | y | z | U(eq) |
|---|---|---|---|---|
| H(2) | 5428 | 8178 | 3918 | 35 |
| H(4A) | 6736 | 3951 | 3943 | 56 |
| H(4B) | 7570 | 6285 | 3921 | 56 |
| H(5) | 7183 | 2320 | 2392 | 34 |
| H(17A) | 9335 | 334 | 4894 | 84 |
| H(17B) | 7938 | 212 | 4441 | 84 |
| H(17C) | 8547 | 2697 | 4996 | 84 |
| H(18A) | 10348 | 627 | 3639 | 74 |
| H(18B) | 9810 | 2168 | 2519 | 74 |
| H(6A) | 8703 | 6486 | 2489 | 53 |
| H(6B) | 8493 | 4362 | 1594 | 53 |
| H(9A) | 5094 | 10436 | 1275 | 38 |
| H(9B) | 5297 | 8828 | 294 | 38 |
| H(10) | 3484 | 7139 | 119 | 37 |
| H(4O) | 2450(60) | 3220(130) | 1120(40) | 54 |
| H(19A) | 1426 | 6385 | -90 | 60 |
| H(19B) | 1170 | 8910 | 465 | 60 |
| H(19C) | 457 | 6445 | 589 | 60 |
| H(13A) | 1460 | 9002 | 2429 | 49 |
| H(13B) | 1398 | 6206 | 2846 | 49 |
| H(14) | 3016 | 9069 | 3907 | 42 |
| H(15) | 4056 | 10232 | 2706 | 33 |

*Table 7. Torsion angles [°] for Diene* ***28***.

| | |
|---|---|
| C(14)-O(1)-C(1)-O(2) | -178.0(5) |
| C(14)-O(1)-C(1)-C(2) | 4.1(6) |
| O(2)-C(1)-C(2)-C(3) | 28.0(9) |
| O(1)-C(1)-C(2)-C(3) | -154.4(5) |
| O(2)-C(1)-C(2)-C(15) | 161.6(6) |
| O(1)-C(1)-C(2)-C(15) | -20.8(6) |
| C(1)-C(2)-C(3)-C(8) | 132.6(6) |
| C(15)-C(2)-C(3)-C(8) | 8.5(8) |
| C(1)-C(2)-C(3)-C(4) | -54.4(7) |
| C(15)-C(2)-C(3)-C(4) | -178.6(5) |
| C(8)-C(3)-C(4)-C(5) | -30.4(9) |
| C(2)-C(3)-C(4)-C(5) | 156.4(5) |
| C(3)-C(4)-C(5)-C(16) | -178.9(5) |
| C(3)-C(4)-C(5)-C(6) | 55.4(7) |
| C(6)-C(5)-C(16)-C(18) | -29.2(8) |
| C(4)-C(5)-C(16)-C(18) | -151.3(6) |
| C(6)-C(5)-C(16)-C(17) | 153.7(6) |
| C(4)-C(5)-C(16)-C(17) | 31.6(8) |
| C(16)-C(5)-C(6)-C(7) | -178.3(5) |
| C(4)-C(5)-C(6)-C(7) | -52.7(7) |
| C(5)-C(6)-C(7)-O(3) | -158.3(6) |
| C(5)-C(6)-C(7)-C(8) | 24.8(8) |
| C(4)-C(3)-C(8)-C(7) | -0.8(8) |
| C(2)-C(3)-C(8)-C(7) | 171.5(5) |
| C(4)-C(3)-C(8)-C(9) | -177.5(6) |
| C(2)-C(3)-C(8)-C(9) | -5.2(9) |
| O(3)-C(7)-C(8)-C(3) | -172.9(5) |
| C(6)-C(7)-C(8)-C(3) | 3.9(8) |
| O(3)-C(7)-C(8)-C(9) | 4.1(8) |
| C(6)-C(7)-C(8)-C(9) | -179.1(5) |
| C(3)-C(8)-C(9)-C(10) | -49.6(8) |
| C(7)-C(8)-C(9)-C(10) | 133.6(5) |
| C(8)-C(9)-C(10)-C(11) | 62.6(8) |
| C(9)-C(10)-C(11)-C(15) | 3.1(9) |

*Table 7. (cont'd)*

| | |
|---|---|
| C(9)-C(10)-C(11)-C(12) | -179.5(5) |
| C(10)-C(11)-C(12)-O(4) | 84.5(7) |
| C(15)-C(11)-C(12)-O(4) | -98.0(5) |
| C(10)-C(11)-C(12)-C(19) | -39.2(8) |
| C(15)-C(11)-C(12)-C(19) | 138.4(5) |
| C(10)-C(11)-C(12)-C(13) | -162.9(6) |
| C(15)-C(11)-C(12)-C(13) | 14.7(6) |
| O(4)-C(12)-C(13)-C(14) | 86.9(6) |
| C(19)-C(12)-C(13)-C(14) | -152.3(5) |
| C(11)-C(12)-C(13)-C(14) | -27.5(6) |
| C(1)-O(1)-C(14)-C(13) | 127.9(5) |
| C(1)-O(1)-C(14)-C(15) | 14.6(6) |
| C(12)-C(13)-C(14)-O(1) | -81.9(6) |
| C(12)-C(13)-C(14)-C(15) | 30.7(6) |
| C(10)-C(11)-C(15)-C(14) | -178.9(5) |
| C(12)-C(11)-C(15)-C(14) | 3.4(6) |
| C(10)-C(11)-C(15)-C(2) | -65.5(7) |
| C(12)-C(11)-C(15)-C(2) | 116.8(5) |
| O(1)-C(14)-C(15)-C(11) | 95.2(5) |
| C(13)-C(14)-C(15)-C(11) | -20.6(6) |
| O(1)-C(14)-C(15)-C(2) | -26.7(5) |
| C(13)-C(14)-C(15)-C(2) | -142.5(5) |
| C(3)-C(2)-C(15)-C(11) | 44.8(7) |
| C(1)-C(2)-C(15)-C(11) | -85.9(6) |
| C(3)-C(2)-C(15)-C(14) | 158.7(5) |
| C(1)-C(2)-C(15)-C(14) | 28.0(5) |

Symmetry transformations used to generate equivalent atoms:

*Table 8. Hydrogen bonds for Diene* ***28*** *[Å and °].*

| D-H...A | d(D-H) | d(H...A) | d(D...A) | <(DHA) |
|---|---|---|---|---|
| C(2)-H(2)...O(2)#1 | 1.00 | 2.65 | 3.309(7) | 123.5 |
| O(4)-H(4O)...O(3)#2 | 0.83(3) | 2.28(3) | 3.094(6) | 165(7) |
| C(15)-H(15)...O(2)#3 | 1.00 | 2.44 | 3.167(7) | 129.5 |

Symmetry transformations used to generate equivalent atoms:

#1 -x+1,y+1/2,-z+1 #2 -x+1,y-1/2,-z #3 x,y+1,z

## X-Ray Crystal Structure Analysis of *ent*-Isoineleganolide A (29)

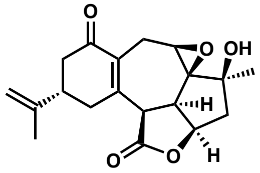


***ent-Isoineleganolide A***
***29***

Contents

Table 1. Experimental Details

Table 2. Crystal Data

Table 3. Atomic Coordinates

Table 4. Full Bond Distances and Angles

Table 5. Anisotropic Displacement Parameters

Table 6. Hydrogen Atomic Coordinates

Table 7. Torsion Angles

Table 8. Hydrogen Bond Distances and Angles

*X-Ray Crystal Structure of ent-Isoineleganolide A (**29**)*

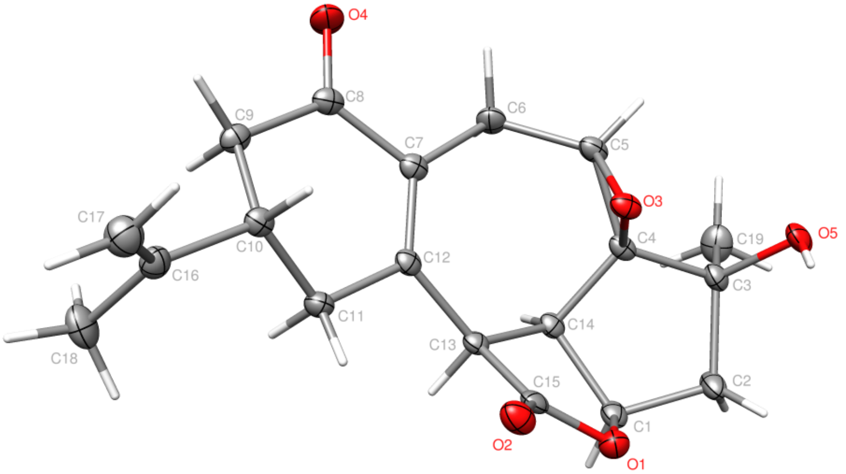


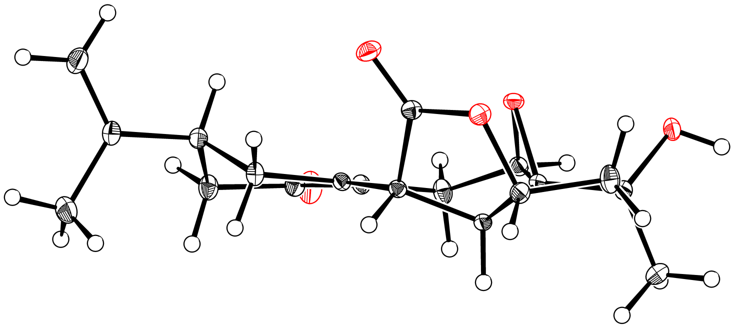

*Table 1. Experimental Details for X-Ray Structure Determination of ent-Isoineleganolide A (**29**).*

Low-temperature diffraction data ($\phi$-and $\omega$-scans) were collected on a Bruker AXS KAPPA APEX II diffractometer coupled to a APEX II CCD detector with graphite monochromated Mo $K_\alpha$ radiation ($\lambda$ = 0.71073 Å) for the structure of *ent*-isoineleganolide A (**29**). The structure was solved by direct methods using SHELXS[i] and refined against $F^2$ on all data by full-matrix least squares with SHELXL-2014[ii] using established refinement techniques.[iii] All non-hydrogen atoms were refined anisotropically. Unless otherwise noted, all hydrogen atoms were included into the model at geometrically calculated positions and refined using a riding model. The isotropic displacement parameters of all hydrogen atoms were fixed to 1.2 times the $U$ value of the atoms they are linked to (1.5 times for methyl groups).

*ent*-Isoineleganolide A (**29**) crystallizes in the monoclinic space group $P2_1$ with one molecule in the asymmetric unit. The coordinates for the hydrogen atom bound to O4 was located in the difference Fourier synthesis and refined semi-freely with the help of a restraint on the O-H distance (0.84(4) Å).

*Table 2. Crystal Data and Structure Refinement for ent-Isoineleganolide A (**29**).*

| | | |
|---|---|---|
| Caltech Identification code | rac03 | |
| CCDC Deposition Number | 853379 / 1061011 | |
| Empirical formula | C19 H22 O5 | |
| Formula weight | 330.36 | |
| Temperature | 100(2) K | |
| Wavelength | 0.71073 Å | |
| Crystal system | Monoclinic | |
| Space group | $P2_1$ | |
| Unit cell dimensions | a = 6.9222(2) Å | α= 90°. |
| | b = 11.1470(4) Å | β= 94.070(2)°. |
| | c = 10.4409(4) Å | γ = 90°. |
| Volume | 803.61(5) $Å^3$ | |
| Z | 2 | |
| Density (calculated) | 1.365 $Mg/m^3$ | |
| Absorption coefficient | 0.098 $mm^{-1}$ | |
| F(000) | 352 | |
| Crystal size | 0.500 x 0.450 x 0.200 $mm^3$ | |
| Theta range for data collection | 1.955 to 36.317°. | |
| Index ranges | –11 ≤ h ≤ 11, –18 ≤ k ≤ 18, –17 ≤ l ≤ 17 | |
| Reflections collected | 30117 | |
| Independent reflections | 7796 [R(int) = 0.0404] | |
| Completeness to theta = 25.242° | 100.0 % | |
| Absorption correction | Semi-empirical from equivalents | |
| Max. and min. transmission | 0.7475 and 0.6239 | |
| Refinement method | Full-matrix least-squares on $F^2$ | |
| Data / restraints / parameters | 7796 / 2 / 222 | |
| Goodness-of-fit on $F^2$ | 1.067 | |
| Final R indices [I>2sigma(I)] | R1 = 0.0445, wR2 = 0.1109 | |
| R indices (all data) | R1 = 0.0538, wR2 = 0.1198 | |
| Absolute structure parameter | -0.5(3) | |
| Extinction coefficient | n/a | |
| Largest diff. peak and hole | 0.732 and -0.318 e.$Å^{-3}$ | |

*Table 3. Atomic coordinates (x $10^4$) and equivalent isotropic displacement parameters (Å$^2$x $10^3$) for ent-Isoineleganolide A (**29**). U(eq) is defined as one third of the trace of the orthogonalized $U^{ij}$ tensor.*

| | x | y | z | U(eq) |
|---|---|---|---|---|
| O(1) | 6780(2) | 3526(1) | 2529(1) | 17(1) |
| C(1) | 6361(2) | 3631(1) | 3770(1) | 13(1) |
| O(2) | 6200(2) | 2761(1) | 4432(1) | 18(1) |
| C(2) | 6086(2) | 4958(1) | 4084(1) | 11(1) |
| C(3) | 6324(2) | 5269(1) | 5490(1) | 11(1) |
| C(4) | 4753(2) | 4700(1) | 6220(1) | 14(1) |
| C(5) | 5248(2) | 4650(1) | 7666(1) | 12(1) |
| C(16) | 3659(2) | 4140(1) | 8421(1) | 15(1) |
| C(17) | 1649(2) | 4643(2) | 8156(2) | 23(1) |
| C(18) | 4057(3) | 3309(2) | 9322(2) | 21(1) |
| C(6) | 5752(2) | 5923(1) | 8097(1) | 14(1) |
| C(7) | 7361(2) | 6450(1) | 7385(1) | 14(1) |
| O(3) | 8395(2) | 7238(1) | 7898(1) | 22(1) |
| C(8) | 7629(2) | 6048(1) | 6053(1) | 12(1) |
| C(9) | 9282(2) | 6681(1) | 5467(2) | 16(1) |
| C(10) | 10375(2) | 6022(1) | 4484(1) | 13(1) |
| O(5) | 10127(1) | 4727(1) | 4366(1) | 13(1) |
| C(11) | 9411(2) | 5510(1) | 3334(1) | 11(1) |
| C(12) | 10337(2) | 5381(1) | 2046(1) | 13(1) |
| O(4) | 12121(2) | 4754(1) | 2164(1) | 18(1) |
| C(19) | 10782(3) | 6615(2) | 1521(2) | 25(1) |
| C(13) | 8747(2) | 4724(2) | 1184(1) | 18(1) |
| C(14) | 6917(2) | 4701(2) | 1921(1) | 14(1) |
| C(15) | 7241(2) | 5555(1) | 3068(1) | 12(1) |

*Table 4. Bond lengths [Å] and angles [°] for ent-Isoineleganolide A (**29**).*

| | |
|---|---|
| O(1)-C(1) | 1.3531(18) |
| O(1)-C(14) | 1.462(2) |
| C(1)-O(2) | 1.2007(19) |
| C(1)-C(2) | 1.5291(19) |
| C(2)-C(3) | 1.5070(18) |
| C(2)-C(15) | 1.5255(19) |
| C(2)-H(2) | 1.0000 |
| C(3)-C(8) | 1.3565(18) |
| C(3)-C(4) | 1.5109(19) |
| C(4)-C(5) | 1.5248(19) |
| C(4)-H(4A) | 0.9900 |
| C(4)-H(4B) | 0.9900 |
| C(5)-C(16) | 1.509(2) |
| C(5)-C(6) | 1.522(2) |
| C(5)-H(5) | 1.0000 |
| C(16)-C(18) | 1.335(2) |
| C(16)-C(17) | 1.508(2) |
| C(17)-H(17A) | 0.9800 |
| C(17)-H(17B) | 0.9800 |
| C(17)-H(17C) | 0.9800 |
| C(18)-H(18A) | 0.9500 |
| C(18)-H(18B) | 0.9500 |
| C(6)-C(7) | 1.502(2) |
| C(6)-H(6A) | 0.9900 |
| C(6)-H(6B) | 0.9900 |
| C(7)-O(3) | 1.2318(18) |
| C(7)-C(8) | 1.485(2) |
| C(8)-C(9) | 1.511(2) |
| C(9)-C(10) | 1.508(2) |
| C(9)-H(9A) | 0.9900 |
| C(9)-H(9B) | 0.9900 |
| C(10)-C(11) | 1.4490(19) |
| C(10)-O(5) | 1.4582(18) |

*Table 4. (cont'd)*

| | |
|---|---|
| C(10)-H(10) | 1.0000 |
| O(5)-C(11) | 1.4458(17) |
| C(11)-C(15) | 1.5093(19) |
| C(11)-C(12) | 1.5377(19) |
| C(12)-O(4) | 1.4163(18) |
| C(12)-C(19) | 1.521(2) |
| C(12)-C(13) | 1.555(2) |
| O(4)-H(4O) | 0.83(2) |
| C(19)-H(19A) | 0.9800 |
| C(19)-H(19B) | 0.9800 |
| C(19)-H(19C) | 0.9800 |
| C(13)-C(14) | 1.528(2) |
| C(13)-H(13A) | 0.9900 |
| C(13)-H(13B) | 0.9900 |
| C(14)-C(15) | 1.534(2) |
| C(14)-H(14) | 1.0000 |
| C(15)-H(15) | 1.0000 |
| | |
| C(1)-O(1)-C(14) | 111.28(11) |
| O(2)-C(1)-O(1) | 121.05(13) |
| O(2)-C(1)-C(2) | 129.80(13) |
| O(1)-C(1)-C(2) | 109.11(12) |
| C(3)-C(2)-C(15) | 123.39(12) |
| C(3)-C(2)-C(1) | 115.12(11) |
| C(15)-C(2)-C(1) | 101.21(11) |
| C(3)-C(2)-H(2) | 105.2 |
| C(15)-C(2)-H(2) | 105.2 |
| C(1)-C(2)-H(2) | 105.2 |
| C(8)-C(3)-C(2) | 126.34(12) |
| C(8)-C(3)-C(4) | 122.11(12) |
| C(2)-C(3)-C(4) | 111.24(11) |
| C(3)-C(4)-C(5) | 113.28(11) |
| C(3)-C(4)-H(4A) | 108.9 |
| C(5)-C(4)-H(4A) | 108.9 |

Table 4. (cont'd)

| | |
|---|---|
| C(3)-C(4)-H(4B) | 108.9 |
| C(5)-C(4)-H(4B) | 108.9 |
| H(4A)-C(4)-H(4B) | 107.7 |
| C(16)-C(5)-C(6) | 110.93(12) |
| C(16)-C(5)-C(4) | 114.17(11) |
| C(6)-C(5)-C(4) | 106.86(11) |
| C(16)-C(5)-H(5) | 108.2 |
| C(6)-C(5)-H(5) | 108.2 |
| C(4)-C(5)-H(5) | 108.2 |
| C(18)-C(16)-C(17) | 121.95(15) |
| C(18)-C(16)-C(5) | 120.53(14) |
| C(17)-C(16)-C(5) | 117.47(13) |
| C(16)-C(17)-H(17A) | 109.5 |
| C(16)-C(17)-H(17B) | 109.5 |
| H(17A)-C(17)-H(17B) | 109.5 |
| C(16)-C(17)-H(17C) | 109.5 |
| H(17A)-C(17)-H(17C) | 109.5 |
| H(17B)-C(17)-H(17C) | 109.5 |
| C(16)-C(18)-H(18A) | 120.0 |
| C(16)-C(18)-H(18B) | 120.0 |
| H(18A)-C(18)-H(18B) | 120.0 |
| C(7)-C(6)-C(5) | 112.36(12) |
| C(7)-C(6)-H(6A) | 109.1 |
| C(5)-C(6)-H(6A) | 109.1 |
| C(7)-C(6)-H(6B) | 109.1 |
| C(5)-C(6)-H(6B) | 109.1 |
| H(6A)-C(6)-H(6B) | 107.9 |
| O(3)-C(7)-C(8) | 121.04(14) |
| O(3)-C(7)-C(6) | 119.45(13) |
| C(8)-C(7)-C(6) | 119.46(12) |
| C(3)-C(8)-C(7) | 118.36(12) |
| C(3)-C(8)-C(9) | 128.65(13) |
| C(7)-C(8)-C(9) | 112.73(12) |
| C(10)-C(9)-C(8) | 118.59(12) |

*Table 4. (cont'd)*

| | |
|---|---|
| C(10)-C(9)-H(9A) | 107.7 |
| C(8)-C(9)-H(9A) | 107.7 |
| C(10)-C(9)-H(9B) | 107.7 |
| C(8)-C(9)-H(9B) | 107.7 |
| H(9A)-C(9)-H(9B) | 107.1 |
| C(11)-C(10)-O(5) | 59.64(9) |
| C(11)-C(10)-C(9) | 122.14(12) |
| O(5)-C(10)-C(9) | 118.60(12) |
| C(11)-C(10)-H(10) | 115.1 |
| O(5)-C(10)-H(10) | 115.1 |
| C(9)-C(10)-H(10) | 115.1 |
| C(11)-O(5)-C(10) | 59.86(9) |
| O(5)-C(11)-C(10) | 60.50(9) |
| O(5)-C(11)-C(15) | 116.21(11) |
| C(10)-C(11)-C(15) | 122.21(12) |
| O(5)-C(11)-C(12) | 116.94(11) |
| C(10)-C(11)-C(12) | 124.47(12) |
| C(15)-C(11)-C(12) | 108.27(11) |
| O(4)-C(12)-C(19) | 106.25(13) |
| O(4)-C(12)-C(11) | 112.63(11) |
| C(19)-C(12)-C(11) | 109.80(13) |
| O(4)-C(12)-C(13) | 113.30(13) |
| C(19)-C(12)-C(13) | 111.77(13) |
| C(11)-C(12)-C(13) | 103.20(11) |
| C(12)-O(4)-H(4O) | 108(2) |
| C(12)-C(19)-H(19A) | 109.5 |
| C(12)-C(19)-H(19B) | 109.5 |
| H(19A)-C(19)-H(19B) | 109.5 |
| C(12)-C(19)-H(19C) | 109.5 |
| H(19A)-C(19)-H(19C) | 109.5 |
| H(19B)-C(19)-H(19C) | 109.5 |
| C(14)-C(13)-C(12) | 107.14(12) |
| C(14)-C(13)-H(13A) | 110.3 |
| C(12)-C(13)-H(13A) | 110.3 |

*Table 4. (cont'd)*

| | |
|---|---|
| C(14)-C(13)-H(13B) | 110.3 |
| C(12)-C(13)-H(13B) | 110.3 |
| H(13A)-C(13)-H(13B) | 108.5 |
| O(1)-C(14)-C(13) | 108.36(13) |
| O(1)-C(14)-C(15) | 103.16(10) |
| C(13)-C(14)-C(15) | 107.59(12) |
| O(1)-C(14)-H(14) | 112.4 |
| C(13)-C(14)-H(14) | 112.4 |
| C(15)-C(14)-H(14) | 112.4 |
| C(11)-C(15)-C(2) | 114.92(11) |
| C(11)-C(15)-C(14) | 102.19(11) |
| C(2)-C(15)-C(14) | 102.50(11) |
| C(11)-C(15)-H(15) | 112.1 |
| C(2)-C(15)-H(15) | 112.1 |
| C(14)-C(15)-H(15) | 112.1 |

Symmetry transformations used to generate equivalent atoms:

*Table 5. Anisotropic displacement parameters ($Å^2x10^3$) for ent-Isoineleganolide A (**29**). The anisotropic displacement factor exponent takes the form: $-2\pi^2[ h^2a^{*2}U^{11} + ... + 2hka^*b^*U^{12}]$.*

| | $U^{11}$ | $U^{22}$ | $U^{33}$ | $U^{23}$ | $U^{13}$ | $U^{12}$ |
|---|---|---|---|---|---|---|
| O(1) | 17(1) | 16(1) | 18(1) | -5(1) | 3(1) | -2(1) |
| C(1) | 8(1) | 13(1) | 17(1) | -3(1) | 0(1) | -2(1) |
| O(2) | 16(1) | 11(1) | 26(1) | 0(1) | 2(1) | -1(1) |
| C(2) | 7(1) | 12(1) | 13(1) | -1(1) | 0(1) | 0(1) |
| C(3) | 9(1) | 10(1) | 13(1) | -1(1) | 0(1) | 1(1) |
| C(4) | 12(1) | 16(1) | 14(1) | -1(1) | 1(1) | -4(1) |
| C(5) | 12(1) | 10(1) | 14(1) | 0(1) | 1(1) | 1(1) |
| C(16) | 16(1) | 13(1) | 16(1) | 0(1) | 3(1) | 0(1) |
| C(17) | 15(1) | 23(1) | 31(1) | 4(1) | 8(1) | 3(1) |
| C(18) | 24(1) | 18(1) | 20(1) | 4(1) | 3(1) | -2(1) |
| C(6) | 16(1) | 11(1) | 15(1) | -1(1) | 1(1) | 1(1) |
| C(7) | 13(1) | 12(1) | 17(1) | -3(1) | -1(1) | 0(1) |
| O(3) | 22(1) | 19(1) | 24(1) | -9(1) | 3(1) | -7(1) |
| C(8) | 11(1) | 9(1) | 15(1) | -1(1) | 1(1) | 0(1) |
| C(9) | 15(1) | 12(1) | 20(1) | -3(1) | 4(1) | -5(1) |
| C(10) | 9(1) | 11(1) | 20(1) | -1(1) | 0(1) | -1(1) |
| O(5) | 11(1) | 10(1) | 18(1) | 1(1) | -2(1) | 1(1) |
| C(11) | 8(1) | 10(1) | 16(1) | 1(1) | 0(1) | 0(1) |
| C(12) | 9(1) | 14(1) | 17(1) | 1(1) | 2(1) | 1(1) |
| O(4) | 8(1) | 19(1) | 25(1) | -3(1) | 1(1) | 2(1) |
| C(19) | 30(1) | 17(1) | 29(1) | 5(1) | 10(1) | -1(1) |
| C(13) | 10(1) | 28(1) | 15(1) | -2(1) | 1(1) | 1(1) |
| C(14) | 9(1) | 20(1) | 14(1) | -1(1) | 0(1) | 0(1) |
| C(15) | 8(1) | 13(1) | 14(1) | 1(1) | 0(1) | 2(1) |

*Table 6. Hydrogen coordinates ($x10^4$) and isotropic displacement parameters ($Å^2x10^3$) for ent-Isoineleganolide A (**29**).*

| | x | y | z | U(eq) |
|---|---|---|---|---|
| H(2) | 4698 | 5133 | 3818 | 13 |
| H(4A) | 4519 | 3874 | 5897 | 17 |
| H(4B) | 3539 | 5160 | 6051 | 17 |
| H(5) | 6428 | 4140 | 7829 | 15 |
| H(17A) | 792 | 4315 | 8777 | 34 |
| H(17B) | 1155 | 4422 | 7285 | 34 |
| H(17C) | 1692 | 5519 | 8233 | 34 |
| H(18A) | 3058 | 3018 | 9819 | 25 |
| H(18B) | 5338 | 3009 | 9467 | 25 |
| H(6A) | 4587 | 6435 | 7960 | 17 |
| H(6B) | 6141 | 5920 | 9028 | 17 |
| H(9A) | 8762 | 7428 | 5061 | 19 |
| H(9B) | 10228 | 6922 | 6176 | 19 |
| H(10) | 11720 | 6319 | 4387 | 16 |
| H(4O) | 11890(40) | 4050(20) | 2350(30) | 26 |
| H(19A) | 11784 | 7003 | 2089 | 37 |
| H(19B) | 9605 | 7106 | 1475 | 37 |
| H(19C) | 11245 | 6532 | 660 | 37 |
| H(13A) | 8498 | 5155 | 360 | 21 |
| H(13B) | 9165 | 3896 | 999 | 21 |
| H(14) | 5727 | 4906 | 1364 | 17 |
| H(15) | 6769 | 6385 | 2859 | 14 |

*Table 7. Torsion angles [°] for ent-Isoineleganolide A (**29**).*

| | |
|---|---|
| C(14)-O(1)-C(1)-O(2) | -179.60(13) |
| C(14)-O(1)-C(1)-C(2) | 2.52(15) |
| O(2)-C(1)-C(2)-C(3) | 23.4(2) |
| O(1)-C(1)-C(2)-C(3) | -158.97(11) |
| O(2)-C(1)-C(2)-C(15) | 158.84(15) |
| O(1)-C(1)-C(2)-C(15) | -23.52(13) |
| C(15)-C(2)-C(3)-C(8) | -3.9(2) |
| C(1)-C(2)-C(3)-C(8) | 120.54(15) |
| C(15)-C(2)-C(3)-C(4) | 169.78(12) |
| C(1)-C(2)-C(3)-C(4) | -65.73(15) |
| C(8)-C(3)-C(4)-C(5) | -23.87(19) |
| C(2)-C(3)-C(4)-C(5) | 162.09(11) |
| C(3)-C(4)-C(5)-C(16) | 177.19(12) |
| C(3)-C(4)-C(5)-C(6) | 54.15(15) |
| C(6)-C(5)-C(16)-C(18) | -106.25(16) |
| C(4)-C(5)-C(16)-C(18) | 132.96(15) |
| C(6)-C(5)-C(16)-C(17) | 71.09(16) |
| C(4)-C(5)-C(16)-C(17) | -49.70(18) |
| C(16)-C(5)-C(6)-C(7) | 178.75(12) |
| C(4)-C(5)-C(6)-C(7) | -56.22(15) |
| C(5)-C(6)-C(7)-O(3) | -153.51(14) |
| C(5)-C(6)-C(7)-C(8) | 28.95(18) |
| C(2)-C(3)-C(8)-C(7) | 166.22(13) |
| C(4)-C(3)-C(8)-C(7) | -6.9(2) |
| C(2)-C(3)-C(8)-C(9) | -7.4(2) |
| C(4)-C(3)-C(8)-C(9) | 179.48(14) |
| O(3)-C(7)-C(8)-C(3) | -173.25(14) |
| C(6)-C(7)-C(8)-C(3) | 4.3(2) |
| O(3)-C(7)-C(8)-C(9) | 1.4(2) |
| C(6)-C(7)-C(8)-C(9) | 178.87(13) |
| C(3)-C(8)-C(9)-C(10) | -36.1(2) |
| C(7)-C(8)-C(9)-C(10) | 149.96(13) |
| C(8)-C(9)-C(10)-C(11) | 55.5(2) |
| C(8)-C(9)-C(10)-O(5) | -14.76(19) |

*Table 7. (cont'd)*

| | |
|---|---|
| C(9)-C(10)-O(5)-C(11) | 112.51(14) |
| C(10)-O(5)-C(11)-C(15) | -113.83(13) |
| C(10)-O(5)-C(11)-C(12) | 116.22(14) |
| C(9)-C(10)-C(11)-O(5) | -106.68(15) |
| O(5)-C(10)-C(11)-C(15) | 104.08(14) |
| C(9)-C(10)-C(11)-C(15) | -2.6(2) |
| O(5)-C(10)-C(11)-C(12) | -104.06(14) |
| C(9)-C(10)-C(11)-C(12) | 149.26(14) |
| O(5)-C(11)-C(12)-O(4) | -16.85(17) |
| C(10)-C(11)-C(12)-O(4) | 54.41(19) |
| C(15)-C(11)-C(12)-O(4) | -150.44(12) |
| O(5)-C(11)-C(12)-C(19) | -135.03(13) |
| C(10)-C(11)-C(12)-C(19) | -63.77(18) |
| C(15)-C(11)-C(12)-C(19) | 91.38(14) |
| O(5)-C(11)-C(12)-C(13) | 105.68(13) |
| C(10)-C(11)-C(12)-C(13) | 176.95(13) |
| C(15)-C(11)-C(12)-C(13) | -27.91(15) |
| O(4)-C(12)-C(13)-C(14) | 131.35(13) |
| C(19)-C(12)-C(13)-C(14) | -108.65(15) |
| C(11)-C(12)-C(13)-C(14) | 9.26(16) |
| C(1)-O(1)-C(14)-C(13) | 133.46(12) |
| C(1)-O(1)-C(14)-C(15) | 19.59(14) |
| C(12)-C(13)-C(14)-O(1) | -99.06(14) |
| C(12)-C(13)-C(14)-C(15) | 11.85(17) |
| O(5)-C(11)-C(15)-C(2) | 11.18(17) |
| C(10)-C(11)-C(15)-C(2) | -59.03(18) |
| C(12)-C(11)-C(15)-C(2) | 145.14(12) |
| O(5)-C(11)-C(15)-C(14) | -98.98(13) |
| C(10)-C(11)-C(15)-C(14) | -169.19(13) |
| C(12)-C(11)-C(15)-C(14) | 34.99(14) |
| C(3)-C(2)-C(15)-C(11) | 54.29(18) |
| C(1)-C(2)-C(15)-C(11) | -76.18(13) |
| C(3)-C(2)-C(15)-C(14) | 164.26(12) |
| C(1)-C(2)-C(15)-C(14) | 33.79(12) |

*Table 7. (cont'd)*

| | |
|---|---|
| O(1)-C(14)-C(15)-C(11) | 86.14(12) |
| C(13)-C(14)-C(15)-C(11) | -28.29(14) |
| O(1)-C(14)-C(15)-C(2) | -33.17(13) |
| C(13)-C(14)-C(15)-C(2) | -147.59(12) |

Symmetry transformations used to generate equivalent atoms:

*Table 8. Hydrogen bonds for ent-Isoineleganolide A (**29**) [Å and °].*

| D-H...A | d(D-H) | d(H...A) | d(D...A) | <(DHA) |
|---|---|---|---|---|
| C(2)-H(2)...O(4)#1 | 1.00 | 2.43 | 3.2893(17) | 143.6 |
| C(6)-H(6A)...O(1)#2 | 0.99 | 2.55 | 3.4282(18) | 147.3 |
| C(10)-H(10)...O(2)#3 | 1.00 | 2.43 | 3.2055(18) | 133.5 |
| O(4)-H(4O)...O(3)#4 | 0.83(2) | 2.04(2) | 2.8276(19) | 158(3) |

Symmetry transformations used to generate equivalent atoms:

#1 x-1,y,z #2 -x+1,y+1/2,-z+1 #3 -x+2,y+1/2,-z+1

#4 -x+2,y-1/2,-z+1

## X-Ray Crystal Structure Analysis of Bromide 30

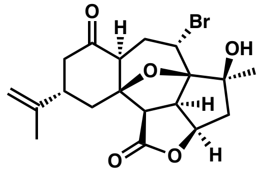


***30***

Contents

Table 1. Experimental Details

Table 2. Crystal Data

Table 3. Atomic Coordinates

Table 4. Full Bond Distances and Angles

Table 5. Anisotropic Displacement Parameters

Table 6. Hydrogen Atomic Coordinates

Table 7. Torsion Angles

Table 8. Hydrogen Bond Distances and Angles

*X-Ray Crystal Structure of Bromide* ***30***

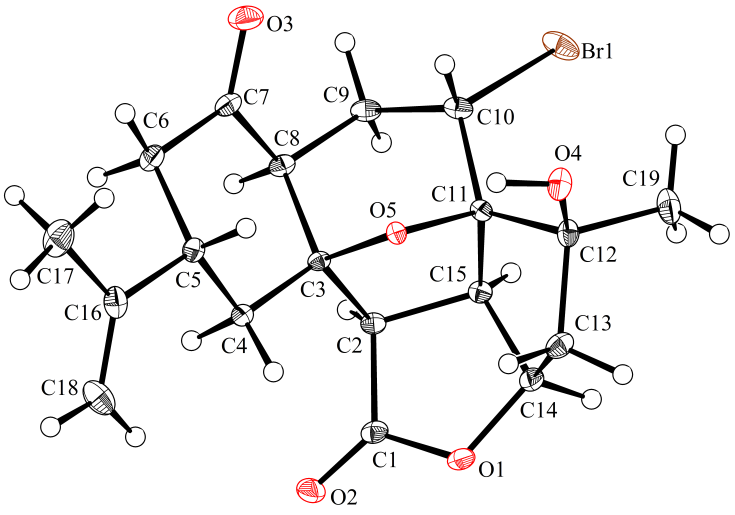

*Table 1. Experimental Details for X-Ray Structure Determination of Bromide **30**.*

Low-temperature diffraction data ($\phi$-and $\omega$-scans) were collected on a Bruker AXS KAPPA APEX II diffractometer coupled to a APEX II CCD detector with graphite monochromated Mo $K_\alpha$ radiation ($\lambda$ = 0.71073 Å) for the structure of bromide **30**. The structure was solved by direct methods using SHELXS[i] and refined against $F^2$ on all data by full-matrix least squares with SHELXL-2014[ii] using established refinement techniques.[iii] All non-hydrogen atoms were refined anisotropically. Unless otherwise noted, all hydrogen atoms were included into the model at geometrically calculated positions and refined using a riding model. The isotropic displacement parameters of all hydrogen atoms were fixed to 1.2 times the $U$ value of the atoms they are linked to (1.5 times for methyl groups).

Bromide **30** crystallizes in the orthorhombic space group $P2_12_12_1$ with one molecule in the asymmetric unit. The coordinates for the hydrogen atom bound to O4 was located in the difference Fourier synthesis and refined semi-freely with the help of a restraint on the O-H distance (0.84(4) Å).

*Table 2. Crystal Data and Structure Refinement for Bromide **30**.*

| | | |
|---|---|---|
| Caltech Identification code | rac10 | |
| CCDC Deposition Number | 1061013 | |
| Empirical formula | C19 H23 Br O5 | |
| Formula weight | 411.28 | |
| Temperature | 100(2) K | |
| Wavelength | 0.71073 Å | |
| Crystal system | Orthorhombic | |
| Space group | $P2_12_12_1$ | |
| Unit cell dimensions | a = 7.6137(4) Å | α= 90°. |
| | b = 9.3584(5) Å | β= 90°. |
| | c = 24.1592(13) Å | γ = 90°. |
| Volume | 1721.39(16) $Å^3$ | |
| Z | 4 | |
| Density (calculated) | 1.587 $Mg/m^3$ | |
| Absorption coefficient | 2.416 $mm^{-1}$ | |
| F(000) | 848 | |
| Crystal size | 0.500 x 0.450 x 0.100 $mm^3$ | |
| Theta range for data collection | 2.334 to 36.318°. | |
| Index ranges | $-12 \leq h \leq 12, -15 \leq k \leq 14, -39 \leq l \leq 40$ | |
| Reflections collected | 51998 | |
| Independent reflections | 8272 [R(int) = 0.0380] | |
| Completeness to theta = 25.242° | 99.9 % | |
| Absorption correction | Semi-empirical from equivalents | |
| Max. and min. transmission | 0.7478 and 0.5973 | |
| Refinement method | Full-matrix least-squares on $F^2$ | |
| Data / restraints / parameters | 8272 / 1 / 231 | |
| Goodness-of-fit on $F^2$ | 1.035 | |
| Final R indices [I>2sigma(I)] | R1 = 0.0251, wR2 = 0.0613 | |
| R indices (all data) | R1 = 0.0297, wR2 = 0.0625 | |
| Absolute structure parameter | 0.014(2) | |
| Extinction coefficient | n/a | |
| Largest diff. peak and hole | 0.746 and -0.367 e.$Å^{-3}$ | |

*Table 3. Atomic coordinates (x $10^4$) and equivalent isotropic displacement parameters ($Å^2$x $10^3$) for Bromide **30**. U(eq) is defined as one third of the trace of the orthogonalized $U^{ij}$ tensor.*

| | x | y | z | U(eq) |
|---|---|---|---|---|
| O(1) | 6345(1) | 6209(1) | 7159(1) | 14(1) |
| C(1) | 4823(2) | 6108(2) | 6871(1) | 11(1) |
| O(2) | 3840(2) | 7103(1) | 6827(1) | 16(1) |
| C(2) | 4583(2) | 4619(1) | 6642(1) | 10(1) |
| C(3) | 5114(2) | 4473(2) | 6017(1) | 8(1) |
| O(5) | 6928(1) | 4038(1) | 6041(1) | 8(1) |
| C(4) | 5024(2) | 5837(2) | 5681(1) | 10(1) |
| C(5) | 5590(2) | 5618(2) | 5077(1) | 11(1) |
| C(6) | 4489(2) | 4411(2) | 4820(1) | 13(1) |
| C(7) | 4584(2) | 3064(2) | 5155(1) | 12(1) |
| O(3) | 5000(2) | 1919(1) | 4955(1) | 19(1) |
| C(8) | 4104(2) | 3212(2) | 5760(1) | 10(1) |
| C(9) | 4411(2) | 1846(2) | 6093(1) | 12(1) |
| C(10) | 6347(2) | 1653(1) | 6251(1) | 11(1) |
| Br(1) | 6456(1) | -42(1) | 6729(1) | 19(1) |
| C(11) | 7078(2) | 3035(1) | 6497(1) | 8(1) |
| C(12) | 9005(2) | 3218(2) | 6685(1) | 11(1) |
| O(4) | 10208(1) | 3067(1) | 6239(1) | 14(1) |
| C(13) | 8951(2) | 4740(2) | 6926(1) | 14(1) |
| C(14) | 7176(2) | 4824(2) | 7226(1) | 12(1) |
| C(15) | 5948(2) | 3720(2) | 6947(1) | 10(1) |
| C(16) | 5502(2) | 6970(2) | 4728(1) | 15(1) |
| C(17) | 5870(2) | 6771(2) | 4117(1) | 22(1) |
| C(18) | 5173(2) | 8249(2) | 4935(1) | 20(1) |
| C(19) | 9621(2) | 2179(2) | 7128(1) | 18(1) |

*Table 4. Bond lengths [Å] and angles [°] for Bromide **30**.*

| | |
|---|---|
| O(1)-C(1) | 1.3544(17) |
| O(1)-C(14) | 1.4514(18) |
| C(1)-O(2) | 1.1989(18) |
| C(1)-C(2) | 1.5115(19) |
| C(2)-C(15) | 1.5264(19) |
| C(2)-C(3) | 1.5679(18) |
| C(2)-H(2) | 1.0000 |
| C(3)-O(5) | 1.4409(14) |
| C(3)-C(4) | 1.515(2) |
| C(3)-C(8) | 1.5388(18) |
| O(5)-C(11) | 1.4519(16) |
| C(4)-C(5) | 1.5342(18) |
| C(4)-H(4A) | 0.9900 |
| C(4)-H(4B) | 0.9900 |
| C(5)-C(16) | 1.522(2) |
| C(5)-C(6) | 1.538(2) |
| C(5)-H(5) | 1.0000 |
| C(6)-C(7) | 1.500(2) |
| C(6)-H(6A) | 0.9900 |
| C(6)-H(6B) | 0.9900 |
| C(7)-O(3) | 1.2170(17) |
| C(7)-C(8) | 1.5142(19) |
| C(8)-C(9) | 1.527(2) |
| C(8)-H(8) | 1.0000 |
| C(9)-C(10) | 1.5334(19) |
| C(9)-H(9A) | 0.9900 |
| C(9)-H(9B) | 0.9900 |
| C(10)-C(11) | 1.5275(19) |
| C(10)-Br(1) | 1.9635(13) |
| C(10)-H(10) | 1.0000 |
| C(11)-C(15) | 1.5273(18) |
| C(11)-C(12) | 1.5456(17) |
| C(12)-O(4) | 1.4205(16) |

*Table 4. (cont'd)*

| | |
|---|---|
| C(12)-C(19) | 1.520(2) |
| C(12)-C(13) | 1.539(2) |
| O(4)-H(4O) | 0.893(18) |
| C(13)-C(14) | 1.5356(18) |
| C(13)-H(13A) | 0.9900 |
| C(13)-H(13B) | 0.9900 |
| C(14)-C(15) | 1.5484(19) |
| C(14)-H(14) | 1.0000 |
| C(15)-H(15) | 1.0000 |
| C(16)-C(18) | 1.322(2) |
| C(16)-C(17) | 1.512(2) |
| C(17)-H(17A) | 0.9800 |
| C(17)-H(17B) | 0.9800 |
| C(17)-H(17C) | 0.9800 |
| C(18)-H(18A) | 0.9500 |
| C(18)-H(18B) | 0.9500 |
| C(19)-H(19A) | 0.9800 |
| C(19)-H(19B) | 0.9800 |
| C(19)-H(19C) | 0.9800 |
| | |
| C(1)-O(1)-C(14) | 111.60(11) |
| O(2)-C(1)-O(1) | 121.69(13) |
| O(2)-C(1)-C(2) | 127.45(13) |
| O(1)-C(1)-C(2) | 110.85(11) |
| C(1)-C(2)-C(15) | 104.40(11) |
| C(1)-C(2)-C(3) | 113.74(11) |
| C(15)-C(2)-C(3) | 103.96(10) |
| C(1)-C(2)-H(2) | 111.4 |
| C(15)-C(2)-H(2) | 111.4 |
| C(3)-C(2)-H(2) | 111.4 |
| O(5)-C(3)-C(4) | 107.61(10) |
| O(5)-C(3)-C(8) | 106.18(10) |
| C(4)-C(3)-C(8) | 114.06(11) |
| O(5)-C(3)-C(2) | 103.45(10) |

*Table 4. (cont'd)*

| | |
|---|---|
| C(4)-C(3)-C(2) | 115.53(11) |
| C(8)-C(3)-C(2) | 109.04(10) |
| C(3)-O(5)-C(11) | 106.78(9) |
| C(3)-C(4)-C(5) | 112.61(11) |
| C(3)-C(4)-H(4A) | 109.1 |
| C(5)-C(4)-H(4A) | 109.1 |
| C(3)-C(4)-H(4B) | 109.1 |
| C(5)-C(4)-H(4B) | 109.1 |
| H(4A)-C(4)-H(4B) | 107.8 |
| C(16)-C(5)-C(4) | 113.83(12) |
| C(16)-C(5)-C(6) | 111.21(11) |
| C(4)-C(5)-C(6) | 109.26(11) |
| C(16)-C(5)-H(5) | 107.4 |
| C(4)-C(5)-H(5) | 107.4 |
| C(6)-C(5)-H(5) | 107.4 |
| C(7)-C(6)-C(5) | 111.86(11) |
| C(7)-C(6)-H(6A) | 109.2 |
| C(5)-C(6)-H(6A) | 109.2 |
| C(7)-C(6)-H(6B) | 109.2 |
| C(5)-C(6)-H(6B) | 109.2 |
| H(6A)-C(6)-H(6B) | 107.9 |
| O(3)-C(7)-C(6) | 122.61(13) |
| O(3)-C(7)-C(8) | 121.70(13) |
| C(6)-C(7)-C(8) | 115.69(12) |
| C(7)-C(8)-C(9) | 113.28(12) |
| C(7)-C(8)-C(3) | 109.82(11) |
| C(9)-C(8)-C(3) | 110.68(11) |
| C(7)-C(8)-H(8) | 107.6 |
| C(9)-C(8)-H(8) | 107.6 |
| C(3)-C(8)-H(8) | 107.6 |
| C(8)-C(9)-C(10) | 112.12(11) |
| C(8)-C(9)-H(9A) | 109.2 |
| C(10)-C(9)-H(9A) | 109.2 |
| C(8)-C(9)-H(9B) | 109.2 |

*Table 4. (cont'd)*

| | |
|---|---|
| C(10)-C(9)-H(9B) | 109.2 |
| H(9A)-C(9)-H(9B) | 107.9 |
| C(11)-C(10)-C(9) | 110.33(11) |
| C(11)-C(10)-Br(1) | 116.09(9) |
| C(9)-C(10)-Br(1) | 106.38(9) |
| C(11)-C(10)-H(10) | 107.9 |
| C(9)-C(10)-H(10) | 107.9 |
| Br(1)-C(10)-H(10) | 107.9 |
| O(5)-C(11)-C(15) | 102.90(10) |
| O(5)-C(11)-C(10) | 102.94(10) |
| C(15)-C(11)-C(10) | 115.27(11) |
| O(5)-C(11)-C(12) | 103.07(10) |
| C(15)-C(11)-C(12) | 106.18(10) |
| C(10)-C(11)-C(12) | 123.66(11) |
| O(4)-C(12)-C(19) | 105.71(11) |
| O(4)-C(12)-C(13) | 113.32(11) |
| C(19)-C(12)-C(13) | 109.53(12) |
| O(4)-C(12)-C(11) | 112.19(11) |
| C(19)-C(12)-C(11) | 115.40(12) |
| C(13)-C(12)-C(11) | 100.89(10) |
| C(12)-O(4)-H(4O) | 108.7(15) |
| C(14)-C(13)-C(12) | 104.44(11) |
| C(14)-C(13)-H(13A) | 110.9 |
| C(12)-C(13)-H(13A) | 110.9 |
| C(14)-C(13)-H(13B) | 110.9 |
| C(12)-C(13)-H(13B) | 110.9 |
| H(13A)-C(13)-H(13B) | 108.9 |
| O(1)-C(14)-C(13) | 112.12(12) |
| O(1)-C(14)-C(15) | 106.49(10) |
| C(13)-C(14)-C(15) | 106.94(11) |
| O(1)-C(14)-H(14) | 110.4 |
| C(13)-C(14)-H(14) | 110.4 |
| C(15)-C(14)-H(14) | 110.4 |
| C(2)-C(15)-C(11) | 105.75(10) |

*Table 4. (cont'd)*

| | |
|---|---|
| C(2)-C(15)-C(14) | 104.71(11) |
| C(11)-C(15)-C(14) | 104.51(10) |
| C(2)-C(15)-H(15) | 113.6 |
| C(11)-C(15)-H(15) | 113.6 |
| C(14)-C(15)-H(15) | 113.6 |
| C(18)-C(16)-C(17) | 121.05(15) |
| C(18)-C(16)-C(5) | 123.41(15) |
| C(17)-C(16)-C(5) | 115.52(14) |
| C(16)-C(17)-H(17A) | 109.5 |
| C(16)-C(17)-H(17B) | 109.5 |
| H(17A)-C(17)-H(17B) | 109.5 |
| C(16)-C(17)-H(17C) | 109.5 |
| H(17A)-C(17)-H(17C) | 109.5 |
| H(17B)-C(17)-H(17C) | 109.5 |
| C(16)-C(18)-H(18A) | 120.0 |
| C(16)-C(18)-H(18B) | 120.0 |
| H(18A)-C(18)-H(18B) | 120.0 |
| C(12)-C(19)-H(19A) | 109.5 |
| C(12)-C(19)-H(19B) | 109.5 |
| H(19A)-C(19)-H(19B) | 109.5 |
| C(12)-C(19)-H(19C) | 109.5 |
| H(19A)-C(19)-H(19C) | 109.5 |
| H(19B)-C(19)-H(19C) | 109.5 |

Symmetry transformations used to generate equivalent atoms:

Table 5. Anisotropic displacement parameters ($Å^2x10^3$) for Bromide **30**. The anisotropic displacement factor exponent takes the form: $-2\pi^2[ h^2a^{*2}U^{11} + ... + 2hka^*b^*U^{12}]$.

| | $U^{11}$ | $U^{22}$ | $U^{33}$ | $U^{23}$ | $U^{13}$ | $U^{12}$ |
|---|---|---|---|---|---|---|
| O(1) | 15(1) | 12(1) | 13(1) | -4(1) | -2(1) | 0(1) |
| C(1) | 14(1) | 12(1) | 8(1) | -1(1) | 3(1) | -1(1) |
| O(2) | 21(1) | 14(1) | 13(1) | -2(1) | 1(1) | 5(1) |
| C(2) | 10(1) | 10(1) | 9(1) | -1(1) | 2(1) | 0(1) |
| C(3) | 7(1) | 9(1) | 9(1) | -2(1) | 1(1) | 0(1) |
| O(5) | 7(1) | 9(1) | 8(1) | 2(1) | 1(1) | 0(1) |
| C(4) | 12(1) | 10(1) | 10(1) | -1(1) | -2(1) | 0(1) |
| C(5) | 11(1) | 12(1) | 9(1) | 1(1) | -1(1) | 0(1) |
| C(6) | 15(1) | 15(1) | 10(1) | -1(1) | -2(1) | -1(1) |
| C(7) | 11(1) | 13(1) | 11(1) | -3(1) | 0(1) | -2(1) |
| O(3) | 28(1) | 15(1) | 14(1) | -5(1) | 2(1) | 1(1) |
| C(8) | 9(1) | 12(1) | 11(1) | -2(1) | 1(1) | -2(1) |
| C(9) | 13(1) | 9(1) | 14(1) | -2(1) | 3(1) | -4(1) |
| C(10) | 14(1) | 9(1) | 11(1) | 0(1) | 5(1) | -1(1) |
| Br(1) | 27(1) | 9(1) | 22(1) | 5(1) | 8(1) | 2(1) |
| C(11) | 10(1) | 8(1) | 8(1) | 1(1) | 2(1) | 1(1) |
| C(12) | 10(1) | 15(1) | 8(1) | 2(1) | 1(1) | 2(1) |
| O(4) | 9(1) | 23(1) | 10(1) | 1(1) | 2(1) | 2(1) |
| C(13) | 11(1) | 16(1) | 13(1) | -3(1) | 0(1) | -2(1) |
| C(14) | 13(1) | 13(1) | 10(1) | -2(1) | 0(1) | 1(1) |
| C(15) | 11(1) | 10(1) | 8(1) | 0(1) | 2(1) | 0(1) |
| C(16) | 12(1) | 16(1) | 15(1) | 5(1) | -2(1) | -1(1) |
| C(17) | 28(1) | 25(1) | 13(1) | 6(1) | -1(1) | -5(1) |
| C(18) | 21(1) | 16(1) | 24(1) | 7(1) | 3(1) | 3(1) |
| C(19) | 17(1) | 23(1) | 14(1) | 6(1) | -1(1) | 6(1) |

*Table 6. Hydrogen coordinates ($x10^4$) and isotropic displacement parameters ($Å^2x10^3$) for Bromide **30**.*

| | x | y | z | U(eq) |
|---|---|---|---|---|
| H(2) | 3367 | 4256 | 6709 | 12 |
| H(4A) | 5792 | 6564 | 5854 | 13 |
| H(4B) | 3806 | 6206 | 5688 | 13 |
| H(5) | 6841 | 5293 | 5081 | 13 |
| H(6A) | 3250 | 4723 | 4792 | 16 |
| H(6B) | 4919 | 4217 | 4440 | 16 |
| H(8) | 2823 | 3441 | 5781 | 13 |
| H(9A) | 4023 | 1014 | 5872 | 15 |
| H(9B) | 3691 | 1877 | 6434 | 15 |
| H(10) | 7020 | 1439 | 5905 | 14 |
| H(4O) | 9730(30) | 3450(20) | 5936(8) | 21 |
| H(13A) | 9934 | 4894 | 7188 | 16 |
| H(13B) | 9022 | 5464 | 6628 | 16 |
| H(14) | 7323 | 4596 | 7628 | 14 |
| H(15) | 5427 | 3016 | 7211 | 11 |
| H(17A) | 4884 | 6268 | 3944 | 33 |
| H(17B) | 6946 | 6209 | 4071 | 33 |
| H(17C) | 6019 | 7708 | 3942 | 33 |
| H(18A) | 5165 | 9063 | 4700 | 24 |
| H(18B) | 4944 | 8356 | 5320 | 24 |
| H(19A) | 9719 | 1219 | 6968 | 27 |
| H(19B) | 8772 | 2164 | 7432 | 27 |
| H(19C) | 10770 | 2482 | 7268 | 27 |

*Table 7. Torsion angles [°] for Bromide **30**.*

| | |
|---|---|
| C(14)-O(1)-C(1)-O(2) | 171.18(12) |
| C(14)-O(1)-C(1)-C(2) | -7.77(14) |
| O(2)-C(1)-C(2)-C(15) | -165.39(13) |
| O(1)-C(1)-C(2)-C(15) | 13.48(14) |
| O(2)-C(1)-C(2)-C(3) | 81.97(16) |
| O(1)-C(1)-C(2)-C(3) | -99.16(12) |
| C(1)-C(2)-C(3)-O(5) | 92.31(12) |
| C(15)-C(2)-C(3)-O(5) | -20.60(12) |
| C(1)-C(2)-C(3)-C(4) | -25.01(14) |
| C(15)-C(2)-C(3)-C(4) | -137.92(11) |
| C(1)-C(2)-C(3)-C(8) | -155.00(11) |
| C(15)-C(2)-C(3)-C(8) | 92.08(12) |
| C(4)-C(3)-O(5)-C(11) | 162.08(10) |
| C(8)-C(3)-O(5)-C(11) | -75.41(12) |
| C(2)-C(3)-O(5)-C(11) | 39.34(12) |
| O(5)-C(3)-C(4)-C(5) | 64.07(13) |
| C(8)-C(3)-C(4)-C(5) | -53.44(13) |
| C(2)-C(3)-C(4)-C(5) | 179.03(10) |
| C(3)-C(4)-C(5)-C(16) | 179.64(10) |
| C(3)-C(4)-C(5)-C(6) | 54.66(13) |
| C(16)-C(5)-C(6)-C(7) | 179.13(11) |
| C(4)-C(5)-C(6)-C(7) | -54.39(15) |
| C(5)-C(6)-C(7)-O(3) | -126.40(14) |
| C(5)-C(6)-C(7)-C(8) | 54.29(15) |
| O(3)-C(7)-C(8)-C(9) | 6.81(18) |
| C(6)-C(7)-C(8)-C(9) | -173.87(11) |
| O(3)-C(7)-C(8)-C(3) | 131.12(14) |
| C(6)-C(7)-C(8)-C(3) | -49.56(14) |
| O(5)-C(3)-C(8)-C(7) | -69.79(13) |
| C(4)-C(3)-C(8)-C(7) | 48.54(14) |
| C(2)-C(3)-C(8)-C(7) | 179.34(10) |
| O(5)-C(3)-C(8)-C(9) | 56.02(13) |
| C(4)-C(3)-C(8)-C(9) | 174.35(10) |
| C(2)-C(3)-C(8)-C(9) | -54.86(13) |

*Table 7. (cont'd)*

| | |
|---|---|
| C(7)-C(8)-C(9)-C(10) | 79.81(14) |
| C(3)-C(8)-C(9)-C(10) | -44.03(14) |
| C(8)-C(9)-C(10)-C(11) | 48.25(14) |
| C(8)-C(9)-C(10)-Br(1) | 174.95(9) |
| C(3)-O(5)-C(11)-C(15) | -41.94(12) |
| C(3)-O(5)-C(11)-C(10) | 78.19(11) |
| C(3)-O(5)-C(11)-C(12) | -152.22(10) |
| C(9)-C(10)-C(11)-O(5) | -62.73(12) |
| Br(1)-C(10)-C(11)-O(5) | 176.18(7) |
| C(9)-C(10)-C(11)-C(15) | 48.48(15) |
| Br(1)-C(10)-C(11)-C(15) | -72.60(13) |
| C(9)-C(10)-C(11)-C(12) | -178.32(11) |
| Br(1)-C(10)-C(11)-C(12) | 60.60(14) |
| O(5)-C(11)-C(12)-O(4) | -52.81(14) |
| C(15)-C(11)-C(12)-O(4) | -160.63(12) |
| C(10)-C(11)-C(12)-O(4) | 62.71(17) |
| O(5)-C(11)-C(12)-C(19) | -173.97(12) |
| C(15)-C(11)-C(12)-C(19) | 78.21(15) |
| C(10)-C(11)-C(12)-C(19) | -58.45(17) |
| O(5)-C(11)-C(12)-C(13) | 68.11(11) |
| C(15)-C(11)-C(12)-C(13) | -39.71(13) |
| C(10)-C(11)-C(12)-C(13) | -176.37(12) |
| O(4)-C(12)-C(13)-C(14) | 159.21(11) |
| C(19)-C(12)-C(13)-C(14) | -83.03(13) |
| C(11)-C(12)-C(13)-C(14) | 39.09(12) |
| C(1)-O(1)-C(14)-C(13) | 115.35(12) |
| C(1)-O(1)-C(14)-C(15) | -1.29(14) |
| C(12)-C(13)-C(14)-O(1) | -141.44(11) |
| C(12)-C(13)-C(14)-C(15) | -25.07(14) |
| C(1)-C(2)-C(15)-C(11) | -123.39(11) |
| C(3)-C(2)-C(15)-C(11) | -3.91(13) |
| C(1)-C(2)-C(15)-C(14) | -13.32(12) |
| C(3)-C(2)-C(15)-C(14) | 106.15(11) |
| O(5)-C(11)-C(15)-C(2) | 26.87(13) |

*Table 7. (cont'd)*

| | |
|---|---|
| C(10)-C(11)-C(15)-C(2) | -84.37(13) |
| C(12)-C(11)-C(15)-C(2) | 134.81(11) |
| O(5)-C(11)-C(15)-C(14) | -83.34(11) |
| C(10)-C(11)-C(15)-C(14) | 165.42(11) |
| C(12)-C(11)-C(15)-C(14) | 24.60(14) |
| O(1)-C(14)-C(15)-C(2) | 9.44(13) |
| C(13)-C(14)-C(15)-C(2) | -110.61(12) |
| O(1)-C(14)-C(15)-C(11) | 120.40(11) |
| C(13)-C(14)-C(15)-C(11) | 0.35(14) |
| C(4)-C(5)-C(16)-C(18) | 7.60(19) |
| C(6)-C(5)-C(16)-C(18) | 131.52(15) |
| C(4)-C(5)-C(16)-C(17) | -174.03(12) |
| C(6)-C(5)-C(16)-C(17) | -50.10(16) |

Symmetry transformations used to generate equivalent atoms:

*Table 8. Hydrogen bonds for Bromide **30** [Å and °].*

| D-H...A | d(D-H) | d(H...A) | d(D...A) | <(DHA) |
|---|---|---|---|---|
| C(8)-H(8)...O(4)#1 | 1.00 | 2.31 | 3.1874(15) | 146.5 |
| O(4)-H(4O)...O(5) | 0.893(18) | 2.22(2) | 2.7004(14) | 113.4(17) |
| O(4)-H(4O)...O(3)#2 | 0.893(18) | 2.19(2) | 2.8908(16) | 135.0(19) |
| C(15)-H(15)...O(1)#3 | 1.00 | 2.65 | 3.6389(17) | 172.5 |
| C(15)-H(15)...O(2)#3 | 1.00 | 2.54 | 3.3305(17) | 136.0 |
| C(19)-H(19A)...Br(1) | 0.98 | 2.81 | 3.3248(17) | 113.5 |

Symmetry transformations used to generate equivalent atoms:

#1 x-1,y,z #2 x+1/2,-y+1/2,-z+1 #3 -x+1,y-1/2,-z+3/2

## X-Ray Crystal Structure Analysis of Ketopyran 31

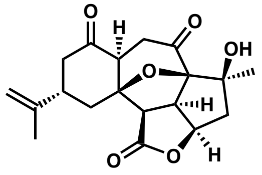

***31***

Contents


Table 1. Experimental Details

Table 2. Crystal Data

Table 3. Atomic Coordinates

Table 4. Full Bond Distances and Angles

Table 5. Anisotropic Displacement Parameters

Table 6. Hydrogen Atomic Coordinates

Table 7. Torsion Angles

Table 8. Hydrogen Bond Distances and Angles


*X-Ray Crystal Structure of Ketopyran **31***

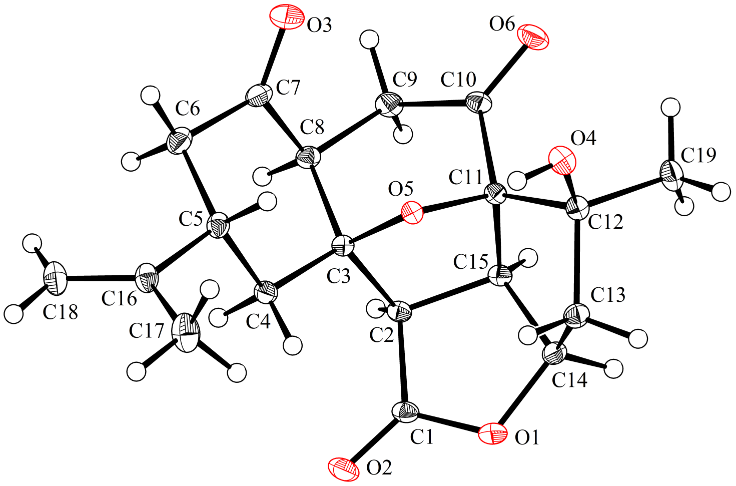

*Table 1. Experimental Details for X-Ray Structure Determination of Ketopyran* ***31***.

Low-temperature diffraction data ($\phi$-and $\omega$-scans) were collected on a Bruker AXS KAPPA APEX II diffractometer coupled to a APEX II CCD detector with graphite monochromated Mo $K_\alpha$ radiation ($\lambda$ = 0.71073 Å) for the structure of ketopyran **31**. The structure was solved by direct methods using SHELXS[i] and refined against $F^2$ on all data by full-matrix least squares with SHELXL-2014[ii] using established refinement techniques.[iii] All non-hydrogen atoms were refined anisotropically. Unless otherwise noted, all hydrogen atoms were included into the model at geometrically calculated positions and refined using a riding model. The isotropic displacement parameters of all hydrogen atoms were fixed to 1.2 times the *U* value of the atoms they are linked to (1.5 times for methyl groups).

Ketopyran **31** crystallizes in the orthorhombic space group $P2_12_12_1$ with one molecule in the asymmetric unit. The coordinates for the hydrogen atom bound to O4 was located in the difference Fourier synthesis and refined semi-freely with the help of a restraint on the O-H distance (0.84(4) Å). The chirality of the molecule cannot be reliably determined from the diffraction data. The molecule could be the wrong enantiomer or a mixture of both enantiomers.

*Table 2. Crystal Data and Structure Refinement for Ketopyran **31**.*

| | | |
|---|---|---|
| Caltech Identification code | rac06 | |
| CCDC Deposition Number | 1061012 | |
| Empirical formula | C19 H22 O6 | |
| Formula weight | 346.36 | |
| Temperature | 100(2) K | |
| Wavelength | 0.71073 Å | |
| Crystal system | Orthorhombic | |
| Space group | $P2_12_12_1$ | |
| Unit cell dimensions | a = 8.2576(3) Å | α= 90°. |
| | b = 10.4049(4) Å | β= 90°. |
| | c = 19.0995(8) Å | γ = 90°. |
| Volume | 1641.02(11) $Å^3$ | |
| Z | 4 | |
| Density (calculated) | 1.402 $Mg/m^3$ | |
| Absorption coefficient | 0.104 $mm^{-1}$ | |
| F(000) | 736 | |
| Crystal size | 0.450 x 0.350 x 0.100 $mm^3$ | |
| Theta range for data collection | 2.133 to 43.738°. | |
| Index ranges | $-16 \le h \le 14, -20 \le k \le 18, -37 \le l \le 37$ | |
| Reflections collected | 85501 | |
| Independent reflections | 12633 [R(int) = 0.0489] | |
| Completeness to theta = 25.242° | 100.0 % | |
| Absorption correction | Semi-empirical from equivalents | |
| Max. and min. transmission | 0.7487 and 0.7039 | |
| Refinement method | Full-matrix least-squares on $F^2$ | |
| Data / restraints / parameters | 12633 / 1 / 231 | |
| Goodness-of-fit on $F^2$ | 1.060 | |
| Final R indices [I>2sigma(I)] | R1 = 0.0442, wR2 = 0.1107 | |
| R indices (all data) | R1 = 0.0613, wR2 = 0.1176 | |
| Absolute structure parameter | 0.35(16) | |
| Extinction coefficient | n/a | |
| Largest diff. peak and hole | 0.468 and -0.291 e.$Å^{-3}$ | |

*Table 3. Atomic coordinates (x $10^4$) and equivalent isotropic displacement parameters ($Å^2$x $10^3$) for Ketopyran **31**. U(eq) is defined as one third of the trace of the orthogonalized $U^{ij}$ tensor.*

| | x | y | z | U(eq) |
|---|---|---|---|---|
| O(1) | 10548(1) | 6025(1) | 1279(1) | 16(1) |
| C(1) | 9704(1) | 5326(1) | 1747(1) | 13(1) |
| O(2) | 10382(1) | 4599(1) | 2142(1) | 18(1) |
| C(2) | 7892(1) | 5564(1) | 1681(1) | 11(1) |
| C(3) | 7159(1) | 6320(1) | 2311(1) | 10(1) |
| O(5) | 7376(1) | 7644(1) | 2113(1) | 10(1) |
| C(4) | 8008(1) | 6121(1) | 3008(1) | 12(1) |
| C(5) | 7310(1) | 6968(1) | 3590(1) | 13(1) |
| C(16) | 8277(1) | 6778(1) | 4253(1) | 14(1) |
| C(17) | 9841(2) | 7514(1) | 4288(1) | 22(1) |
| C(18) | 7820(2) | 6000(1) | 4772(1) | 19(1) |
| C(6) | 5484(1) | 6699(1) | 3661(1) | 16(1) |
| C(7) | 4701(1) | 6954(1) | 2966(1) | 14(1) |
| O(3) | 3728(1) | 7814(1) | 2870(1) | 22(1) |
| C(8) | 5303(1) | 6116(1) | 2362(1) | 12(1) |
| C(9) | 4451(1) | 6481(1) | 1677(1) | 14(1) |
| C(10) | 5157(1) | 7655(1) | 1315(1) | 12(1) |
| O(6) | 4336(1) | 8392(1) | 971(1) | 20(1) |
| C(11) | 6996(1) | 7729(1) | 1379(1) | 10(1) |
| C(12) | 8007(1) | 8868(1) | 1102(1) | 12(1) |
| O(4) | 7848(1) | 9969(1) | 1533(1) | 15(1) |
| C(19) | 7572(2) | 9275(1) | 359(1) | 17(1) |
| C(13) | 9732(1) | 8287(1) | 1121(1) | 14(1) |
| C(14) | 9514(1) | 6892(1) | 888(1) | 13(1) |
| C(15) | 7753(1) | 6507(1) | 1067(1) | 10(1) |

*Table 4. Bond lengths [Å] and angles [°] for Ketopyran* ***31***.

| | |
|---|---|
| O(1)-C(1) | 1.3464(14) |
| O(1)-C(14) | 1.4494(13) |
| C(1)-O(2) | 1.2070(13) |
| C(1)-C(2) | 1.5217(14) |
| C(2)-C(15) | 1.5328(13) |
| C(2)-C(3) | 1.5593(13) |
| C(2)-H(2) | 1.0000 |
| C(3)-O(5) | 1.4396(12) |
| C(3)-C(4) | 1.5185(13) |
| C(3)-C(8) | 1.5501(13) |
| O(5)-C(11) | 1.4381(11) |
| C(4)-C(5) | 1.5318(13) |
| C(4)-H(4A) | 0.9900 |
| C(4)-H(4B) | 0.9900 |
| C(5)-C(16) | 1.5101(15) |
| C(5)-C(6) | 1.5397(15) |
| C(5)-H(5) | 1.0000 |
| C(16)-C(18) | 1.3338(15) |
| C(16)-C(17) | 1.5029(17) |
| C(17)-H(17A) | 0.9800 |
| C(17)-H(17B) | 0.9800 |
| C(17)-H(17C) | 0.9800 |
| C(18)-H(18A) | 0.9500 |
| C(18)-H(18B) | 0.9500 |
| C(6)-C(7) | 1.5010(16) |
| C(6)-H(6A) | 0.9900 |
| C(6)-H(6B) | 0.9900 |
| C(7)-O(3) | 1.2165(14) |
| C(7)-C(8) | 1.5292(14) |
| C(8)-C(9) | 1.5326(15) |
| C(8)-H(8) | 1.0000 |
| C(9)-C(10) | 1.5204(15) |
| C(9)-H(9A) | 0.9900 |

*Table 4. (cont'd)*

| | |
|---|---|
| C(9)-H(9B) | 0.9900 |
| C(10)-O(6) | 1.2150(13) |
| C(10)-C(11) | 1.5255(13) |
| C(11)-C(15) | 1.5379(13) |
| C(11)-C(12) | 1.5436(13) |
| C(12)-O(4) | 1.4169(13) |
| C(12)-C(19) | 1.5238(14) |
| C(12)-C(13) | 1.5477(14) |
| O(4)-H(4O) | 0.797(17) |
| C(19)-H(19A) | 0.9800 |
| C(19)-H(19B) | 0.9800 |
| C(19)-H(19C) | 0.9800 |
| C(13)-C(14) | 1.5296(15) |
| C(13)-H(13A) | 0.9900 |
| C(13)-H(13B) | 0.9900 |
| C(14)-C(15) | 1.5462(14) |
| C(14)-H(14) | 1.0000 |
| C(15)-H(15) | 1.0000 |

| | |
|---|---|
| C(1)-O(1)-C(14) | 111.90(8) |
| O(2)-C(1)-O(1) | 120.92(10) |
| O(2)-C(1)-C(2) | 127.58(10) |
| O(1)-C(1)-C(2) | 111.48(8) |
| C(1)-C(2)-C(15) | 103.94(8) |
| C(1)-C(2)-C(3) | 113.59(7) |
| C(15)-C(2)-C(3) | 103.82(7) |
| C(1)-C(2)-H(2) | 111.7 |
| C(15)-C(2)-H(2) | 111.7 |
| C(3)-C(2)-H(2) | 111.7 |
| O(5)-C(3)-C(4) | 107.67(7) |
| O(5)-C(3)-C(8) | 105.65(8) |
| C(4)-C(3)-C(8) | 112.54(8) |
| O(5)-C(3)-C(2) | 103.36(7) |
| C(4)-C(3)-C(2) | 115.38(8) |

*Table 4. (cont'd)*

| | |
|---|---|
| C(8)-C(3)-C(2) | 111.27(7) |
| C(11)-O(5)-C(3) | 106.77(7) |
| C(3)-C(4)-C(5) | 112.61(8) |
| C(3)-C(4)-H(4A) | 109.1 |
| C(5)-C(4)-H(4A) | 109.1 |
| C(3)-C(4)-H(4B) | 109.1 |
| C(5)-C(4)-H(4B) | 109.1 |
| H(4A)-C(4)-H(4B) | 107.8 |
| C(16)-C(5)-C(4) | 109.53(8) |
| C(16)-C(5)-C(6) | 114.87(8) |
| C(4)-C(5)-C(6) | 109.15(8) |
| C(16)-C(5)-H(5) | 107.7 |
| C(4)-C(5)-H(5) | 107.7 |
| C(6)-C(5)-H(5) | 107.7 |
| C(18)-C(16)-C(17) | 121.35(10) |
| C(18)-C(16)-C(5) | 123.55(11) |
| C(17)-C(16)-C(5) | 115.09(9) |
| C(16)-C(17)-H(17A) | 109.5 |
| C(16)-C(17)-H(17B) | 109.5 |
| H(17A)-C(17)-H(17B) | 109.5 |
| C(16)-C(17)-H(17C) | 109.5 |
| H(17A)-C(17)-H(17C) | 109.5 |
| H(17B)-C(17)-H(17C) | 109.5 |
| C(16)-C(18)-H(18A) | 120.0 |
| C(16)-C(18)-H(18B) | 120.0 |
| H(18A)-C(18)-H(18B) | 120.0 |
| C(7)-C(6)-C(5) | 108.16(8) |
| C(7)-C(6)-H(6A) | 110.1 |
| C(5)-C(6)-H(6A) | 110.1 |
| C(7)-C(6)-H(6B) | 110.1 |
| C(5)-C(6)-H(6B) | 110.1 |
| H(6A)-C(6)-H(6B) | 108.4 |
| O(3)-C(7)-C(6) | 123.18(10) |
| O(3)-C(7)-C(8) | 121.40(10) |

*Table 4. (cont'd)*

| | |
|---|---|
| C(6)-C(7)-C(8) | 115.28(9) |
| C(7)-C(8)-C(9) | 110.70(9) |
| C(7)-C(8)-C(3) | 106.90(8) |
| C(9)-C(8)-C(3) | 111.54(8) |
| C(7)-C(8)-H(8) | 109.2 |
| C(9)-C(8)-H(8) | 109.2 |
| C(3)-C(8)-H(8) | 109.2 |
| C(10)-C(9)-C(8) | 114.27(8) |
| C(10)-C(9)-H(9A) | 108.7 |
| C(8)-C(9)-H(9A) | 108.7 |
| C(10)-C(9)-H(9B) | 108.7 |
| C(8)-C(9)-H(9B) | 108.7 |
| H(9A)-C(9)-H(9B) | 107.6 |
| O(6)-C(10)-C(9) | 122.61(9) |
| O(6)-C(10)-C(11) | 124.53(10) |
| C(9)-C(10)-C(11) | 112.67(8) |
| O(5)-C(11)-C(10) | 107.03(7) |
| O(5)-C(11)-C(15) | 103.75(7) |
| C(10)-C(11)-C(15) | 109.36(8) |
| O(5)-C(11)-C(12) | 105.34(7) |
| C(10)-C(11)-C(12) | 123.33(8) |
| C(15)-C(11)-C(12) | 106.36(8) |
| O(4)-C(12)-C(19) | 107.12(8) |
| O(4)-C(12)-C(11) | 111.75(8) |
| C(19)-C(12)-C(11) | 113.94(8) |
| O(4)-C(12)-C(13) | 112.79(8) |
| C(19)-C(12)-C(13) | 110.36(9) |
| C(11)-C(12)-C(13) | 100.94(8) |
| C(12)-O(4)-H(4O) | 108.3(15) |
| C(12)-C(19)-H(19A) | 109.5 |
| C(12)-C(19)-H(19B) | 109.5 |
| H(19A)-C(19)-H(19B) | 109.5 |
| C(12)-C(19)-H(19C) | 109.5 |
| H(19A)-C(19)-H(19C) | 109.5 |

*Table 4. (cont'd)*

| | |
|---|---|
| H(19B)-C(19)-H(19C) | 109.5 |
| C(14)-C(13)-C(12) | 104.80(8) |
| C(14)-C(13)-H(13A) | 110.8 |
| C(12)-C(13)-H(13A) | 110.8 |
| C(14)-C(13)-H(13B) | 110.8 |
| C(12)-C(13)-H(13B) | 110.8 |
| H(13A)-C(13)-H(13B) | 108.9 |
| O(1)-C(14)-C(13) | 111.79(8) |
| O(1)-C(14)-C(15) | 106.19(8) |
| C(13)-C(14)-C(15) | 106.98(8) |
| O(1)-C(14)-H(14) | 110.6 |
| C(13)-C(14)-H(14) | 110.6 |
| C(15)-C(14)-H(14) | 110.6 |
| C(2)-C(15)-C(11) | 105.24(7) |
| C(2)-C(15)-C(14) | 105.35(7) |
| C(11)-C(15)-C(14) | 104.72(8) |
| C(2)-C(15)-H(15) | 113.5 |
| C(11)-C(15)-H(15) | 113.5 |
| C(14)-C(15)-H(15) | 113.5 |

Symmetry transformations used to generate equivalent atoms:

*Table 5. Anisotropic displacement parameters (Å$^2$x10$^3$) for Ketopyran **31**. The anisotropic displacement factor exponent takes the form: -2$\pi^2$[ $h^2a^{*2}U^{11}$ + ... + 2hka*b*$U^{12}$].*

| | $U^{11}$ | $U^{22}$ | $U^{33}$ | $U^{23}$ | $U^{13}$ | $U^{12}$ |
|---|---|---|---|---|---|---|
| O(1) | 10(1) | 18(1) | 19(1) | 1(1) | 0(1) | 3(1) |
| C(1) | 12(1) | 13(1) | 12(1) | -3(1) | -1(1) | 3(1) |
| O(2) | 19(1) | 20(1) | 16(1) | 0(1) | -3(1) | 9(1) |
| C(2) | 11(1) | 10(1) | 11(1) | -2(1) | -1(1) | 1(1) |
| C(3) | 10(1) | 9(1) | 10(1) | -1(1) | 0(1) | 0(1) |
| O(5) | 11(1) | 9(1) | 10(1) | 0(1) | -1(1) | -1(1) |
| C(4) | 13(1) | 13(1) | 10(1) | -1(1) | -1(1) | 2(1) |
| C(5) | 15(1) | 12(1) | 11(1) | -1(1) | 1(1) | 0(1) |
| C(16) | 20(1) | 12(1) | 10(1) | -1(1) | 1(1) | 0(1) |
| C(17) | 28(1) | 23(1) | 15(1) | 0(1) | -5(1) | -8(1) |
| C(18) | 26(1) | 18(1) | 14(1) | 3(1) | 1(1) | -1(1) |
| C(6) | 17(1) | 16(1) | 14(1) | -1(1) | 4(1) | 1(1) |
| C(7) | 12(1) | 12(1) | 18(1) | 0(1) | 4(1) | 0(1) |
| O(3) | 21(1) | 18(1) | 27(1) | 0(1) | 3(1) | 9(1) |
| C(8) | 10(1) | 11(1) | 15(1) | 0(1) | 1(1) | -1(1) |
| C(9) | 9(1) | 16(1) | 18(1) | 1(1) | -3(1) | -3(1) |
| C(10) | 10(1) | 13(1) | 14(1) | 0(1) | -2(1) | 1(1) |
| O(6) | 14(1) | 21(1) | 26(1) | 6(1) | -4(1) | 2(1) |
| C(11) | 9(1) | 10(1) | 10(1) | 0(1) | -2(1) | -1(1) |
| C(12) | 12(1) | 11(1) | 12(1) | 0(1) | -1(1) | -2(1) |
| O(4) | 18(1) | 11(1) | 15(1) | -1(1) | -2(1) | -2(1) |
| C(19) | 19(1) | 18(1) | 13(1) | 4(1) | -2(1) | -2(1) |
| C(13) | 10(1) | 16(1) | 16(1) | 1(1) | 0(1) | -2(1) |
| C(14) | 11(1) | 16(1) | 12(1) | 0(1) | 1(1) | 1(1) |
| C(15) | 10(1) | 11(1) | 10(1) | -2(1) | -2(1) | 0(1) |

*Table 6. Hydrogen coordinates (x10[4]) and isotropic displacement parameters (Å[2]x10[3]) for Ketopyran **31**.*

| | x | y | z | U(eq) |
|---|---|---|---|---|
| H(2) | 7285 | 4750 | 1588 | 13 |
| H(4A) | 9174 | 6316 | 2952 | 14 |
| H(4B) | 7909 | 5208 | 3147 | 14 |
| H(5) | 7440 | 7884 | 3443 | 15 |
| H(17A) | 10447 | 7253 | 4705 | 33 |
| H(17B) | 9609 | 8436 | 4311 | 33 |
| H(17C) | 10487 | 7331 | 3869 | 33 |
| H(18A) | 8487 | 5902 | 5174 | 23 |
| H(18B) | 6826 | 5543 | 4740 | 23 |
| H(6A) | 5304 | 5794 | 3801 | 19 |
| H(6B) | 5007 | 7264 | 4024 | 19 |
| H(8) | 5077 | 5194 | 2471 | 14 |
| H(9A) | 4510 | 5741 | 1352 | 17 |
| H(9B) | 3293 | 6644 | 1777 | 17 |
| H(4O) | 8170(20) | 9790(20) | 1914(10) | 22 |
| H(19A) | 6468 | 9622 | 352 | 25 |
| H(19B) | 7635 | 8529 | 47 | 25 |
| H(19C) | 8332 | 9937 | 199 | 25 |
| H(13A) | 10463 | 8753 | 798 | 16 |
| H(13B) | 10188 | 8329 | 1600 | 16 |
| H(14) | 9716 | 6805 | 374 | 15 |
| H(15) | 7139 | 6154 | 659 | 12 |

*Table 7. Torsion angles [°] for Ketopyran **31**.*

| | |
|---|---|
| C(14)-O(1)-C(1)-O(2) | -176.30(10) |
| C(14)-O(1)-C(1)-C(2) | 5.14(12) |
| O(2)-C(1)-C(2)-C(15) | -176.46(10) |
| O(1)-C(1)-C(2)-C(15) | 1.98(10) |
| O(2)-C(1)-C(2)-C(3) | 71.40(13) |
| O(1)-C(1)-C(2)-C(3) | -110.16(9) |
| C(1)-C(2)-C(3)-O(5) | 87.64(9) |
| C(15)-C(2)-C(3)-O(5) | -24.57(9) |
| C(1)-C(2)-C(3)-C(4) | -29.62(12) |
| C(15)-C(2)-C(3)-C(4) | -141.83(8) |
| C(1)-C(2)-C(3)-C(8) | -159.41(8) |
| C(15)-C(2)-C(3)-C(8) | 88.38(9) |
| C(4)-C(3)-O(5)-C(11) | 163.62(7) |
| C(8)-C(3)-O(5)-C(11) | -75.92(8) |
| C(2)-C(3)-O(5)-C(11) | 41.07(9) |
| O(5)-C(3)-C(4)-C(5) | 60.78(10) |
| C(8)-C(3)-C(4)-C(5) | -55.23(11) |
| C(2)-C(3)-C(4)-C(5) | 175.59(8) |
| C(3)-C(4)-C(5)-C(16) | -176.57(8) |
| C(3)-C(4)-C(5)-C(6) | 56.88(11) |
| C(4)-C(5)-C(16)-C(18) | -98.21(13) |
| C(6)-C(5)-C(16)-C(18) | 25.02(15) |
| C(4)-C(5)-C(16)-C(17) | 80.52(12) |
| C(6)-C(5)-C(16)-C(17) | -156.25(10) |
| C(16)-C(5)-C(6)-C(7) | 178.99(9) |
| C(4)-C(5)-C(6)-C(7) | -57.58(11) |
| C(5)-C(6)-C(7)-O(3) | -114.46(12) |
| C(5)-C(6)-C(7)-C(8) | 61.17(12) |
| O(3)-C(7)-C(8)-C(9) | -3.01(14) |
| C(6)-C(7)-C(8)-C(9) | -178.72(8) |
| O(3)-C(7)-C(8)-C(3) | 118.66(11) |
| C(6)-C(7)-C(8)-C(3) | -57.06(11) |
| O(5)-C(3)-C(8)-C(7) | -65.30(9) |
| C(4)-C(3)-C(8)-C(7) | 51.92(10) |

*Table 7. (cont'd)*

| | |
|---|---|
| C(2)-C(3)-C(8)-C(7) | -176.81(8) |
| O(5)-C(3)-C(8)-C(9) | 55.83(10) |
| C(4)-C(3)-C(8)-C(9) | 173.05(8) |
| C(2)-C(3)-C(8)-C(9) | -55.68(11) |
| C(7)-C(8)-C(9)-C(10) | 80.85(11) |
| C(3)-C(8)-C(9)-C(10) | -38.03(12) |
| C(8)-C(9)-C(10)-O(6) | -147.94(11) |
| C(8)-C(9)-C(10)-C(11) | 36.97(12) |
| C(3)-O(5)-C(11)-C(10) | 75.12(9) |
| C(3)-O(5)-C(11)-C(15) | -40.47(9) |
| C(3)-O(5)-C(11)-C(12) | -152.05(7) |
| O(6)-C(10)-C(11)-O(5) | 131.33(11) |
| C(9)-C(10)-C(11)-O(5) | -53.70(11) |
| O(6)-C(10)-C(11)-C(15) | -116.89(12) |
| C(9)-C(10)-C(11)-C(15) | 58.08(11) |
| O(6)-C(10)-C(11)-C(12) | 9.15(16) |
| C(9)-C(10)-C(11)-C(12) | -175.88(9) |
| O(5)-C(11)-C(12)-O(4) | -48.50(10) |
| C(10)-C(11)-C(12)-O(4) | 74.44(11) |
| C(15)-C(11)-C(12)-O(4) | -158.22(8) |
| O(5)-C(11)-C(12)-C(19) | -170.10(8) |
| C(10)-C(11)-C(12)-C(19) | -47.15(13) |
| C(15)-C(11)-C(12)-C(19) | 80.18(10) |
| O(5)-C(11)-C(12)-C(13) | 71.63(9) |
| C(10)-C(11)-C(12)-C(13) | -165.42(9) |
| C(15)-C(11)-C(12)-C(13) | -38.09(9) |
| O(4)-C(12)-C(13)-C(14) | 158.08(8) |
| C(19)-C(12)-C(13)-C(14) | -82.15(10) |
| C(11)-C(12)-C(13)-C(14) | 38.69(9) |
| C(1)-O(1)-C(14)-C(13) | 106.34(10) |
| C(1)-O(1)-C(14)-C(15) | -9.99(11) |
| C(12)-C(13)-C(14)-O(1) | -141.52(8) |
| C(12)-C(13)-C(14)-C(15) | -25.67(10) |
| C(1)-C(2)-C(15)-C(11) | -117.99(8) |

*Table 7. (cont'd)*

| | |
|---|---|
| C(3)-C(2)-C(15)-C(11) | 1.06(9) |
| C(1)-C(2)-C(15)-C(14) | -7.63(9) |
| C(3)-C(2)-C(15)-C(14) | 111.42(8) |
| O(5)-C(11)-C(15)-C(2) | 22.86(9) |
| C(10)-C(11)-C(15)-C(2) | -91.07(9) |
| C(12)-C(11)-C(15)-C(2) | 133.69(8) |
| O(5)-C(11)-C(15)-C(14) | -87.95(8) |
| C(10)-C(11)-C(15)-C(14) | 158.12(8) |
| C(12)-C(11)-C(15)-C(14) | 22.89(9) |
| O(1)-C(14)-C(15)-C(2) | 10.64(10) |
| C(13)-C(14)-C(15)-C(2) | -108.89(8) |
| O(1)-C(14)-C(15)-C(11) | 121.37(8) |
| C(13)-C(14)-C(15)-C(11) | 1.84(10) |

Symmetry transformations used to generate equivalent atoms:

*Table 8. Hydrogen bonds for Ketopyran* ***31*** *[Å and °].*

| D-H...A | d(D-H) | d(H...A) | d(D...A) | <(DHA) |
|---|---|---|---|---|
| C(2)-H(2)...O(3)#1 | 1.00 | 2.41 | 3.2731(13) | 143.6 |
| C(6)-H(6A)...O(6)#1 | 0.99 | 2.55 | 3.5149(15) | 163.6 |
| C(9)-H(9B)...O(1)#2 | 0.99 | 2.54 | 3.3451(13) | 138.2 |
| O(4)-H(4O)...O(2)#3 | 0.797(17) | 2.171(17) | 2.9477(12) | 164.6(19) |
| C(19)-H(19A)...O(6) | 0.98 | 2.48 | 3.0578(15) | 117.7 |
| C(14)-H(14)...O(6)#4 | 1.00 | 2.60 | 3.5664(14) | 163.4 |

Symmetry transformations used to generate equivalent atoms:

#1 -x+1,y-1/2,-z+1/2 #2 x-1,y,z #3 -x+2,y+1/2,-z+1/2

#4 x+1/2,-y+3/2,-z

## X-Ray Crystal Structure Analysis of Hemiketal S2

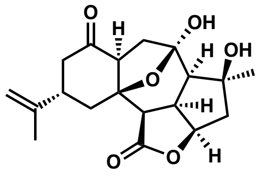


***S2***

Contents

Table 1. Experimental Details

Table 2. Crystal Data

Table 3. Atomic Coordinates

Table 4. Full Bond Distances and Angles

Table 5. Anisotropic Displacement Parameters

Table 6. Hydrogen Atomic Coordinates

Table 7. Torsion Angles

Table 8. Hydrogen Bond Distances and Angles

*X-Ray Crystal Structure of Hemiketal **S2***

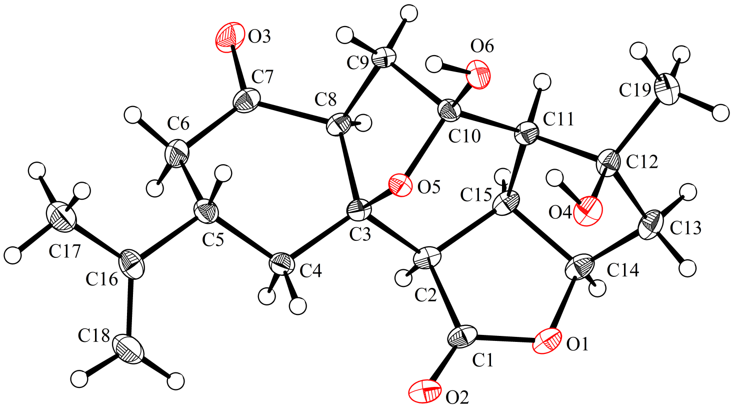

*Table 1. Experimental Details for X-Ray Structure Determination of Hemiketal* ***S2****.*

Low-temperature diffraction data ($\phi$-and $\omega$-scans) were collected on a Bruker AXS KAPPA APEX II diffractometer coupled to a APEX II CCD detector with graphite monochromated Mo $K_\alpha$ radiation ($\lambda$ = 0.71073 Å) for the structure of hemiketal **S2** and on a Bruker AXS D8 VENTURE KAPPA diffractometer coupled to a PHOTON 100 CMOS detector with Cu $K_\alpha$ radiation ($\lambda$ = 1.54178 Å) from an I$\mu$S micro-source for the structure of compound P15149 and P15156. The structure was solved by direct methods using SHELXS[i] and refined against $F^2$ on all data by full-matrix least squares with SHELXL-2014[ii] using established refinement techniques.[iii] All non-hydrogen atoms were refined anisotropically. Unless otherwise noted, all hydrogen atoms were included into the model at geometrically calculated positions and refined using a riding model. The isotropic displacement parameters of all hydrogen atoms were fixed to 1.2 times the $U$ value of the atoms they are linked to (1.5 times for methyl groups).

Hemiketal **S2** crystallizes in the orthorhombic space group $P2_12_12_1$ with one molecule in the asymmetric unit. The coordinates for all hydrogen atoms were located in the difference Fourier synthesis and refined freely.

*Table 2. Crystal Data and Structure Refinement for Hemiketal **S2**.*

| | | |
|---|---|---|
| Caltech Identification code | p15156cu | |
| CCDC Deposition Number | 1061009 | |
| Empirical formula | C19 H24 O6 | |
| Formula weight | 348.38 | |
| Temperature | 100 K | |
| Wavelength | 1.54178 Å | |
| Crystal system | Orthorhombic | |
| Space group | $P2_12_12_1$ | |
| Unit cell dimensions | a = 8.2463(2) Å | α= 90° |
| | b = 10.3683(3) Å | β= 90° |
| | c = 19.3151(5) Å | γ = 90° |
| Volume | 1651.44(8) $Å^3$ | |
| Z | 4 | |
| Density (calculated) | 1.401 $Mg/m^3$ | |
| Absorption coefficient | 0.859 $mm^{-1}$ | |
| F(000) | 744 | |
| Crystal size | 0.16 x 0.09 x 0.08 $mm^3$ | |
| Theta range for data collection | 4.578 to 79.097°. | |
| Index ranges | –10 ≤ h ≤ 10, –13 ≤ k ≤ 13, –24 ≤ l ≤ 24 | |
| Reflections collected | 40519 | |
| Independent reflections | 3557 [R(int) = 0.0399] | |
| Completeness to theta = 67.000° | 100.0 % | |
| Absorption correction | Semi-empirical from equivalents | |
| Max. and min. transmission | 1.0000 and 0.8788 | |
| Refinement method | Full-matrix least-squares on $F^2$ | |
| Data / restraints / parameters | 3557 / 0 / 322 | |
| Goodness-of-fit on $F^2$ | 1.070 | |
| Final R indices [I>2sigma(I)] | R1 = 0.0252, wR2 = 0.0607 | |
| R indices (all data) | R1 = 0.0268, wR2 = 0.0619 | |
| Absolute structure parameter | 0.03(4) | |
| Extinction coefficient | n/a | |
| Largest diff. peak and hole | 0.170 and -0.183 e.$Å^{-3}$ | |

*Table 3. Atomic coordinates (x $10^4$) and equivalent isotropic displacement parameters (Å$^2$x $10^3$) for Hemiketal* ***S2****. U(eq) is defined as one third of the trace of the orthogonalized $U^{ij}$ tensor.*

| | x | y | z | U(eq) |
|---|---|---|---|---|
| O(1) | 23254(14) | 24829(11) | 25005(6) | 192(2) |
| O(2) | 33537(15) | 14564(11) | 34069(6) | 209(3) |
| O(3) | 100576(16) | 50516(12) | 31547(7) | 248(3) |
| O(4) | 42360(16) | 11573(11) | 13350(6) | 193(2) |
| O(5) | 67107(13) | 18290(10) | 24612(5) | 128(2) |
| O(6) | 75229(14) | 16546(12) | 13315(6) | 180(2) |
| C(1) | 34786(19) | 23022(15) | 29832(8) | 161(3) |
| C(2) | 48069(19) | 33135(15) | 29051(8) | 144(3) |
| C(3) | 65329(19) | 27942(14) | 29950(7) | 133(3) |
| C(4) | 68360(20) | 21649(15) | 36942(8) | 158(3) |
| C(5) | 86350(20) | 19583(16) | 38463(8) | 165(3) |
| C(6) | 94700(20) | 32790(17) | 38913(9) | 202(3) |
| C(7) | 91720(20) | 41371(16) | 32757(8) | 168(3) |
| C(8) | 77580(20) | 38877(15) | 27971(8) | 148(3) |
| C(9) | 83759(19) | 34565(16) | 20787(8) | 164(3) |
| C(10) | 70447(19) | 25448(15) | 18354(7) | 139(3) |
| C(11) | 55282(19) | 32674(15) | 15786(8) | 140(3) |
| C(12) | 43210(20) | 24883(15) | 11226(8) | 161(3) |
| C(13) | 26650(20) | 30857(16) | 12851(9) | 194(3) |
| C(14) | 27380(20) | 35427(16) | 20304(9) | 181(3) |
| C(15) | 45172(19) | 38751(14) | 21820(8) | 146(3) |
| C(16) | 89610(20) | 11948(16) | 45052(8) | 190(3) |
| C(17) | 106770(30) | 7743(19) | 46225(10) | 250(4) |
| C(18) | 77930(30) | 8900(20) | 49571(9) | 270(4) |
| C(19) | 47170(20) | 25661(18) | 3518(9) | 218(3) |

*Table 4. Bond lengths [Å] and angles [°] for Hemiketal **S2**.*

| | |
|---|---|
| O(1)-C(1) | 1.345(2) |
| O(1)-C(14) | 1.466(2) |
| O(2)-C(1) | 1.204(2) |
| O(3)-C(7) | 1.220(2) |
| O(4)-H(4) | 0.86(3) |
| O(4)-C(12) | 1.4414(18) |
| O(5)-C(3) | 1.4443(17) |
| O(5)-C(10) | 1.4448(18) |
| O(6)-H(6) | 0.88(3) |
| O(6)-C(10) | 1.3982(18) |
| C(1)-C(2) | 1.524(2) |
| C(2)-H(2) | 0.97(2) |
| C(2)-C(3) | 1.532(2) |
| C(2)-C(15) | 1.532(2) |
| C(3)-C(4) | 1.520(2) |
| C(3)-C(8) | 1.566(2) |
| C(4)-H(4A) | 0.98(2) |
| C(4)-H(4B) | 0.97(2) |
| C(4)-C(5) | 1.528(2) |
| C(5)-H(5) | 1.00(2) |
| C(5)-C(6) | 1.535(2) |
| C(5)-C(16) | 1.523(2) |
| C(6)-H(6A) | 0.99(3) |
| C(6)-H(6B) | 0.96(3) |
| C(6)-C(7) | 1.505(2) |
| C(7)-C(8) | 1.510(2) |
| C(8)-H(8) | 1.01(2) |
| C(8)-C(9) | 1.544(2) |
| C(9)-H(9A) | 0.94(2) |
| C(9)-H(9B) | 0.96(2) |
| C(9)-C(10) | 1.523(2) |
| C(10)-C(11) | 1.540(2) |
| C(11)-H(11) | 0.97(2) |

*Table 4. (cont'd)*

| | |
|---|---|
| C(11)-C(12) | 1.556(2) |
| C(11)-C(15) | 1.565(2) |
| C(12)-C(13) | 1.531(2) |
| C(12)-C(19) | 1.526(2) |
| C(13)-H(13A) | 1.00(2) |
| C(13)-H(13B) | 1.01(3) |
| C(13)-C(14) | 1.517(2) |
| C(14)-H(14) | 0.99(2) |
| C(14)-C(15) | 1.535(2) |
| C(15)-H(15) | 0.98(2) |
| C(16)-C(17) | 1.497(3) |
| C(16)-C(18) | 1.338(3) |
| C(17)-H(17A) | 0.98(3) |
| C(17)-H(17B) | 1.00(3) |
| C(17)-H(17C) | 0.99(3) |
| C(18)-H(18A) | 0.97(3) |
| C(18)-H(18B) | 0.99(3) |
| C(19)-H(19A) | 1.00(3) |
| C(19)-H(19B) | 0.97(2) |
| C(19)-H(19C) | 1.00(3) |
| | |
| C(1)-O(1)-C(14) | 111.70(12) |
| C(12)-O(4)-H(4) | 103.7(18) |
| C(3)-O(5)-C(10) | 105.11(11) |
| C(10)-O(6)-H(6) | 109.4(16) |
| O(1)-C(1)-C(2) | 110.10(13) |
| O(2)-C(1)-O(1) | 120.81(14) |
| O(2)-C(1)-C(2) | 129.06(15) |
| C(1)-C(2)-H(2) | 105.4(13) |
| C(1)-C(2)-C(3) | 114.51(12) |
| C(1)-C(2)-C(15) | 103.87(13) |
| C(3)-C(2)-H(2) | 109.7(13) |
| C(3)-C(2)-C(15) | 112.45(12) |
| C(15)-C(2)-H(2) | 110.5(12) |

*Table 4. (cont'd)*

| | |
|---|---|
| O(5)-C(3)-C(2) | 104.89(12) |
| O(5)-C(3)-C(4) | 108.66(12) |
| O(5)-C(3)-C(8) | 105.18(11) |
| C(2)-C(3)-C(8) | 108.50(12) |
| C(4)-C(3)-C(2) | 113.85(13) |
| C(4)-C(3)-C(8) | 114.93(13) |
| C(3)-C(4)-H(4A) | 109.1(13) |
| C(3)-C(4)-H(4B) | 107.6(13) |
| C(3)-C(4)-C(5) | 112.99(13) |
| H(4A)-C(4)-H(4B) | 105.8(18) |
| C(5)-C(4)-H(4A) | 110.7(13) |
| C(5)-C(4)-H(4B) | 110.3(13) |
| C(4)-C(5)-H(5) | 110.4(12) |
| C(4)-C(5)-C(6) | 108.74(13) |
| C(6)-C(5)-H(5) | 107.1(12) |
| C(16)-C(5)-C(4) | 113.91(13) |
| C(16)-C(5)-H(5) | 106.8(12) |
| C(16)-C(5)-C(6) | 109.71(13) |
| C(5)-C(6)-H(6A) | 110.0(15) |
| C(5)-C(6)-H(6B) | 112.6(14) |
| H(6A)-C(6)-H(6B) | 108(2) |
| C(7)-C(6)-C(5) | 114.17(13) |
| C(7)-C(6)-H(6A) | 105.2(14) |
| C(7)-C(6)-H(6B) | 106.8(14) |
| O(3)-C(7)-C(6) | 120.88(15) |
| O(3)-C(7)-C(8) | 118.57(15) |
| C(6)-C(7)-C(8) | 120.55(14) |
| C(3)-C(8)-H(8) | 112.3(13) |
| C(7)-C(8)-C(3) | 118.21(13) |
| C(7)-C(8)-H(8) | 103.8(12) |
| C(7)-C(8)-C(9) | 110.18(13) |
| C(9)-C(8)-C(3) | 102.86(12) |
| C(9)-C(8)-H(8) | 109.4(12) |
| C(8)-C(9)-H(9A) | 113.1(13) |

*Table 4. (cont'd)*

| | |
|---|---|
| C(8)-C(9)-H(9B) | 109.8(12) |
| H(9A)-C(9)-H(9B) | 111.1(18) |
| C(10)-C(9)-C(8) | 102.66(12) |
| C(10)-C(9)-H(9A) | 112.2(13) |
| C(10)-C(9)-H(9B) | 107.6(12) |
| O(5)-C(10)-C(9) | 101.42(11) |
| O(5)-C(10)-C(11) | 111.38(12) |
| O(6)-C(10)-O(5) | 107.28(12) |
| O(6)-C(10)-C(9) | 114.91(13) |
| O(6)-C(10)-C(11) | 109.02(12) |
| C(9)-C(10)-C(11) | 112.51(13) |
| C(10)-C(11)-H(11) | 106.9(12) |
| C(10)-C(11)-C(12) | 116.72(13) |
| C(10)-C(11)-C(15) | 112.86(12) |
| C(12)-C(11)-H(11) | 106.0(12) |
| C(12)-C(11)-C(15) | 106.84(12) |
| C(15)-C(11)-H(11) | 106.9(12) |
| O(4)-C(12)-C(11) | 111.53(12) |
| O(4)-C(12)-C(13) | 106.61(13) |
| O(4)-C(12)-C(19) | 109.78(13) |
| C(13)-C(12)-C(11) | 104.15(12) |
| C(19)-C(12)-C(11) | 112.81(13) |
| C(19)-C(12)-C(13) | 111.68(14) |
| C(12)-C(13)-H(13A) | 109.2(12) |
| C(12)-C(13)-H(13B) | 112.6(14) |
| H(13A)-C(13)-H(13B) | 107.3(18) |
| C(14)-C(13)-C(12) | 106.59(13) |
| C(14)-C(13)-H(13A) | 109.6(12) |
| C(14)-C(13)-H(13B) | 111.5(14) |
| O(1)-C(14)-C(13) | 110.16(13) |
| O(1)-C(14)-H(14) | 106.1(12) |
| O(1)-C(14)-C(15) | 105.79(13) |
| C(13)-C(14)-H(14) | 113.8(12) |
| C(13)-C(14)-C(15) | 106.81(13) |

*Table 4. (cont'd)*

| | |
|---|---|
| C(15)-C(14)-H(14) | 113.8(12) |
| C(2)-C(15)-C(11) | 116.28(12) |
| C(2)-C(15)-C(14) | 103.75(13) |
| C(2)-C(15)-H(15) | 110.5(13) |
| C(11)-C(15)-H(15) | 109.8(13) |
| C(14)-C(15)-C(11) | 106.05(13) |
| C(14)-C(15)-H(15) | 110.1(13) |
| C(17)-C(16)-C(5) | 116.41(14) |
| C(18)-C(16)-C(5) | 122.73(16) |
| C(18)-C(16)-C(17) | 120.85(16) |
| C(16)-C(17)-H(17A) | 110.5(17) |
| C(16)-C(17)-H(17B) | 111.6(16) |
| C(16)-C(17)-H(17C) | 113.0(17) |
| H(17A)-C(17)-H(17B) | 108(2) |
| H(17A)-C(17)-H(17C) | 107(2) |
| H(17B)-C(17)-H(17C) | 106(2) |
| C(16)-C(18)-H(18A) | 118.5(17) |
| C(16)-C(18)-H(18B) | 121.4(15) |
| H(18A)-C(18)-H(18B) | 120(2) |
| C(12)-C(19)-H(19A) | 110.0(13) |
| C(12)-C(19)-H(19B) | 112.3(13) |
| C(12)-C(19)-H(19C) | 108.5(14) |
| H(19A)-C(19)-H(19B) | 107(2) |
| H(19A)-C(19)-H(19C) | 110.7(19) |
| H(19B)-C(19)-H(19C) | 108(2) |

Symmetry transformations used to generate equivalent atoms:

*Table 5. Anisotropic displacement parameters ($Å^2x10^3$) for Hemiketal **S2**. The anisotropic displacement factor exponent takes the form: $-2\pi^2[ h^2a^{*2}U^{11} + ... + 2hka^*b^*U^{12}]$.*

| | $U^{11}$ | $U^{22}$ | $U^{33}$ | $U^{23}$ | $U^{13}$ | $U^{12}$ |
|---|---|---|---|---|---|---|
| O(1) | 128(5) | 177(5) | 271(6) | 6(5) | 5(4) | -21(4) |
| O(2) | 179(6) | 197(6) | 252(6) | 19(5) | 52(5) | -36(5) |
| O(3) | 222(6) | 220(6) | 303(6) | 33(5) | -55(5) | -112(5) |
| O(4) | 199(6) | 118(5) | 262(6) | 16(4) | -21(5) | 1(5) |
| O(5) | 152(5) | 113(5) | 118(4) | -14(4) | 9(4) | 2(4) |
| O(6) | 166(5) | 232(6) | 140(5) | -24(4) | 2(4) | 80(5) |
| C(1) | 127(7) | 149(7) | 207(7) | -50(6) | 50(6) | -1(6) |
| C(2) | 135(7) | 116(7) | 181(7) | -30(6) | 35(6) | -11(6) |
| C(3) | 136(7) | 125(6) | 138(6) | -33(5) | 18(5) | -20(6) |
| C(4) | 172(7) | 169(7) | 134(6) | -10(6) | 17(6) | -33(6) |
| C(5) | 190(7) | 183(7) | 123(6) | -9(6) | 4(6) | -19(6) |
| C(6) | 199(8) | 226(8) | 183(7) | -1(6) | -43(6) | -58(7) |
| C(7) | 146(7) | 161(7) | 197(7) | -35(6) | 14(6) | -22(6) |
| C(8) | 130(7) | 136(7) | 177(7) | -3(6) | 0(6) | -30(6) |
| C(9) | 122(7) | 212(8) | 159(7) | 38(6) | 13(6) | -24(7) |
| C(10) | 134(7) | 158(7) | 124(6) | 9(6) | 15(5) | 29(6) |
| C(11) | 125(7) | 121(7) | 174(7) | 17(6) | 1(6) | 15(6) |
| C(12) | 156(7) | 126(7) | 201(7) | 19(6) | -31(6) | 15(6) |
| C(13) | 138(7) | 177(7) | 266(8) | 15(7) | -45(6) | 10(6) |
| C(14) | 122(7) | 144(7) | 277(8) | 21(6) | 9(6) | 23(6) |
| C(15) | 109(7) | 100(6) | 230(7) | -7(6) | 7(6) | 20(6) |
| C(16) | 256(9) | 179(7) | 134(7) | -28(6) | -16(6) | 0(7) |
| C(17) | 276(9) | 280(9) | 193(8) | 38(7) | 11(7) | 41(8) |
| C(18) | 308(10) | 323(10) | 178(8) | 56(7) | 30(7) | 22(8) |
| C(19) | 241(9) | 227(8) | 186(7) | 18(7) | -40(7) | 21(7) |

*Table 6. Hydrogen coordinates (x10[4]) and isotropic displacement parameters (Å[2]x10[3]) for Hemiketal **S2**.*

| | x | y | z | U(eq) |
|---|---|---|---|---|
| H(4) | 5230(40) | 900(30) | 1322(13) | 38(7) |
| H(6) | 8360(30) | 1210(20) | 1486(12) | 34(6) |
| H(2) | 4580(30) | 3950(20) | 3257(11) | 19(5) |
| H(4A) | 6250(30) | 1350(20) | 3715(11) | 24(5) |
| H(4B) | 6350(30) | 2710(20) | 4045(11) | 21(5) |
| H(5) | 9160(30) | 1480(20) | 3459(10) | 19(5) |
| H(6A) | 9050(30) | 3760(20) | 4292(13) | 36(6) |
| H(6B) | 10620(30) | 3210(20) | 3943(12) | 28(6) |
| H(8) | 7220(30) | 4750(20) | 2755(11) | 19(5) |
| H(9A) | 8530(30) | 4150(20) | 1771(11) | 21(5) |
| H(9B) | 9350(30) | 2960(19) | 2130(10) | 14(4) |
| H(11) | 5910(30) | 3980(20) | 1292(10) | 17(5) |
| H(13A) | 2480(30) | 3840(20) | 972(11) | 18(5) |
| H(13B) | 1740(30) | 2460(20) | 1210(12) | 33(6) |
| H(14) | 1970(30) | 4250(20) | 2137(10) | 16(5) |
| H(15) | 4670(30) | 4810(20) | 2186(11) | 22(5) |
| H(17A) | 11020(30) | 170(30) | 4263(14) | 43(7) |
| H(17B) | 10810(30) | 340(20) | 5083(13) | 37(7) |
| H(17C) | 11460(40) | 1500(30) | 4620(14) | 48(8) |
| H(18A) | 8080(30) | 390(30) | 5363(14) | 39(7) |
| H(18B) | 6650(30) | 1110(30) | 4869(13) | 37(7) |
| H(19A) | 5790(30) | 2140(20) | 258(12) | 32(6) |
| H(19B) | 4800(30) | 3450(20) | 190(12) | 27(6) |
| H(19C) | 3830(30) | 2130(20) | 88(12) | 29(6) |

*Table 7. Torsion angles [°] for Hemiketal **S2**.*

| | |
|---|---|
| O(1)-C(1)-C(2)-C(3) | -138.72(13) |
| O(1)-C(1)-C(2)-C(15) | -15.70(16) |
| O(1)-C(14)-C(15)-C(2) | -20.00(15) |
| O(1)-C(14)-C(15)-C(11) | 103.00(14) |
| O(2)-C(1)-C(2)-C(3) | 43.2(2) |
| O(2)-C(1)-C(2)-C(15) | 166.23(16) |
| O(3)-C(7)-C(8)-C(3) | -174.28(15) |
| O(3)-C(7)-C(8)-C(9) | 67.94(19) |
| O(4)-C(12)-C(13)-C(14) | 86.84(15) |
| O(5)-C(3)-C(4)-C(5) | 77.86(15) |
| O(5)-C(3)-C(8)-C(7) | -114.56(14) |
| O(5)-C(3)-C(8)-C(9) | 7.03(15) |
| O(5)-C(10)-C(11)-C(12) | -84.55(15) |
| O(5)-C(10)-C(11)-C(15) | 39.79(17) |
| O(6)-C(10)-C(11)-C(12) | 33.65(18) |
| O(6)-C(10)-C(11)-C(15) | 157.99(12) |
| C(1)-O(1)-C(14)-C(13) | 126.20(14) |
| C(1)-O(1)-C(14)-C(15) | 11.11(16) |
| C(1)-C(2)-C(3)-O(5) | 60.00(15) |
| C(1)-C(2)-C(3)-C(4) | -58.66(17) |
| C(1)-C(2)-C(3)-C(8) | 172.01(12) |
| C(1)-C(2)-C(15)-C(11) | -94.84(15) |
| C(1)-C(2)-C(15)-C(14) | 21.15(15) |
| C(2)-C(3)-C(4)-C(5) | -165.66(13) |
| C(2)-C(3)-C(8)-C(7) | 133.63(14) |
| C(2)-C(3)-C(8)-C(9) | -104.78(13) |
| C(3)-O(5)-C(10)-O(6) | 169.22(12) |
| C(3)-O(5)-C(10)-C(9) | 48.36(14) |
| C(3)-O(5)-C(10)-C(11) | -71.54(14) |
| C(3)-C(2)-C(15)-C(11) | 29.52(18) |
| C(3)-C(2)-C(15)-C(14) | 145.52(12) |
| C(3)-C(4)-C(5)-C(6) | 63.83(16) |
| C(3)-C(4)-C(5)-C(16) | -173.48(13) |
| C(3)-C(8)-C(9)-C(10) | 21.03(15) |

*Table 7. (cont'd)*

| | |
|---|---|
| C(4)-C(3)-C(8)-C(7) | 4.9(2) |
| C(4)-C(3)-C(8)-C(9) | 126.49(13) |
| C(4)-C(5)-C(6)-C(7) | -52.59(18) |
| C(4)-C(5)-C(16)-C(17) | 170.32(14) |
| C(4)-C(5)-C(16)-C(18) | -8.8(2) |
| C(5)-C(6)-C(7)-O(3) | -161.18(16) |
| C(5)-C(6)-C(7)-C(8) | 19.3(2) |
| C(6)-C(5)-C(16)-C(17) | -67.52(19) |
| C(6)-C(5)-C(16)-C(18) | 113.37(19) |
| C(6)-C(7)-C(8)-C(3) | 5.3(2) |
| C(6)-C(7)-C(8)-C(9) | -112.51(17) |
| C(7)-C(8)-C(9)-C(10) | 147.92(13) |
| C(8)-C(3)-C(4)-C(5) | -39.64(18) |
| C(8)-C(9)-C(10)-O(5) | -42.13(14) |
| C(8)-C(9)-C(10)-O(6) | -157.47(12) |
| C(8)-C(9)-C(10)-C(11) | 76.97(15) |
| C(9)-C(10)-C(11)-C(12) | 162.34(13) |
| C(9)-C(10)-C(11)-C(15) | -73.31(16) |
| C(10)-O(5)-C(3)-C(2) | 79.80(13) |
| C(10)-O(5)-C(3)-C(4) | -158.10(12) |
| C(10)-O(5)-C(3)-C(8) | -34.56(14) |
| C(10)-C(11)-C(12)-O(4) | 34.69(18) |
| C(10)-C(11)-C(12)-C(13) | 149.29(13) |
| C(10)-C(11)-C(12)-C(19) | -89.42(16) |
| C(10)-C(11)-C(15)-C(2) | -19.82(19) |
| C(10)-C(11)-C(15)-C(14) | -134.52(14) |
| C(11)-C(12)-C(13)-C(14) | -31.21(16) |
| C(12)-C(11)-C(15)-C(2) | 109.78(14) |
| C(12)-C(11)-C(15)-C(14) | -4.92(15) |
| C(12)-C(13)-C(14)-O(1) | -85.69(15) |
| C(12)-C(13)-C(14)-C(15) | 28.74(16) |
| C(13)-C(14)-C(15)-C(2) | -137.36(13) |
| C(13)-C(14)-C(15)-C(11) | -14.36(16) |
| C(14)-O(1)-C(1)-O(2) | -178.78(14) |

*Table 7. (cont'd)*

| | |
|---|---|
| C(14)-O(1)-C(1)-C(2) | 2.96(17) |
| C(15)-C(2)-C(3)-O(5) | -58.27(15) |
| C(15)-C(2)-C(3)-C(4) | -176.92(12) |
| C(15)-C(2)-C(3)-C(8) | 53.75(16) |
| C(15)-C(11)-C(12)-O(4) | -92.67(15) |
| C(15)-C(11)-C(12)-C(13) | 21.93(15) |
| C(15)-C(11)-C(12)-C(19) | 143.23(13) |
| C(16)-C(5)-C(6)-C(7) | -177.78(14) |
| C(19)-C(12)-C(13)-C(14) | -153.25(14) |

Symmetry transformations used to generate equivalent atoms:

*Table 8. Hydrogen bonds for Hemiketal **S2** [Å and °].*

| D-H...A | d(D-H) | d(H...A) | d(D...A) | <(DHA) |
|---|---|---|---|---|
| O(4)-H(4)...O(6) | 0.86(3) | 2.04(3) | 2.7591(18) | 140(2) |
| O(6)-H(6)...O(3)#1 | 0.88(3) | 1.90(3) | 2.7799(17) | 172(2) |
| C(2)-H(2)...O(4)#2 | 0.97(2) | 2.61(2) | 3.3870(19) | 138.0(16) |
| C(4)-H(4A)...O(2) | 0.98(2) | 2.46(2) | 3.015(2) | 115.7(16) |
| C(8)-H(8)...O(4)#2 | 1.01(2) | 2.58(2) | 3.324(2) | 130.9(15) |
| C(9)-H(9B)...O(1)#3 | 0.96(2) | 2.60(2) | 3.506(2) | 156.9(16) |
| C(15)-H(15)...O(2)#2 | 0.98(2) | 2.62(2) | 3.397(2) | 136.0(17) |
| C(15)-H(15)...O(5)#2 | 0.98(2) | 2.48(2) | 3.2986(18) | 141.5(17) |
| C(19)-H(19A)...O(6) | 1.00(3) | 2.57(2) | 3.135(2) | 115.5(16) |

Symmetry transformations used to generate equivalent atoms:

#1 -x+2,y-1/2,-z+1/2 #2 -x+1,y+1/2,-z+1/2 #3 x+1,y,z

## X-Ray Crystal Structure Analysis of Epoxide 36

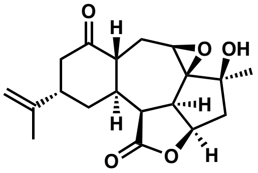


***36***

Contents

Table 1. Experimental Details

Table 2. Crystal Data

Table 3. Atomic Coordinates

Table 4. Full Bond Distances and Angles

Table 5. Anisotropic Displacement Parameters

Table 6. Hydrogen Atomic Coordinates

Table 7. Torsion Angles

Table 8. Hydrogen Bond Distances and Angle

*X-Ray Crystal Structure of Epoxide **36***

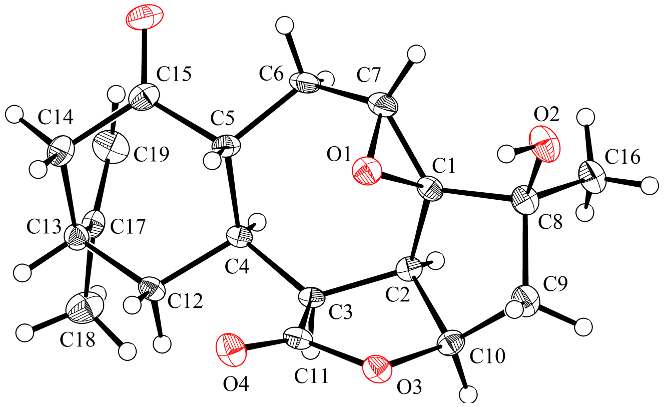


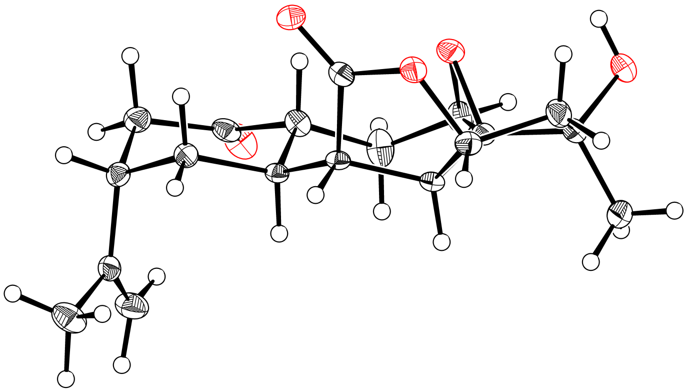

*Table 1. Experimental Details for X-Ray Structure Determination of Epoxide **36**.*

Low-temperature diffraction data ($\phi$-and $\omega$-scans) were collected on a Bruker AXS KAPPA APEX II diffractometer coupled to a APEX II CCD detector with graphite monochromated Mo $K_\alpha$ radiation ($\lambda$ = 0.71073 Å) for the structure of epoxide **36** and on a Bruker AXS D8 VENTURE KAPPA diffractometer coupled to a PHOTON 100 CMOS detector with Cu $K_\alpha$ radiation ($\lambda$ = 1.54178 Å) from an I$\mu$S micro-source for the structure of compound P15149 and P15156. The structure was solved by direct methods using SHELXS[i] and refined against $F^2$ on all data by full-matrix least squares with SHELXL-2014[ii] using established refinement techniques.[iii] All non-hydrogen atoms were refined anisotropically. Unless otherwise noted, all hydrogen atoms were included into the model at geometrically calculated positions and refined using a riding model. The isotropic displacement parameters of all hydrogen atoms were fixed to 1.2 times the $U$ value of the atoms they are linked to (1.5 times for methyl groups).

Epoxide **36** crystallizes in the orthorhombic space group $P2_12_12_1$ with one molecule in the asymmetric unit. The coordinates for all hydrogen atoms were located in the difference Fourier synthesis and refined freely.

*Table 2. Crystal Data and Structure Refinement for Epoxide* ***36***.

| | | |
|---|---|---|
| Caltech Identification code | a14286 | |
| CCDC Deposition Number | 1061014 | |
| Empirical formula | C19 H24 O5 | |
| Formula weight | 332.38 | |
| Temperature | 100 K | |
| Wavelength | 0.71073 Å | |
| Crystal system | Orthorhombic | |
| Space group | P 21 21 21 | |
| Unit cell dimensions | a = 6.2703(9) Å | $\alpha$= 90° |
| | b = 11.2931(16) Å | $\beta$= 90° |
| | c = 23.177(4) Å | $\gamma$ = 90° |
| Volume | 1641.2(4) Å$^3$ | |
| Z | 4 | |
| Density (calculated) | 1.345 Mg/m$^3$ | |
| Absorption coefficient | 0.097 mm$^{-1}$ | |
| F(000) | 712 | |
| Crystal size | 0.57 x 0.25 x 0.03 mm$^3$ | |
| Theta range for data collection | 1.757 to 33.933°. | |
| Index ranges | $-9 \le h \le 9, -17 \le k \le 17, -36 \le l \le 35$ | |
| Reflections collected | 42176 | |
| Independent reflections | 6378 [R(int) = 0.0463] | |
| Completeness to theta = 25.000° | 100.0 % | |
| Absorption correction | Semi-empirical from equivalents | |
| Max. and min. transmission | 1.0000 and 0.8611 | |
| Refinement method | Full-matrix least-squares on F$^2$ | |
| Data / restraints / parameters | 6378 / 0 / 313 | |
| Goodness-of-fit on F$^2$ | 1.447 | |
| Final R indices [I>2sigma(I)] | R1 = 0.0413, wR2 = 0.0809 | |
| R indices (all data) | R1 = 0.0538, wR2 = 0.0831 | |
| Absolute structure parameter | -0.6(2) | |
| Extinction coefficient | n/a | |
| Largest diff. peak and hole | 0.392 and -0.199 e.Å$^{-3}$ | |

*Table 3. Atomic coordinates (x $10^4$) and equivalent isotropic displacement parameters ($Å^2$x $10^3$) for Epoxide* ***36****. U(eq) is defined as one third of the trace of the orthogonalized $U^{ij}$ tensor.*

| | x | y | z | U(eq) |
|---|---|---|---|---|
| O(1) | 60147(17) | 38434(9) | 46552(5) | 183(2) |
| O(2) | 76160(20) | 34388(10) | 35657(5) | 243(3) |
| O(3) | 43551(17) | 62722(10) | 44133(4) | 179(2) |
| O(4) | 26855(17) | 58723(10) | 52373(5) | 212(2) |
| O(5) | 82780(20) | 28834(10) | 65931(5) | 252(3) |
| C(1) | 78760(20) | 45191(12) | 44797(6) | 146(3) |
| C(2) | 78900(20) | 57950(12) | 46746(6) | 134(2) |
| C(3) | 66120(20) | 60975(12) | 52169(5) | 121(2) |
| C(4) | 71270(20) | 55487(12) | 58083(6) | 124(2) |
| C(5) | 69870(20) | 41806(12) | 58441(6) | 161(3) |
| C(6) | 86020(30) | 35128(14) | 54757(7) | 229(3) |
| C(7) | 81550(30) | 34815(13) | 48377(6) | 202(3) |
| C(8) | 81980(20) | 45267(13) | 38266(6) | 175(3) |
| C(9) | 67800(30) | 55912(15) | 36501(6) | 203(3) |
| C(10) | 65480(20) | 63631(13) | 41907(6) | 161(3) |
| C(11) | 43420(20) | 60300(12) | 49886(6) | 153(3) |
| C(12) | 57290(20) | 61462(13) | 62695(6) | 153(3) |
| C(13) | 62610(20) | 57484(13) | 68893(6) | 156(3) |
| C(14) | 59610(30) | 44006(14) | 69216(6) | 192(3) |
| C(15) | 72060(30) | 37435(13) | 64686(6) | 179(3) |
| C(16) | 105320(30) | 47240(15) | 36731(7) | 214(3) |
| C(17) | 84150(20) | 61913(14) | 70905(6) | 161(3) |
| C(18) | 85030(30) | 74981(15) | 72048(8) | 263(3) |
| C(19) | 101020(30) | 55096(17) | 71833(8) | 247(3) |

*Table 4. Bond lengths [Å] and angles [°] for Epoxide **36**.*

| | |
|---|---|
| O(1)-C(1) | 1.4523(18) |
| O(1)-C(7) | 1.465(2) |
| O(2)-H(2) | 0.87(3) |
| O(2)-C(8) | 1.4172(18) |
| O(3)-C(10) | 1.4725(18) |
| O(3)-C(11) | 1.3611(17) |
| O(4)-C(11) | 1.2015(18) |
| O(5)-C(15) | 1.2160(19) |
| C(1)-C(2) | 1.5101(19) |
| C(1)-C(7) | 1.446(2) |
| C(1)-C(8) | 1.527(2) |
| C(2)-H(2A) | 0.97(2) |
| C(2)-C(3) | 1.5290(19) |
| C(2)-C(10) | 1.542(2) |
| C(3)-H(3) | 0.970(17) |
| C(3)-C(4) | 1.5385(19) |
| C(3)-C(11) | 1.5205(19) |
| C(4)-H(4) | 0.987(18) |
| C(4)-C(5) | 1.5497(19) |
| C(4)-C(12) | 1.5383(19) |
| C(5)-H(5) | 1.010(19) |
| C(5)-C(6) | 1.524(2) |
| C(5)-C(15) | 1.5356(19) |
| C(6)-H(6A) | 1.00(2) |
| C(6)-H(6B) | 0.944(19) |
| C(6)-C(7) | 1.505(2) |
| C(7)-H(7) | 0.990(18) |
| C(8)-C(9) | 1.550(2) |
| C(8)-C(16) | 1.522(2) |
| C(9)-H(9A) | 0.970(19) |
| C(9)-H(9B) | 0.95(2) |
| C(9)-C(10) | 1.533(2) |
| C(10)-H(10) | 0.952(18) |

*Table 4. (cont'd)*

| | |
|---|---|
| C(12)-H(12A) | 0.961(19) |
| C(12)-H(12B) | 0.97(2) |
| C(12)-C(13) | 1.542(2) |
| C(13)-H(13) | 0.981(18) |
| C(13)-C(14) | 1.536(2) |
| C(13)-C(17) | 1.514(2) |
| C(14)-H(14A) | 0.991(18) |
| C(14)-H(14B) | 1.05(2) |
| C(14)-C(15) | 1.504(2) |
| C(16)-H(16A) | 0.96(2) |
| C(16)-H(16B) | 0.98(2) |
| C(16)-H(16C) | 0.93(2) |
| C(17)-C(18) | 1.500(2) |
| C(17)-C(19) | 1.326(2) |
| C(18)-H(18A) | 0.96(2) |
| C(18)-H(18B) | 1.00(2) |
| C(18)-H(18C) | 0.99(2) |
| C(19)-H(19A) | 0.95(2) |
| C(19)-H(19B) | 0.93(2) |
| | |
| C(1)-O(1)-C(7) | 59.43(9) |
| C(8)-O(2)-H(2) | 109.1(16) |
| C(11)-O(3)-C(10) | 111.27(11) |
| O(1)-C(1)-C(2) | 114.97(11) |
| O(1)-C(1)-C(8) | 112.77(12) |
| C(2)-C(1)-C(8) | 106.88(11) |
| C(7)-C(1)-O(1) | 60.74(10) |
| C(7)-C(1)-C(2) | 126.92(12) |
| C(7)-C(1)-C(8) | 123.85(13) |
| C(1)-C(2)-H(2A) | 108.7(13) |
| C(1)-C(2)-C(3) | 117.13(11) |
| C(1)-C(2)-C(10) | 100.15(11) |
| C(3)-C(2)-H(2A) | 112.2(13) |
| C(3)-C(2)-C(10) | 102.68(11) |

*Table 4. (cont'd)*

| | |
|---|---|
| C(10)-C(2)-H(2A) | 115.6(13) |
| C(2)-C(3)-H(3) | 106.1(10) |
| C(2)-C(3)-C(4) | 122.18(11) |
| C(4)-C(3)-H(3) | 105.1(10) |
| C(11)-C(3)-C(2) | 101.12(11) |
| C(11)-C(3)-H(3) | 100.8(10) |
| C(11)-C(3)-C(4) | 119.10(12) |
| C(3)-C(4)-H(4) | 107.0(9) |
| C(3)-C(4)-C(5) | 115.93(11) |
| C(5)-C(4)-H(4) | 106.2(10) |
| C(12)-C(4)-C(3) | 108.83(11) |
| C(12)-C(4)-H(4) | 106.7(9) |
| C(12)-C(4)-C(5) | 111.58(12) |
| C(4)-C(5)-H(5) | 106.1(10) |
| C(6)-C(5)-C(4) | 115.19(13) |
| C(6)-C(5)-H(5) | 110.2(10) |
| C(6)-C(5)-C(15) | 108.03(12) |
| C(15)-C(5)-C(4) | 111.45(11) |
| C(15)-C(5)-H(5) | 105.4(10) |
| C(5)-C(6)-H(6A) | 108.0(11) |
| C(5)-C(6)-H(6B) | 107.6(12) |
| H(6A)-C(6)-H(6B) | 107.5(16) |
| C(7)-C(6)-C(5) | 115.99(14) |
| C(7)-C(6)-H(6A) | 109.5(11) |
| C(7)-C(6)-H(6B) | 107.8(12) |
| O(1)-C(7)-C(6) | 116.57(13) |
| O(1)-C(7)-H(7) | 109.1(10) |
| C(1)-C(7)-O(1) | 59.83(9) |
| C(1)-C(7)-C(6) | 124.54(13) |
| C(1)-C(7)-H(7) | 115.0(10) |
| C(6)-C(7)-H(7) | 117.0(10) |
| O(2)-C(8)-C(1) | 112.57(12) |
| O(2)-C(8)-C(9) | 114.32(13) |
| O(2)-C(8)-C(16) | 105.96(13) |

*Table 4. (cont'd)*

| | |
|---|---|
| C(1)-C(8)-C(9) | 100.95(11) |
| C(16)-C(8)-C(1) | 111.10(12) |
| C(16)-C(8)-C(9) | 112.09(13) |
| C(8)-C(9)-H(9A) | 111.3(11) |
| C(8)-C(9)-H(9B) | 110.2(13) |
| H(9A)-C(9)-H(9B) | 105.8(17) |
| C(10)-C(9)-C(8) | 106.24(11) |
| C(10)-C(9)-H(9A) | 113.4(11) |
| C(10)-C(9)-H(9B) | 110.0(13) |
| O(3)-C(10)-C(2) | 103.03(11) |
| O(3)-C(10)-C(9) | 109.59(12) |
| O(3)-C(10)-H(10) | 107.0(10) |
| C(2)-C(10)-H(10) | 114.8(10) |
| C(9)-C(10)-C(2) | 107.82(12) |
| C(9)-C(10)-H(10) | 113.9(10) |
| O(3)-C(11)-C(3) | 109.00(12) |
| O(4)-C(11)-O(3) | 120.33(13) |
| O(4)-C(11)-C(3) | 130.53(13) |
| C(4)-C(12)-H(12A) | 109.4(11) |
| C(4)-C(12)-H(12B) | 109.7(11) |
| C(4)-C(12)-C(13) | 113.34(12) |
| H(12A)-C(12)-H(12B) | 105.9(16) |
| C(13)-C(12)-H(12A) | 109.8(11) |
| C(13)-C(12)-H(12B) | 108.5(11) |
| C(12)-C(13)-H(13) | 107.1(10) |
| C(14)-C(13)-C(12) | 107.93(12) |
| C(14)-C(13)-H(13) | 106.9(10) |
| C(17)-C(13)-C(12) | 112.57(12) |
| C(17)-C(13)-H(13) | 106.9(11) |
| C(17)-C(13)-C(14) | 114.95(13) |
| C(13)-C(14)-H(14A) | 110.5(11) |
| C(13)-C(14)-H(14B) | 107.1(12) |
| H(14A)-C(14)-H(14B) | 108.6(16) |
| C(15)-C(14)-C(13) | 113.04(12) |

*Table 4. (cont'd)*

| | |
|---|---|
| C(15)-C(14)-H(14A) | 110.0(11) |
| C(15)-C(14)-H(14B) | 107.4(12) |
| O(5)-C(15)-C(5) | 122.00(13) |
| O(5)-C(15)-C(14) | 121.02(13) |
| C(14)-C(15)-C(5) | 116.94(13) |
| C(8)-C(16)-H(16A) | 112.6(13) |
| C(8)-C(16)-H(16B) | 112.6(12) |
| C(8)-C(16)-H(16C) | 109.2(12) |
| H(16A)-C(16)-H(16B) | 102.8(17) |
| H(16A)-C(16)-H(16C) | 110.6(17) |
| H(16B)-C(16)-H(16C) | 108.9(16) |
| C(18)-C(17)-C(13) | 114.35(14) |
| C(19)-C(17)-C(13) | 124.73(15) |
| C(19)-C(17)-C(18) | 120.88(16) |
| C(17)-C(18)-H(18A) | 110.7(13) |
| C(17)-C(18)-H(18B) | 110.1(12) |
| C(17)-C(18)-H(18C) | 109.0(14) |
| H(18A)-C(18)-H(18B) | 107.1(18) |
| H(18A)-C(18)-H(18C) | 107.8(18) |
| H(18B)-C(18)-H(18C) | 112.1(17) |
| C(17)-C(19)-H(19A) | 118.4(14) |
| C(17)-C(19)-H(19B) | 122.6(15) |
| H(19A)-C(19)-H(19B) | 119(2) |

Symmetry transformations used to generate equivalent atoms:

*Table 5. Anisotropic displacement parameters ($Å^2x10^3$) for Epoxide **36**. The anisotropic displacement factor exponent takes the form: $-2\pi^2[ h^2a^{*2}U^{11} + ... + 2hka^*b^*U^{12}]$.*

| | $U^{11}$ | $U^{22}$ | $U^{33}$ | $U^{23}$ | $U^{13}$ | $U^{12}$ |
|---|---|---|---|---|---|---|
| O(1) | 194(5) | 141(5) | 216(5) | -23(4) | 1(4) | -37(4) |
| O(2) | 261(6) | 241(6) | 228(5) | -109(4) | 20(5) | -44(5) |
| O(3) | 135(5) | 224(5) | 176(5) | -14(4) | -40(4) | 36(4) |
| O(4) | 115(5) | 294(6) | 226(5) | -25(4) | -17(4) | 11(4) |
| O(5) | 362(7) | 157(5) | 238(5) | 21(4) | -73(5) | 38(5) |
| C(1) | 123(6) | 145(6) | 169(6) | -27(5) | -3(5) | 3(5) |
| C(2) | 111(6) | 138(6) | 152(6) | 0(5) | -26(5) | -14(5) |
| C(3) | 98(5) | 99(6) | 167(6) | -5(5) | -20(5) | 1(5) |
| C(4) | 103(6) | 115(6) | 154(6) | -11(5) | -19(5) | 2(5) |
| C(5) | 197(7) | 118(6) | 170(6) | 2(5) | -23(6) | 17(5) |
| C(6) | 309(9) | 172(7) | 207(7) | 3(5) | -1(6) | 128(7) |
| C(7) | 244(8) | 148(6) | 213(7) | -27(5) | 10(6) | 50(6) |
| C(8) | 176(7) | 186(7) | 164(6) | -39(5) | -15(5) | -15(6) |
| C(9) | 192(7) | 265(8) | 152(6) | -20(5) | -33(6) | 16(6) |
| C(10) | 154(6) | 159(7) | 170(6) | 21(5) | -20(5) | -6(5) |
| C(11) | 146(6) | 131(6) | 183(6) | -29(5) | -34(5) | 31(5) |
| C(12) | 130(6) | 161(6) | 168(6) | -19(5) | -10(5) | 29(5) |
| C(13) | 139(6) | 177(7) | 153(6) | 1(5) | 5(5) | 10(5) |
| C(14) | 195(7) | 201(7) | 179(6) | 19(6) | 4(6) | -37(6) |
| C(15) | 210(7) | 128(6) | 199(6) | -1(5) | -32(6) | -49(6) |
| C(16) | 177(7) | 264(8) | 202(7) | -39(6) | 23(6) | 4(6) |
| C(17) | 186(7) | 178(7) | 119(5) | 6(5) | 6(5) | -23(6) |
| C(18) | 330(9) | 184(7) | 276(8) | -7(6) | -68(7) | -44(7) |
| C(19) | 185(7) | 263(9) | 294(8) | -41(7) | -57(6) | -7(6) |

*Table 6. Hydrogen coordinates (x10[4]) and isotropic displacement parameters (Å[2]x10[3]) for Epoxide **36**.*

| | x | y | z | U(eq) |
|---|---|---|---|---|
| H(2) | 6290(50) | 3280(20) | 3652(11) | 50(7) |
| H(2A) | 9360(40) | 6063(19) | 4700(10) | 37(6) |
| H(3) | 6760(30) | 6945(15) | 5272(7) | 13(4) |
| H(4) | 8620(30) | 5757(15) | 5899(7) | 8(4) |
| H(5) | 5490(30) | 3966(16) | 5724(8) | 18(5) |
| H(6A) | 10040(30) | 3871(16) | 5545(8) | 21(5) |
| H(6B) | 8650(30) | 2724(17) | 5610(8) | 24(5) |
| H(7) | 8570(30) | 2753(16) | 4627(7) | 14(4) |
| H(9A) | 7380(30) | 6011(17) | 3323(8) | 24(5) |
| H(9B) | 5420(40) | 5320(19) | 3527(9) | 29(5) |
| H(10) | 6820(30) | 7182(16) | 4126(7) | 13(4) |
| H(12A) | 5870(30) | 6992(17) | 6239(8) | 18(4) |
| H(12B) | 4240(30) | 5975(16) | 6195(8) | 20(5) |
| H(13) | 5190(30) | 6103(15) | 7144(7) | 14(4) |
| H(14A) | 6340(30) | 4104(16) | 7310(8) | 19(4) |
| H(14B) | 4330(40) | 4230(20) | 6848(10) | 40(6) |
| H(16A) | 11450(40) | 4137(19) | 3839(9) | 31(5) |
| H(16B) | 11090(30) | 5461(18) | 3832(8) | 24(5) |
| H(16C) | 10680(30) | 4740(16) | 3273(9) | 23(5) |
| H(18A) | 8190(30) | 7938(19) | 6859(10) | 36(6) |
| H(18B) | 9970(40) | 7730(18) | 7331(10) | 34(6) |
| H(18C) | 7410(40) | 7700(20) | 7496(10) | 42(6) |
| H(19A) | 11370(40) | 5870(20) | 7323(9) | 35(5) |
| H(19B) | 10090(40) | 4700(20) | 7118(9) | 36(6) |

*Table 7. Torsion angles [°] for Epoxide **36**.*

| | |
|---|---|
| O(1)-C(1)-C(2)-C(3) | 25.82(17) |
| O(1)-C(1)-C(2)-C(10) | -84.19(13) |
| O(1)-C(1)-C(7)-C(6) | -103.18(18) |
| O(1)-C(1)-C(8)-O(2) | -35.68(17) |
| O(1)-C(1)-C(8)-C(9) | 86.65(14) |
| O(1)-C(1)-C(8)-C(16) | -154.33(12) |
| O(2)-C(8)-C(9)-C(10) | 143.34(13) |
| C(1)-O(1)-C(7)-C(6) | 116.27(15) |
| C(1)-C(2)-C(3)-C(4) | 61.50(17) |
| C(1)-C(2)-C(3)-C(11) | -73.68(14) |
| C(1)-C(2)-C(10)-O(3) | 89.58(12) |
| C(1)-C(2)-C(10)-C(9) | -26.26(15) |
| C(1)-C(8)-C(9)-C(10) | 22.25(15) |
| C(2)-C(1)-C(7)-O(1) | 100.62(16) |
| C(2)-C(1)-C(7)-C(6) | -2.6(3) |
| C(2)-C(1)-C(8)-O(2) | -162.97(12) |
| C(2)-C(1)-C(8)-C(9) | -40.64(14) |
| C(2)-C(1)-C(8)-C(16) | 78.38(15) |
| C(2)-C(3)-C(4)-C(5) | -59.89(17) |
| C(2)-C(3)-C(4)-C(12) | 173.39(12) |
| C(2)-C(3)-C(11)-O(3) | -27.05(14) |
| C(2)-C(3)-C(11)-O(4) | 157.47(15) |
| C(3)-C(2)-C(10)-O(3) | -31.42(13) |
| C(3)-C(2)-C(10)-C(9) | -147.27(12) |
| C(3)-C(4)-C(5)-C(6) | 64.81(17) |
| C(3)-C(4)-C(5)-C(15) | -171.66(12) |
| C(3)-C(4)-C(12)-C(13) | -174.53(12) |
| C(4)-C(3)-C(11)-O(3) | -163.99(11) |
| C(4)-C(3)-C(11)-O(4) | 20.5(2) |
| C(4)-C(5)-C(6)-C(7) | -75.40(18) |
| C(4)-C(5)-C(15)-O(5) | -137.70(15) |
| C(4)-C(5)-C(15)-C(14) | 44.60(18) |
| C(4)-C(12)-C(13)-C(14) | -58.76(16) |
| C(4)-C(12)-C(13)-C(17) | 69.16(16) |

*Table 7. (cont'd)*

| | |
|---|---|
| C(5)-C(4)-C(12)-C(13) | 56.30(15) |
| C(5)-C(6)-C(7)-O(1) | -13.7(2) |
| C(5)-C(6)-C(7)-C(1) | 56.5(2) |
| C(6)-C(5)-C(15)-O(5) | -10.2(2) |
| C(6)-C(5)-C(15)-C(14) | 172.10(14) |
| C(7)-O(1)-C(1)-C(2) | -119.91(14) |
| C(7)-O(1)-C(1)-C(8) | 117.21(14) |
| C(7)-C(1)-C(2)-C(3) | -45.2(2) |
| C(7)-C(1)-C(2)-C(10) | -155.26(15) |
| C(7)-C(1)-C(8)-O(2) | 33.4(2) |
| C(7)-C(1)-C(8)-C(9) | 155.75(15) |
| C(7)-C(1)-C(8)-C(16) | -85.23(18) |
| C(8)-C(1)-C(2)-C(3) | 151.80(12) |
| C(8)-C(1)-C(2)-C(10) | 41.79(14) |
| C(8)-C(1)-C(7)-O(1) | -99.12(15) |
| C(8)-C(1)-C(7)-C(6) | 157.70(15) |
| C(8)-C(9)-C(10)-O(3) | -109.06(13) |
| C(8)-C(9)-C(10)-C(2) | 2.41(16) |
| C(10)-O(3)-C(11)-O(4) | -176.63(13) |
| C(10)-O(3)-C(11)-C(3) | 7.35(15) |
| C(10)-C(2)-C(3)-C(4) | 170.06(12) |
| C(10)-C(2)-C(3)-C(11) | 34.87(13) |
| C(11)-O(3)-C(10)-C(2) | 15.50(14) |
| C(11)-O(3)-C(10)-C(9) | 130.07(12) |
| C(11)-C(3)-C(4)-C(5) | 67.78(16) |
| C(11)-C(3)-C(4)-C(12) | -58.94(15) |
| C(12)-C(4)-C(5)-C(6) | -169.87(12) |
| C(12)-C(4)-C(5)-C(15) | -46.34(16) |
| C(12)-C(13)-C(14)-C(15) | 53.98(16) |
| C(12)-C(13)-C(17)-C(18) | 71.11(16) |
| C(12)-C(13)-C(17)-C(19) | -111.20(17) |
| C(13)-C(14)-C(15)-O(5) | 132.62(15) |
| C(13)-C(14)-C(15)-C(5) | -49.66(18) |
| C(14)-C(13)-C(17)-C(18) | -164.76(13) |

*Table 7. (cont'd)*

| | |
|---|---|
| C(14)-C(13)-C(17)-C(19) | 12.9(2) |
| C(15)-C(5)-C(6)-C(7) | 159.27(14) |
| C(16)-C(8)-C(9)-C(10) | -96.06(15) |
| C(17)-C(13)-C(14)-C(15) | -72.57(16) |

Symmetry transformations used to generate equivalent atoms:

*Table 8. Hydrogen bonds for Epoxide **36** [Å and °].*

| D-H...A | d(D-H) | d(H...A) | d(D...A) | <(DHA) |
|---|---|---|---|---|
| O(2)-H(2)...O(5)#1 | 0.87(3) | 2.37(3) | 3.1244(19) | 145(2) |
| C(2)-H(2A)...O(4)#2 | 0.97(2) | 2.44(2) | 3.2790(18) | 144.5(17) |
| C(3)-H(3)...O(3)#3 | 0.970(17) | 2.688(17) | 3.5378(17) | 146.5(13) |
| C(6)-H(6B)...O(1)#4 | 0.944(19) | 2.39(2) | 3.0758(19) | 129.3(15) |
| C(16)-H(16B)...O(3)#2 | 0.98(2) | 2.61(2) | 3.427(2) | 140.8(16) |

Symmetry transformations used to generate equivalent atoms:

#1 x-1/2,-y+1/2,-z+1 #2 x+1,y,z #3 x+1/2,-y+3/2,-z+1

#4 x+1/2,-y+1/2,-z+1

## X-Ray Crystal Structure Analysis of 2*H*-*ent*-Ineleganolide (37)

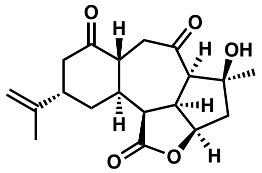


***2H-ent-Ineleganolide***
***37***

Contents

Table 1. Experimental Details

Table 2. Crystal Data

Table 3. Atomic Coordinates

Table 4. Full Bond Distances and Angles

Table 5. Anisotropic Displacement Parameters

Table 6. Hydrogen Atomic Coordinates

Table 7. Torsion Angles

Table 8. Hydrogen Bond Distances and Angles

*X-Ray Crystal Structure of 2H-ent-Ineleganolide (**37**)*

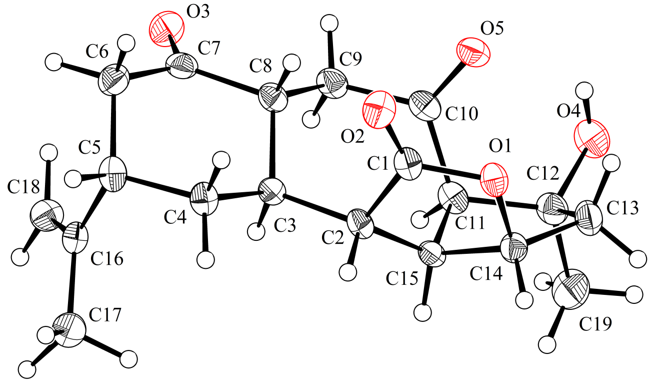


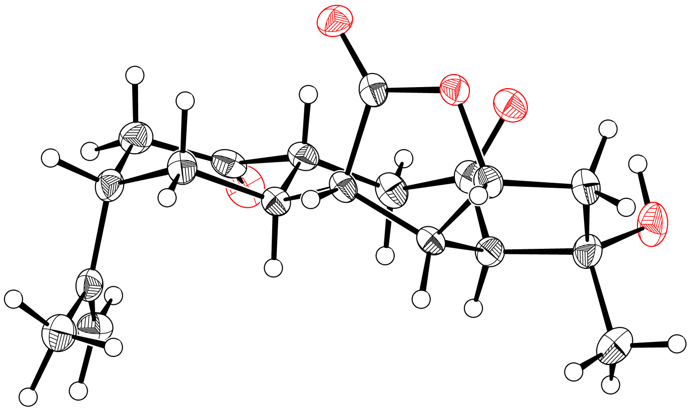

*Table 1. Experimental Details for X-Ray Structure Determination of 2H-ent-Ineleganolide (**37**).*

Low-temperature diffraction data ($\phi$-and $\omega$-scans) were collected on a Bruker AXS KAPPA APEX II diffractometer coupled to a APEX II CCD detector with graphite monochromated Mo $K_\alpha$ radiation ($\lambda$ = 0.71073 Å) for the structure of 2*H-ent*-ineleganolide (**37**) and on a Bruker AXS D8 VENTURE KAPPA diffractometer coupled to a PHOTON 100 CMOS detector with Cu $K_\alpha$ radiation ($\lambda$ = 1.54178 Å) from an I$\mu$S micro-source for the structure of compound P15149 and P15156. The structure was solved by direct methods using SHELXS[i] and refined against $F^2$ on all data by full-matrix least squares with SHELXL-2014[ii] using established refinement techniques.[iii] All non-hydrogen atoms were refined anisotropically. Unless otherwise noted, all hydrogen atoms were included into the model at geometrically calculated positions and refined using a riding model. The isotropic displacement parameters of all hydrogen atoms were fixed to 1.2 times the *U* value of the atoms they are linked to (1.5 times for methyl groups).

2*H-ent*-Ineleganolide (**37**) crystallizes in the orthorhombic space group $P2_12_12_1$ with two molecules in the asymmetric unit. The coordinates for all hydrogen atoms were located in the difference Fourier synthesis and refined freely.

*Table 2. Crystal Data and Structure Refinement for of 2H-ent-Ineleganolide (**37**).*

| | | |
|---|---|---|
| Caltech Identification code | p15149 | |
| CCDC Deposition Number | 1061016 | |
| Empirical formula | C19 H24 O5 | |
| Formula weight | 332.38 | |
| Temperature | 100 K | |
| Wavelength | 1.54178 Å | |
| Crystal system | Orthorhombic | |
| Space group | $P2_12_12_1$ | |
| Unit cell dimensions | a = 10.4079(3) Å | α= 90° |
| | b = 14.7042(4) Å | β= 90° |
| | c = 21.8592(6) Å | γ = 90° |
| Volume | 3345.33(16) $Å^3$ | |
| Z | 8 | |
| Density (calculated) | 1.320 $Mg/m^3$ | |
| Absorption coefficient | 0.776 $mm^{-1}$ | |
| F(000) | 1424 | |
| Crystal size | 0.11 x 0.10 x 0.05 $mm^3$ | |
| Theta range for data collection | 3.623 to 79.108°. | |
| Index ranges | $-12 \le h \le 13, -18 \le k \le 18, -27 \le l \le 27$ | |
| Reflections collected | 46504 | |
| Independent reflections | 7174 [R(int) = 0.0796] | |
| Completeness to theta = 66.500° | 100.0 % | |
| Absorption correction | Semi-empirical from equivalents | |
| Max. and min. transmission | 0.9591 and 0.8890 | |
| Refinement method | Full-matrix least-squares on $F^2$ | |
| Data / restraints / parameters | 7174 / 0 / 625 | |
| Goodness-of-fit on $F^2$ | 1.059 | |
| Final R indices [I>2sigma(I)] | R1 = 0.0395, wR2 = 0.0780 | |
| R indices (all data) | R1 = 0.0533, wR2 = 0.0832 | |
| Absolute structure parameter | 0.00(9) | |
| Extinction coefficient | n/a | |
| Largest diff. peak and hole | 0.164 and -0.204 e.$Å^{-3}$ | |

*Table 3. Atomic coordinates (x $10^4$) and equivalent isotropic displacement parameters ($Å^2$x $10^3$) for 2H-ent-Ineleganolide (**37**). U(eq) is defined as one third of the trace of the orthogonalized $U^{ij}$ tensor.*

| | x | y | z | U(eq) |
|---|---|---|---|---|
| O(5) | -3492(18) | 56047(13) | 60617(9) | 257(4) |
| O(2) | 5550(20) | 41629(12) | 48143(9) | 272(5) |
| O(1) | 15419(18) | 40753(12) | 57144(8) | 224(4) |
| O(4) | 8710(20) | 60085(16) | 71685(10) | 344(5) |
| O(3) | -5710(20) | 77121(13) | 43577(10) | 317(5) |
| C(15) | 26550(30) | 54941(17) | 57380(12) | 186(5) |
| C(11) | 18080(30) | 61455(18) | 61256(12) | 217(6) |
| C(10) | 4620(30) | 61450(18) | 58779(12) | 215(6) |
| C(9) | 1640(30) | 67917(19) | 53641(13) | 239(6) |
| C(8) | 4060(30) | 63525(18) | 47397(13) | 209(6) |
| C(3) | 17970(30) | 60187(17) | 46347(12) | 183(5) |
| C(2) | 22450(30) | 52704(17) | 50825(11) | 189(5) |
| C(1) | 13390(30) | 44637(17) | 51635(12) | 205(5) |
| C(14) | 25330(30) | 45637(18) | 60602(12) | 216(5) |
| C(13) | 20580(30) | 47320(20) | 67087(13) | 270(6) |
| C(12) | 19300(30) | 57710(20) | 67840(12) | 253(6) |
| C(19) | 31090(40) | 61710(30) | 70917(15) | 386(8) |
| C(7) | -280(30) | 70064(19) | 42368(13) | 236(6) |
| C(6) | 2140(30) | 67000(20) | 35906(13) | 258(6) |
| C(5) | 16110(30) | 63940(18) | 34952(12) | 228(6) |
| C(4) | 19450(30) | 56703(18) | 39723(12) | 225(6) |
| C(16) | 25680(30) | 71734(18) | 34857(12) | 216(6) |
| C(18) | 22410(30) | 80350(20) | 35596(13) | 255(6) |
| C(17) | 39360(30) | 69060(20) | 33625(15) | 283(6) |
| O(5B) | 59070(20) | 50022(12) | 60452(9) | 262(4) |
| O(2B) | 40407(18) | 34681(13) | 49667(9) | 239(4) |
| O(1B) | 48604(18) | 29282(13) | 58299(8) | 230(4) |
| O(4B) | 76480(20) | 44953(14) | 70004(9) | 308(5) |

*Table 3. (cont'd)*

| | | | | |
|---|---|---|---|---|
| O(3B) | 73160(20) | 59914(14) | 40281(9) | 348(5) |
| C(15B) | 71090(30) | 30487(17) | 56261(12) | 189(5) |
| C(11B) | 76280(30) | 39347(18) | 59232(12) | 207(5) |
| C(10B) | 68140(30) | 47391(17) | 57380(12) | 214(5) |
| C(9B) | 71380(30) | 51781(19) | 51349(13) | 257(6) |
| C(8B) | 64360(30) | 47390(18) | 45923(12) | 197(5) |
| C(3B) | 67320(30) | 37204(18) | 44942(12) | 196(5) |
| C(2B) | 63390(30) | 30974(17) | 50267(12) | 184(5) |
| C(1B) | 49680(30) | 31980(16) | 52432(12) | 198(5) |
| C(14B) | 61090(30) | 26871(18) | 60892(13) | 216(6) |
| C(13B) | 63060(30) | 31860(20) | 66917(13) | 237(6) |
| C(12B) | 75770(30) | 37126(18) | 66164(12) | 224(6) |
| C(19B) | 87110(30) | 31200(20) | 68043(15) | 315(7) |
| C(7B) | 67240(30) | 52828(18) | 40098(12) | 232(6) |
| C(6B) | 61770(30) | 49090(20) | 34202(13) | 250(6) |
| C(5B) | 64620(30) | 38920(20) | 33323(12) | 247(6) |
| C(4B) | 60490(30) | 33846(19) | 39122(13) | 229(6) |
| C(16B) | 78200(30) | 36610(20) | 31480(12) | 292(7) |
| C(18B) | 87410(30) | 42570(30) | 30882(16) | 402(8) |
| C(17B) | 80480(40) | 26660(20) | 30363(15) | 382(8) |

*Table 4. Bond lengths [Å] and angles [°] for 2H-ent-Ineleganolide (**37**).*

| | |
|---|---|
| O(5)-C(10) | 1.227(3) |
| O(2)-C(1) | 1.201(3) |
| O(1)-C(1) | 1.349(3) |
| O(1)-C(14) | 1.467(3) |
| O(4)-H(4) | 0.94(5) |
| O(4)-C(12) | 1.429(3) |
| O(3)-C(7) | 1.211(3) |
| C(15)-H(15) | 0.96(3) |
| C(15)-C(11) | 1.553(4) |
| C(15)-C(2) | 1.531(4) |
| C(15)-C(14) | 1.544(3) |
| C(11)-H(11) | 0.97(3) |
| C(11)-C(10) | 1.503(4) |
| C(11)-C(12) | 1.546(4) |
| C(10)-C(9) | 1.504(4) |
| C(9)-H(9A) | 1.00(4) |
| C(9)-H(9B) | 0.96(3) |
| C(9)-C(8) | 1.531(4) |
| C(8)-H(8) | 0.94(3) |
| C(8)-C(3) | 1.545(4) |
| C(8)-C(7) | 1.529(4) |
| C(3)-H(3) | 1.00(3) |
| C(3)-C(2) | 1.545(3) |
| C(3)-C(4) | 1.544(4) |
| C(2)-H(2) | 0.96(3) |
| C(2)-C(1) | 1.526(4) |
| C(14)-H(14) | 0.97(3) |
| C(14)-C(13) | 1.522(4) |
| C(13)-H(13A) | 0.99(3) |
| C(13)-H(13B) | 0.96(3) |
| C(13)-C(12) | 1.542(4) |
| C(12)-C(19) | 1.518(4) |
| C(19)-H(19A) | 0.98(4) |

*Table 4. (cont'd)*

| | |
|---|---|
| C(19)-H(19B) | 1.02(4) |
| C(19)-H(19C) | 0.98(4) |
| C(7)-C(6) | 1.504(4) |
| C(6)-H(6A) | 1.03(4) |
| C(6)-H(6B) | 0.94(4) |
| C(6)-C(5) | 1.536(4) |
| C(5)-H(5) | 1.03(3) |
| C(5)-C(4) | 1.530(4) |
| C(5)-C(16) | 1.519(4) |
| C(4)-H(4A) | 1.00(3) |
| C(4)-H(4B) | 1.00(3) |
| C(16)-C(18) | 1.322(4) |
| C(16)-C(17) | 1.501(4) |
| C(18)-H(18A) | 0.98(3) |
| C(18)-H(18B) | 1.02(3) |
| C(17)-H(17A) | 1.03(4) |
| C(17)-H(17B) | 1.07(4) |
| C(17)-H(17C) | 0.99(4) |
| O(5B)-C(10B) | 1.221(3) |
| O(2B)-C(1B) | 1.206(3) |
| O(1B)-C(1B) | 1.347(3) |
| O(1B)-C(14B) | 1.461(3) |
| O(4B)-H(4BA) | 0.90(4) |
| O(4B)-C(12B) | 1.426(3) |
| O(3B)-C(7B) | 1.211(3) |
| C(15B)-H(15B) | 0.96(3) |
| C(15B)-C(11B) | 1.552(4) |
| C(15B)-C(2B) | 1.538(4) |
| C(15B)-C(14B) | 1.547(4) |
| C(11B)-H(11B) | 0.91(3) |
| C(11B)-C(10B) | 1.510(4) |
| C(11B)-C(12B) | 1.551(4) |
| C(10B)-C(9B) | 1.506(4) |
| C(9B)-H(9BA) | 0.95(3) |

*Table 4. (cont'd)*

| | |
|---|---|
| C(9B)-H(9BB) | 0.99(3) |
| C(9B)-C(8B) | 1.535(4) |
| C(8B)-H(8B) | 0.97(3) |
| C(8B)-C(3B) | 1.544(4) |
| C(8B)-C(7B) | 1.533(4) |
| C(3B)-H(3B) | 1.00(3) |
| C(3B)-C(2B) | 1.537(4) |
| C(3B)-C(4B) | 1.539(4) |
| C(2B)-H(2B) | 0.99(3) |
| C(2B)-C(1B) | 1.511(4) |
| C(14B)-H(14B) | 0.98(3) |
| C(14B)-C(13B) | 1.522(4) |
| C(13B)-H(13C) | 1.02(3) |
| C(13B)-H(13D) | 1.02(3) |
| C(13B)-C(12B) | 1.541(4) |
| C(12B)-C(19B) | 1.523(4) |
| C(19B)-H(19D) | 0.92(4) |
| C(19B)-H(19E) | 0.98(4) |
| C(19B)-H(19F) | 1.00(3) |
| C(7B)-C(6B) | 1.512(4) |
| C(6B)-H(6BA) | 1.00(3) |
| C(6B)-H(6BB) | 1.00(4) |
| C(6B)-C(5B) | 1.537(4) |
| C(5B)-H(5B) | 0.98(3) |
| C(5B)-C(4B) | 1.532(4) |
| C(5B)-C(16B) | 1.508(4) |
| C(4B)-H(4BB) | 0.94(3) |
| C(4B)-H(4BC) | 1.05(3) |
| C(16B)-C(18B) | 1.305(5) |
| C(16B)-C(17B) | 1.503(5) |
| C(18B)-H(18C) | 1.03(4) |
| C(18B)-H(18D) | 0.89(3) |
| C(17B)-H(17D) | 1.07(3) |
| C(17B)-H(17E) | 1.00(4) |

*Table 4. (cont'd)*

| | |
|---|---|
| C(17B)-H(17F) | 1.02(4) |
| | |
| C(1)-O(1)-C(14) | 111.3(2) |
| C(12)-O(4)-H(4) | 104(3) |
| C(11)-C(15)-H(15) | 108.3(17) |
| C(2)-C(15)-H(15) | 111.4(17) |
| C(2)-C(15)-C(11) | 119.0(2) |
| C(2)-C(15)-C(14) | 102.3(2) |
| C(14)-C(15)-H(15) | 110.6(17) |
| C(14)-C(15)-C(11) | 104.5(2) |
| C(15)-C(11)-H(11) | 108.4(18) |
| C(10)-C(11)-C(15) | 109.4(2) |
| C(10)-C(11)-H(11) | 107.4(18) |
| C(10)-C(11)-C(12) | 114.3(2) |
| C(12)-C(11)-C(15) | 104.0(2) |
| C(12)-C(11)-H(11) | 113.1(17) |
| O(5)-C(10)-C(11) | 121.6(2) |
| O(5)-C(10)-C(9) | 120.8(3) |
| C(11)-C(10)-C(9) | 117.4(2) |
| C(10)-C(9)-H(9A) | 110.1(19) |
| C(10)-C(9)-H(9B) | 109.0(18) |
| C(10)-C(9)-C(8) | 111.4(2) |
| H(9A)-C(9)-H(9B) | 108(3) |
| C(8)-C(9)-H(9A) | 109(2) |
| C(8)-C(9)-H(9B) | 109.5(18) |
| C(9)-C(8)-H(8) | 109.7(18) |
| C(9)-C(8)-C(3) | 114.9(2) |
| C(3)-C(8)-H(8) | 105.4(17) |
| C(7)-C(8)-C(9) | 109.1(2) |
| C(7)-C(8)-H(8) | 105.7(18) |
| C(7)-C(8)-C(3) | 111.7(2) |
| C(8)-C(3)-H(3) | 108.9(16) |
| C(2)-C(3)-C(8) | 114.5(2) |
| C(2)-C(3)-H(3) | 105.2(16) |

*Table 4. (cont'd)*

| | |
|---|---|
| C(4)-C(3)-C(8) | 109.8(2) |
| C(4)-C(3)-H(3) | 109.1(16) |
| C(4)-C(3)-C(2) | 109.1(2) |
| C(15)-C(2)-C(3) | 121.6(2) |
| C(15)-C(2)-H(2) | 106.2(16) |
| C(3)-C(2)-H(2) | 106.0(16) |
| C(1)-C(2)-C(15) | 103.3(2) |
| C(1)-C(2)-C(3) | 116.2(2) |
| C(1)-C(2)-H(2) | 101.4(16) |
| O(2)-C(1)-O(1) | 121.2(2) |
| O(2)-C(1)-C(2) | 129.2(2) |
| O(1)-C(1)-C(2) | 109.6(2) |
| O(1)-C(14)-C(15) | 104.9(2) |
| O(1)-C(14)-H(14) | 105.5(17) |
| O(1)-C(14)-C(13) | 109.3(2) |
| C(15)-C(14)-H(14) | 113.0(17) |
| C(13)-C(14)-C(15) | 107.9(2) |
| C(13)-C(14)-H(14) | 115.6(18) |
| C(14)-C(13)-H(13A) | 111.3(18) |
| C(14)-C(13)-H(13B) | 111.8(19) |
| C(14)-C(13)-C(12) | 106.8(2) |
| H(13A)-C(13)-H(13B) | 106(3) |
| C(12)-C(13)-H(13A) | 112.3(18) |
| C(12)-C(13)-H(13B) | 109.0(19) |
| O(4)-C(12)-C(11) | 113.4(2) |
| O(4)-C(12)-C(13) | 111.8(2) |
| O(4)-C(12)-C(19) | 105.5(2) |
| C(13)-C(12)-C(11) | 105.1(2) |
| C(19)-C(12)-C(11) | 109.9(2) |
| C(19)-C(12)-C(13) | 111.2(3) |
| C(12)-C(19)-H(19A) | 108(2) |
| C(12)-C(19)-H(19B) | 113(2) |
| C(12)-C(19)-H(19C) | 108(2) |
| H(19A)-C(19)-H(19B) | 109(3) |

*Table 4. (cont'd)*

| | |
|---|---|
| H(19A)-C(19)-H(19C) | 110(3) |
| H(19B)-C(19)-H(19C) | 109(3) |
| O(3)-C(7)-C(8) | 121.3(3) |
| O(3)-C(7)-C(6) | 122.7(3) |
| C(6)-C(7)-C(8) | 115.9(2) |
| C(7)-C(6)-H(6A) | 110(2) |
| C(7)-C(6)-H(6B) | 108(2) |
| C(7)-C(6)-C(5) | 112.0(2) |
| H(6A)-C(6)-H(6B) | 107(3) |
| C(5)-C(6)-H(6A) | 111(2) |
| C(5)-C(6)-H(6B) | 109(2) |
| C(6)-C(5)-H(5) | 109.8(17) |
| C(4)-C(5)-C(6) | 109.0(2) |
| C(4)-C(5)-H(5) | 106.5(17) |
| C(16)-C(5)-C(6) | 113.7(2) |
| C(16)-C(5)-H(5) | 104.9(17) |
| C(16)-C(5)-C(4) | 112.7(2) |
| C(3)-C(4)-H(4A) | 107.4(17) |
| C(3)-C(4)-H(4B) | 109.2(17) |
| C(5)-C(4)-C(3) | 112.7(2) |
| C(5)-C(4)-H(4A) | 111.0(17) |
| C(5)-C(4)-H(4B) | 108.6(17) |
| H(4A)-C(4)-H(4B) | 108(2) |
| C(18)-C(16)-C(5) | 123.6(3) |
| C(18)-C(16)-C(17) | 121.1(3) |
| C(17)-C(16)-C(5) | 115.3(2) |
| C(16)-C(18)-H(18A) | 121.9(19) |
| C(16)-C(18)-H(18B) | 122.2(18) |
| H(18A)-C(18)-H(18B) | 116(3) |
| C(16)-C(17)-H(17A) | 110(2) |
| C(16)-C(17)-H(17B) | 113(2) |
| C(16)-C(17)-H(17C) | 113(2) |
| H(17A)-C(17)-H(17B) | 105(3) |
| H(17A)-C(17)-H(17C) | 110(3) |

*Table 4. (cont'd)*

| | |
|---|---|
| H(17B)-C(17)-H(17C) | 105(3) |
| C(1B)-O(1B)-C(14B) | 111.5(2) |
| C(12B)-O(4B)-H(4BA) | 102(2) |
| C(11B)-C(15B)-H(15B) | 107.5(16) |
| C(2B)-C(15B)-H(15B) | 111.5(15) |
| C(2B)-C(15B)-C(11B) | 119.9(2) |
| C(2B)-C(15B)-C(14B) | 102.9(2) |
| C(14B)-C(15B)-H(15B) | 110.1(15) |
| C(14B)-C(15B)-C(11B) | 104.4(2) |
| C(15B)-C(11B)-H(11B) | 109.2(18) |
| C(10B)-C(11B)-C(15B) | 110.5(2) |
| C(10B)-C(11B)-H(11B) | 110.0(18) |
| C(10B)-C(11B)-C(12B) | 114.1(2) |
| C(12B)-C(11B)-C(15B) | 102.7(2) |
| C(12B)-C(11B)-H(11B) | 110.1(18) |
| O(5B)-C(10B)-C(11B) | 122.3(3) |
| O(5B)-C(10B)-C(9B) | 121.2(3) |
| C(9B)-C(10B)-C(11B) | 116.4(2) |
| C(10B)-C(9B)-H(9BA) | 110.0(17) |
| C(10B)-C(9B)-H(9BB) | 107.7(18) |
| C(10B)-C(9B)-C(8B) | 112.9(2) |
| H(9BA)-C(9B)-H(9BB) | 110(3) |
| C(8B)-C(9B)-H(9BA) | 108.2(18) |
| C(8B)-C(9B)-H(9BB) | 107.7(18) |
| C(9B)-C(8B)-H(8B) | 111.1(17) |
| C(9B)-C(8B)-C(3B) | 114.9(2) |
| C(3B)-C(8B)-H(8B) | 106.2(17) |
| C(7B)-C(8B)-C(9B) | 109.2(2) |
| C(7B)-C(8B)-H(8B) | 104.4(17) |
| C(7B)-C(8B)-C(3B) | 110.6(2) |
| C(8B)-C(3B)-H(3B) | 109.2(16) |
| C(2B)-C(3B)-C(8B) | 114.8(2) |
| C(2B)-C(3B)-H(3B) | 107.6(16) |
| C(2B)-C(3B)-C(4B) | 108.2(2) |

*Table 4. (cont'd)*

| | |
|---|---|
| C(4B)-C(3B)-C(8B) | 109.5(2) |
| C(4B)-C(3B)-H(3B) | 107.3(16) |
| C(15B)-C(2B)-H(2B) | 105.6(18) |
| C(3B)-C(2B)-C(15B) | 122.3(2) |
| C(3B)-C(2B)-H(2B) | 107.1(17) |
| C(1B)-C(2B)-C(15B) | 103.3(2) |
| C(1B)-C(2B)-C(3B) | 115.5(2) |
| C(1B)-C(2B)-H(2B) | 100.5(18) |
| O(2B)-C(1B)-O(1B) | 120.5(2) |
| O(2B)-C(1B)-C(2B) | 129.1(2) |
| O(1B)-C(1B)-C(2B) | 110.4(2) |
| O(1B)-C(14B)-C(15B) | 105.2(2) |
| O(1B)-C(14B)-H(14B) | 107(2) |
| O(1B)-C(14B)-C(13B) | 109.8(2) |
| C(15B)-C(14B)-H(14B) | 111(2) |
| C(13B)-C(14B)-C(15B) | 108.0(2) |
| C(13B)-C(14B)-H(14B) | 115(2) |
| C(14B)-C(13B)-H(13C) | 114.2(18) |
| C(14B)-C(13B)-H(13D) | 109.1(16) |
| C(14B)-C(13B)-C(12B) | 105.4(2) |
| H(13C)-C(13B)-H(13D) | 108(2) |
| C(12B)-C(13B)-H(13C) | 111.9(18) |
| C(12B)-C(13B)-H(13D) | 108.0(15) |
| O(4B)-C(12B)-C(11B) | 113.8(2) |
| O(4B)-C(12B)-C(13B) | 112.7(2) |
| O(4B)-C(12B)-C(19B) | 105.2(2) |
| C(13B)-C(12B)-C(11B) | 103.9(2) |
| C(19B)-C(12B)-C(11B) | 110.9(2) |
| C(19B)-C(12B)-C(13B) | 110.4(2) |
| C(12B)-C(19B)-H(19D) | 110(2) |
| C(12B)-C(19B)-H(19E) | 114(2) |
| C(12B)-C(19B)-H(19F) | 108.8(18) |
| H(19D)-C(19B)-H(19E) | 109(3) |
| H(19D)-C(19B)-H(19F) | 108(3) |

*Table 4. (cont'd)*

| | |
|---|---|
| H(19E)-C(19B)-H(19F) | 108(3) |
| O(3B)-C(7B)-C(8B) | 121.4(2) |
| O(3B)-C(7B)-C(6B) | 122.2(3) |
| C(6B)-C(7B)-C(8B) | 116.4(2) |
| C(7B)-C(6B)-H(6BA) | 106.7(17) |
| C(7B)-C(6B)-H(6BB) | 107.4(19) |
| C(7B)-C(6B)-C(5B) | 112.8(2) |
| H(6BA)-C(6B)-H(6BB) | 110(3) |
| C(5B)-C(6B)-H(6BA) | 114.7(17) |
| C(5B)-C(6B)-H(6BB) | 105.2(19) |
| C(6B)-C(5B)-H(5B) | 106.9(19) |
| C(4B)-C(5B)-C(6B) | 108.4(2) |
| C(4B)-C(5B)-H(5B) | 108.1(19) |
| C(16B)-C(5B)-C(6B) | 115.7(3) |
| C(16B)-C(5B)-H(5B) | 105(2) |
| C(16B)-C(5B)-C(4B) | 112.0(2) |
| C(3B)-C(4B)-H(4BB) | 108.3(19) |
| C(3B)-C(4B)-H(4BC) | 107.6(15) |
| C(5B)-C(4B)-C(3B) | 113.4(2) |
| C(5B)-C(4B)-H(4BB) | 109.7(18) |
| C(5B)-C(4B)-H(4BC) | 109.8(15) |
| H(4BB)-C(4B)-H(4BC) | 108(2) |
| C(18B)-C(16B)-C(5B) | 124.3(3) |
| C(18B)-C(16B)-C(17B) | 121.4(3) |
| C(17B)-C(16B)-C(5B) | 114.2(3) |
| C(16B)-C(18B)-H(18C) | 123(2) |
| C(16B)-C(18B)-H(18D) | 124(2) |
| H(18C)-C(18B)-H(18D) | 112(3) |
| C(16B)-C(17B)-H(17D) | 111.6(18) |
| C(16B)-C(17B)-H(17E) | 112(2) |
| C(16B)-C(17B)-H(17F) | 110(2) |
| H(17D)-C(17B)-H(17E) | 107(3) |
| H(17D)-C(17B)-H(17F) | 112(3) |

*Table 4. (cont'd)*

| | |
|---|---|
| H(17E)-C(17B)-H(17F) | 104(3) |

Symmetry transformations used to generate equivalent atoms:

*Table 5. Anisotropic displacement parameters (Å$^2$x10$^3$) for 2H-ent-Ineleganolide (**37**). The anisotropic displacement factor exponent takes the form: $-2\pi^2[ h^2a^{*2}U^{11} + ... + 2hka^*b^*U^{12}]$.*

| | $U^{11}$ | $U^{22}$ | $U^{33}$ | $U^{23}$ | $U^{13}$ | $U^{12}$ |
|---|---|---|---|---|---|---|
| O(5) | 208(10) | 281(10) | 281(10) | 1(9) | 44(8) | 2(8) |
| O(2) | 355(12) | 181(9) | 278(11) | -13(8) | -41(8) | -37(8) |
| O(1) | 284(10) | 179(9) | 208(9) | 7(8) | 18(8) | -22(8) |
| O(4) | 364(13) | 446(13) | 222(11) | -58(10) | 89(9) | 54(11) |
| O(3) | 312(12) | 266(11) | 373(13) | 61(9) | 22(9) | 104(9) |
| C(15) | 167(13) | 185(12) | 207(13) | 15(10) | 36(10) | -4(10) |
| C(11) | 259(15) | 191(13) | 201(13) | -42(11) | 42(11) | -20(11) |
| C(10) | 226(13) | 200(12) | 219(13) | -58(11) | 74(10) | 30(11) |
| C(9) | 229(16) | 221(13) | 267(15) | 8(11) | 64(11) | 47(12) |
| C(8) | 213(14) | 176(12) | 237(14) | 7(11) | 27(10) | -22(11) |
| C(3) | 190(13) | 155(11) | 203(13) | 3(10) | 21(10) | 12(10) |
| C(2) | 219(13) | 148(11) | 199(13) | 3(10) | 63(10) | 15(10) |
| C(1) | 265(15) | 150(11) | 201(13) | -12(10) | 52(11) | 37(10) |
| C(14) | 227(14) | 211(12) | 210(13) | 29(11) | 10(11) | 12(11) |
| C(13) | 305(16) | 301(15) | 204(14) | 39(12) | 26(11) | -14(13) |
| C(12) | 255(15) | 309(15) | 196(13) | -45(11) | 31(11) | -8(12) |
| C(19) | 410(20) | 480(20) | 267(17) | -45(16) | -71(14) | -89(17) |
| C(7) | 167(13) | 232(13) | 310(15) | 41(12) | 3(11) | -26(11) |
| C(6) | 252(16) | 263(14) | 260(16) | 21(12) | -45(11) | -15(12) |
| C(5) | 317(16) | 183(12) | 183(13) | -16(11) | 6(11) | -8(12) |
| C(4) | 283(15) | 185(12) | 207(13) | 1(11) | 24(11) | 20(11) |
| C(16) | 254(15) | 227(13) | 166(13) | 19(10) | -11(11) | 6(11) |
| C(18) | 252(16) | 218(13) | 296(15) | 1(12) | -19(11) | -21(12) |
| C(17) | 300(16) | 279(15) | 272(16) | -15(13) | 8(12) | 30(14) |
| O(5B) | 318(11) | 205(9) | 264(10) | -40(8) | 20(8) | 50(8) |
| O(2B) | 179(10) | 255(9) | 282(10) | -41(8) | -45(7) | -18(8) |
| O(1B) | 200(10) | 267(10) | 223(10) | 1(8) | 10(8) | -25(8) |
| O(4B) | 413(13) | 305(11) | 205(10) | -40(8) | -60(9) | -18(10) |
| O(3B) | 443(13) | 300(11) | 299(11) | 48(9) | 18(9) | -128(10) |
| C(15B) | 189(13) | 167(12) | 210(13) | -19(11) | 18(10) | 33(11) |

*Table 5. (cont'd)*

| | | | | | | |
|---|---|---|---|---|---|---|
| C(11B) | 163(13) | 241(13) | 216(13) | -12(10) | 17(10) | -11(11) |
| C(10B) | 256(14) | 170(12) | 217(13) | -59(11) | -29(11) | -55(11) |
| C(9B) | 333(17) | 211(13) | 227(14) | -1(11) | -21(12) | -72(12) |
| C(8B) | 194(13) | 189(13) | 208(13) | -5(11) | -12(10) | -21(11) |
| C(3B) | 192(14) | 205(13) | 192(13) | -16(10) | 2(10) | 6(11) |
| C(2B) | 217(14) | 153(12) | 181(13) | -30(10) | -5(10) | 9(10) |
| C(1B) | 232(14) | 142(11) | 220(13) | -56(10) | 16(11) | -44(11) |
| C(14B) | 224(14) | 189(13) | 234(14) | 22(11) | -6(11) | 9(11) |
| C(13B) | 258(15) | 260(14) | 194(14) | 26(11) | 23(11) | 4(12) |
| C(12B) | 226(14) | 249(13) | 198(13) | -24(11) | -9(10) | 4(11) |
| C(19B) | 254(17) | 402(19) | 289(17) | 34(15) | -36(12) | 43(14) |
| C(7B) | 228(14) | 224(13) | 244(14) | 19(11) | 11(11) | 5(11) |
| C(6B) | 296(16) | 275(15) | 180(14) | 4(12) | -2(11) | 50(13) |
| C(5B) | 256(15) | 307(15) | 179(13) | -24(12) | -49(11) | 43(12) |
| C(4B) | 287(16) | 213(14) | 188(13) | -33(11) | -34(11) | -2(11) |
| C(16B) | 313(16) | 423(17) | 139(13) | -7(12) | -26(11) | 107(14) |
| C(18B) | 336(19) | 520(20) | 347(18) | -35(17) | 49(14) | 9(17) |
| C(17B) | 460(20) | 449(19) | 240(16) | -29(14) | -45(15) | 209(17) |

*Table 6. Hydrogen coordinates (x10$^4$) and isotropic displacement parameters (Å$^2$x10$^3$) for 2H-ent-Ineleganolide (**37**).*

| | x | y | z | U(eq) |
|---|---|---|---|---|
| H(4) | 170(40) | 5710(30) | 6990(20) | 75(15) |
| H(15) | 3530(30) | 5708(19) | 5753(13) | 17(7) |
| H(11) | 2140(30) | 6760(20) | 6082(13) | 23(8) |
| H(9A) | 700(30) | 7350(20) | 5401(15) | 36(9) |
| H(9B) | -720(30) | 6980(20) | 5393(14) | 26(8) |
| H(8) | -110(30) | 5833(19) | 4697(13) | 19(7) |
| H(3) | 2400(30) | 6536(19) | 4701(12) | 18(7) |
| H(2) | 2970(30) | 4986(18) | 4893(12) | 14(7) |
| H(14) | 3310(30) | 4190(20) | 6025(13) | 21(7) |
| H(13A) | 2650(30) | 4460(20) | 7015(14) | 26(8) |
| H(13B) | 1240(30) | 4460(20) | 6779(14) | 29(9) |
| H(19A) | 3000(30) | 6830(20) | 7103(15) | 38(9) |
| H(19B) | 3940(40) | 6020(30) | 6863(19) | 54(12) |
| H(19C) | 3160(30) | 5920(20) | 7510(17) | 42(10) |
| H(6A) | -30(40) | 7210(30) | 3291(17) | 54(12) |
| H(6B) | -330(40) | 6210(20) | 3506(16) | 38(10) |
| H(5) | 1700(30) | 6090(20) | 3072(14) | 24(8) |
| H(4A) | 2850(30) | 5460(20) | 3924(13) | 20(7) |
| H(4B) | 1360(30) | 5130(20) | 3910(14) | 23(8) |
| H(18A) | 2870(30) | 8530(20) | 3529(14) | 28(8) |
| H(18B) | 1310(30) | 8230(20) | 3630(14) | 28(8) |
| H(17A) | 4340(40) | 6650(30) | 3753(18) | 56(12) |
| H(17B) | 4540(40) | 7470(20) | 3236(16) | 44(10) |
| H(17C) | 4030(40) | 6470(30) | 3024(18) | 51(11) |
| H(4BA) | 6990(40) | 4840(30) | 6858(18) | 50(12) |
| H(15B) | 7820(30) | 2630(17) | 5594(11) | 10(6) |
| H(11B) | 8460(30) | 4023(19) | 5804(13) | 18(7) |
| H(9BA) | 8040(30) | 5140(19) | 5062(13) | 21(8) |
| H(9BB) | 6870(30) | 5820(20) | 5157(14) | 29(8) |
| H(8B) | 5510(30) | 4784(19) | 4640(13) | 19(7) |

*Table 6. (cont'd)*

| | | | | |
|---|---|---|---|---|
| H(3B) | 7680(30) | 3641(19) | 4428(12) | 18(7) |
| H(2B) | 6320(30) | 2470(20) | 4867(13) | 24(8) |
| H(14B) | 6150(30) | 2020(20) | 6113(15) | 37(9) |
| H(13C) | 6320(30) | 2770(20) | 7065(15) | 29(9) |
| H(13D) | 5590(30) | 3649(18) | 6748(12) | 12(7) |
| H(19D) | 9470(40) | 3410(20) | 6706(15) | 34(9) |
| H(19E) | 8710(40) | 2510(30) | 6616(17) | 45(11) |
| H(19F) | 8690(30) | 3040(20) | 7259(15) | 28(8) |
| H(6BA) | 6500(30) | 5310(20) | 3082(14) | 25(8) |
| H(6BB) | 5220(40) | 4950(20) | 3449(15) | 37(9) |
| H(5B) | 5920(30) | 3680(20) | 2994(15) | 32(9) |
| H(4BB) | 6220(30) | 2760(20) | 3867(13) | 23(8) |
| H(4BC) | 5050(30) | 3464(19) | 3980(12) | 19(7) |
| H(18C) | 9650(40) | 4090(30) | 2945(18) | 59(12) |
| H(18D) | 8630(30) | 4860(20) | 3113(14) | 21(8) |
| H(17D) | 8130(30) | 2300(20) | 3454(15) | 39(9) |
| H(17E) | 8870(40) | 2560(20) | 2804(16) | 35(9) |
| H(17F) | 7350(40) | 2410(20) | 2756(17) | 50(11) |

*Table 7. Torsion angles [°] for 2H-ent-Ineleganolide (**37**).*

| | |
|---|---|
| O(5)-C(10)-C(9)-C(8) | -86.1(3) |
| O(1)-C(14)-C(13)-C(12) | -115.2(2) |
| O(3)-C(7)-C(6)-C(5) | 131.8(3) |
| C(15)-C(11)-C(10)-O(5) | 89.7(3) |
| C(15)-C(11)-C(10)-C(9) | -86.3(3) |
| C(15)-C(11)-C(12)-O(4) | -155.9(2) |
| C(15)-C(11)-C(12)-C(13) | -33.5(3) |
| C(15)-C(11)-C(12)-C(19) | 86.2(3) |
| C(15)-C(2)-C(1)-O(2) | 162.0(3) |
| C(15)-C(2)-C(1)-O(1) | -19.2(3) |
| C(15)-C(14)-C(13)-C(12) | -1.6(3) |
| C(11)-C(15)-C(2)-C(3) | 45.8(3) |
| C(11)-C(15)-C(2)-C(1) | -86.9(3) |
| C(11)-C(15)-C(14)-O(1) | 97.4(2) |
| C(11)-C(15)-C(14)-C(13) | -19.1(3) |
| C(11)-C(10)-C(9)-C(8) | 90.0(3) |
| C(10)-C(11)-C(12)-O(4) | -36.7(3) |
| C(10)-C(11)-C(12)-C(13) | 85.7(3) |
| C(10)-C(11)-C(12)-C(19) | -154.5(3) |
| C(10)-C(9)-C(8)-C(3) | -58.9(3) |
| C(10)-C(9)-C(8)-C(7) | 174.8(2) |
| C(9)-C(8)-C(3)-C(2) | 61.9(3) |
| C(9)-C(8)-C(3)-C(4) | -175.0(2) |
| C(9)-C(8)-C(7)-O(3) | -5.7(4) |
| C(9)-C(8)-C(7)-C(6) | 176.6(2) |
| C(8)-C(3)-C(2)-C(15) | -77.8(3) |
| C(8)-C(3)-C(2)-C(1) | 49.4(3) |
| C(8)-C(3)-C(4)-C(5) | 57.8(3) |
| C(8)-C(7)-C(6)-C(5) | -50.5(3) |
| C(3)-C(8)-C(7)-O(3) | -133.8(3) |
| C(3)-C(8)-C(7)-C(6) | 48.5(3) |
| C(3)-C(2)-C(1)-O(2) | 26.3(4) |
| C(3)-C(2)-C(1)-O(1) | -155.0(2) |
| C(2)-C(15)-C(11)-C(10) | 23.1(3) |

*Table 7. (cont'd)*

| | |
|---|---|
| C(2)-C(15)-C(11)-C(12) | 145.6(2) |
| C(2)-C(15)-C(14)-O(1) | -27.4(3) |
| C(2)-C(15)-C(14)-C(13) | -143.9(2) |
| C(2)-C(3)-C(4)-C(5) | -176.0(2) |
| C(1)-O(1)-C(14)-C(15) | 16.8(3) |
| C(1)-O(1)-C(14)-C(13) | 132.3(2) |
| C(14)-O(1)-C(1)-O(2) | -179.6(2) |
| C(14)-O(1)-C(1)-C(2) | 1.5(3) |
| C(14)-C(15)-C(11)-C(10) | -90.2(3) |
| C(14)-C(15)-C(11)-C(12) | 32.3(3) |
| C(14)-C(15)-C(2)-C(3) | 160.3(2) |
| C(14)-C(15)-C(2)-C(1) | 27.7(3) |
| C(14)-C(13)-C(12)-O(4) | 145.3(2) |
| C(14)-C(13)-C(12)-C(11) | 21.9(3) |
| C(14)-C(13)-C(12)-C(19) | -97.0(3) |
| C(12)-C(11)-C(10)-O(5) | -26.4(4) |
| C(12)-C(11)-C(10)-C(9) | 157.6(2) |
| C(7)-C(8)-C(3)-C(2) | -173.2(2) |
| C(7)-C(8)-C(3)-C(4) | -50.1(3) |
| C(7)-C(6)-C(5)-C(4) | 53.9(3) |
| C(7)-C(6)-C(5)-C(16) | -72.7(3) |
| C(6)-C(5)-C(4)-C(3) | -59.3(3) |
| C(6)-C(5)-C(16)-C(18) | 0.8(4) |
| C(6)-C(5)-C(16)-C(17) | -177.1(2) |
| C(4)-C(3)-C(2)-C(15) | 158.7(2) |
| C(4)-C(3)-C(2)-C(1) | -74.1(3) |
| C(4)-C(5)-C(16)-C(18) | -123.9(3) |
| C(4)-C(5)-C(16)-C(17) | 58.2(3) |
| C(16)-C(5)-C(4)-C(3) | 67.9(3) |
| O(5B)-C(10B)-C(9B)-C(8B) | -89.2(3) |
| O(1B)-C(14B)-C(13B)-C(12B) | -124.2(2) |
| O(3B)-C(7B)-C(6B)-C(5B) | 133.4(3) |
| C(15B)-C(11B)-C(10B)-O(5B) | 92.9(3) |
| C(15B)-C(11B)-C(10B)-C(9B) | -84.2(3) |

*Table 7. (cont'd)*

| | |
|---|---|
| C(15B)-C(11B)-C(12B)-O(4B) | -162.8(2) |
| C(15B)-C(11B)-C(12B)-C(13B) | -39.8(3) |
| C(15B)-C(11B)-C(12B)-C(19B) | 78.9(3) |
| C(15B)-C(2B)-C(1B)-O(2B) | 161.2(3) |
| C(15B)-C(2B)-C(1B)-O(1B) | -19.2(3) |
| C(15B)-C(14B)-C(13B)-C(12B) | -10.0(3) |
| C(11B)-C(15B)-C(2B)-C(3B) | 42.2(4) |
| C(11B)-C(15B)-C(2B)-C(1B) | -90.0(3) |
| C(11B)-C(15B)-C(14B)-O(1B) | 102.5(2) |
| C(11B)-C(15B)-C(14B)-C(13B) | -14.7(3) |
| C(11B)-C(10B)-C(9B)-C(8B) | 88.0(3) |
| C(10B)-C(11B)-C(12B)-O(4B) | -43.2(3) |
| C(10B)-C(11B)-C(12B)-C(13B) | 79.8(3) |
| C(10B)-C(11B)-C(12B)-C(19B) | -161.6(2) |
| C(10B)-C(9B)-C(8B)-C(3B) | -60.4(3) |
| C(10B)-C(9B)-C(8B)-C(7B) | 174.7(2) |
| C(9B)-C(8B)-C(3B)-C(2B) | 62.1(3) |
| C(9B)-C(8B)-C(3B)-C(4B) | -175.9(2) |
| C(9B)-C(8B)-C(7B)-O(3B) | -6.6(4) |
| C(9B)-C(8B)-C(7B)-C(6B) | 176.2(2) |
| C(8B)-C(3B)-C(2B)-C(15B) | -75.4(3) |
| C(8B)-C(3B)-C(2B)-C(1B) | 51.7(3) |
| C(8B)-C(3B)-C(4B)-C(5B) | 59.9(3) |
| C(8B)-C(7B)-C(6B)-C(5B) | -49.3(3) |
| C(3B)-C(8B)-C(7B)-O(3B) | -133.9(3) |
| C(3B)-C(8B)-C(7B)-C(6B) | 48.8(3) |
| C(3B)-C(2B)-C(1B)-O(2B) | 25.0(4) |
| C(3B)-C(2B)-C(1B)-O(1B) | -155.3(2) |
| C(2B)-C(15B)-C(11B)-C(10B) | 25.7(3) |
| C(2B)-C(15B)-C(11B)-C(12B) | 147.7(2) |
| C(2B)-C(15B)-C(14B)-O(1B) | -23.4(2) |
| C(2B)-C(15B)-C(14B)-C(13B) | -140.6(2) |
| C(2B)-C(3B)-C(4B)-C(5B) | -174.3(2) |
| C(1B)-O(1B)-C(14B)-C(15B) | 12.6(3) |

*Table 7. (cont'd)*

| | |
|---|---|
| C(1B)-O(1B)-C(14B)-C(13B) | 128.6(2) |
| C(14B)-O(1B)-C(1B)-O(2B) | -176.1(2) |
| C(14B)-O(1B)-C(1B)-C(2B) | 4.2(3) |
| C(14B)-C(15B)-C(11B)-C(10B) | -88.7(2) |
| C(14B)-C(15B)-C(11B)-C(12B) | 33.3(3) |
| C(14B)-C(15B)-C(2B)-C(3B) | 157.4(2) |
| C(14B)-C(15B)-C(2B)-C(1B) | 25.2(2) |
| C(14B)-C(13B)-C(12B)-O(4B) | 154.6(2) |
| C(14B)-C(13B)-C(12B)-C(11B) | 30.9(3) |
| C(14B)-C(13B)-C(12B)-C(19B) | -88.1(3) |
| C(12B)-C(11B)-C(10B)-O(5B) | -22.2(4) |
| C(12B)-C(11B)-C(10B)-C(9B) | 160.7(2) |
| C(7B)-C(8B)-C(3B)-C(2B) | -173.7(2) |
| C(7B)-C(8B)-C(3B)-C(4B) | -51.7(3) |
| C(7B)-C(6B)-C(5B)-C(4B) | 51.5(3) |
| C(7B)-C(6B)-C(5B)-C(16B) | -75.3(3) |
| C(6B)-C(5B)-C(4B)-C(3B) | -58.7(3) |
| C(6B)-C(5B)-C(16B)-C(18B) | 3.0(4) |
| C(6B)-C(5B)-C(16B)-C(17B) | -177.2(2) |
| C(4B)-C(3B)-C(2B)-C(15B) | 162.0(2) |
| C(4B)-C(3B)-C(2B)-C(1B) | -70.9(3) |
| C(4B)-C(5B)-C(16B)-C(18B) | -121.9(3) |
| C(4B)-C(5B)-C(16B)-C(17B) | 57.9(3) |
| C(16B)-C(5B)-C(4B)-C(3B) | 70.1(3) |

Symmetry transformations used to generate equivalent atoms:

*Table 8. Hydrogen bonds for 2H-ent-Ineleganolide (**37**) [Å and °].*

| D-H...A | d(D-H) | d(H...A) | d(D...A) | <(DHA) |
|---|---|---|---|---|
| O(4)-H(4)...O(5) | 0.94(5) | 2.11(5) | 2.796(3) | 129(4) |
| C(15)-H(15)...O(3)#1 | 0.96(3) | 2.52(3) | 3.227(3) | 131(2) |
| C(8)-H(8)...O(2) | 0.94(3) | 2.57(3) | 3.228(3) | 128(2) |
| C(2)-H(2)...O(2B) | 0.96(3) | 2.50(3) | 3.253(3) | 136(2) |
| C(14)-H(14)...O(1B) | 0.97(3) | 2.50(3) | 3.450(3) | 164(2) |
| O(4B)-H(4BA)...O(5B) | 0.90(4) | 2.12(4) | 2.863(3) | 139(3) |
| C(15B)-H(15B)...O(2B)#2 | 0.96(3) | 2.39(3) | 3.270(3) | 151(2) |
| C(8B)-H(8B)...O(2B) | 0.97(3) | 2.57(3) | 3.222(3) | 125(2) |
| C(2B)-H(2B)...O(2)#2 | 0.99(3) | 2.62(3) | 3.440(3) | 140(2) |
| C(2B)-H(2B)...O(1)#2 | 0.99(3) | 2.61(3) | 3.588(3) | 169(2) |
| C(13B)-H(13D)...O(5B) | 1.02(3) | 2.54(3) | 3.049(3) | 110.7(18) |
| C(6B)-H(6BA)...O(4B)#3 | 1.00(3) | 2.54(3) | 3.449(4) | 150(2) |

Symmetry transformations used to generate equivalent atoms:

#1 x+1/2,-y+3/2,-z+1 #2 x+1/2,-y+1/2,-z+1 #3 -x+3/2,-y+1,z-1/2
