# Supplementary material for: Correction: Enantioselective, convergent synthesis of the ineleganolide core by a tandem annulation cascade
Source: Chem Sci. 2019 Jan 14;10(4):1254–5. doi: 10.1039/c8sc90236d (PMC6349058; doi:10.1039/c8sc90236d)
Supplement: Supplementary file 1 [file SC-010-C8SC90236D-s001.pdf]

Electronic Supplementary Material (ESI) for Chemical Science.
This journal is © The Royal Society of Chemistry 2019

Correction: Enantioselective, Convergent Synthesis of the Ineleganolide Core by a Tandem Annulation Cascade

Robert A. Craig, II, Jennifer L. Roizen, Russell C. Smith,
Amanda C. Jones, Scott C. Virgil, and Brian M. Stoltz*

Warren and Katharine Schlinger Laboratory for Chemistry and Chemical Engineering, Division of Chemistry and Chemical Engineering, California Institute of Technology, MC 101-20, Pasadena, CA 91125, U.S.A.

Table of Contents

1. X-Ray Crystal Structure Analysis of Enone ***ent*-12A** S2

2. Notes & References S15

1. X-Ray Crystal Structure Analysis of Enone *ent*-12A

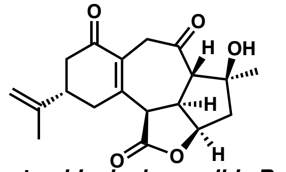

ent-epi-Isoineleganolide B
ent-12A

Contents

Table 1.1. Experimental Details

Table 1.2. Crystal Data

Table 1.3. Atomic Coordinates

Table 1.4. Full Bond Distances and Angles

Table 1.5. Anisotropic Displacement Parameters

Table 1.6. Hydrogen Atomic Coordinates

Table 1.7. Torsion Angles

*Figure 1.1. X-Ray Crystal Structure of Enone **ent-12A***

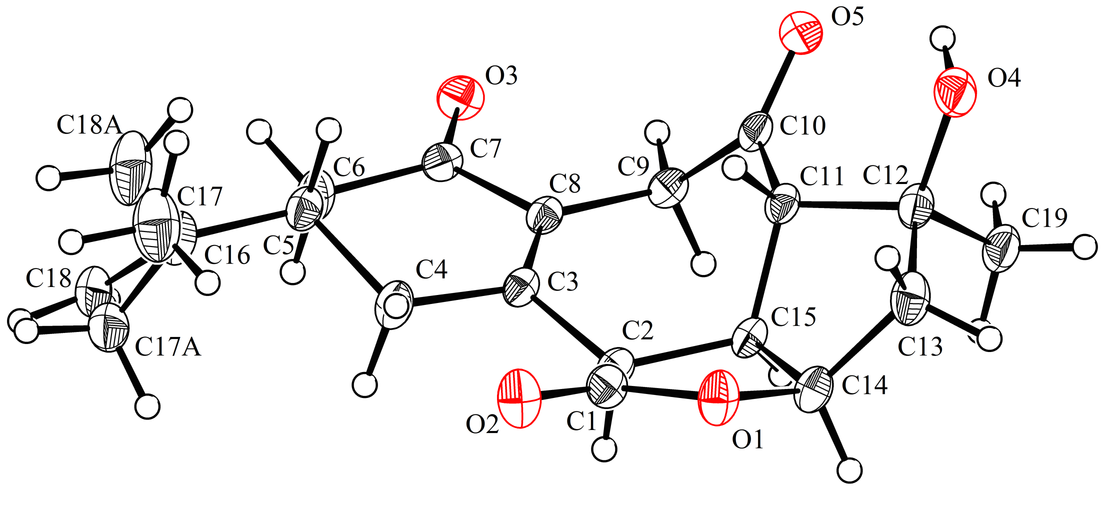

Table 1.1. Experimental Details for X-Ray Structure Determination of Enone ***ent-12A****.*

Low-temperature diffraction data (ϕ-and ω-scans) were collected on a Bruker AXS D8 VENTURE KAPPA diffractometer coupled to a PHOTON 100 CMOS detector with Cu $K\alpha$ radiation (λ = 1.54178 Å) from an IμS micro-source for the structure of *ent-epi*-Isoineleganolide B (***ent*-12A**). The structure was solved by direct methods using SHELXS[1] and refined against F^2 on all data by full-matrix least squares with SHELXL-2014[2] using established refinement techniques.[3] All non-hydrogen atoms were refined anisotropically. All hydrogen atoms were included into the model at geometrically calculated positions and refined using a riding model. The isotropic displacement parameters of all hydrogen atoms were fixed to 1.2 times the U value of the atoms they are linked to (1.5 times for methyl groups).

ent-epi-Isoineleganolide B (***ent*-12A**) crystallizes in the monoclinic space group *C2* with one molecule in the asymmetric unit. The isopropenyl group was disordered in the crystal (51.5:48.5) and the corresponding positions were labeled C17-C18 and C17A-C18A, respectively.

Table 1.2. *Crystal Data and Structure Refinement for Enone* ***ent-12A***.

Identification code	p17139	
Empirical formula	C19 H22 O5	
Formula weight	330.36	
Temperature	100 K	
Wavelength	1.54178 Å	
Crystal system	Monoclinic	
Space group	C 1 2 1	
Unit cell dimensions	a = 22.5468(19) Å	α = 90°
	b = 10.4722(9) Å	β = 107.751(5)°
	c = 7.3277(5) Å	γ = 90°
Volume	1647.8(2) Å3	
Z	4	
Density (calculated)	1.332 Mg/m3	
Absorption coefficient	0.787 mm-1	
F(000)	704	
Crystal size	0.32 x 0.13 x 0.06 mm3	
Theta range for data collection	4.698 to 79.209°.	
Index ranges	-28<=h<=28, -12<=k<=13, -9<=l<=9	
Reflections collected	19495	
Independent reflections	3531 [R(int) = 0.0367]	
Completeness to theta =	67.000° 99.9 %	
Absorption correction	Semi-empirical from equivalents	
Max. and min. transmission	1.0000 and 0.8733	
Refinement method	Full-matrix least-squares on F2	
Data / restraints / parameters	3531 / 1 / 240	
Goodness-of-fit on F2	1.048	
Final R indices	[I>2sigma(I)]R1 = 0.0274, wR2 = 0.0697	
R indices (all data)	R1 = 0.0277, wR2 = 0.0699	
Absolute structure parameter	0.06(4)	
Extinction coefficient	n/a	
Largest diff. peak and hole	0.212 and -0.159 e.Å-3	

Table 1.3. Atomic coordinates (x 10^4) and equivalent isotropic displacement parameters ($Å^2$x 10^3) for Enone ***ent-12A****. U(eq) is defined as one third of the trace of the orthogonalized U^{ij} tensor.*

	x	y	z	U(eq)
O(1)	23712(6)	35782(12)	113320(18)	263(3)
O(2)	33298(6)	33911(13)	111652(18)	289(3)
O(3)	26431(6)	67848(12)	31508(17)	249(3)
O(4)	5991(6)	32240(13)	65740(20)	301(3)
O(5)	10499(6)	49135(14)	39801(17)	295(3)
C(1)	28415(8)	39480(16)	106680(20)	215(3)
C(2)	26543(7)	51484(15)	94210(20)	180(3)
C(3)	29080(7)	53076(14)	77310(20)	173(3)
C(4)	35799(8)	49235(18)	81350(20)	236(3)
C(5)	37965(7)	48220(16)	63610(20)	211(3)
C(6)	36087(8)	60431(18)	52050(30)	256(4)
C(7)	29179(8)	62554(15)	46600(20)	192(3)
C(8)	25865(7)	58747(15)	60600(20)	173(3)
C(9)	19067(8)	62977(16)	54540(20)	192(3)
C(10)	14604(7)	52167(16)	54220(20)	197(3)
C(11)	15547(7)	45161(15)	72810(20)	184(3)
C(12)	9576(8)	41864(16)	77910(30)	220(3)
C(13)	12370(8)	36506(18)	98240(30)	268(4)
C(14)	18327(8)	44223(17)	106900(20)	223(3)
C(15)	19512(7)	52125(15)	90640(20)	178(3)
C(16)	44859(8)	45242(19)	69090(30)	277(4)
C(17)	46690(20)	32160(50)	76470(160)	440(20)
C(17A)	49280(60)	55260(140)	76100(140)	327(18)
C(18)	49260(50)	54390(110)	70230(140)	278(16)
C(18A)	46540(20)	33160(50)	65170(170)	430(20)
C(19)	5505(8)	53574(18)	77820(30)	262(4)

Table 1.4. Bond lengths [Å] and angles [°] for Enone ***ent-12A***.

O(1)-C(1)	1.351(2)
O(1)-C(14)	1.459(2)
O(2)-C(1)	1.200(2)
O(3)-C(7)	1.223(2)
O(4)-H(4)	0.8400
O(4)-C(12)	1.423(2)
O(5)-C(10)	1.215(2)
C(1)-C(2)	1.535(2)
C(2)-H(2)	1.0000
C(2)-C(3)	1.5237(19)
C(2)-C(15)	1.527(2)
C(3)-C(4)	1.507(2)
C(3)-C(8)	1.355(2)
C(4)-H(4A)	0.9900
C(4)-H(4B)	0.9900
C(4)-C(5)	1.525(2)
C(5)-H(5)	1.0000
C(5)-C(6)	1.521(2)
C(5)-C(16)	1.515(2)
C(6)-H(6A)	0.9900
C(6)-H(6B)	0.9900
C(6)-C(7)	1.502(2)
C(7)-C(8)	1.496(2)
C(8)-C(9)	1.526(2)
C(9)-H(9A)	0.9900
C(9)-H(9B)	0.9900
C(9)-C(10)	1.510(2)
C(10)-C(11)	1.504(2)
C(11)-H(11)	1.0000
C(11)-C(12)	1.542(2)
C(11)-C(15)	1.525(2)
C(12)-C(13)	1.536(2)
C(12)-C(19)	1.531(2)

Table 1.4. (cont'd)

C(13)-H(13A)	0.9900
C(13)-H(13B)	0.9900
C(13)-C(14)	1.530(2)
C(14)-H(14)	1.0000
C(14)-C(15)	1.540(2)
C(15)-H(15)	1.0000
C(16)-C(17)	1.485(6)
C(16)-C(17A)	1.430(13)
C(16)-C(18)	1.363(11)
C(16)-C(18A)	1.376(5)
C(17)-H(17A)	0.9800
C(17)-H(17B)	0.9800
C(17)-H(17C)	0.9800
C(17A)-H(17D)	0.9800
C(17A)-H(17E)	0.9800
C(17A)-H(17F)	0.9800
C(18)-H(18A)	0.9500
C(18)-H(18B)	0.9500
C(18A)-H(18C)	0.9500
C(18A)-H(18D)	0.9500
C(19)-H(19A)	0.9800
C(19)-H(19B)	0.9800
C(19)-H(19C)	0.9800
C(1)-O(1)-C(14)	112.18(12)
C(12)-O(4)-H(4)	109.5
O(1)-C(1)-C(2)	110.20(14)
O(2)-C(1)-O(1)	119.94(15)
O(2)-C(1)-C(2)	129.72(15)
C(1)-C(2)-H(2)	105.3
C(3)-C(2)-C(1)	118.02(13)
C(3)-C(2)-H(2)	105.3
C(3)-C(2)-C(15)	118.89(12)
C(15)-C(2)-C(1)	102.75(12)

Table 1.4. (cont'd)

C(15)-C(2)-H(2)	105.3
C(4)-C(3)-C(2)	114.52(13)
C(8)-C(3)-C(2)	123.54(13)
C(8)-C(3)-C(4)	121.56(13)
C(3)-C(4)-H(4A)	108.6
C(3)-C(4)-H(4B)	108.6
C(3)-C(4)-C(5)	114.51(13)
H(4A)-C(4)-H(4B)	107.6
C(5)-C(4)-H(4A)	108.6
C(5)-C(4)-H(4B)	108.6
C(4)-C(5)-H(5)	107.8
C(6)-C(5)-C(4)	107.99(13)
C(6)-C(5)-H(5)	107.8
C(16)-C(5)-C(4)	110.96(14)
C(16)-C(5)-H(5)	107.8
C(16)-C(5)-C(6)	114.19(14)
C(5)-C(6)-H(6A)	109.4
C(5)-C(6)-H(6B)	109.4
H(6A)-C(6)-H(6B)	108.0
C(7)-C(6)-C(5)	111.28(13)
C(7)-C(6)-H(6A)	109.4
C(7)-C(6)-H(6B)	109.4
O(3)-C(7)-C(6)	120.40(14)
O(3)-C(7)-C(8)	121.31(14)
C(8)-C(7)-C(6)	118.19(13)
C(3)-C(8)-C(7)	119.68(14)
C(3)-C(8)-C(9)	127.11(13)
C(7)-C(8)-C(9)	112.99(13)
C(8)-C(9)-H(9A)	109.0
C(8)-C(9)-H(9B)	109.0
H(9A)-C(9)-H(9B)	107.8
C(10)-C(9)-C(8)	113.11(13)
C(10)-C(9)-H(9A)	109.0
C(10)-C(9)-H(9B)	109.0

Table 1.4. *(cont'd)*

O(5)-C(10)-C(9)	122.45(15)
O(5)-C(10)-C(11)	121.31(15)
C(11)-C(10)-C(9)	116.24(13)
C(10)-C(11)-H(11)	107.4
C(10)-C(11)-C(12)	115.84(13)
C(10)-C(11)-C(15)	115.26(13)
C(12)-C(11)-H(11)	107.4
C(15)-C(11)-H(11)	107.4
C(15)-C(11)-C(12)	103.09(12)
O(4)-C(12)-C(11)	112.21(13)
O(4)-C(12)-C(13)	109.61(14)
O(4)-C(12)-C(19)	109.84(14)
C(13)-C(12)-C(11)	100.76(13)
C(19)-C(12)-C(11)	112.72(14)
C(19)-C(12)-C(13)	111.40(14)
C(12)-C(13)-H(13A)	110.8
C(12)-C(13)-H(13B)	110.8
H(13A)-C(13)-H(13B)	108.8
C(14)-C(13)-C(12)	104.96(13)
C(14)-C(13)-H(13A)	110.8
C(14)-C(13)-H(13B)	110.8
O(1)-C(14)-C(13)	110.66(14)
O(1)-C(14)-H(14)	111.3
O(1)-C(14)-C(15)	104.56(12)
C(13)-C(14)-H(14)	111.3
C(13)-C(14)-C(15)	107.48(14)
C(15)-C(14)-H(14)	111.3
C(2)-C(15)-C(14)	105.05(12)
C(2)-C(15)-H(15)	111.1
C(11)-C(15)-C(2)	115.63(12)
C(11)-C(15)-C(14)	102.20(13)
C(11)-C(15)-H(15)	111.1
C(14)-C(15)-H(15)	111.1
C(17)-C(16)-C(5)	115.8(3)

Table 1.4. (cont'd)

C(17A)-C(16)-C(5)	119.3(6)
C(18)-C(16)-C(5)	122.7(5)
C(18)-C(16)-C(17)	120.6(6)
C(18A)-C(16)-C(5)	117.4(3)
C(18A)-C(16)-C(17A)	122.9(6)
C(16)-C(17)-H(17A)	109.5
C(16)-C(17)-H(17B)	109.5
C(16)-C(17)-H(17C)	109.5
H(17A)-C(17)-H(17B)	109.5
H(17A)-C(17)-H(17C)	109.5
H(17B)-C(17)-H(17C)	109.5
C(16)-C(17A)-H(17D)	109.5
C(16)-C(17A)-H(17E)	109.5
C(16)-C(17A)-H(17F)	109.5
H(17D)-C(17A)-H(17E)	109.5
H(17D)-C(17A)-H(17F)	109.5
H(17E)-C(17A)-H(17F)	109.5
C(16)-C(18)-H(18A)	120.0
C(16)-C(18)-H(18B)	120.0
H(18A)-C(18)-H(18B)	120.0
C(16)-C(18A)-H(18C)	120.0
C(16)-C(18A)-H(18D)	120.0
H(18C)-C(18A)-H(18D)	120.0
C(12)-C(19)-H(19A)	109.5
C(12)-C(19)-H(19B)	109.5
C(12)-C(19)-H(19C)	109.5
H(19A)-C(19)-H(19B)	109.5
H(19A)-C(19)-H(19C)	109.5
H(19B)-C(19)-H(19C)	109.5

Symmetry transformations used to generate equivalent atoms:

*Table 1.5. Anisotropic displacement parameters ($Å^2x10^3$) for Enone **ent-12A**. The anisotropic displacement factor exponent takes the form: $-2\pi^2[h^2a^{*2}U^{11} + ... + 2hka^*b^*U^{12}]$.*

	U11	U22	U33	U23	U13
O(1)	255(6)	280(7)	290(6)	121(5)	137(5)
O(2)	255(6)	286(7)	341(7)	137(6)	113(5)
O(3)	293(6)	270(6)	195(5)	43(5)	87(5)
O(4)	262(6)	256(6)	421(7)	-99(6)	157(6)
O(5)	239(6)	416(8)	237(6)	-53(5)	83(5)
C(1)	231(8)	226(8)	203(7)	41(6)	89(6)
C(2)	209(7)	175(7)	175(7)	14(6)	88(6)
C(3)	209(7)	134(7)	207(7)	-3(6)	111(6)
C(4)	223(7)	282(9)	233(8)	59(6)	111(6)
C(5)	219(7)	198(7)	255(8)	-1(6)	129(6)
C(6)	239(8)	294(9)	281(8)	67(7)	146(7)
C(7)	236(8)	165(7)	194(7)	-1(6)	94(6)
C(8)	201(7)	144(7)	199(7)	-15(5)	97(6)
C(9)	228(8)	179(7)	184(7)	18(6)	84(6)
C(10)	188(7)	210(8)	222(7)	-33(6)	106(6)
C(11)	190(7)	153(7)	238(8)	-22(6)	106(6)
C(12)	210(8)	179(7)	314(8)	-38(6)	143(6)
C(13)	257(8)	243(8)	357(9)	44(7)	171(7)
C(14)	241(8)	226(8)	239(8)	35(6)	128(6)
C(15)	210(7)	162(7)	199(7)	8(6)	116(6)
C(16)	241(9)	297(9)	339(9)	39(7)	160(7)
C(17)	320(20)	340(20)	700(50)	90(30)	210(20)
C(17A)	230(20)	500(40)	270(40)	-10(40)	100(30)
C(18)	220(20)	350(30)	320(40)	0(40)	140(30)
C(18A)	320(20)	280(20)	760(60)	40(30)	290(30)
C(19)	226(8)	272(9)	326(9)	-36(7)	140(7)

Table 1.6. Hydrogen coordinates (x10^4) and isotropic displacement parameters (Å^{2}x10^3) for Enone ***ent-12A***.

	x	y	z	U(eq)
H(4)	518	3454	5426	45
H(2)	2830	5881	10295	22
H(4A)	3644	4087	8797	28
H(4B)	3845	5556	9021	28
H(5)	3567	4098	5565	25
H(6A)	3827	6778	5971	31
H(6B)	3737	5991	4031	31
H(9A)	1854	6964	6349	23
H(9B)	1801	6682	4160	23
H(11)	1769	3694	7192	22
H(13A)	945	3764	10582	32
H(13B)	1333	2731	9782	32
H(14)	1796	4983	11755	27
H(15)	1809	6115	9090	21
H(17A)	4567	3092	8843	66
H(17B)	5118	3104	7889	66
H(17C)	4443	2588	6697	66
H(17D)	4841	6230	6684	49
H(17E)	5349	5201	7788	49
H(17F)	4895	5835	8839	49
H(18A)	5355	5242	7579	33
H(18B)	4804	6273	6546	33
H(18C)	5080	3118	6711	51
H(18D)	4344	2680	6053	51
H(19A)	432	5755	6511	39
H(19B)	784	5972	8741	39
H(19C)	175	5095	8088	39

Table 1.7. Torsion angles [°] for Enone ***ent-12A****.*

O(1)-C(1)-C(2)-C(3)	-146.69(14)
O(1)-C(1)-C(2)-C(15)	-13.73(17)
O(1)-C(14)-C(15)-C(2)	-22.20(16)
O(1)-C(14)-C(15)-C(11)	98.89(14)
O(2)-C(1)-C(2)-C(3)	37.7(3)
O(2)-C(1)-C(2)-C(15)	170.70(18)
O(3)-C(7)-C(8)-C(3)	-178.99(15)
O(3)-C(7)-C(8)-C(9)	-3.9(2)
O(4)-C(12)-C(13)-C(14)	152.44(13)
O(5)-C(10)-C(11)-C(12)	-42.8(2)
O(5)-C(10)-C(11)-C(15)	-163.26(15)
C(1)-O(1)-C(14)-C(13)	129.81(15)
C(1)-O(1)-C(14)-C(15)	14.37(19)
C(1)-C(2)-C(3)-C(4)	-40.78(19)
C(1)-C(2)-C(3)-C(8)	146.15(15)
C(1)-C(2)-C(15)-C(11)	-90.37(15)
C(1)-C(2)-C(15)-C(14)	21.45(15)
C(2)-C(3)-C(4)-C(5)	168.14(13)
C(2)-C(3)-C(8)-C(7)	166.15(14)
C(2)-C(3)-C(8)-C(9)	-8.1(2)
C(3)-C(2)-C(15)-C(11)	42.1(2)
C(3)-C(2)-C(15)-C(14)	153.91(14)
C(3)-C(4)-C(5)-C(6)	50.14(18)
C(3)-C(4)-C(5)-C(16)	176.00(15)
C(3)-C(8)-C(9)-C(10)	-58.7(2)
C(4)-C(3)-C(8)-C(7)	-6.4(2)
C(4)-C(3)-C(8)-C(9)	179.26(15)
C(4)-C(5)-C(6)-C(7)	-57.90(18)
C(4)-C(5)-C(16)-C(17)	70.8(5)
C(4)-C(5)-C(16)-C(17A)	-78.4(5)
C(4)-C(5)-C(16)-C(18)	-98.9(5)
C(4)-C(5)-C(16)-C(18A)	108.1(6)
C(5)-C(6)-C(7)-O(3)	-147.67(16)
C(5)-C(6)-C(7)-C(8)	35.9(2)

Table 1.7. (cont'd)

C(6)-C(5)-C(16)-C(17)	-166.9(5)
C(6)-C(5)-C(16)-C(17A)	43.9(5)
C(6)-C(5)-C(16)-C(18)	23.4(5)
C(6)-C(5)-C(16)-C(18A)	-129.6(6)
C(6)-C(7)-C(8)-C(3)	-2.6(2)
C(6)-C(7)-C(8)-C(9)	172.42(15)
C(7)-C(8)-C(9)-C(10)	126.68(14)
C(8)-C(3)-C(4)-C(5)	-18.6(2)
C(8)-C(9)-C(10)-O(5)	-120.83(16)
C(8)-C(9)-C(10)-C(11)	58.53(17)
C(9)-C(10)-C(11)-C(12)	137.82(14)
C(9)-C(10)-C(11)-C(15)	17.37(19)
C(10)-C(11)-C(12)-O(4)	70.14(18)
C(10)-C(11)-C(12)-C(13)	-173.34(14)
C(10)-C(11)-C(12)-C(19)	-54.51(19)
C(10)-C(11)-C(15)-C(2)	-79.03(17)
C(10)-C(11)-C(15)-C(14)	167.49(13)
C(11)-C(12)-C(13)-C(14)	34.01(16)
C(12)-C(11)-C(15)-C(2)	153.77(13)
C(12)-C(11)-C(15)-C(14)	40.29(15)
C(12)-C(13)-C(14)-O(1)	-123.42(14)
C(12)-C(13)-C(14)-C(15)	-9.82(17)
C(13)-C(14)-C(15)-C(2)	-139.84(14)
C(13)-C(14)-C(15)-C(11)	-18.75(16)
C(14)-O(1)-C(1)-O(2)	175.66(16)
C(14)-O(1)-C(1)-C(2)	-0.42(19)
C(15)-C(2)-C(3)-C(4)	-166.18(14)
C(15)-C(2)-C(3)-C(8)	20.7(2)
C(15)-C(11)-C(12)-O(4)	-163.04(14)
C(15)-C(11)-C(12)-C(13)	-46.51(15)
C(15)-C(11)-C(12)-C(19)	72.32(17)
C(16)-C(5)-C(6)-C(7)	178.17(15)
C(19)-C(12)-C(13)-C(14)	-85.78(16)

2. Notes & References

1. Sheldrick, G. M. *Acta Cryst*. **1990**, A46, 467–473.

2. Sheldrick, G. M. *Acta Cryst*. **2008**, A64, 112–122.

3. Müller, P. *Crystallography Reviews* **2009**, *15*, 57–83.
